# Barcoding of Plant Viruses with Circular Single-Stranded DNA Based on Rolling Circle Amplification

**DOI:** 10.3390/v10090469

**Published:** 2018-08-31

**Authors:** Holger Jeske

**Affiliations:** Department of Molecular Biology and Plant Virology, Institute of Biomaterials and Biomolecular Systems, University of Stuttgart, Pfaffenwaldring 57, 70550 Stuttgart, Germany; holger.jeske@bio.uni-stuttgart.de; Tel.: +49-711-685-65070

**Keywords:** geminivirus, nanovirus, satellite, RCA, RFLP, Circomics

## Abstract

The experience with a diagnostic technology based on rolling circle amplification (RCA), restriction fragment length polymorphism (RFLP) analyses, and direct or deep sequencing (Circomics) over the past 15 years is surveyed for the plant infecting geminiviruses, nanoviruses and associated satellite DNAs, which have had increasing impact on agricultural and horticultural losses due to global transportation and recombination-aided diversification. Current state methods for quarantine measures are described to identify individual DNA components with great accuracy and to recognize the crucial role of the molecular viral population structure as an important factor for sustainable plant protection.

## 1. Introduction

The symptoms of plant viruses, later known to contain circular single-stranded DNA, were already described in ancient times: 752 ACE, in a poem, and 1868 ACE, in garden literature [1,2,3]. Only in more recent decades have they become devastating pests in agriculture and horticulture due to intensified farming and global transportation of plant material, as well as recombination and pseudo-recombination (reassortment of genomic components) of viruses [4]. Geminivirus genomes consist of one (“DNA A-like”) or two (“DNA A”, “DNA B”) DNA circles of, in total, 2500–5200 nucleotides (nt); nanovirus genomes consist of more (>6) components, each of a smaller size (900–1100 nt). Geminiviruses may have adopted DNAs from nanoviruses (named alpha-satellites, here abbreviated “aSat”) or of other unknown origin (especially the beta-satellites; “bSat”), and delta-satellites, which may or may not be essential for symptom development. For diagnostic purposes, it is necessary to emphasize that geminiviruses may be prone to generate defective DNAs (“D-DNA”) depending on the virus and the host plant. If D-DNAs interfere with the multiplication of the parental virus, which is not necessarily the case, they are also referred to as “defective interfering, DI-DNAs”. The detection of single-stranded (ssDNA) circles by RCA/restriction fragment length polymorphism (RFLP) in a plant is a first good diagnostic hint for a virus infection in most plants, but some plant species harbor small circular mitochondrial plasmids that may be misinterpreted as viral agents. Such plasmids might be useful as internal standards. For the practical purposes of plant protection, it may be necessary to discriminate between potentially infectious components and defective ones, which may occur as a cocktail in field isolates. Under certain circumstances, the accumulation of DI-DNAs may protect the plant from an infection by more severe viruses, and it may thus be a wrong decision to remove these plants, in particular for perennial species. For quarantine measures in general, however, it is more of a cautious measure to eradicate all circular viral ssDNAs from the plant material in stockbreeding. A few reports have appeared during the last years that discuss seed transmission of geminiviruses controversially [5,6,7]. In general, sexual crossing is an excellent way to purge breeding material. Vegetative propagation and grafting, however, promote virus dissemination very efficiently. More sophisticated diagnostics are necessary for these cases (such as cassava, sweet potato, and ornamentals), since the viruses may be distributed unequally within a plant and multiply differentially under certain environmental conditions (temperature and light intensity, quality, or period). In spite of some progress in the field of conventional breeding and genetic engineering to combat these viruses, quarantine measures to restrict their spread are still the most effective way for plant production. Immunological and PCR-based techniques have been widely used to detect the viruses, but rolling circle amplification (RCA), in combination with restriction fragment length polymorphism (RFLP) analyses, have become more popular during the last decade due to their simplicity, accuracy and lack of *a priori* knowledge, which will be explained in detail in this review.

The molecular biology of geminiviruses, nanoviruses, and their satellites has been reviewed previously [8,9,10,11,12,13,14,15]; a taxonomy profile for geminiviruses has been published recently [16] separating nine genera currently. The nomenclature of aSats has become more and more complex, with an ICTV-approved family *Alphasatellitidae* now including two subfamilies (*Geminialalphasatellitinae* and *Nanoalphasatellitinae*) [17].

## 2. The Broad Practical Application of RCA

A literature survey on the Web of Science for the years 2004 to 2018 yielded 146 papers in which RCA was mentioned explicitly for geminiviruses, mastreviruses, begomoviruses, curtoviruses, and nanoviruses [2,18,19,20,21,22,23,24,25,26,27,28,29,30,31,32,33,34,35,36,37,38,39,40,41,42,43,44,45,46,47,48,49,50,51,52,53,54,55,56,57,58,59,60,61,62,63,64,65,66,67,68,69,70,71,72,73,74,75,76,77,78,79,80,81,82,83,84,85,86,87,88,89,90,91,92,93,94,95,96,97,98,99,100,101,102,103,104,105,106,107,108,109,110,111,112,113,114,115,116,117,118,119,120,121,122,123,124,125,126,127,128,129,130,131,132,133,134,135,136,137,138,139,140,141,142,143,144,145,146,147,148,149,150,151,152,153,154,155,156,157,158,159,160]. It showed a representative sample for the broad application of the technique for various plant hosts, environmental specimens and viruses for various countries worldwide in fundamental as well as applied science, as summarized in Table S1. Many more excellent publications have appeared in this context, mainly in the field of classification and taxonomy of known and newly detected ssDNA viruses, as well as of resistance breeding and biotechnology, which cannot be cited here due to space limitations. The reader is referred to comprehensive overviews for those aspects [161,162,163,164,165,166,167,168,169,170]. The following review rather tries to focus on certain aspects of RCA diagnostics to describe the state-of-the-art technique and its unique advantages.

## 3. Sampling Is Simple

In particular for tropical and subtropical countries, where most geminiviruses and nanoviruses are pests, an important prerequisite for reliable virus detection is the collection of plant material and the preservation of its DNA. The simplest way to do this is by harvesting fresh young leaves without any sign of necrosis, if possible, and placing them immediately between two sheets of blotting paper to allow for slow drying at room temperature. The samples are stored dry at room temperature in the dark to avoid hydrolytic DNA base deamination and oxidation and they may be analyzed several years later for RCA/RFLP without any loss of resolution [2,26,72,96]. This easy technique has proven to be successful for various plant species, including woody perennials. More elaborate and expensive sampling and storage variants by means of Whatman FTA^TM^ cards have been used to avoid any risk of virus spread [22], but the dried samples of plant sap on simple paper may be generally regarded as safe, since insect vectors (whiteflies, leafhoppers, aphids) are needed for further spread of geminiviruses and nanoviruses, which will not feed on this material at all.

## 4. Extraction of Nucleic Acids

Clipping off a small disk from a dried leaf, e.g., with an Eppendorf tube lid, yields sufficient material for several RCA/RFLP analyses. Extraction of nucleic acids based on the cationic detergent cetyltrimethylammonium bromide (CTAB) is reliable for most plants, even those which are technically recalcitrant due to mucilage and other secondary plant components [19]. Including antioxidants may be necessary for certain plants that react with necrosis and browning, such as some vetch and potato variants. After chloroform extraction and alcohol precipitation, the sample is ready for RCA. Several attempts to increase the efficiency of the reaction by further purification of the nucleic acids failed to improve the detection [19]. Heating the sample to 65 °C for a short period (10 min) assists in dissolving the nucleic acids, denatures residual proteins, and minimizes the breakage of viral ssDNA rings. For double-stranded DNA (dsDNA) such as plasmids, protocols frequently recommend a heating step at 95 °C to nick one strand of the ring and denature the DNA to allow for hybridization of primers; this is, however, counterproductive for circular ssDNA viruses (see below). Most importantly, it generates a high background of RCA templates of the (linear) host DNA, which should be excluded from the further process.

## 5. Run-Off and De Novo Primed Replication

**Figure 1:**
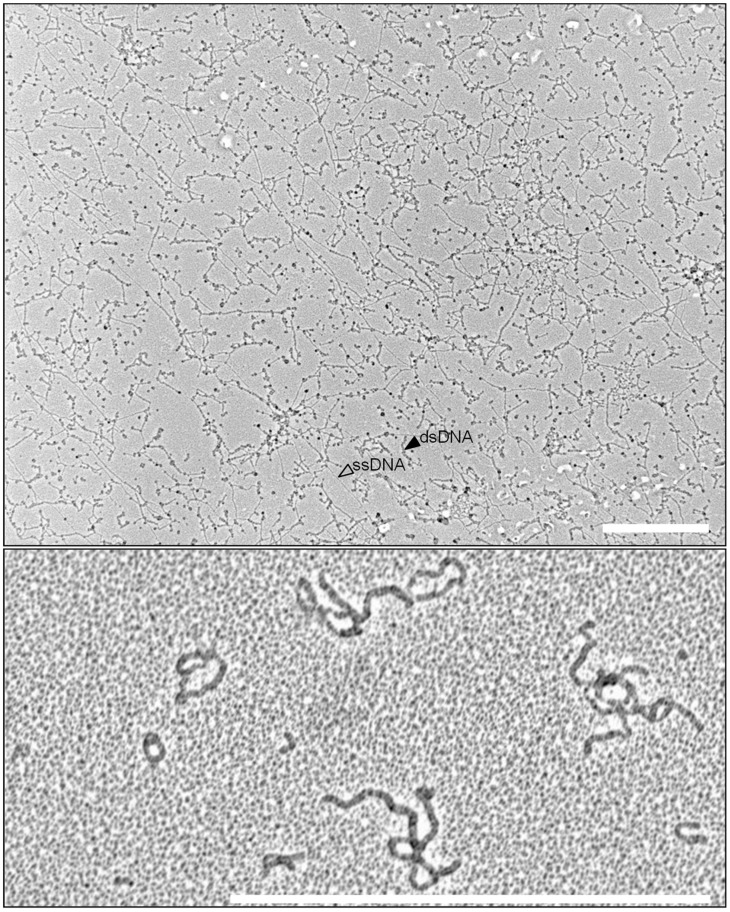
African Cassava Mosaic Virus TempliPhi rolling circle amplification (RCA) product spread on a cetyltrimethylammonium bromide (CTAB)-activated carbon film, contrast is obtained by Pt evaporation, shown at two magnifications (bar = 1 µm). Note the pinwheel structures, indicative of multiple-primed circular DNA (asterisk).

For an optimal usage of the RCA reaction, it is necessary to consider some basic prerequisites and peculiarities of geminiviruses and nanoviruses (Figure 1). In a leaf sample, the majority of the viral DNA is usually circular ssDNA. However, substantial amounts are engaged in replication, leading to heterogeneous ssDNA and dsDNA forms, as well as combinations of both. In one-dimensional gels subjected to Southern analysis with virus-specific probes, this DNA portion is underestimated because it forms a smear signal after hybridization. Two-dimensional gels have shown a more appropriate representation of the multitude of DNA intermediates for various geminivirus-host combinations [171,172,173,174,175,176,177,178,179,180,181,182]. These intermediates for complementary strand replication (CSR), rolling circle replication (RCR) and recombination-dependent replication (RDR) can serve directly as templates for run-off RCA. Their direct use would be prevented if the sample was heated to 95 °C beforehand. The only form which would need nicking to start replication is the usually smaller portion of covalently closed circular (ccc) dsDNA. Viral ssDNA needs a primer for further propagation; this can be a random hexamer that is added to the reaction mixture. Due to the intrinsic 3′-5′ exonuclease activity of the Phi 29 polymerase, the 3′ end of the primer should be protected by phosphoro-thioate-linked nucleotides for optimal use [183]. In addition, endogenous RNAs such as small interfering RNA (siRNA) and viral transcripts can probably serve as primers as well, and hybridize to the ssDNA at 65 °C, possibly explaining why a further purification of the crude nucleic acids may be less efficient for the final outcome of the tests.

**Figure 2:**
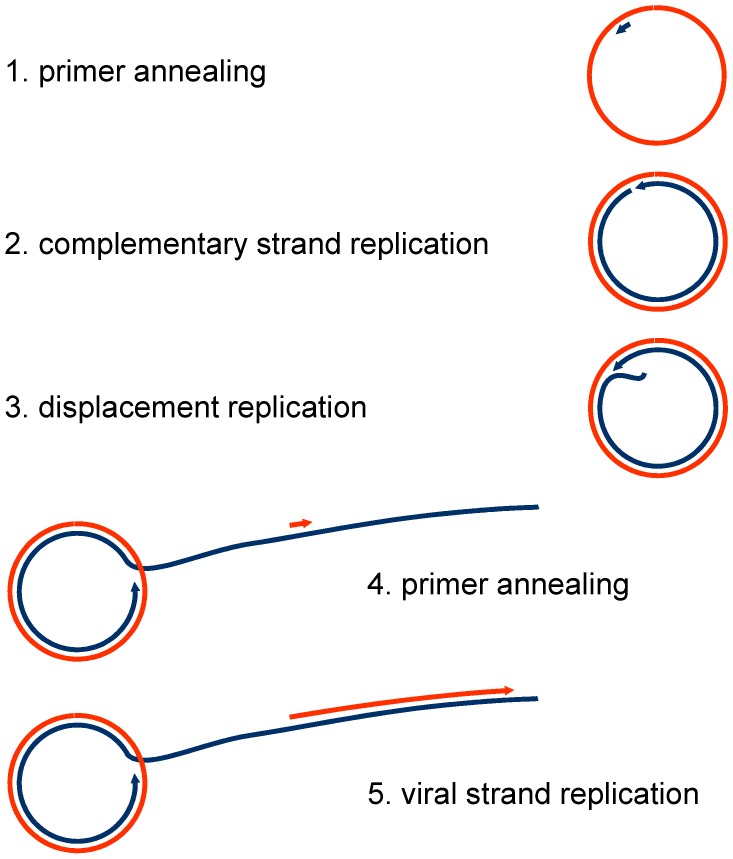
Steps of RCA on geminiviral single-stranded (ssDNA). Primers may be RNA or DNA of plant origin, or random hexamer phosphorothioate-protected primers added to the reaction mixture. Colours: red viral strand, blue complementary strand. Similar intermediates are generated in vivo during geminiviral replication modes, in CSR, RCR, or RDR.

The primary product of RCA is a complex mixture of ssDNA and dsDNA, building a huge network due to the high processivity and strand-displacement activity of the Phi 29 polymerase (Figure 1). Multiple priming on a single circular molecule can yield pinwheel-like products. For efficient RFLP analyses, it is important to note that the primary ssDNA products need a second strand replication to be cleavable by the restriction enzymes (Figure 2). The relative proportion of template DNA and the added phosphorothioate-modified primers (usually in surplus) are therefore crucial for the outcome of the assay. Infected plant samples with very high concentrations of ssDNA may need a serial dilution in two-fold steps to obtain the optimum concentration of dsDNA RCA products and a clean RFLP pattern. Incomplete digestion is frequently caused by incomplete second strand replication and results in a misleading complex fragment pattern.

## 6. The Best Choice of the Restriction Enzymes

Depending on the virus to be detected, appropriate restriction enzymes have been used, mostly *Hpa*II and *Sau*3A. For a genome size of 2500–5000 nt, enzymes that recognize 4 nt yield a sufficient number of fragments to differentiate genomic components, as well as different viruses in general [72]. The resulting fragments are usually separated in agarose (>2%; for >250 bps) or polyacrylamide (>5%; for <1000 bps) gels and are stained after the electrophoresis by ethidium bromide or SybrGold [2,19,184]. Slower migration for longer time periods during electrophoresis extends the range of resolution for a greater range of fragment sizes. Under optimal experimental conditions, the migration distances in the gel are reliable estimates of the fragment sizes (see below); anomalous migration behavior has only been observed in very few cases, probably due to a bent conformation of the particular DNA fragment [185]. In such cases, the choice of another restriction enzyme solves the problem.

The power of resolution may be enhanced by Southern blot hybridization using component specific probes, which also visualize the multitude of variants in the pool of viral DNAs and/or incompletely cut RCA products [2,96].

The best diagnostic fragments are those that are sufficiently different between various DNA components, independently of the size of the fragments, which only requires the choice of optimal gels. For statistical reasons, most fragments do not fulfill this prerequisite immediately. The most frequent fragment sizes will be more similar between different viruses just by chance. It is, therefore, interesting to analyze the basis of variability with reference to fragment size and frequency in the database of geminiviruses. In 2017, all entries for geminiviruses in the GenBank were retrieved, and 9140 non-redundant entries for individual components from this database selected to digest them by in silico restriction using Python scripts. The nucleotide contents were analyzed for the most prominent genera (*Begomovirus*, *Curtovirus*, *Mastrevirus*) (Table 1).

**Figure 3:**
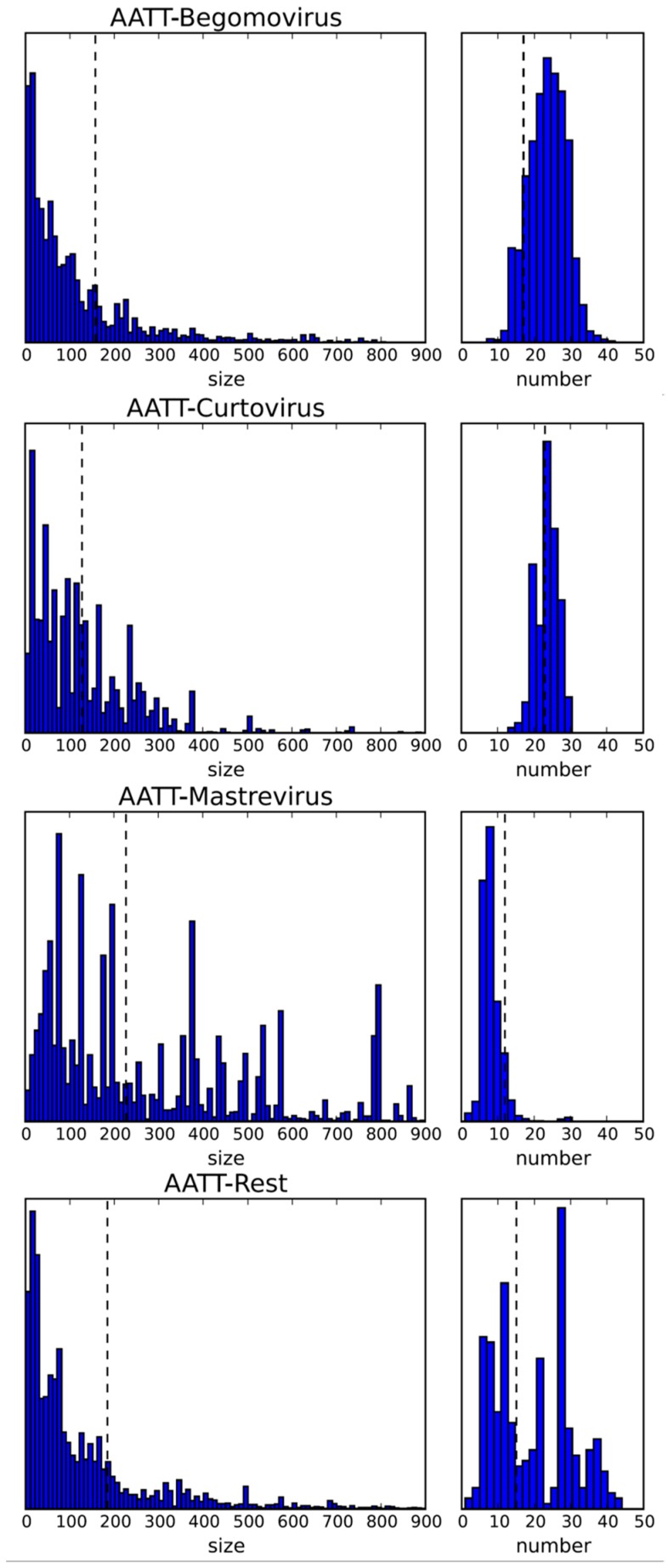
Statistics of the restriction enzyme recognition sequence AATT (for MluI) for the indicated genera and all others (rest). Left column for fragment size, right column for distribution by number of fragments for the indicated genera based on the data as shown in Table 1. Stippled lines indicate the expected value, calculated on the basis of the nucleotide frequencies. y axis: relative frequency; size in bp. The whole data set for all four nucleotides recognition sites is given in Figure S1.

**Figure 4:**
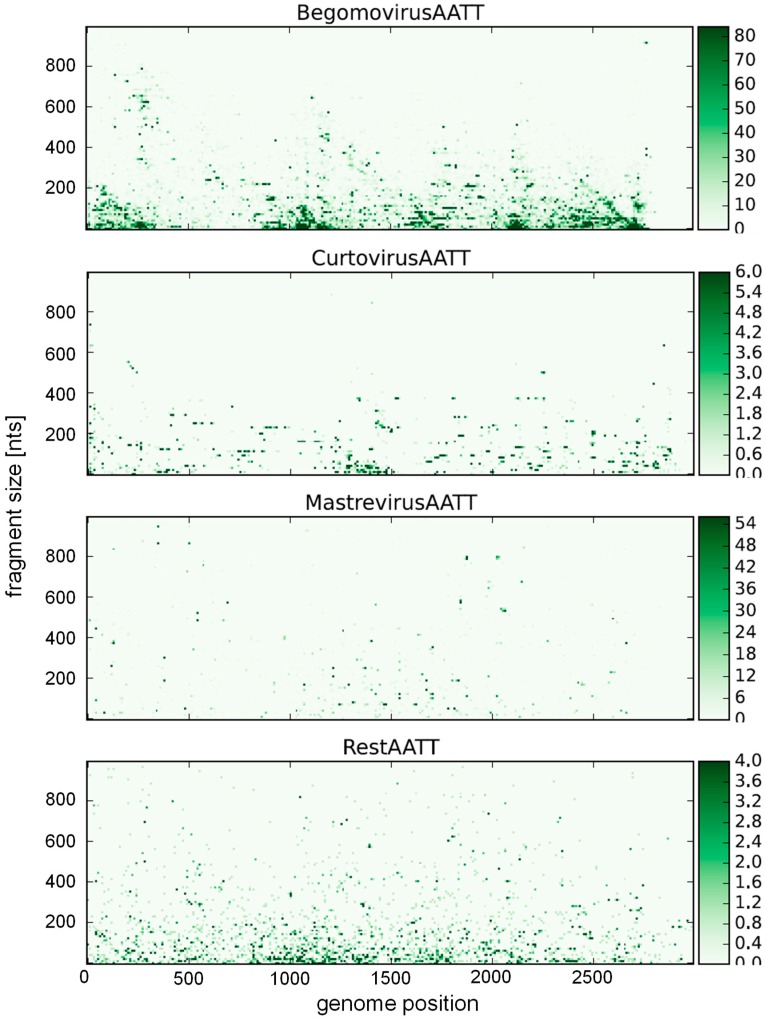
Statistics of the restriction enzyme recognition sequence AATT (for MluI) as in Figure 3, but relating fragment sizes to genome position. Dot intensity indicates frequency. The whole data set for all four nucleotides recognition site is given in Figure S2.

A bias towards an enhanced T frequency over the expected frequency has to be noted for begomoviruses (30.1%) and curtoviruses (31.4%), possibly the result of a high deamination rate leading to C > T transitions, as observed experimentally [180,186]. Consequently, A&T-rich recognition sequences of restriction enzymes should yield more and smaller fragments. The resulting statistics for all available restriction enzymes is provided in the Supplementary (Figures S1 and S2), with an example given for the recognition sequence AATT (*Mlu*I) (Figure 3 and Figure 4). This sequence is clearly overrepresented for begomoviruses, and underrepresented for mastreviruses (Figure 3). The distribution along the genome (Figure 4) is mostly random with only few hot spots for begomoviruses in regulatory DNA elements (such as promoters, ~2500 to 200 nt, and terminators, ~1000 to 1200 nt). As a consequence of these distributions, more and smaller fragments can be obtained for begomoviruses than for mastreviruses, and the chance to detect diagnostic fragments upon comparing the RFLP products of all components under consideration increases. For novel projects, the data provided in the Supplementary material may be a guide to choosing the best restriction enzymes.

## 7. Standardization to Determine Fragment Sizes

**Figure 5:**
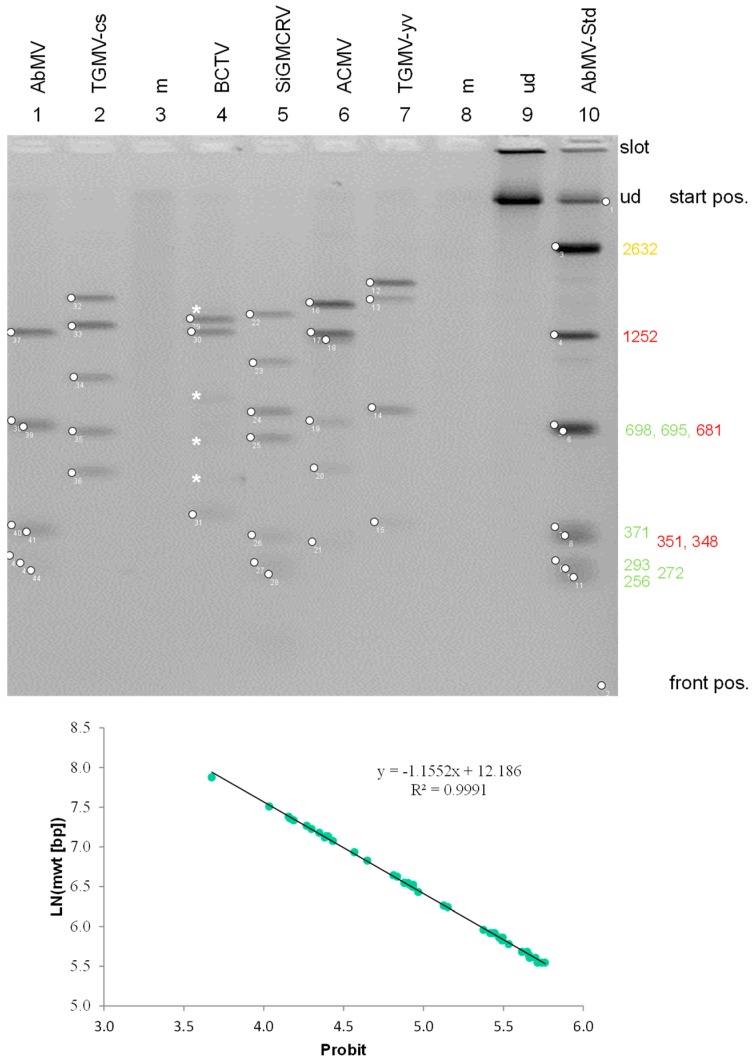
Accuracy of the fragment size determination. RCA/RFLP (HpaII) of various geminivirus samples (Abutilon mosaic virus, AbMV; tomato golden mosaic virus-common strain, TGMV-cs; beet curly top virus, BCTV; Sida golden mosaic Costa Rica virus, SiGMVCRV; African cassava mosaic virus, ACMV; tomato golden mosaic virus-yellow vein, TGMV-yv) are compared with an AbMV standard (mixture of *Pst*I- and *Hpa*II-cut RCA products), mock-inoculated (m), and undigested (ud) of AbMV RCA product. Separation was performed in a 2% agarose gel and staining with ethidium bromide (inverse image presentation). The positions of genomic fragments (circles) were determined using Fiji (Image J), the corresponding migration distances in numbers of Pixel recalculated in Excel (lower graph) to relate the fragment sizes (in bp) in logarithmic scale to probit values. All data fit to a line with Pearson’s correlation coefficient of R^2^ = 0.991 corresponding to a mean error of 1.6±1.3. Asterisks indicate fragments from defective DNAs of BCTV. Molecular weights (nt) for the standards are indicated on the right, yellow genomic size, red DNA A, green DNA B. Relative migration distance (Rf) values were determined in relation to the start and front positions.

An accurate determination of the fragment sizes obtained by gel electrophoresis is crucial to discriminate between matching and non-matching fragments, in order to differentiate viral components. Usually, a log relationship between the molecular weight and the migration distance (normalized as retardation factor (Rf) values) can be obtained in the central part of a gel, but for the complete gel, a sigmoid curve gives a better fit [2,72] (Figure 5, Table S2). The inverse function to linearize a sigmoid curve is obtained by a Probit analysis [187], which can be performed easily with the Excel function [=5 + NORM.INV(D5;0;1)] (Table S2) This approach optimized the log-lin fit (R^2^ = 0.991) and led to an error for fragment size determinations below 3% (Figure 5) in a large variety of sample analyses irrespective of virus, plant and gel in our lab [72] (Table S2).

The appropriate standard fragments in each gel are absolutely necessary to reach this degree of accuracy. The use of RCA/RFLP products of sequenced viruses for comparison has been found superior to other marker restriction fragment mixtures. If a specific virus is suspected in the plant, the RCA products of this virus retrieved from cloned material may be sufficient and easy to compare for calibrating the analysis. If unknown viruses have to be compared to the electronic database, a broad range of fragment sizes for the standards is desirable. The RCA products of plasmids may be useful, or Abutilon mosaic virus (AbMV) DNA may be chosen, since this virus is available worldwide in botanical and market gardens [2]. AbMV is, in addition, an excellent training object, since the woody Abutilon plants, harbor mucilage and secondary metabolites that may be a challenge for beginners.

An incorrect assignment of a band in the gel to a fragment size is tested most easily by way of graphical evaluation (Table S2). The whole set of results can be scrutinized for internal consistency (Table S2), and corrected if necessary. The more bands are correctly assigned, the more accurate the determination of the remaining unsure bands.

If digestion with the restriction enzyme is complete for each component, the band intensities of the proper genomic fragments should follow a consistent molar series for each component. This can be graphically checked by scanning a lane by ImageJ software and judging the peak heights decline. Double and multiple bands for fragments with similar molecular weight can be identified, as well as products from D-DNAs, which can also be identified if samples from separate plants are compared, since they are usually different in different individual plants [43,54,105]. Some limits for the representation of single components in RCA pools have be reported for nanoviruses [188].

## 8. Get the Unknown and Unexpected

The major advantage of RCA, in comparison to PCR and immunodetection, is its ability to detect circular DNA molecules without any *a priori* knowledge. When viruses are imported into a new country, such as tomato yellow leaf curl virus (TYLCV) in the Americas [189] or Northern Europe, [95] and squash leaf curl virus (SLCV) in the Middle East [103], they can be recognized. Samples with aSats, which were believed to be restricted to the Old World before, were found in Brazil, Venezuela, and Cuba [38,96,190]. Moreover, mitochondrial plasmids were amplified from certain plant species [24,43]. Screening germplasm bank collections with RCA/RFLP, in particular for vegetatively propagated plants, with RCA/RFLP provides important precautions to prevent dissemination of geminivirus-infected material [43].

## 9. Resurrection of Viruses

Although the plain RCA products can be generally regarded as safe with reference to natural spread, it is possible to raise infectious viruses by biolistic inoculation of the RCA-amplified DNA to test plants under laboratory conditions [19,36,68,178,179,180,184,186]. This procedure has the advantage of preserving the whole population structure of a virus quasispecies [180,186]. In combination with the selection of fragments by gel purification, religation to circularize the DNA fragments, and a second RCA, a proper infectivity was obtained for viruses that were otherwise recalcitrant to bacterial cloning [184]. Only in rare cases is a linear fragment of genomic size sufficiently infectious using rub-inoculation, such as for ACMV [191]. Typically, partial dimers (“bitmers”), dimers, or the multimers of the RCA products without any further digestion are the prerequisite for replicational or recombinational release of unit length DNA circles inside cells, in order to retain redundant sequence regions [192].

If infectious DNA is to be cloned in bacterial plasmids, RCA products are digested by restriction enzymes at limited concentration or incubation time [27,57]. Fragments of sizes larger than genomic length are gel-purified and inserted into plasmids. Usually, limited cleavage of the RCA product with *Sau*3A (or *Mlu*I) and insertion into *Bam*HI (or *Eco*RI) sites of the plasmids is generally applicable for all genomic components without a need to search for proper restriction enzymes. The versatile small agrobacterial plasmid pGreen [193] has been used for *in planta* delivery by stem inoculation or leaf infiltration with many viruses, aSats and plants [57,68,72,108,124,179], but other Ti-based plasmids are used routinely as well.

## 10. Get the Whole Sequence Information

If some sequence information is known to deduce a specific primer, and if the plant under investigation is infected by a single virus, direct sequencing of the RCA product is the technique of choice to obtain the whole sequence of the virus components by primer walking [68,184], in addition to conventional cloning and sequencing. The resulting sequence will represent the master or consensus of the DNA population, ignoring the variability within the population, and may deviate from individual clones. In addition, several deep sequencing techniques have been applied directly to the RCA products (Circomics). Pyrosequencing had the advantage of longer reads, but more errors were also observed, in particular for homo-polynucleotide stretches [72,96]. Illumina sequencing exhibited a lower error rate and yielded less extended, but still sufficient, reads to determine the mutational variability within a viral DNA population, when proper plasmid controls were included to check the error rate with this technique [2,180,186]. Unexpectedly high proportions of D-DNAs were identified in addition. Recently, single molecule sequencing has been applied to many individual samples in a single assay [194]. This approach has a great advantage in the detection of intra- and intermolecular recombinant components.

For practical purposes, such as the identification of known viruses, direct sequencing of RCA products across the short segment of the most variable intergenic region in the virus component will be satisfactory for most applications. With the decreasing costs of commercial sequencing, this approach will outperform the RFLP analyses for cases of known conserved primer sequences, e.g., within the coat protein or Rep gene. The information thus obtained is much more precise and can be easily compared to the electronic databases.

For fundamental research, Circomics extends the view to the complexity of DNA virus variation and the unexpected molecules generated in response to different plants and plant lines. A better understanding of the viral population structure will have many implications for resistance breeding, irrespectively of whether a conventional method or genetic engineering is used. It is important to note that viruses may be regarded as highly polyploid entities. An error-prone lifestyle is, therefore, not only possible because wild type (wt) versions can complement mutants in a cell, but it is also advantageous for the viral evolution, because a reservoir of potential survivors for changing environments is created before it is needed. Following this basic concept, begomoviruses (Euphorbia yellow mosaic virus, EuYMV; Cleome leaf crumple virus, ClLCrV) and their aSats were investigated in various T-DNA insertion lines of *Arabidopsis thaliana* to determine the shifts in the population structure and the influence of single host genes on the replication of the virus [108,178,179,180,186]. Biolistic inoculation of RCA products proved to be more efficient than agroinfectious clones in this case, and avoided a contribution of mutations within the bacteria. The changing viral DNA populations were determined by Illumina sequencing, and the technical error rates were checked with plasmid controls spiked into the samples. With these precautions, high substitution rates were regularly determined. Some variations were observed in different genetic plant lines, e.g., when a factor for non-homologous end-joining (KU80) retarded geminiviral multiplication [178], or when the homologous recombination mediator RAD51D promoted the geminiviral infection [186]. The role of error-prone translesion DNA synthesis in early viral replication inside phloem cells has been investigated [180]. Typically, high substitution rates of 10^−4^ to 10^−3^ were noticed in all these data sets, with preferences for deamination and oxidative changes of the nucleobases. In addition, high levels of D-DNAs were detected which had been overlooked before, since they form a heterogeneous population not represented in discrete bands in the RFLP analyses, but in background smears only. Raising elevated levels of DI-DNAs may be important as a means for cross-protection. However, D-DNAs may have ambivalent effects on symptoms and viral multiplication [54,105], as shown for beet curly top virus (BCTV). This allowed for the conclusion that it is more appropriate to employ the whole viral DNA pool to experimentally challenge a resistance trait, than to use a single infectious clone with unknown relevance for the field situation. Moreover, RCA technology makes it possible to follow the changes in the population structure when new cultivars and environmental conditions are applied, to establish sustainable plant protection.

## 11. Outlook

In spite of some progress in engineering resistance in plants against geminiviruses and nanoviruses, quarantine measures, including early detection of invading viruses, are still the method of choice to combat epidemics worldwide. Educational training of farmers and plant protection staff to enable early eradication remains the key remedy. The RCA technology described here is simple and can be applied cost-effectively, in particular for tropical and subtropical countries with low budgets. Although commercial kits may be still too expensive for many applications in agriculture in these countries, an alternative exists, since the core enzyme, the Phi29 DNA polymerase, can be easily expressed in *E. coli* as a fusion protein [195]. For more sophisticated analyses, the growing treasure of databases allows immediate recognition of newly invading viruses. Improvements and economization of sequencing technology (in particular of single molecules) in central laboratories will expand the applicability of this diagnostic technology. A debate on the usage of the expanding knowledge has been raised, following the negotiations of the Nagoya protocol. Since the problem raised by geminiviruses and nanoviruses is a global one, rather than restricted to individual nations, an open access policy for fundamental research would be a wise option [196].

## Figures and Tables

**Table 1:** Occurrence of nucleotides in the different geminivirus genera (%); *n*, the number of data base entries analyzed.

Genus	Mean Length	Mean Frequencies						Entries	Standard Deviations					
		A	C	G	T	A + T	G + C	n	A	C	G	T	A + T	G + C
*Begomovirus*	2719	26.6	20.2	23.1	30.1	56.6	43.3	7001	1.1	1.5	1.6	1.5	2.1	2.1
*Curtovirus*	2932	28.2	17.3	23.1	31.4	59.6	40.4	193	1.1	0.4	1.1	0.6	1.4	1.4
*Mastrevirus*	2684	25.4	22.6	25.8	26.2	51.6	48.4	1619	1.4	1.9	1.0	1.7	2.4	2.4
Other	2708	26.5	22.6	23.5	27.4	53.9	46.1	327	2.6	4.3	2.1	3.7	4.9	4.9

## Supplementary Materials

The following are available online at http://www.mdpi.com/1999-4915/10/9/469/s1. Figure S1: Statistics of the restriction enzyme recognition sequences as described in Figure 3 for the whole data set. Figure S2: Statistics of the restriction enzyme recognition sequences as described in Figure 4 for the whole data set. Table S1: Literature survey, 2004–2018. Table S2: Worksheet to calculate fragment sizes in relation to migration distances after gel electrophoresis.
