# Supplementary material for: Barcoding of Plant Viruses with Circular Single-Stranded DNA Based on Rolling Circle Amplification
Source: Viruses. 2018 Aug 31;10(9):469. doi: 10.3390/v10090469 (PMC6164888; doi:10.3390/v10090469)
Supplement: Supplementary file 1 [file viruses-10-00469-s001.zip › 3-viruses-345336-supplymentary/Jeske18R_Figures S1.pdf]

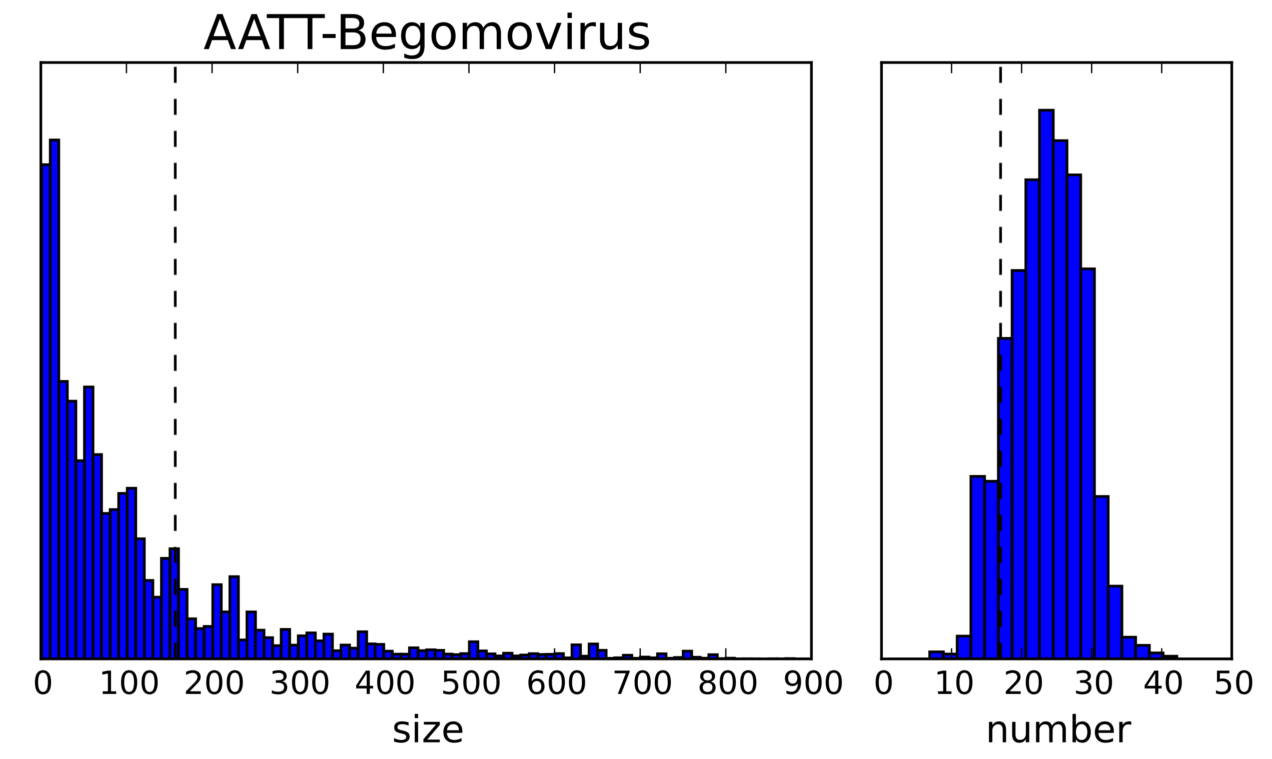
AATT-Begomovirus
0
100
200
300
400
500
600
700
800
900
size
0
10
20
30
40
50
number

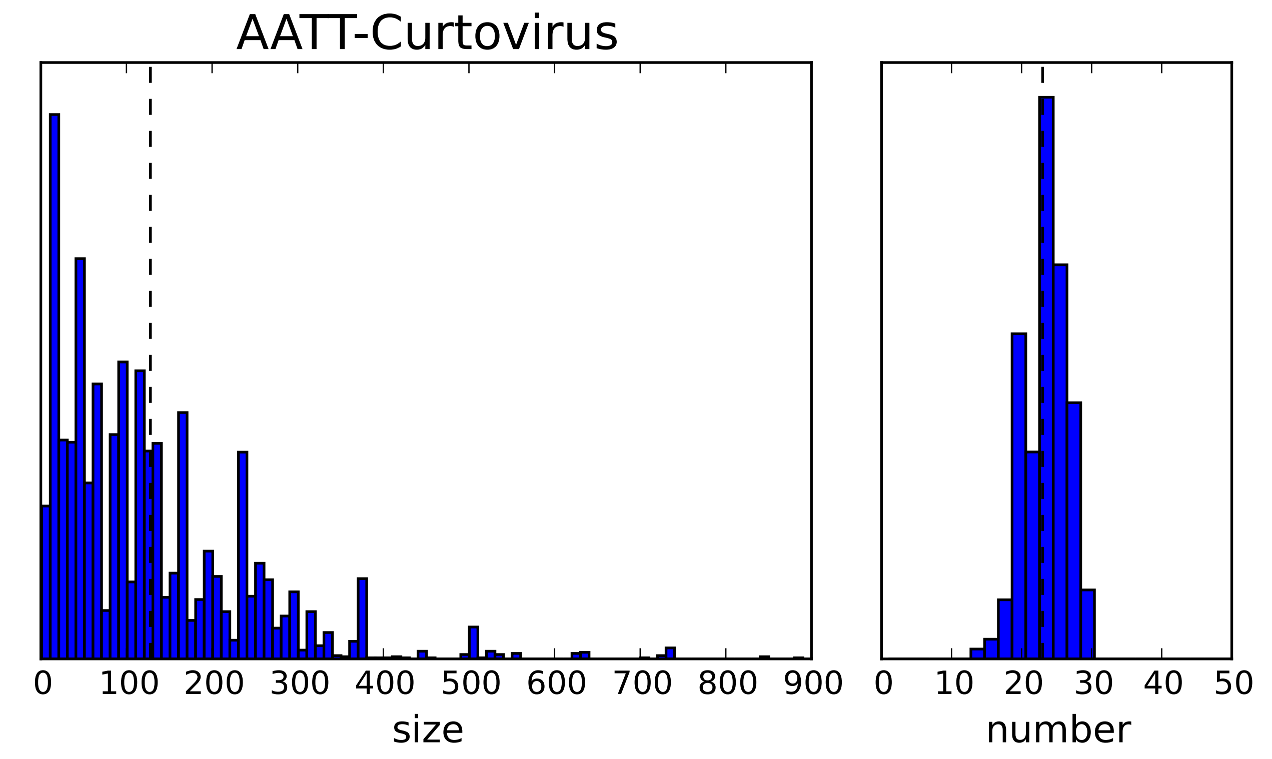
AATT-Curtovirus
0
100
200
300
400
500
600
700
800
900
size
0
10
20
30
40
50
number

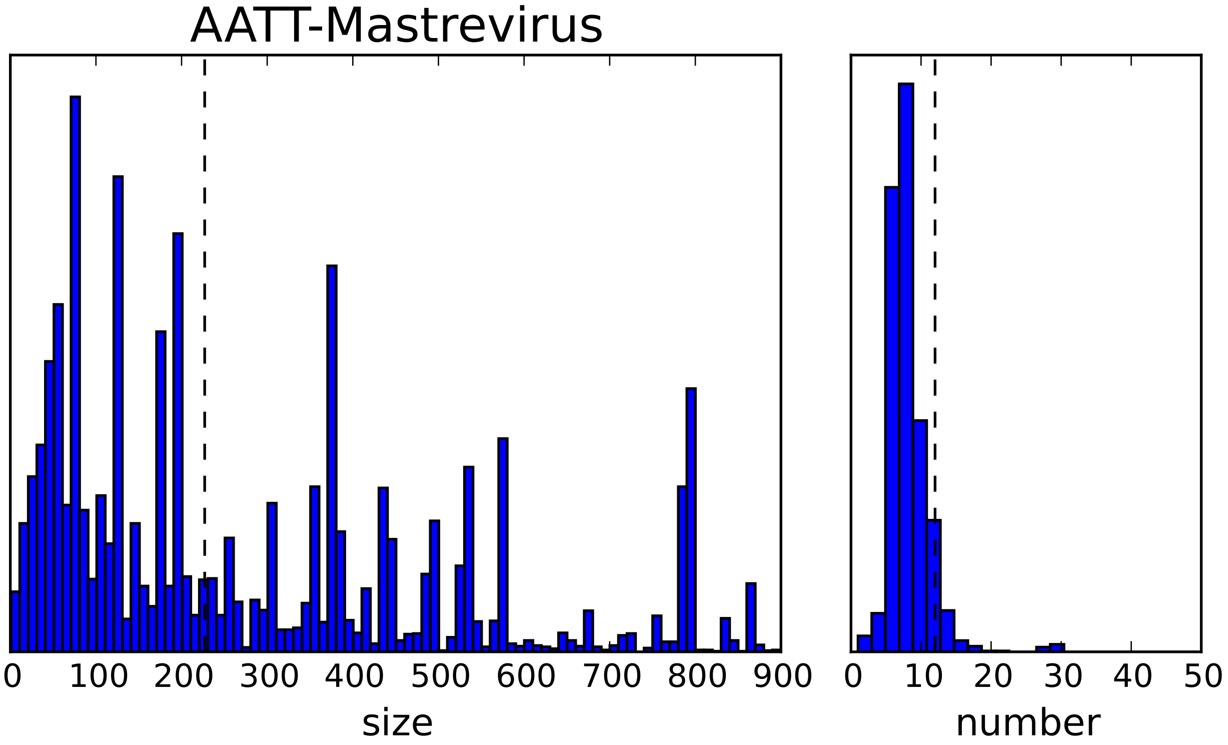
AATT-Mastrevirus
0
100
200
300
400
500
600
700
800
900
size
0
10
20
30
40
50
number

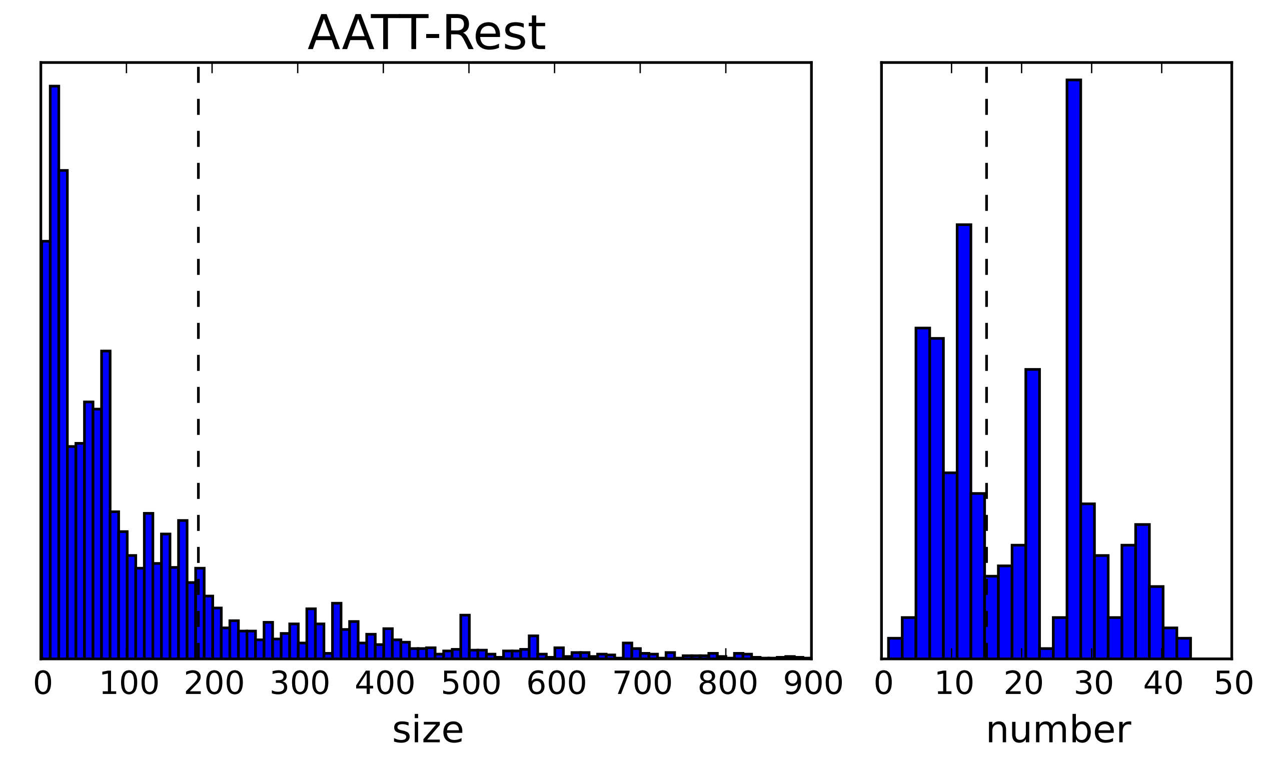
AATT-Rest
0
100
200
300
400
500
600
700
800
900
size
0
10
20
30
40
50
number

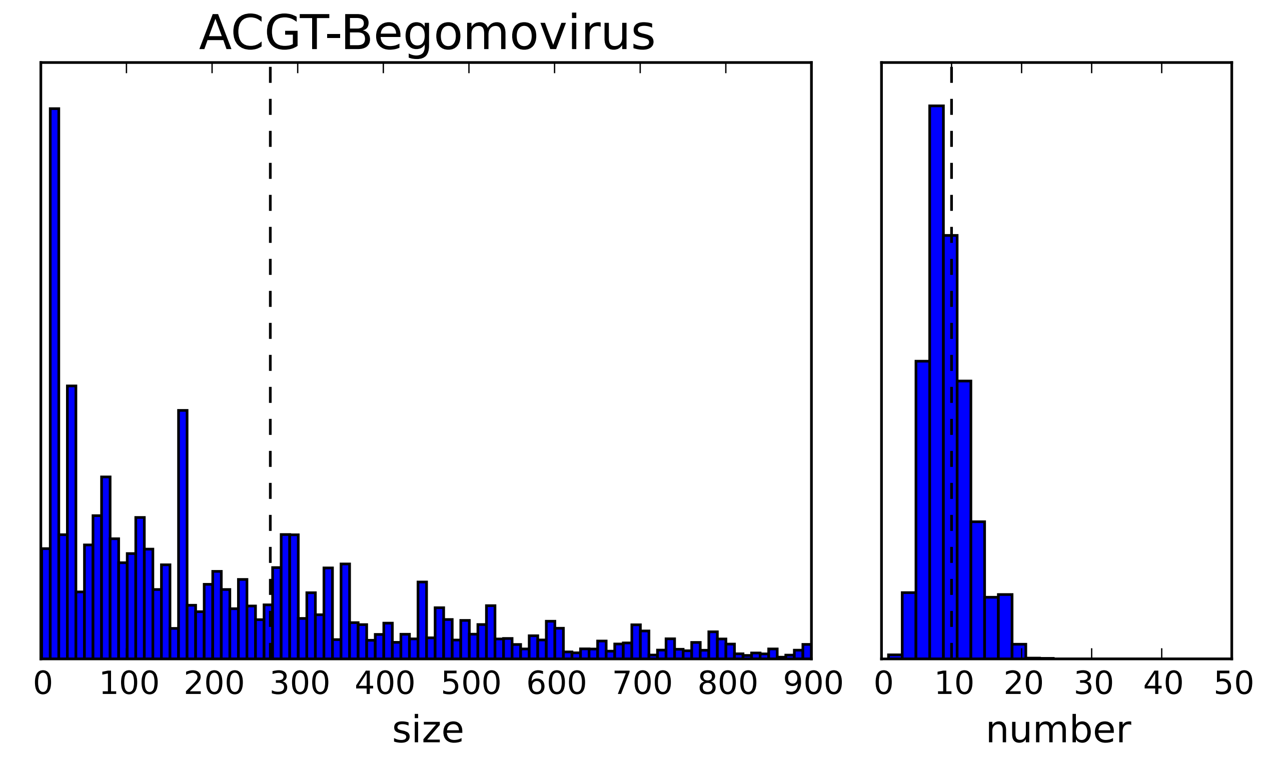

ACGT-Begomovirus
0
100
200
300
400
500
600
700
800
900
size
0
10
20
30
40
50
number

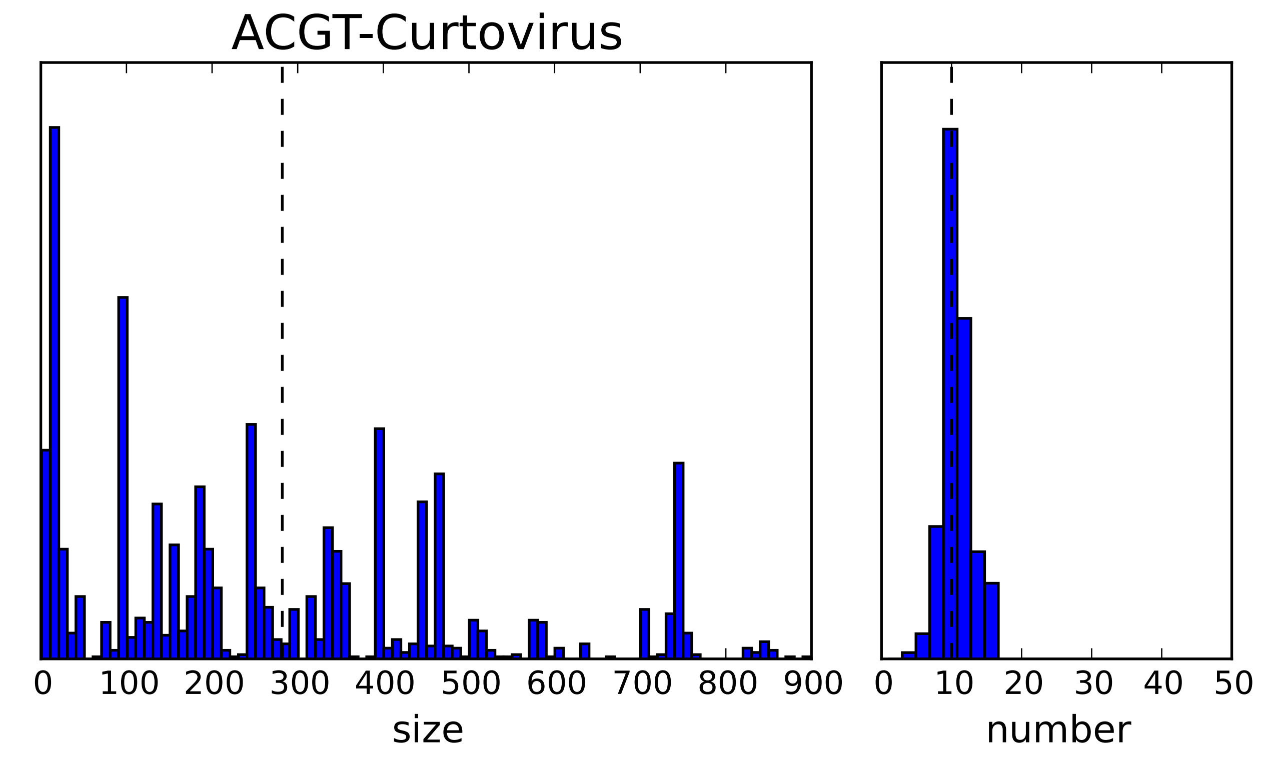
ACGT-Curtovirus
0
100
200
300
400
500
600
700
800
900
size
0
10
20
30
40
50
number

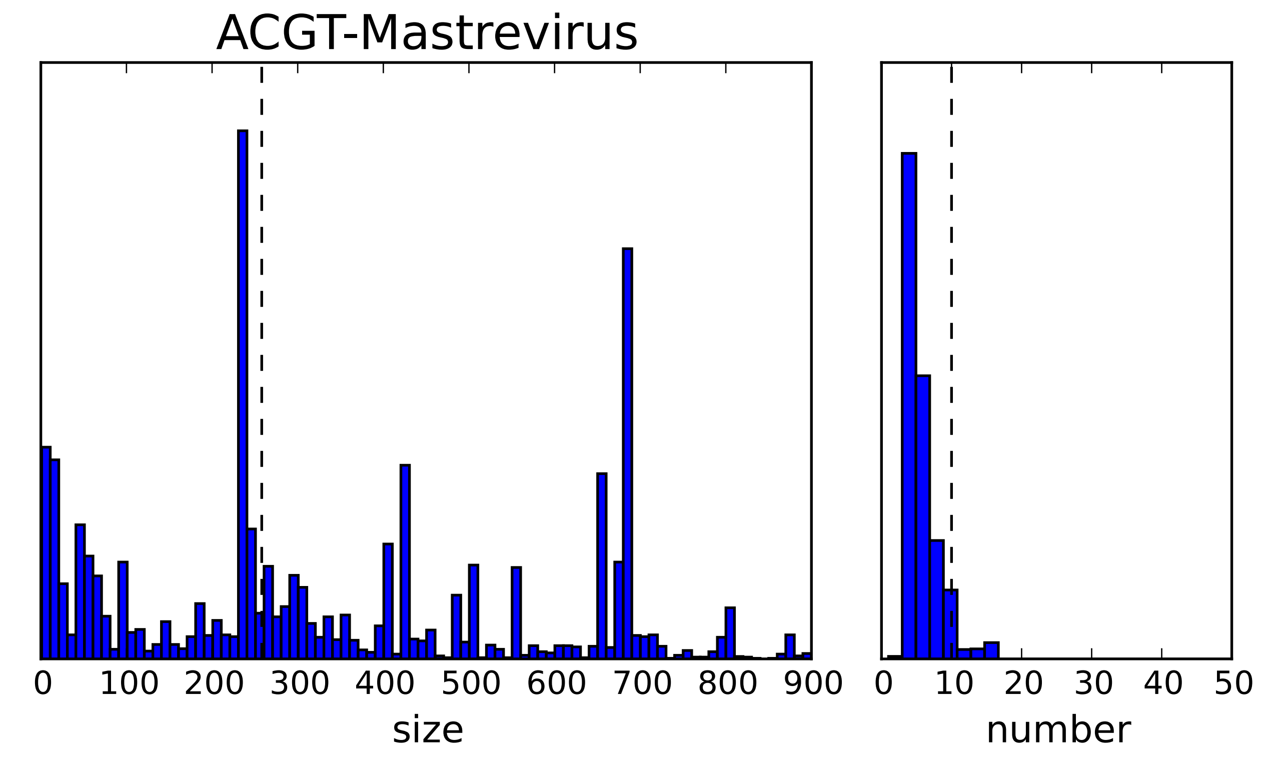
ACGT-Mastrevirus
0
100
200
300
400
500
600
700
800
900
size
0
10
20
30
40
50
number

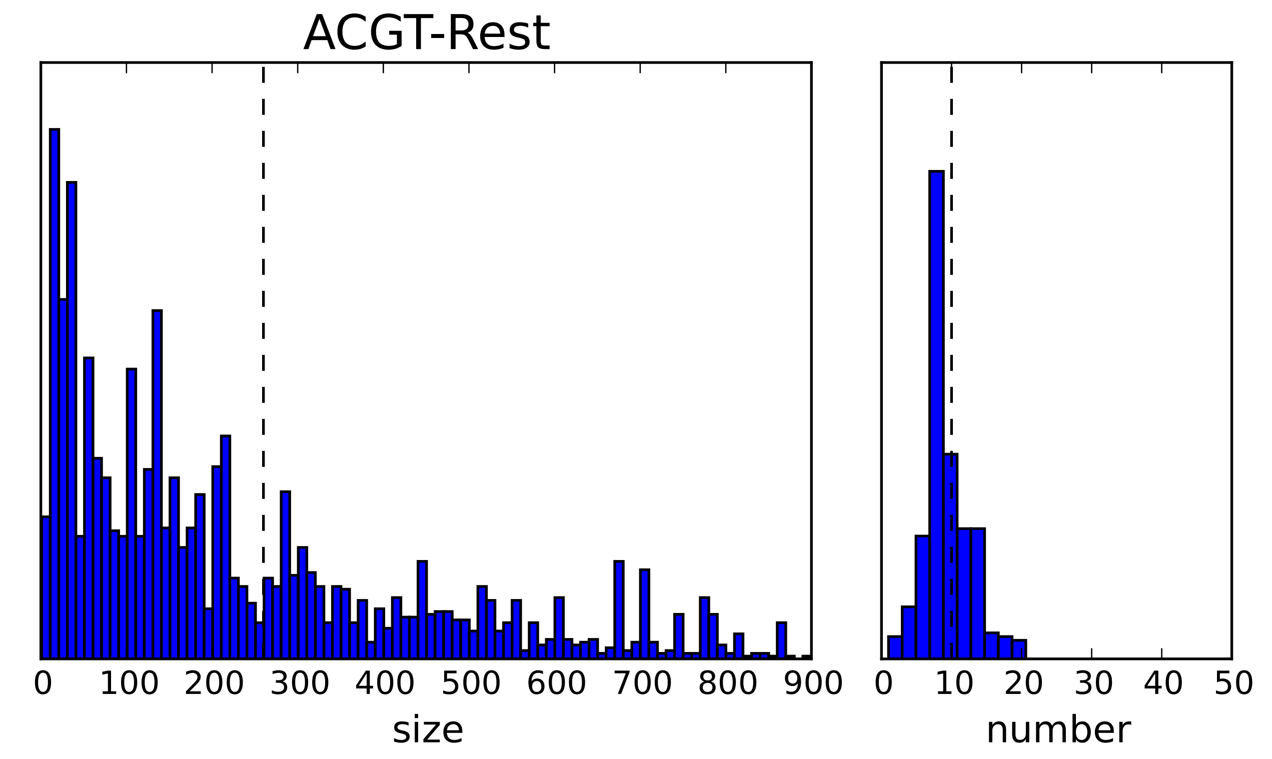

ACGT-Rest
0
100
200
300
400
500
600
700
800
900
size
0
10
20
30
40
50
number

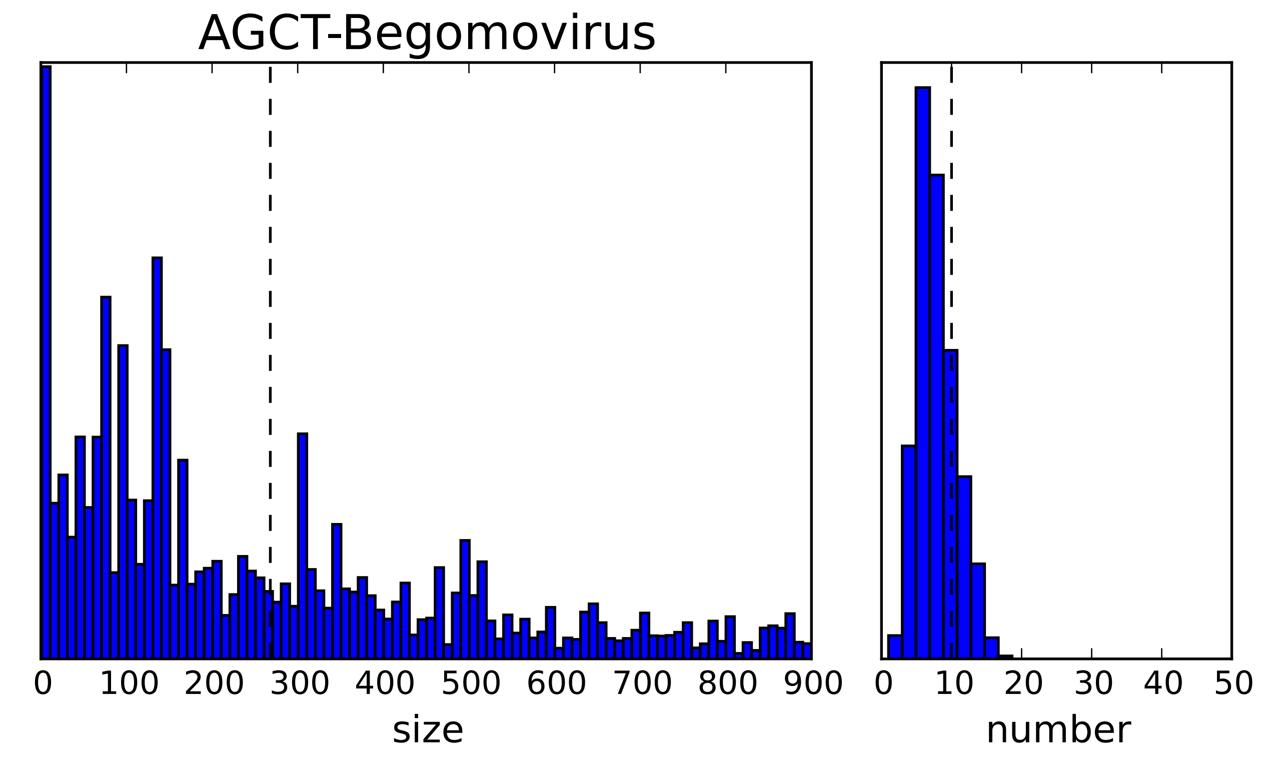
AGCT-Begomovirus
0
100
200
300
400
500
600
700
800
900
size
0
10
20
30
40
50
number

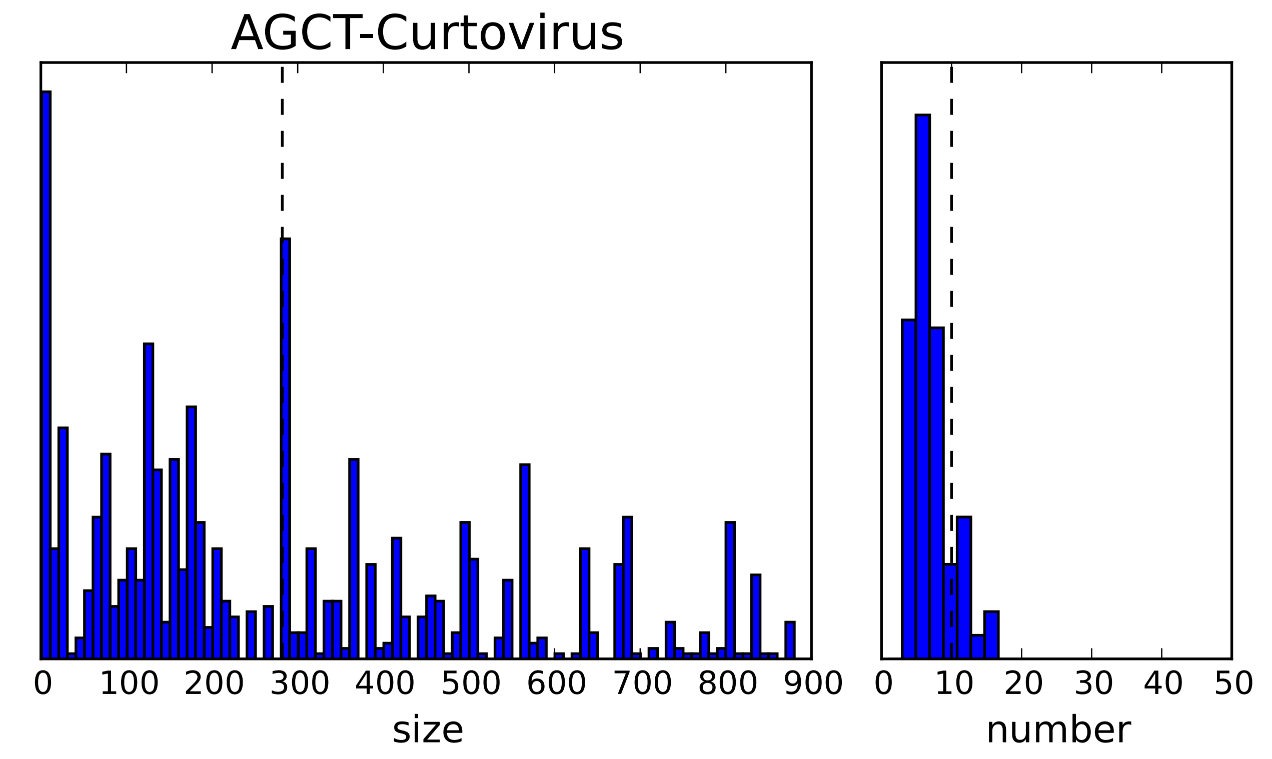
AGCT-Curtovirus
0
100
200
300
400
500
600
700
800
900
size
0
10
20
30
40
50
number

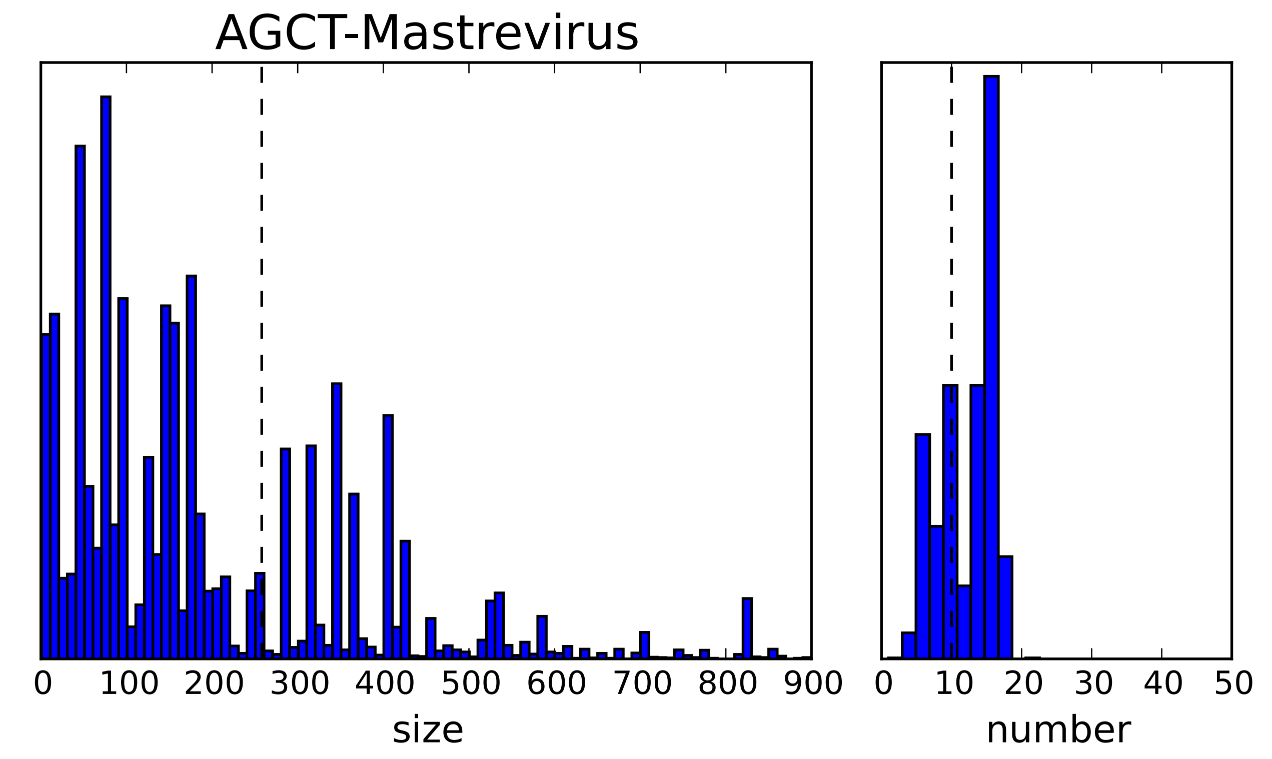

AGCT-Mastrevirus
0
100
200
300
400
500
600
700
800
900
size
0
10
20
30
40
50
number

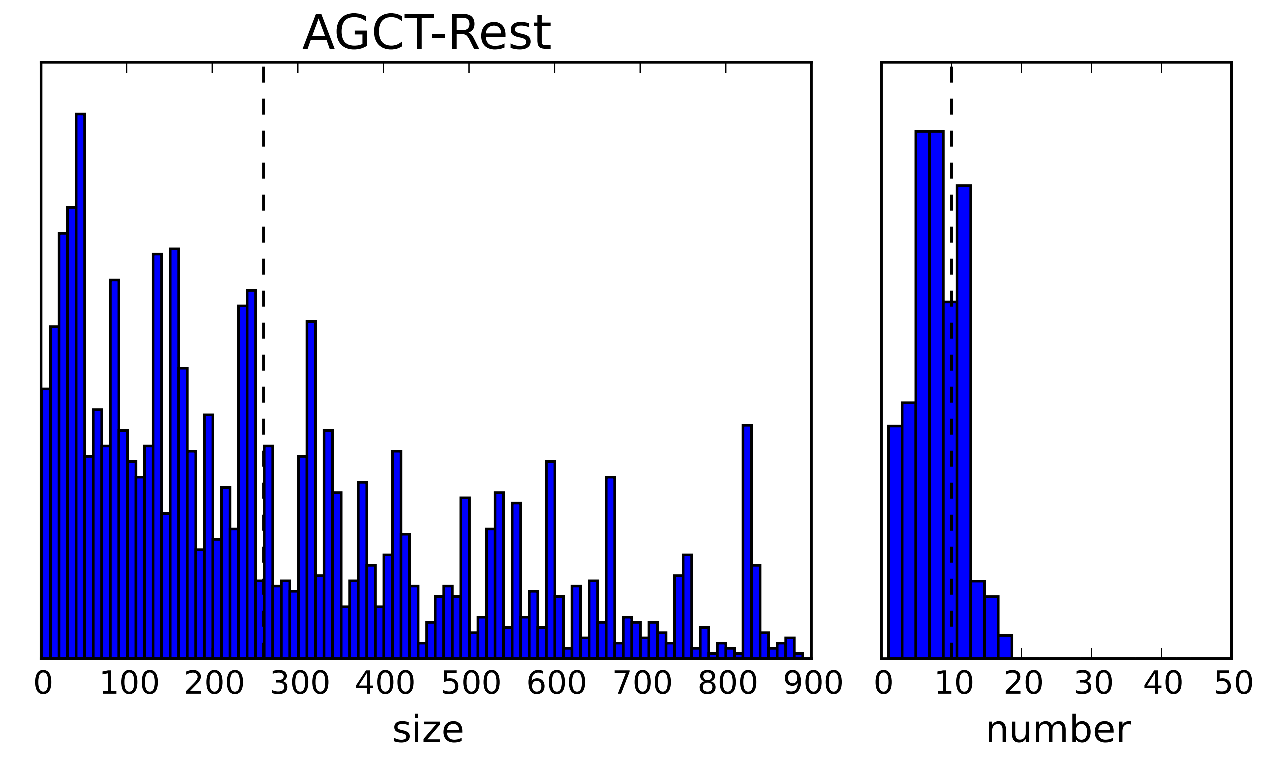
AGCT-Rest
0
100
200
300
400
500
600
700
800
900
size
0
10
20
30
40
50
number

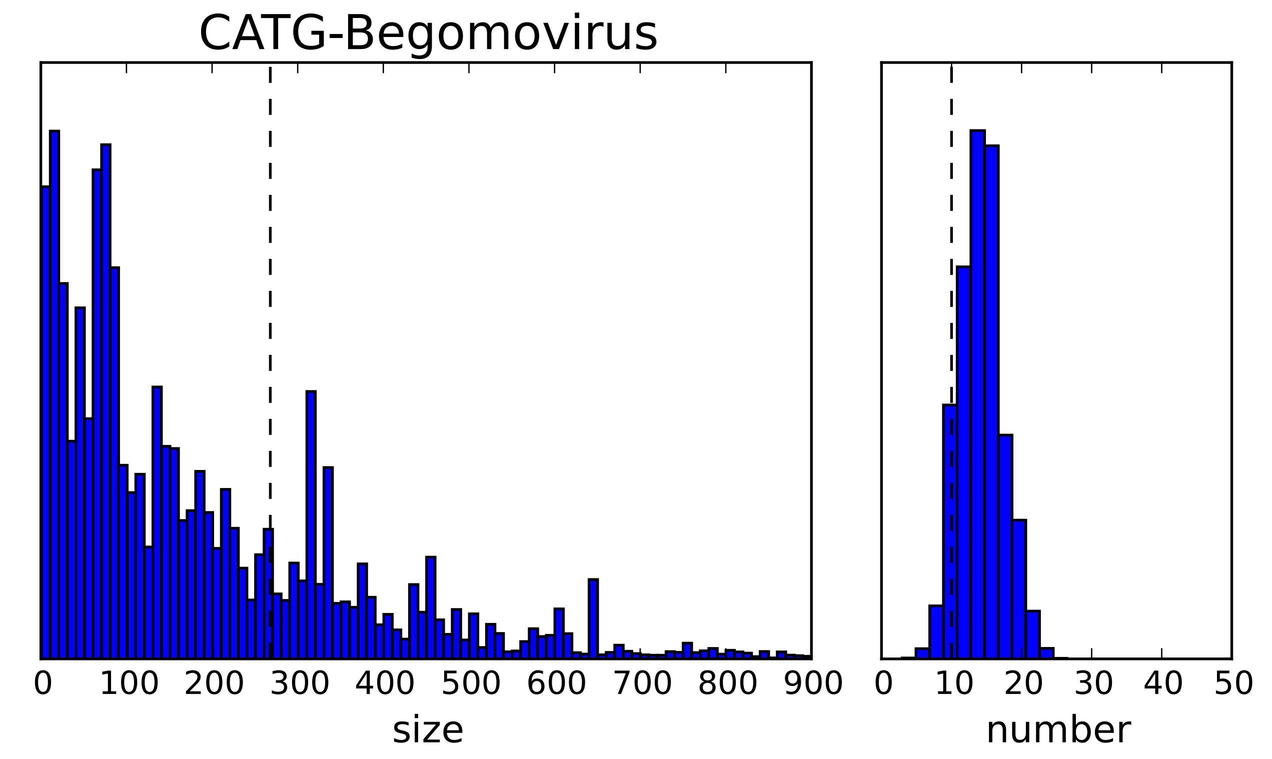
CATG-Begomovirus
0
100
200
300
400
500
600
700
800
900
size
0
10
20
30
40
50
number

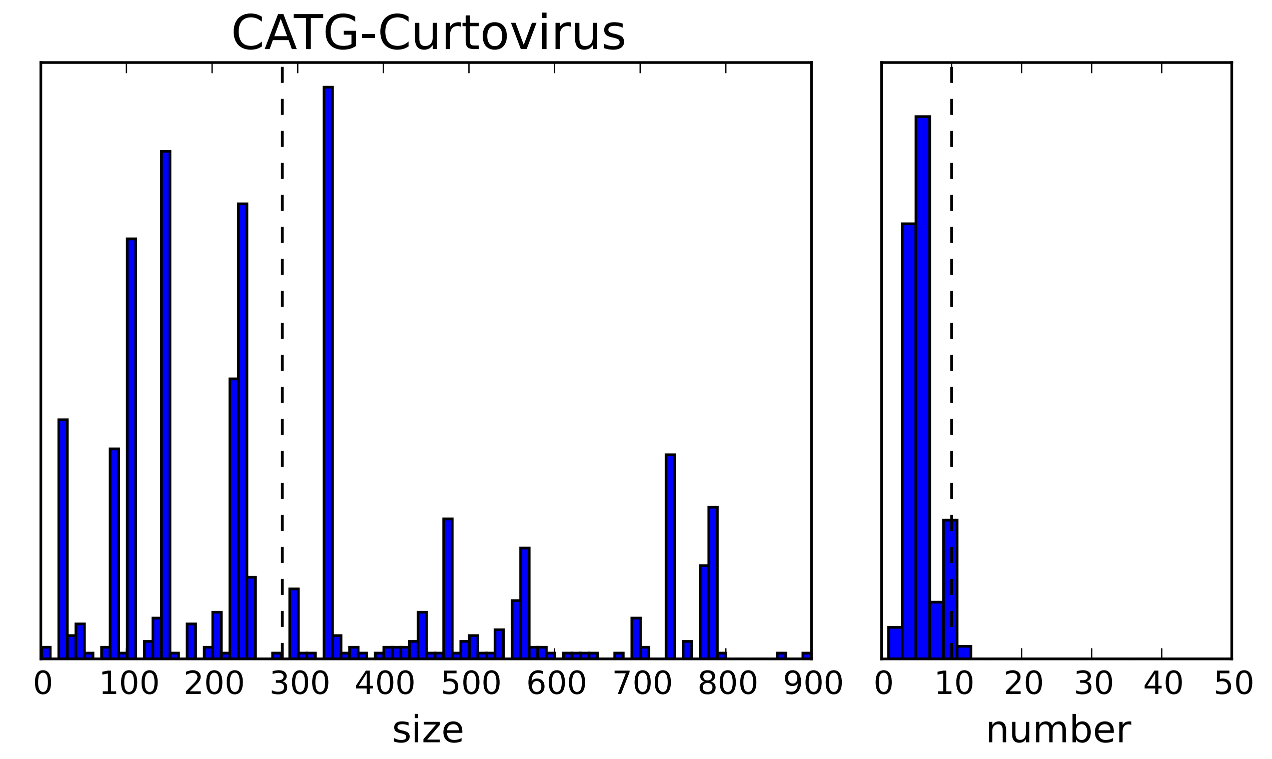
CATG-Curtovirus
0
100
200
300
400
500
600
700
800
900
size
0
10
20
30
40
50
number

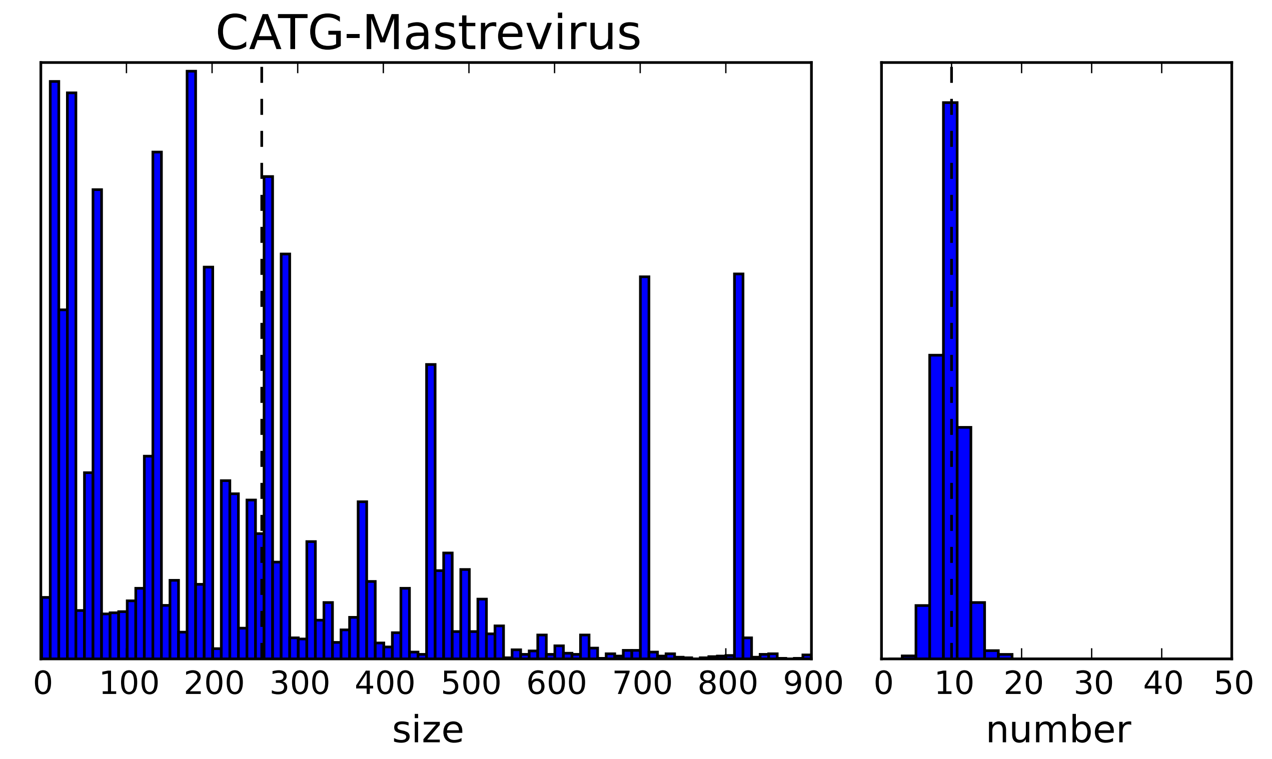
CATG-Mastrevirus
0
100
200
300
400
500
600
700
800
900
size
0
10
20
30
40
50
number

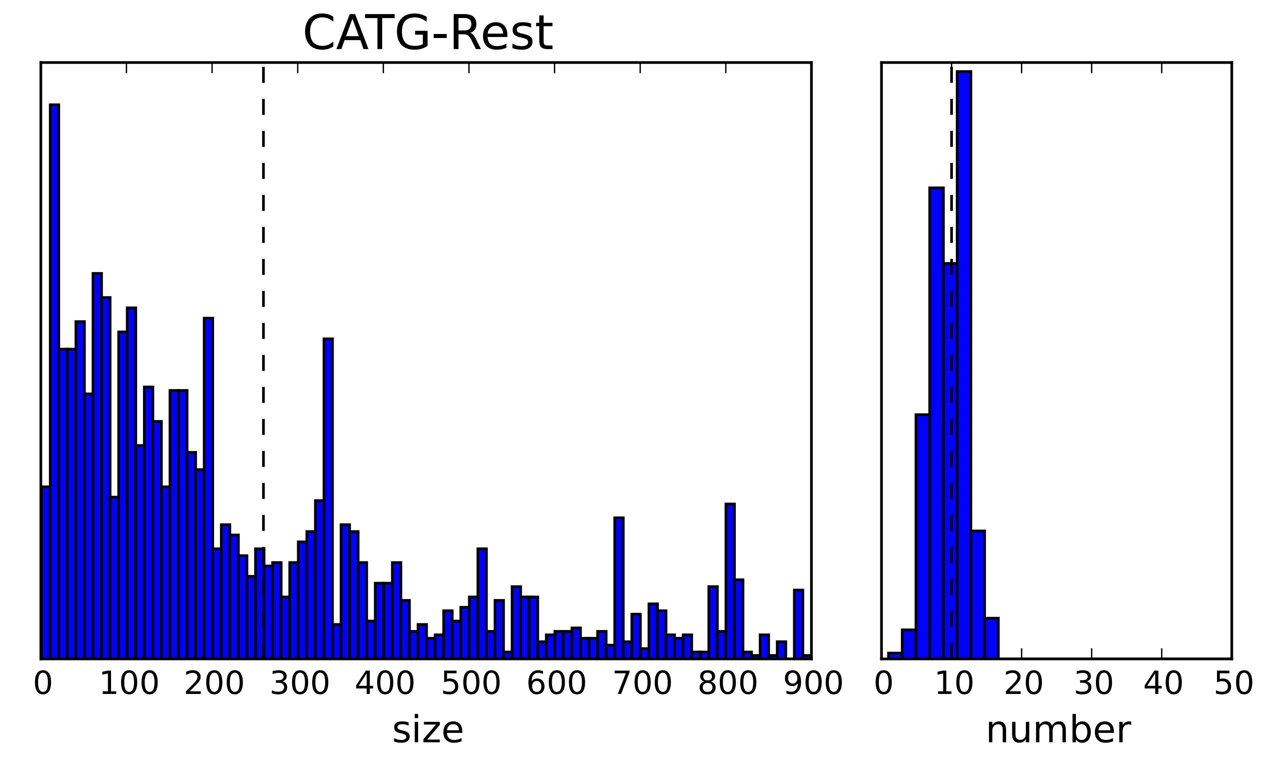
CATG-Rest
0
100
200
300
400
500
600
700
800
900
size
0
10
20
30
40
50
number

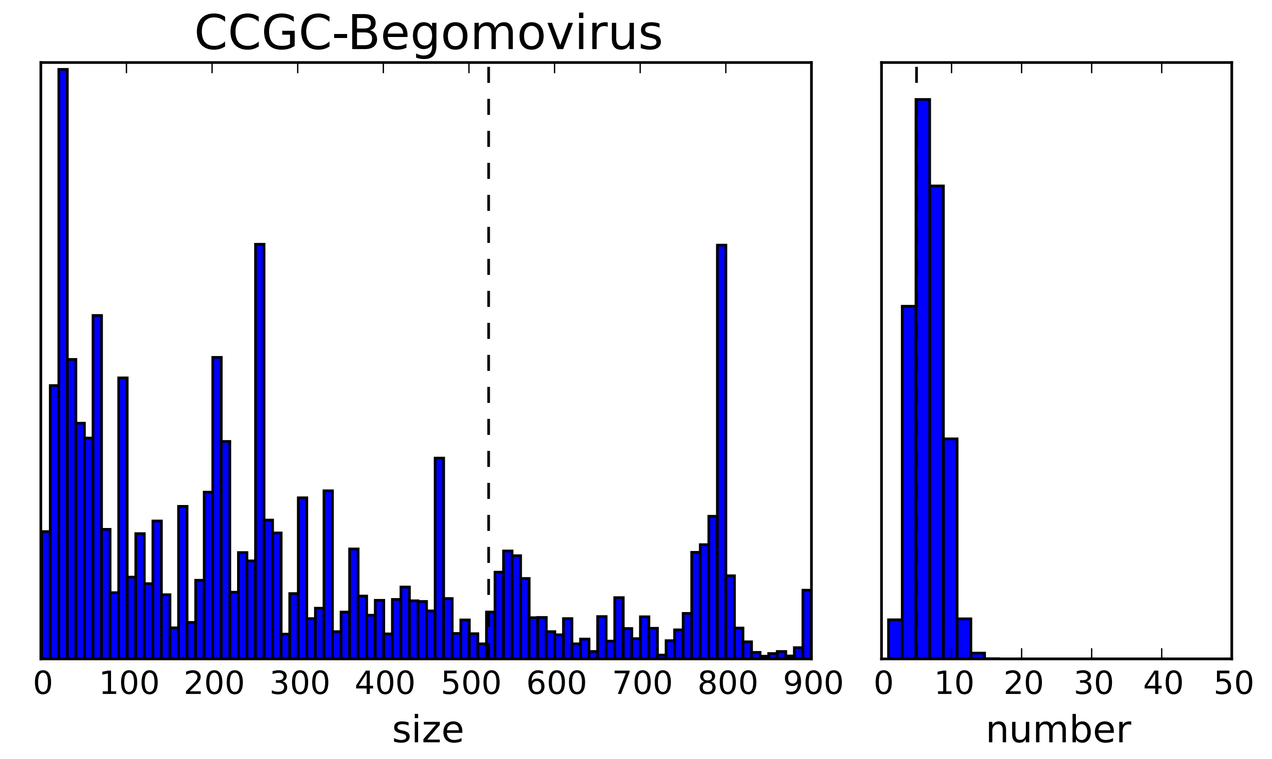
CCGC-Begomovirus
0
100
200
300
400
500
600
700
800
900
size
0
10
20
30
40
50
number

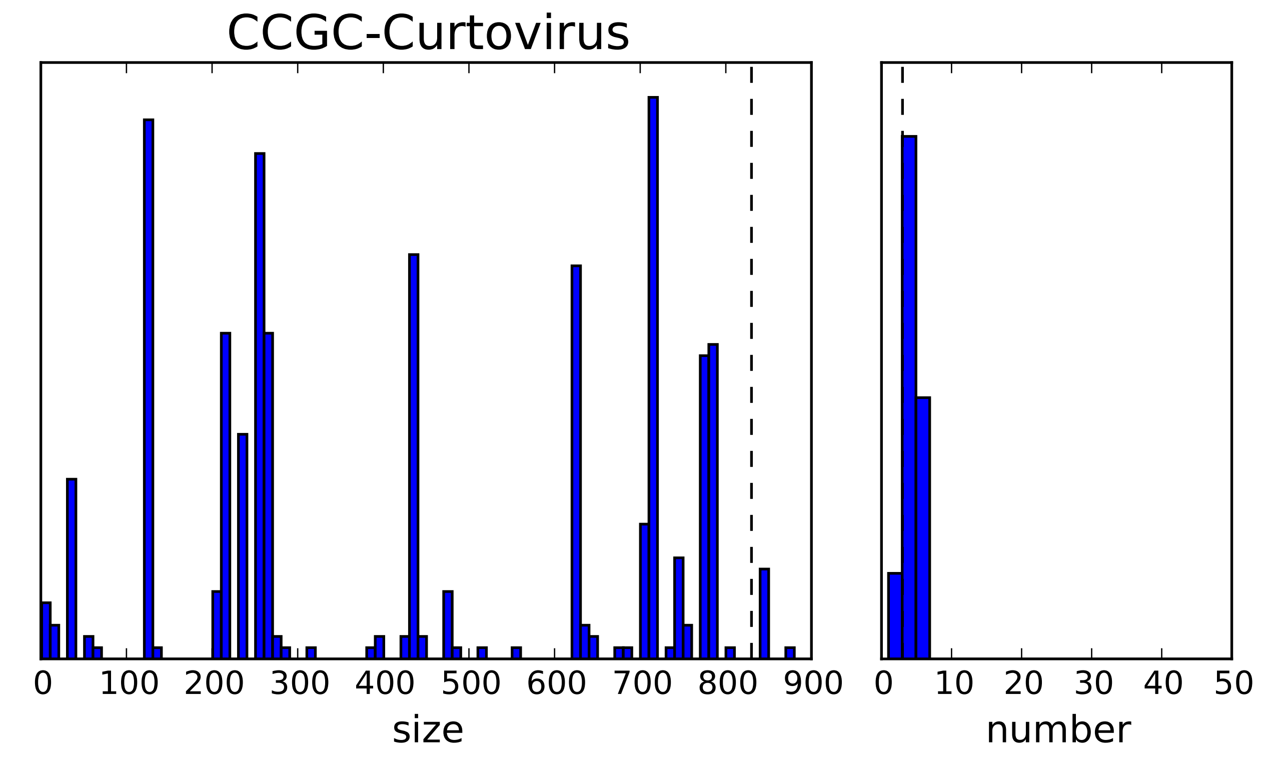
CCGC-Curtovirus
0
100
200
300
400
500
600
700
800
900
size
0
10
20
30
40
50
number

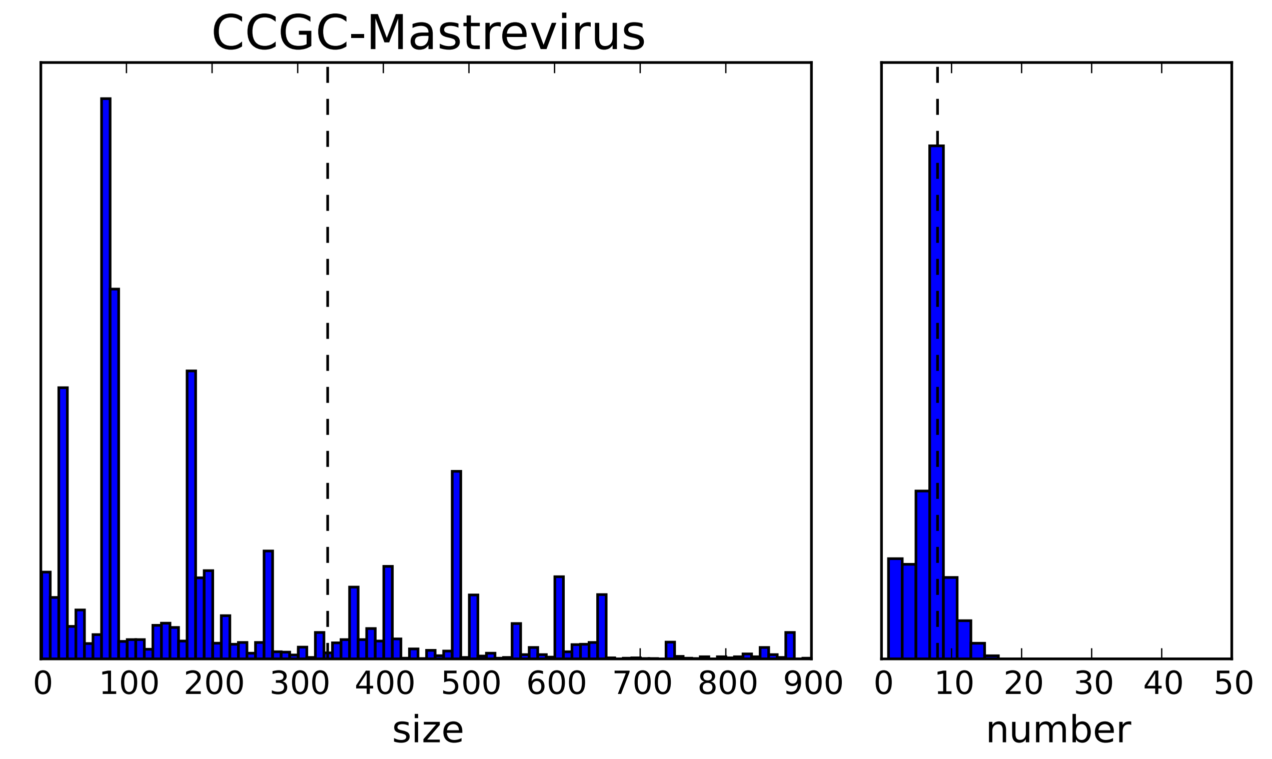
CCGC-Mastrevirus
0
100
200
300
400
500
600
700
800
900
size
0
10
20
30
40
50
number

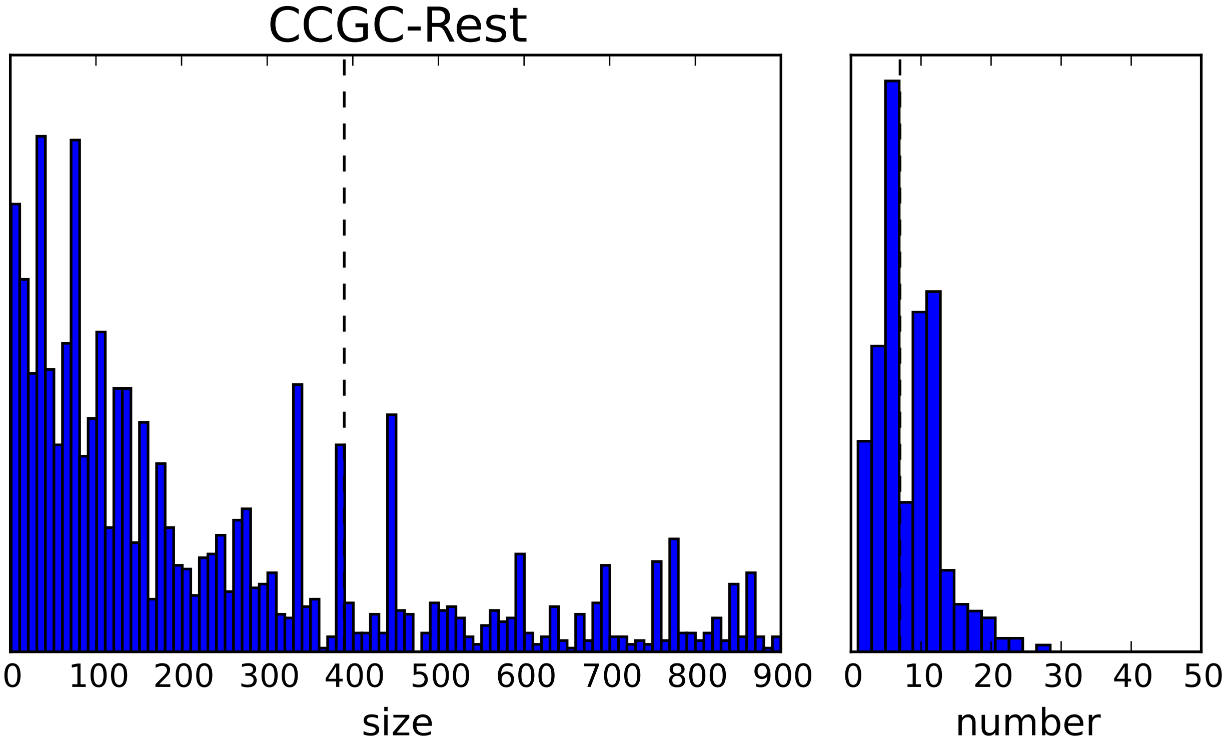

CCGC-Rest
0
100
200
300
400
500
600
700
800
900
size
0
10
20
30
40
50
number

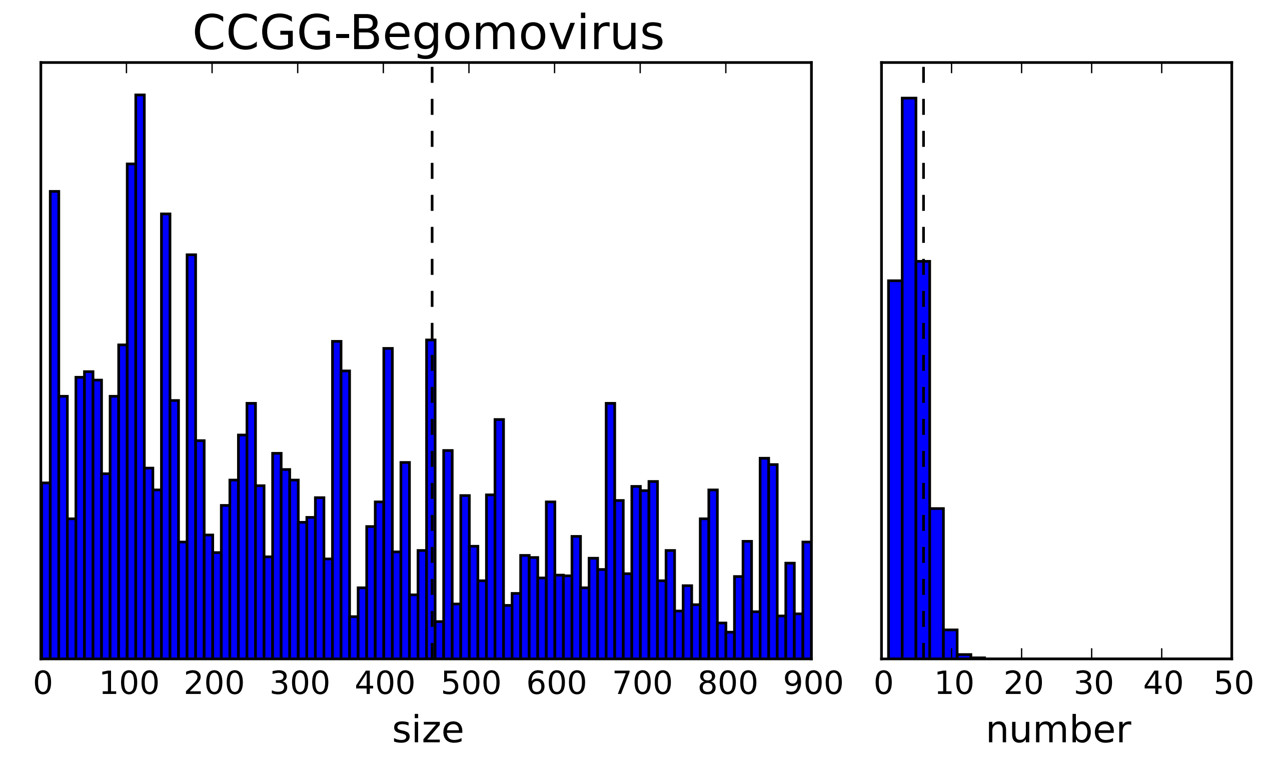
CCGG-Begomovirus
0
100
200
300
400
500
600
700
800
900
size
0
10
20
30
40
50
number

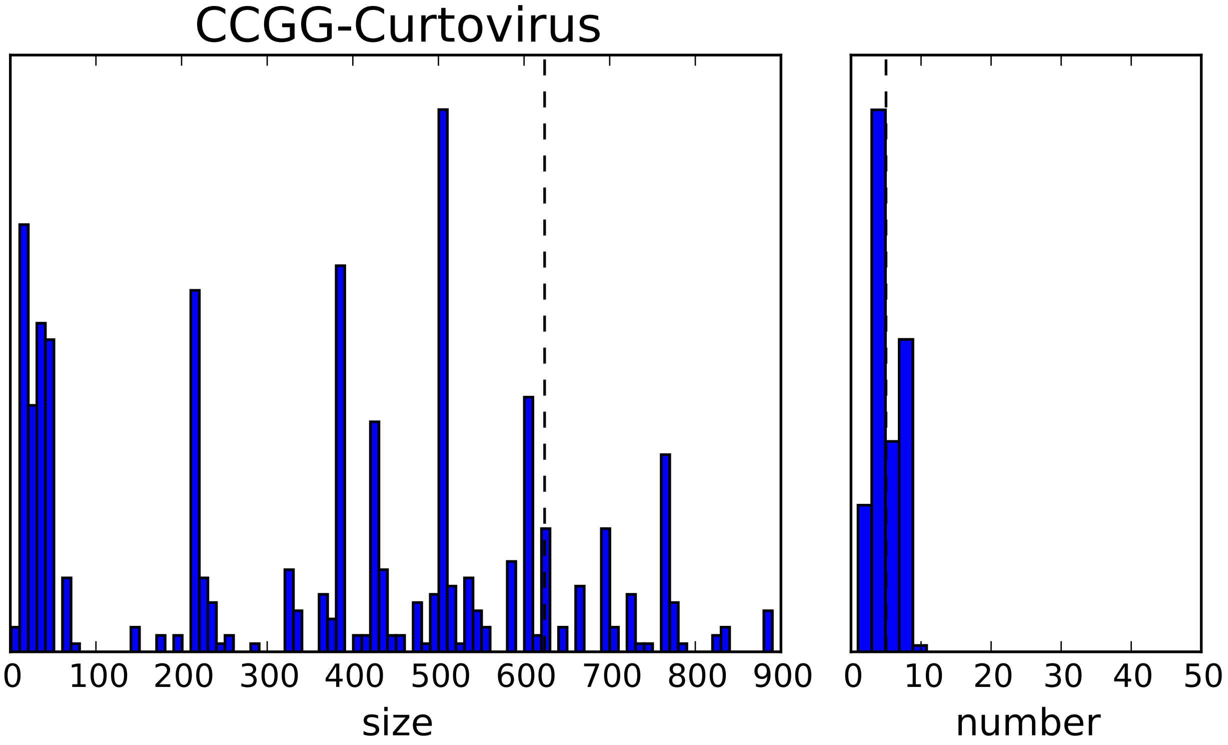
CCGG-Curtovirus
0
100
200
300
400
500
600
700
800
900
size
0
10
20
30
40
50
number

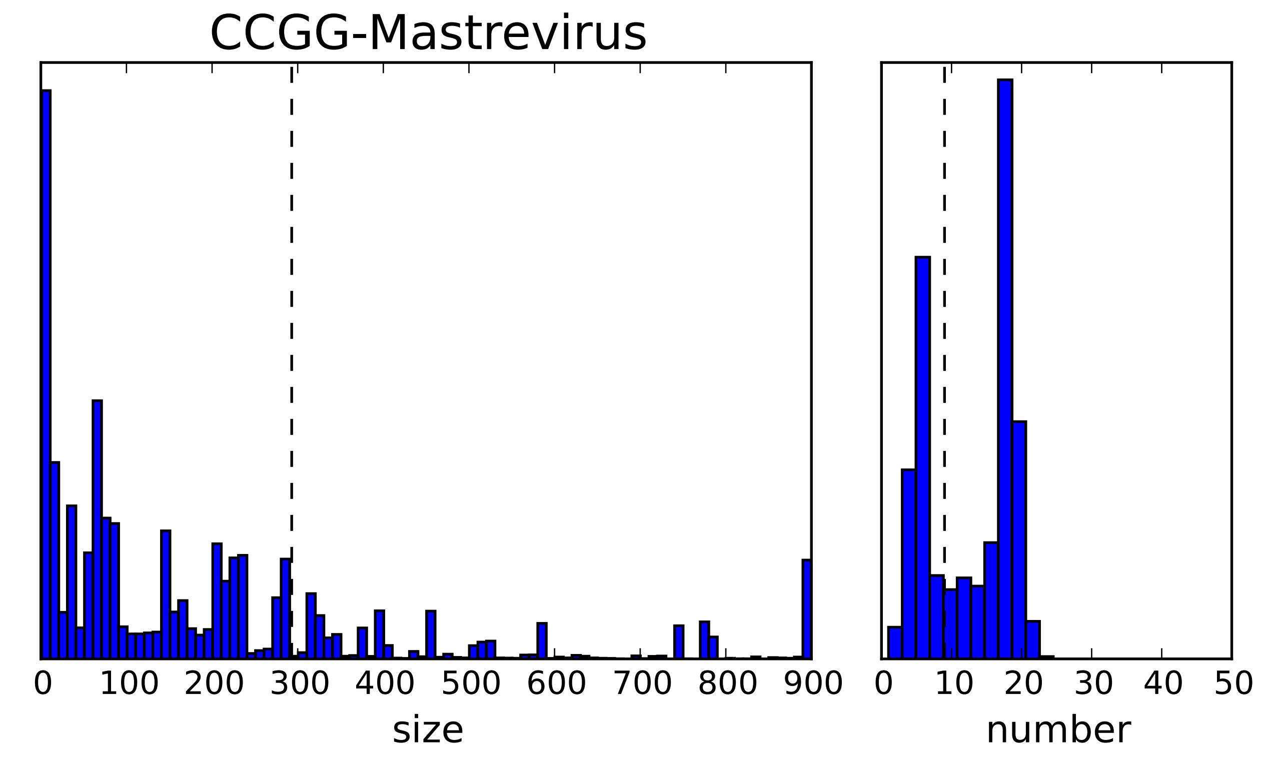

CCGG-Mastrevirus
0
100
200
300
400
500
600
700
800
900
size
0
10
20
30
40
50
number

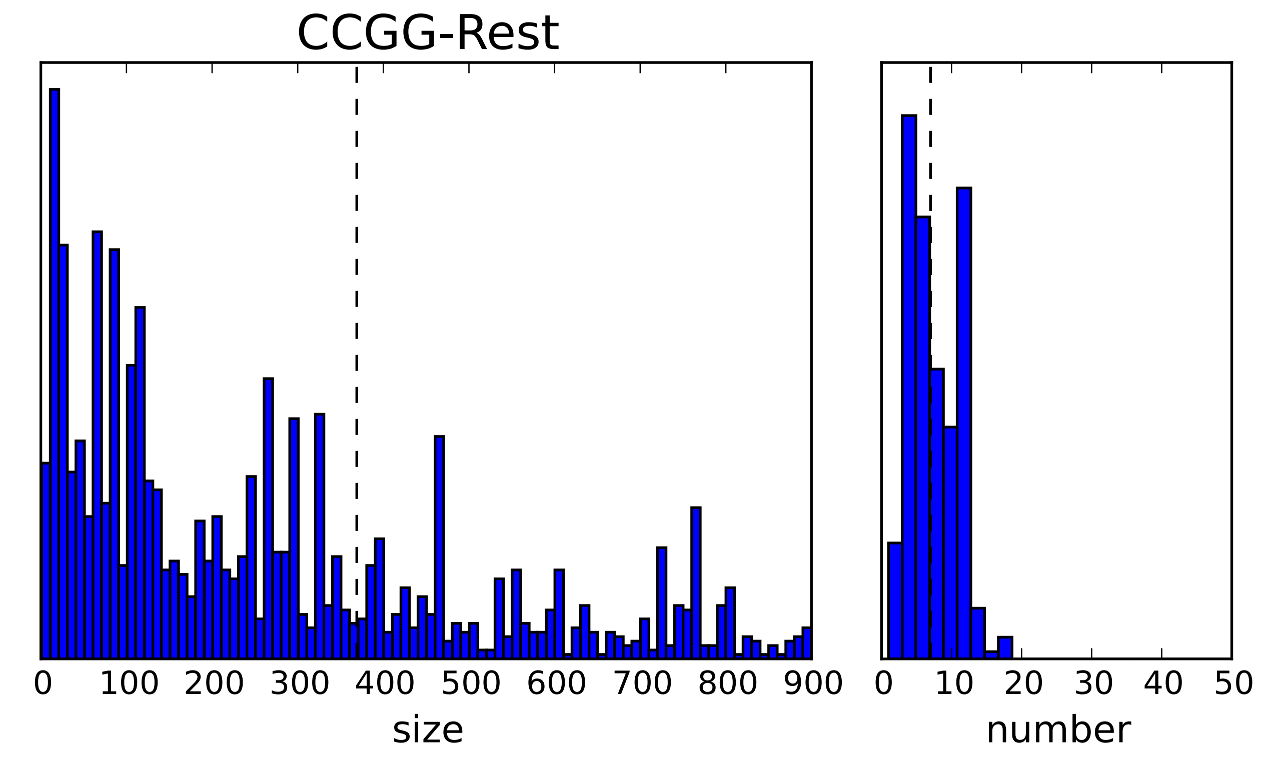

CCGG-Rest
0
100
200
300
400
500
600
700
800
900
size
0
10
20
30
40
50
number

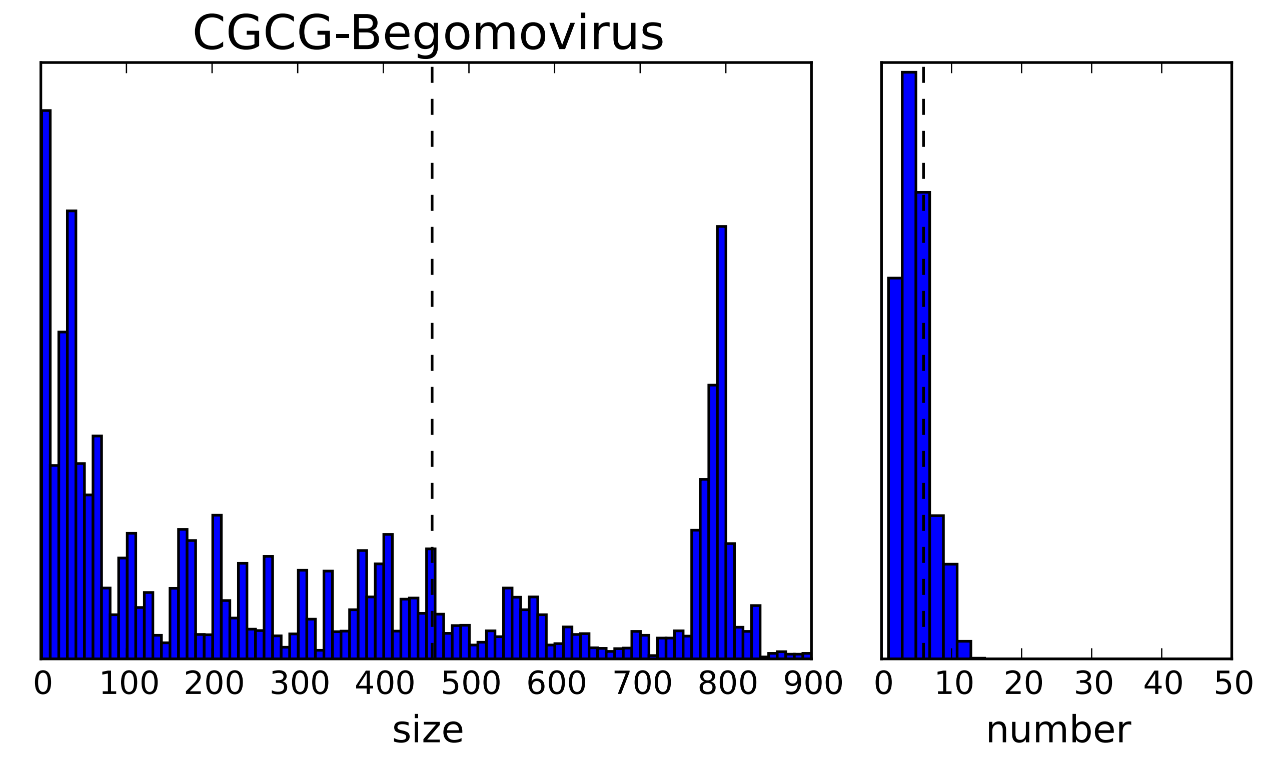

CGCG-Begomovirus
0
100
200
300
400
500
600
700
800
900
size
0
10
20
30
40
50
number

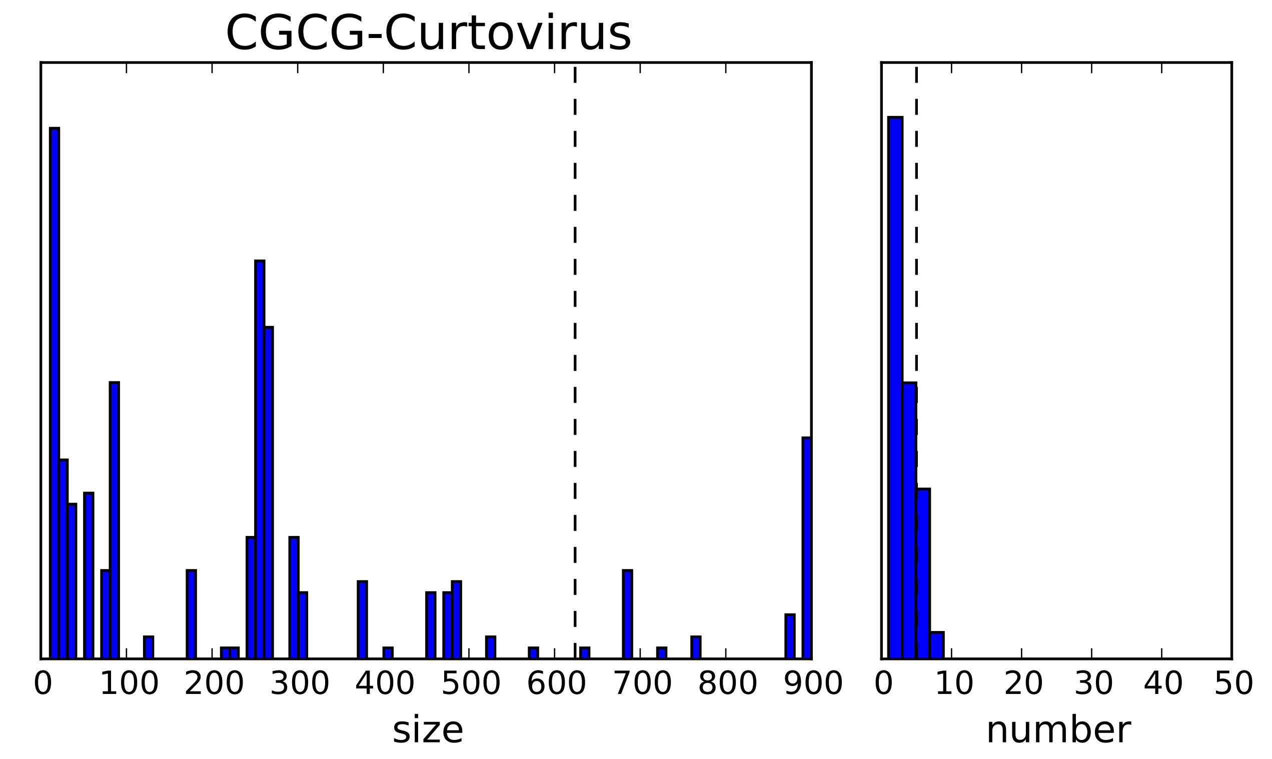
CGCG-Curtovirus
0
100
200
300
400
500
600
700
800
900
size
0
10
20
30
40
50
number

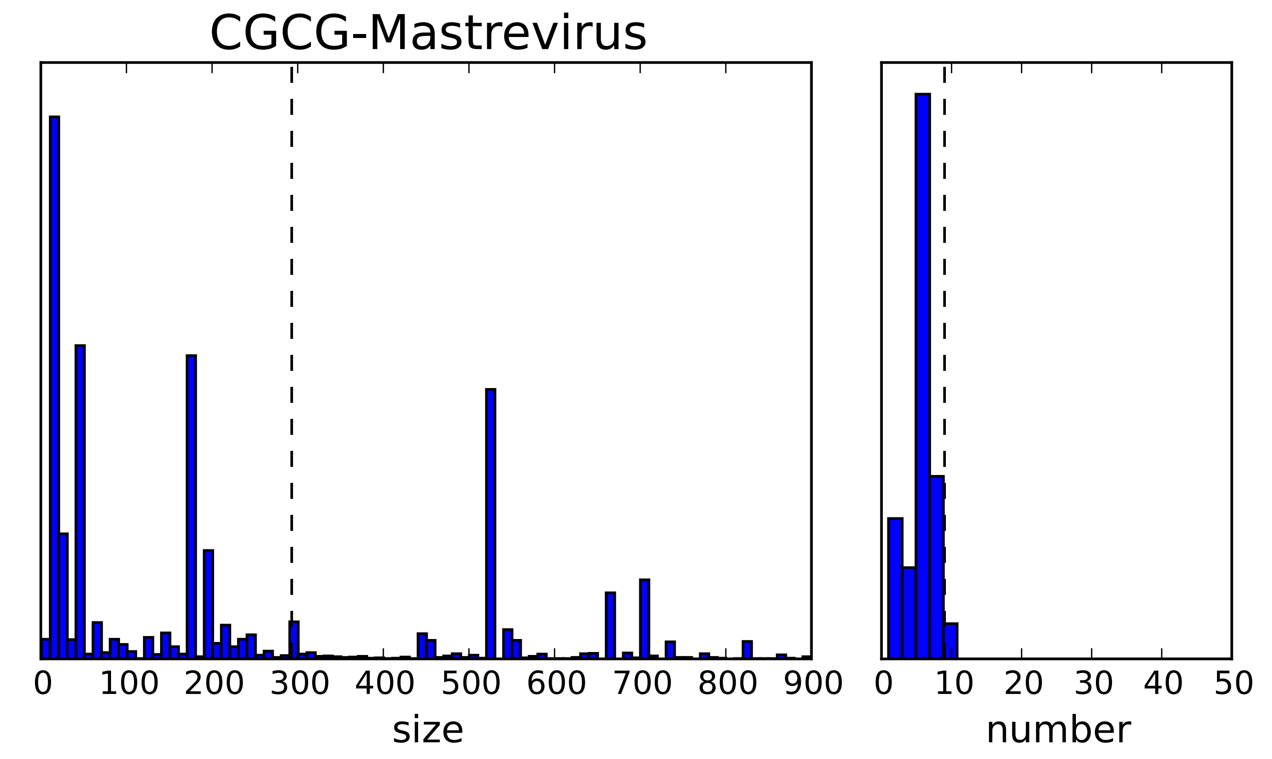
CGCG-Mastrevirus
0
100
200
300
400
500
600
700
800
900
size
0
10
20
30
40
50
number

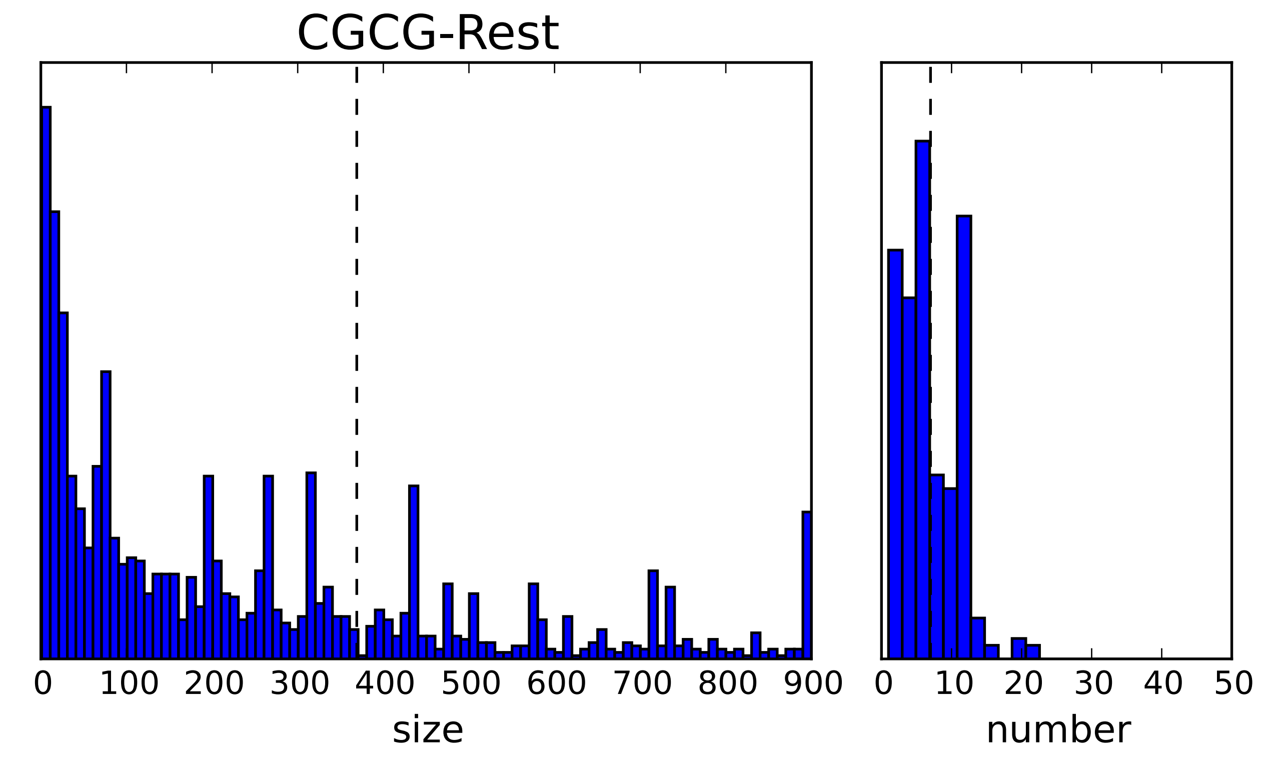
CGCG-Rest
0
100
200
300
400
500
600
700
800
900
size
0
10
20
30
40
50
number

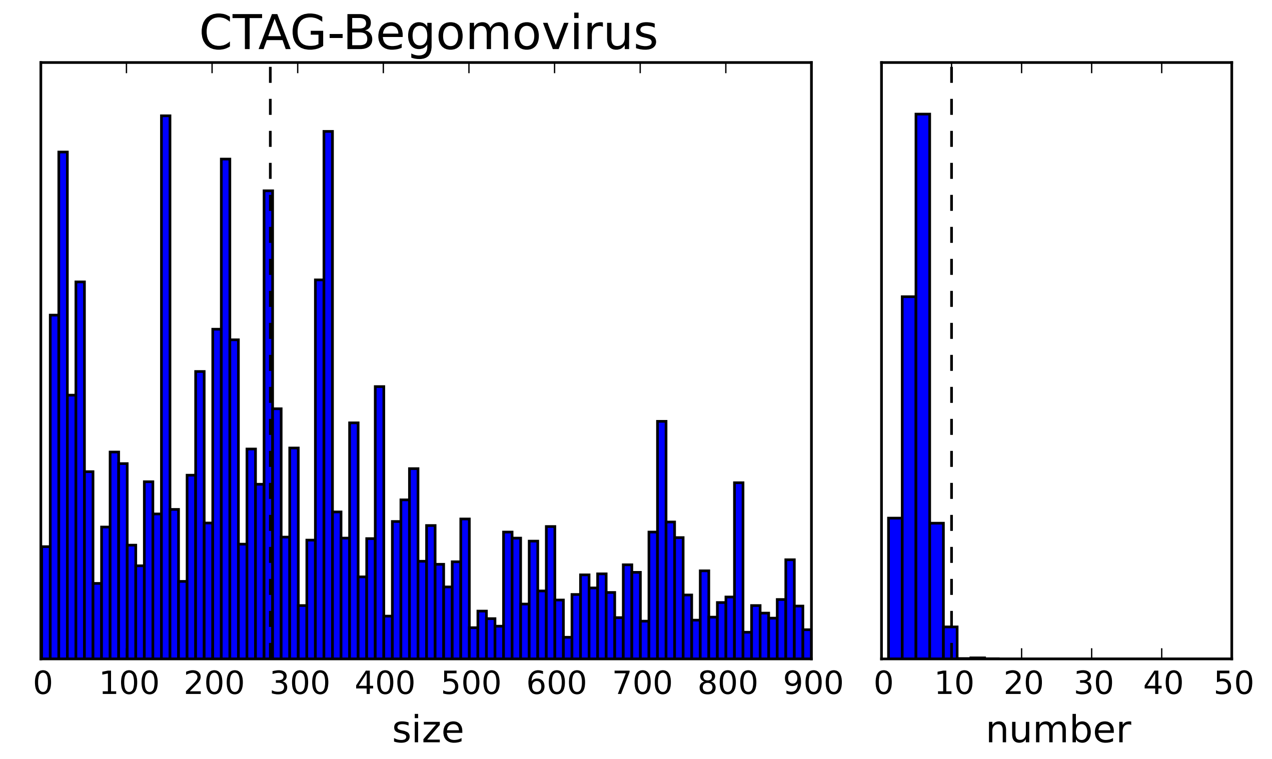
CTAG-Begomovirus
0
100
200
300
400
500
600
700
800
900
size
0
10
20
30
40
50
number

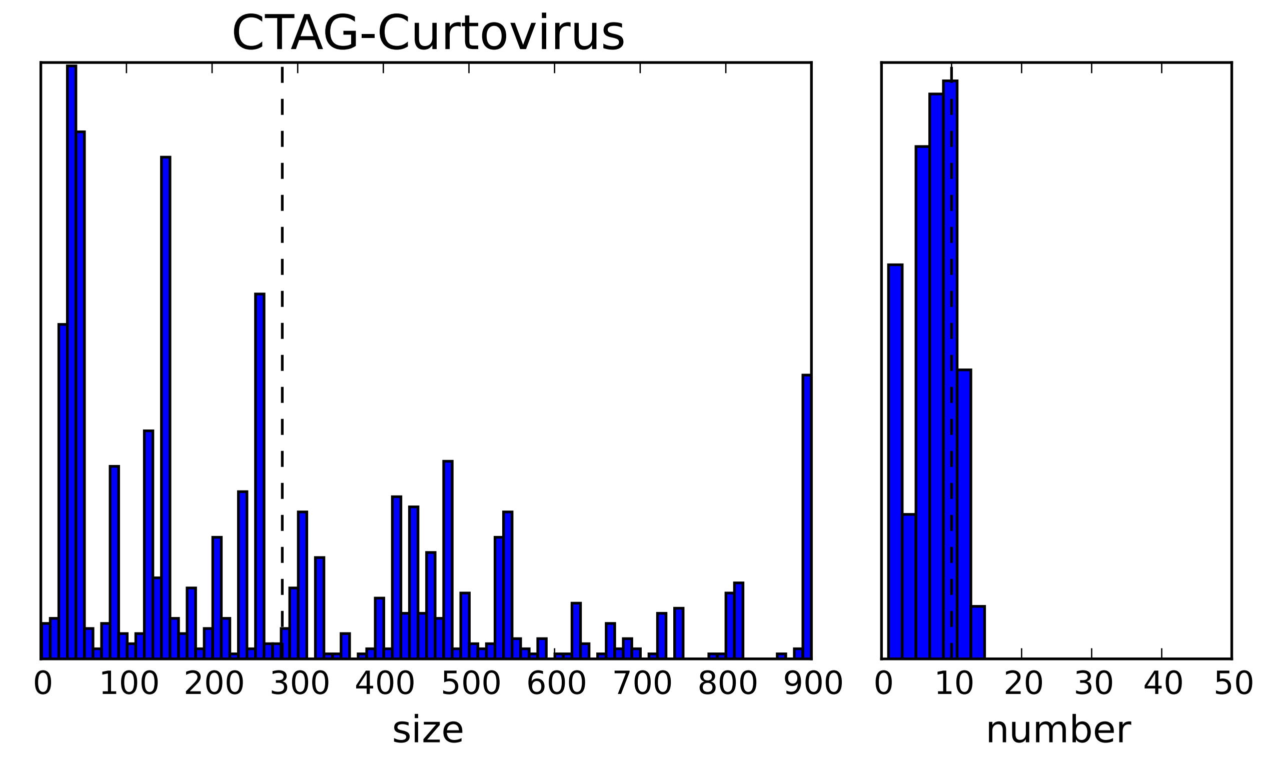
CTAG-Curtovirus
0
100
200
300
400
500
600
700
800
900
size
0
10
20
30
40
50
number

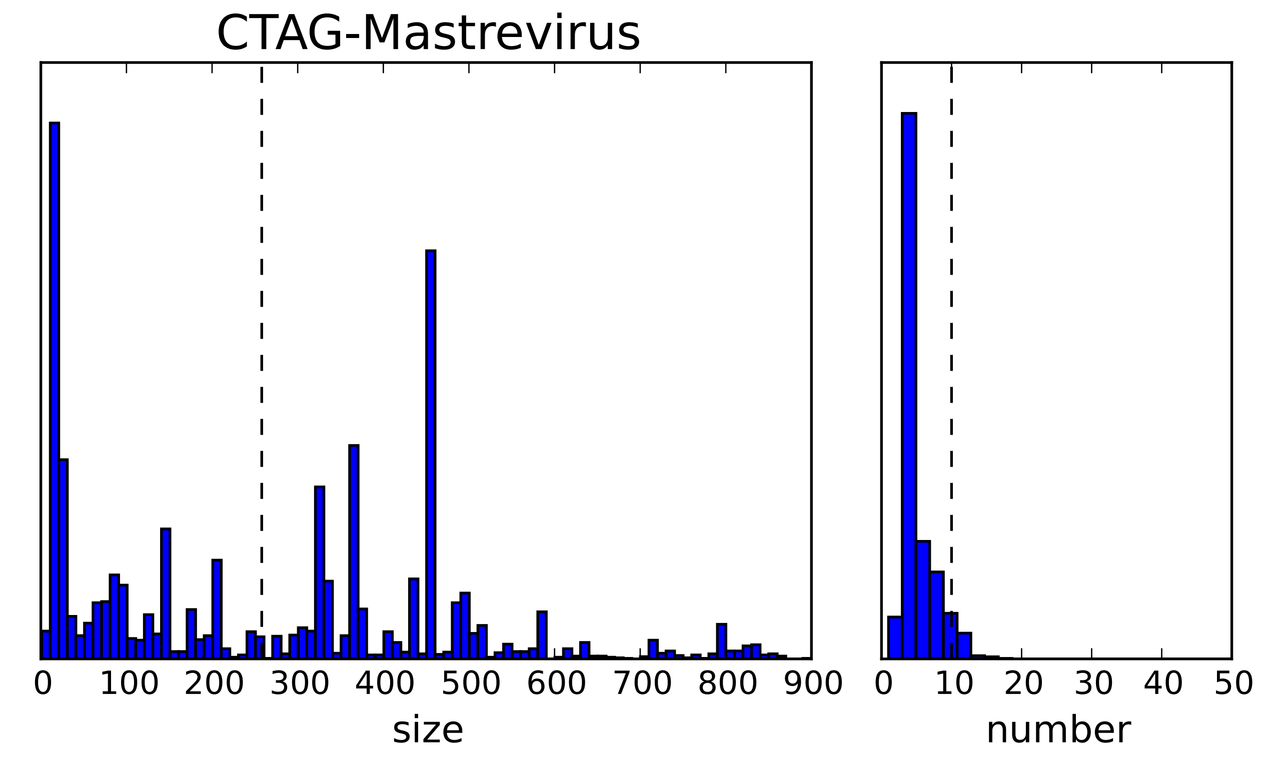
CTAG-Mastrevirus
0
100
200
300
400
500
600
700
800
900
size
0
10
20
30
40
50
number

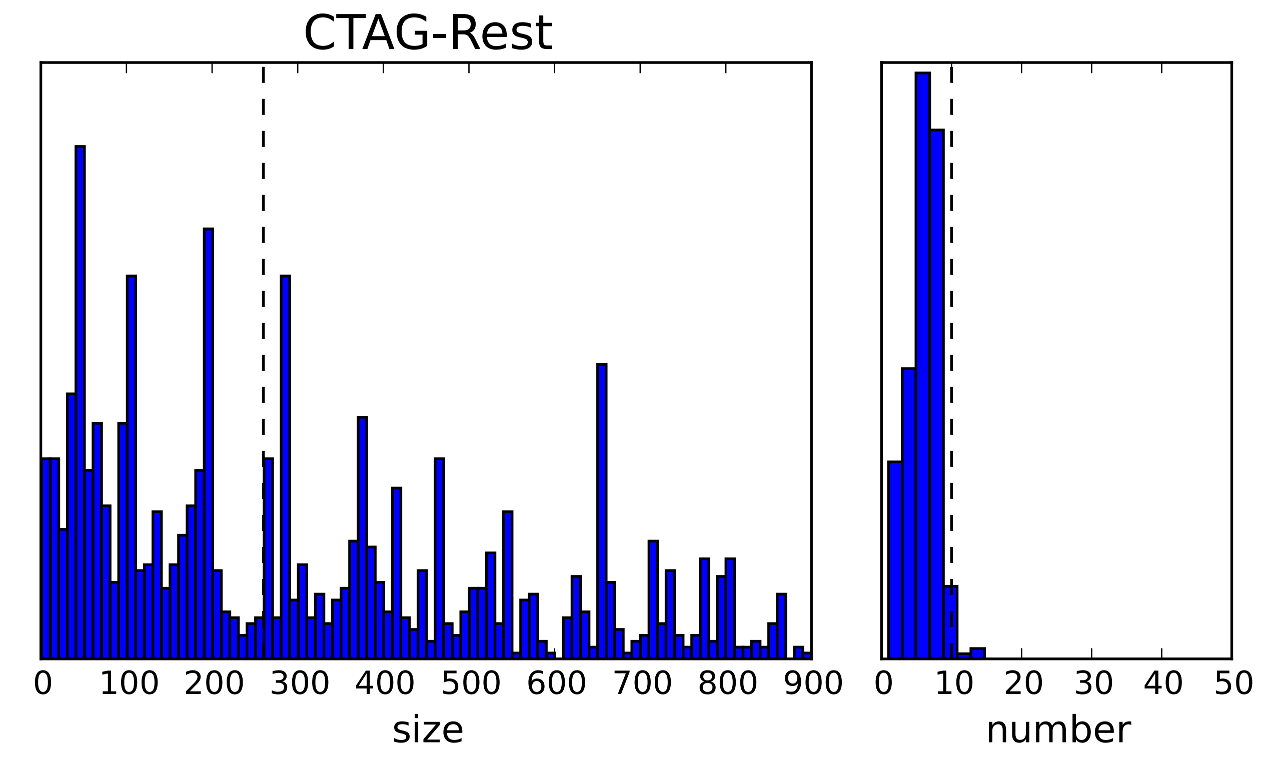
CTAG-Rest
0
100
200
300
400
500
600
700
800
900
size
0
10
20
30
40
50
number

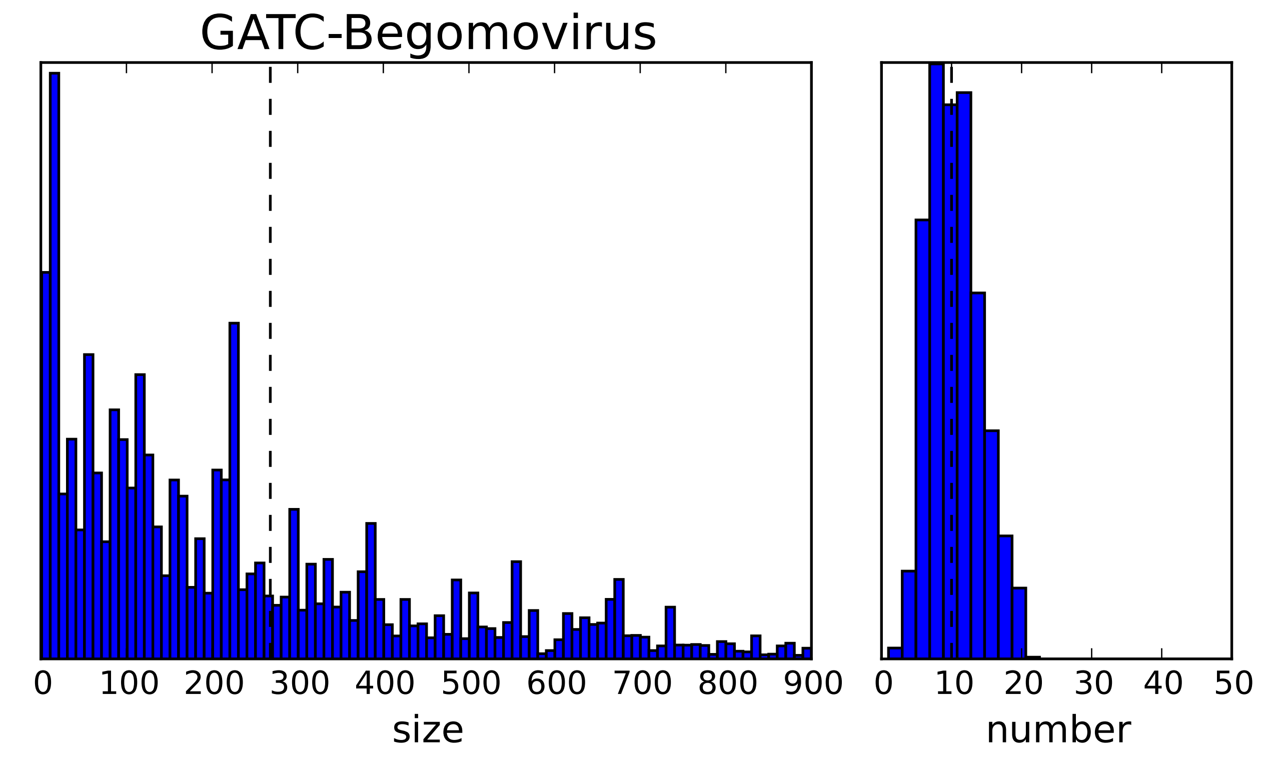
GATC-Begomovirus
0
100
200
300
400
500
600
700
800
900
size
0
10
20
30
40
50
number

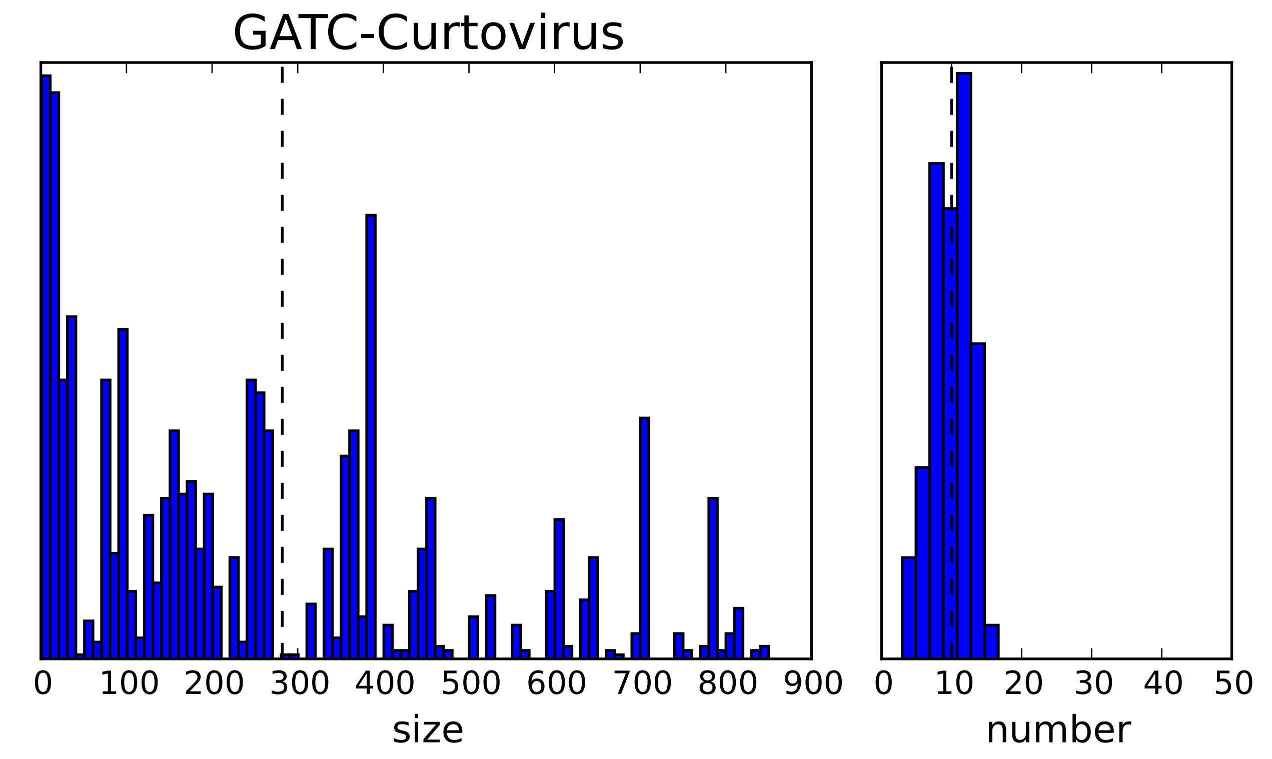
GATC-Curtovirus
0
100
200
300
400
500
600
700
800
900
size
0
10
20
30
40
50
number

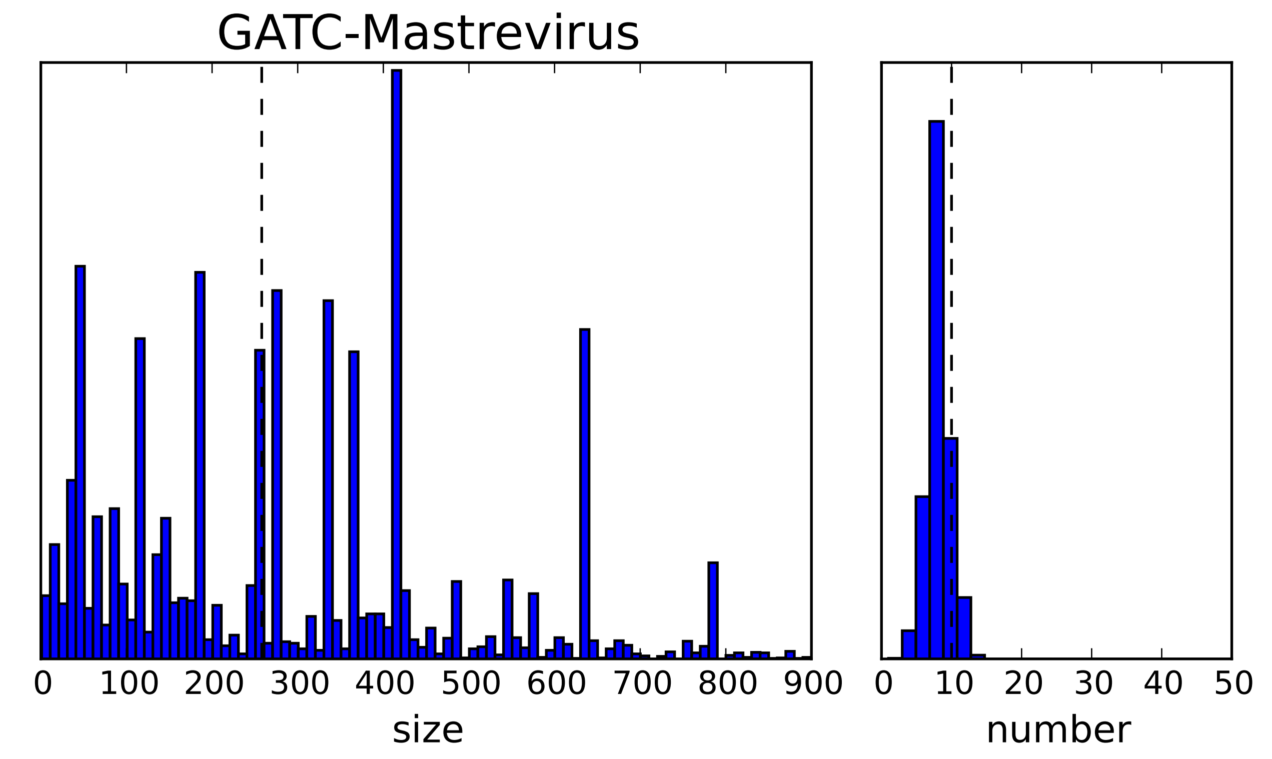
GATC-Mastrevirus
0
100
200
300
400
500
600
700
800
900
size
0
10
20
30
40
50
number

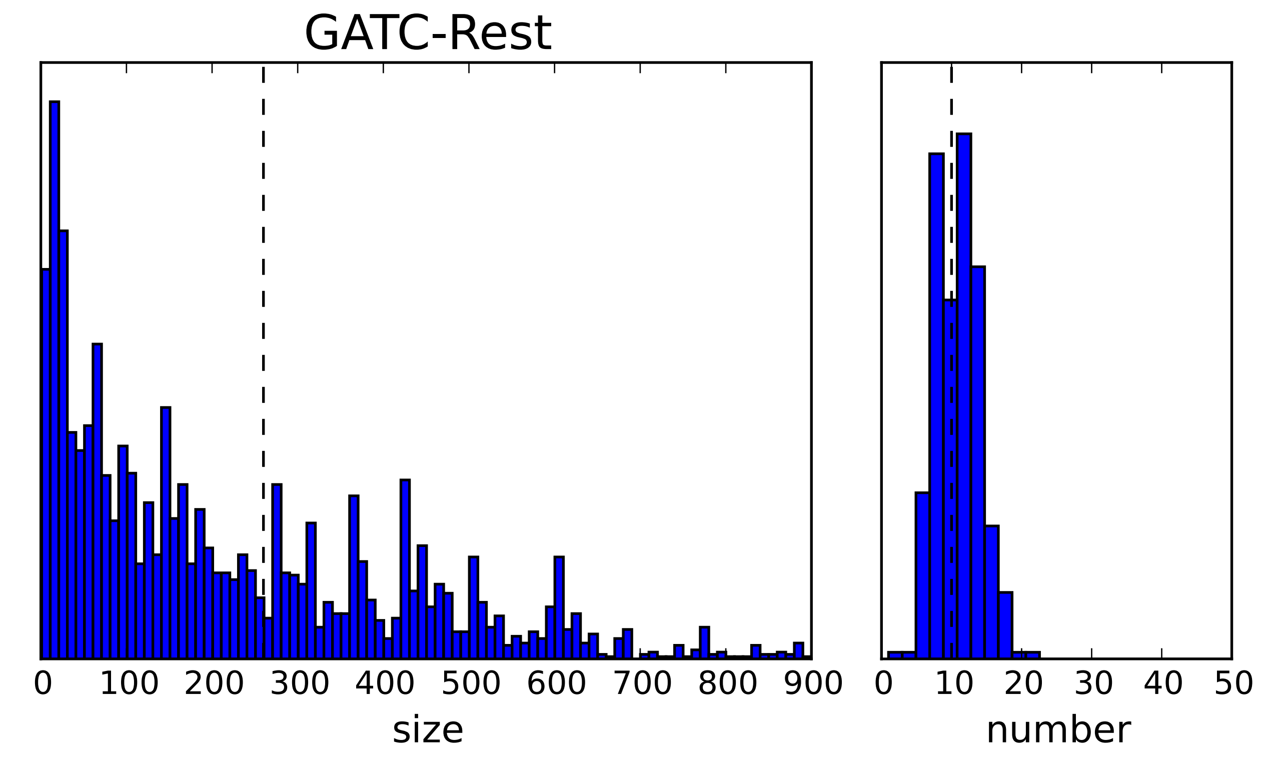
GATC-Rest
0
100
200
300
400
500
600
700
800
900
size
0
10
20
30
40
50
number

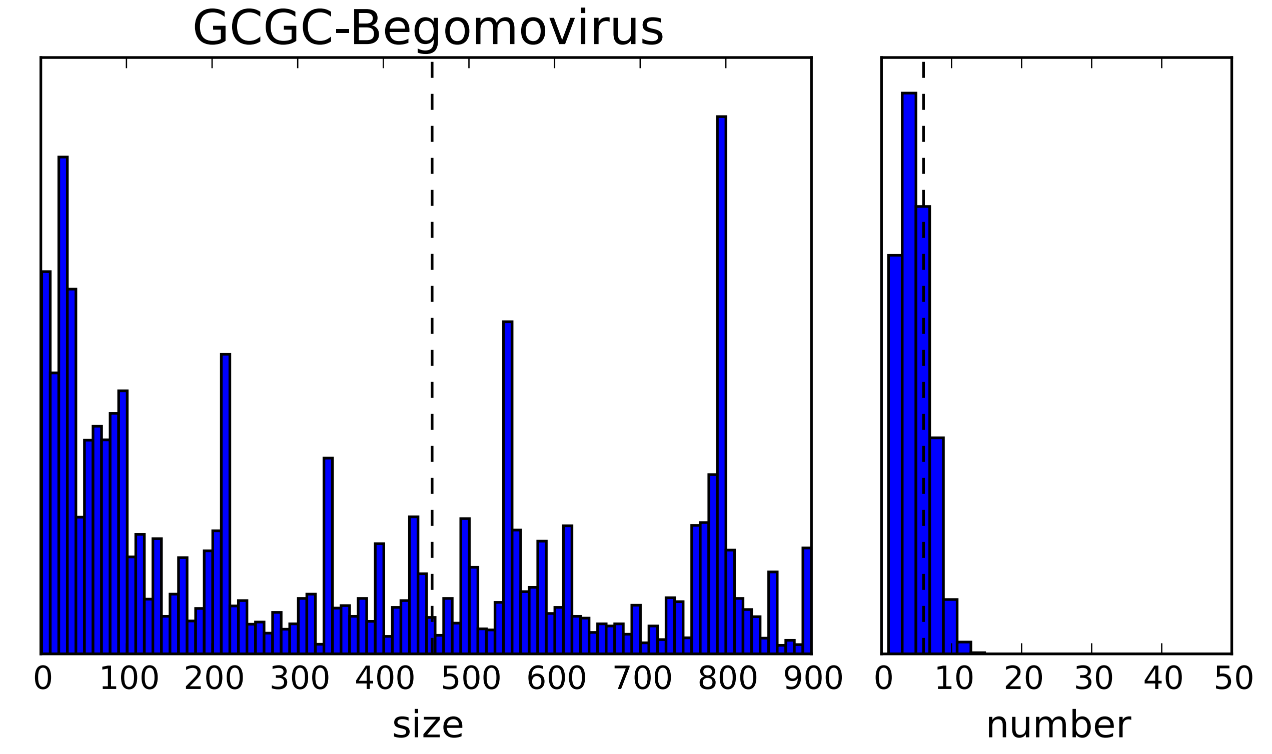
GCGC-Begomovirus
0
100
200
300
400
500
600
700
800
900
size
0
10
20
30
40
50
number

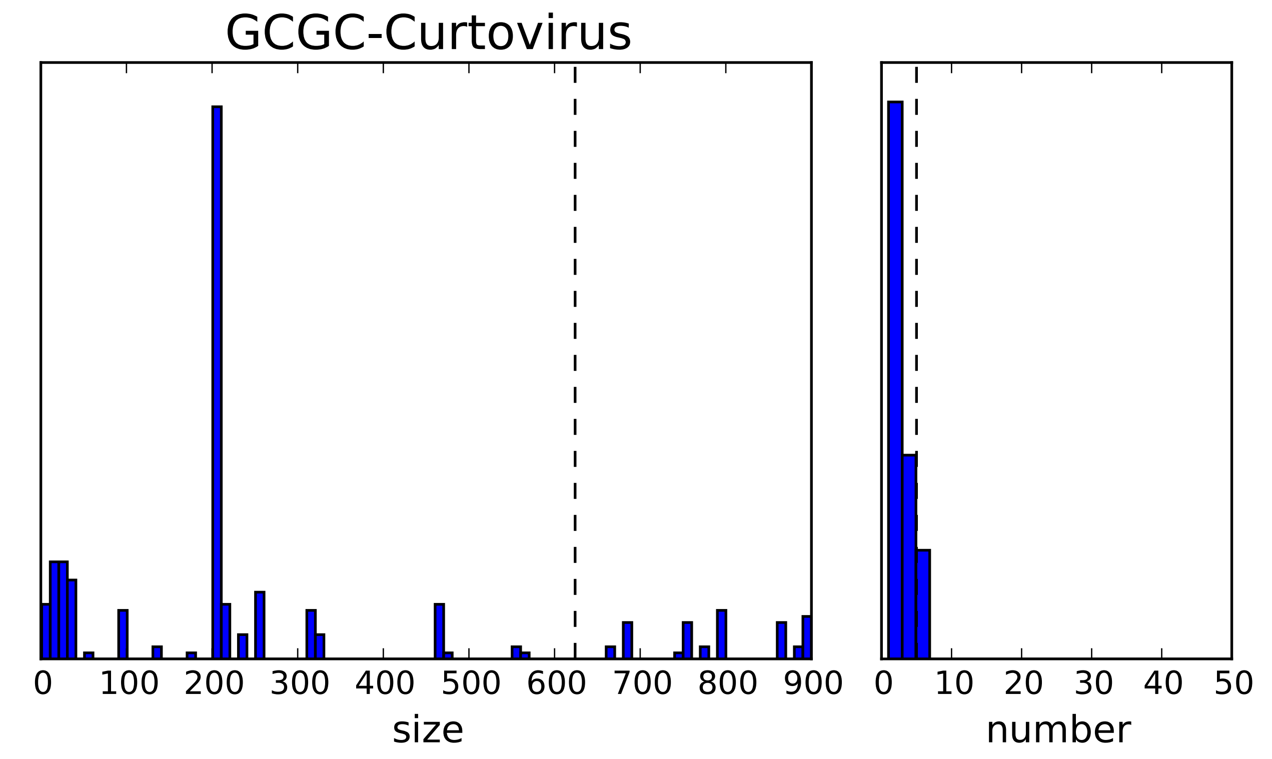
GCGC-Curtovirus
0
100
200
300
400
500
600
700
800
900
size
0
10
20
30
40
50
number

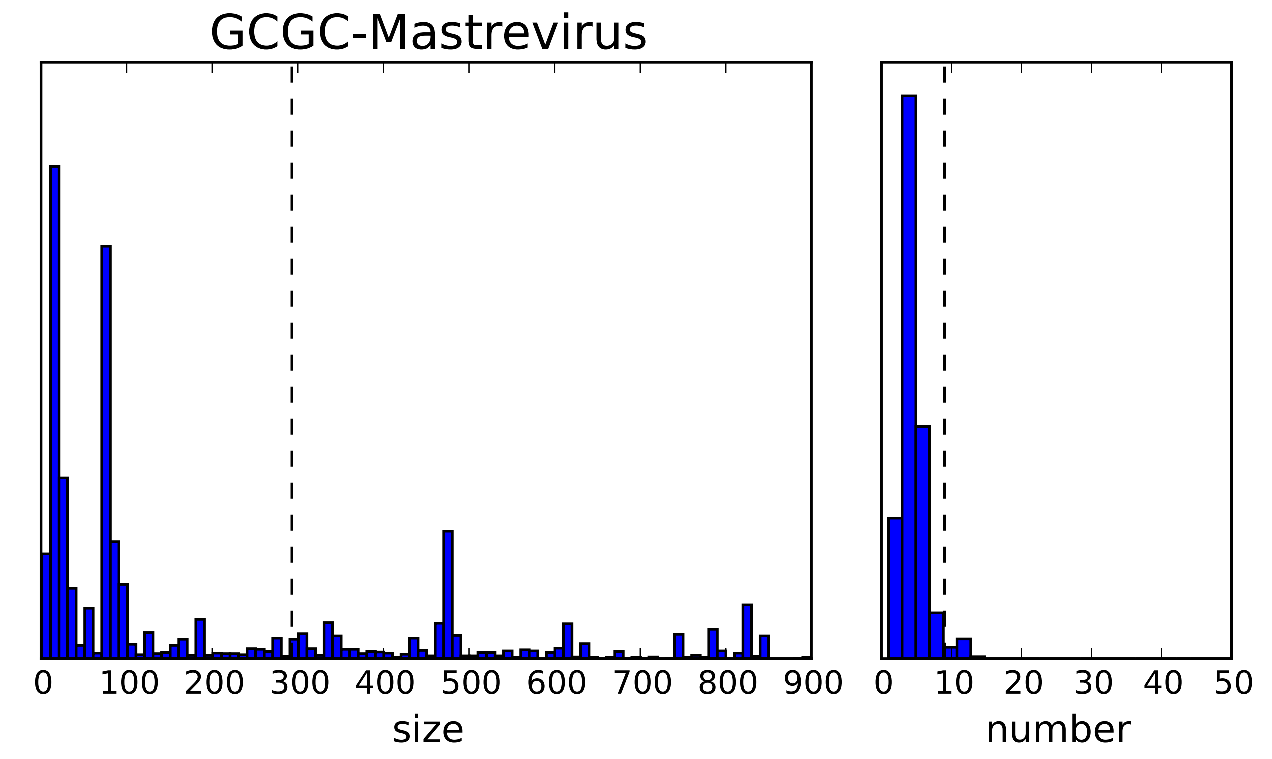
GCGC-Mastrevirus
0
100
200
300
400
500
600
700
800
900
size
0
10
20
30
40
50
number

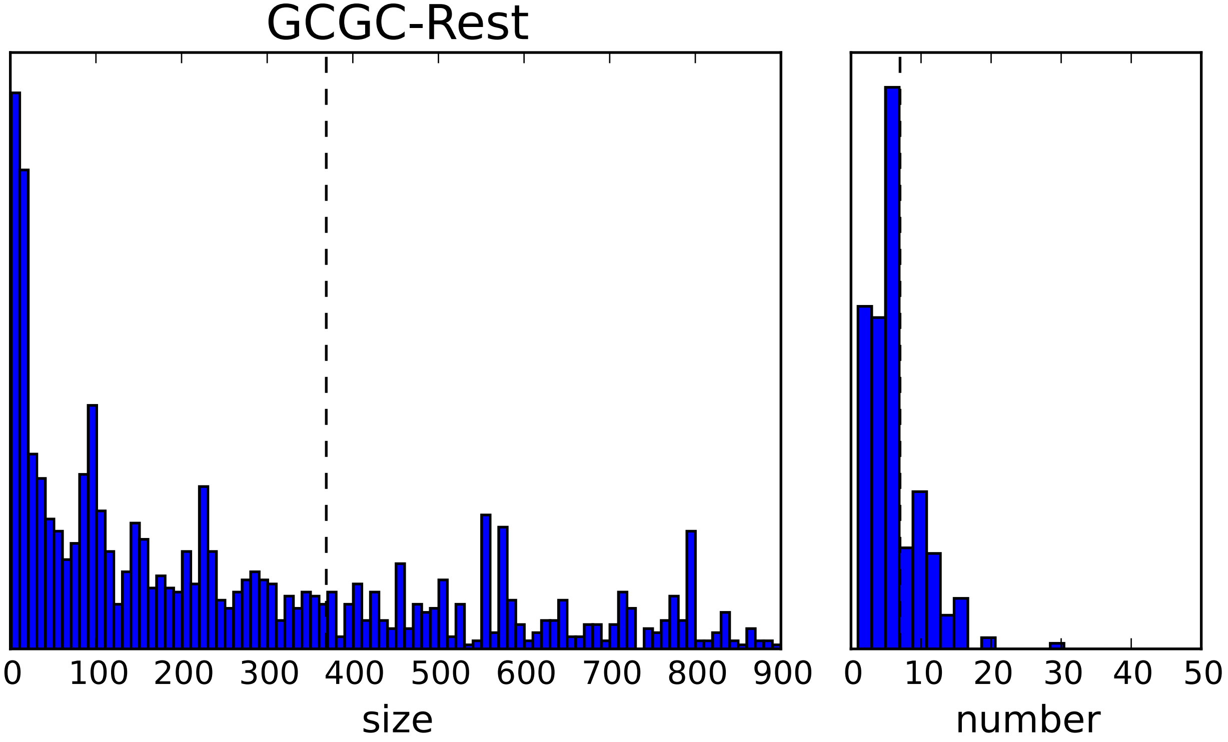
GCGC-Rest
0
100
200
300
400
500
600
700
800
900
size
0
10
20
30
40
50
number

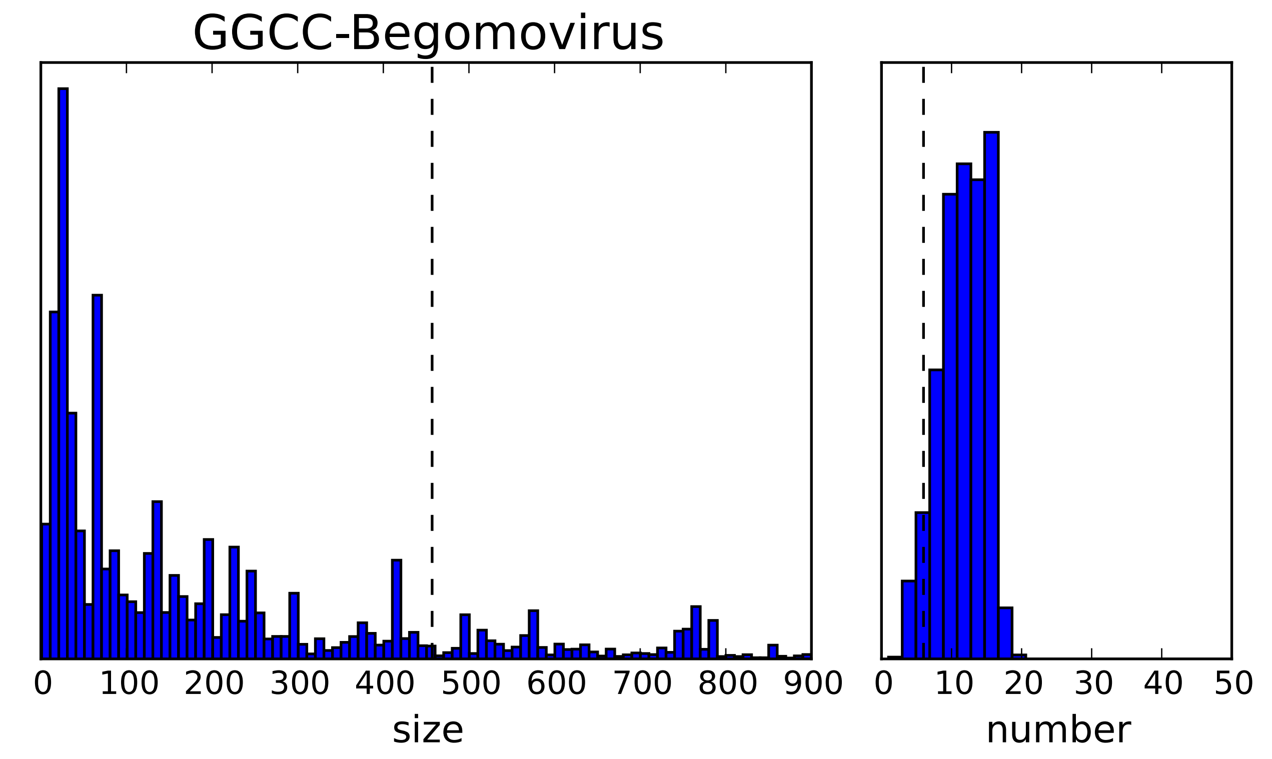
GGCC-Begomovirus
0
100
200
300
400
500
600
700
800
900
size
0
10
20
30
40
50
number

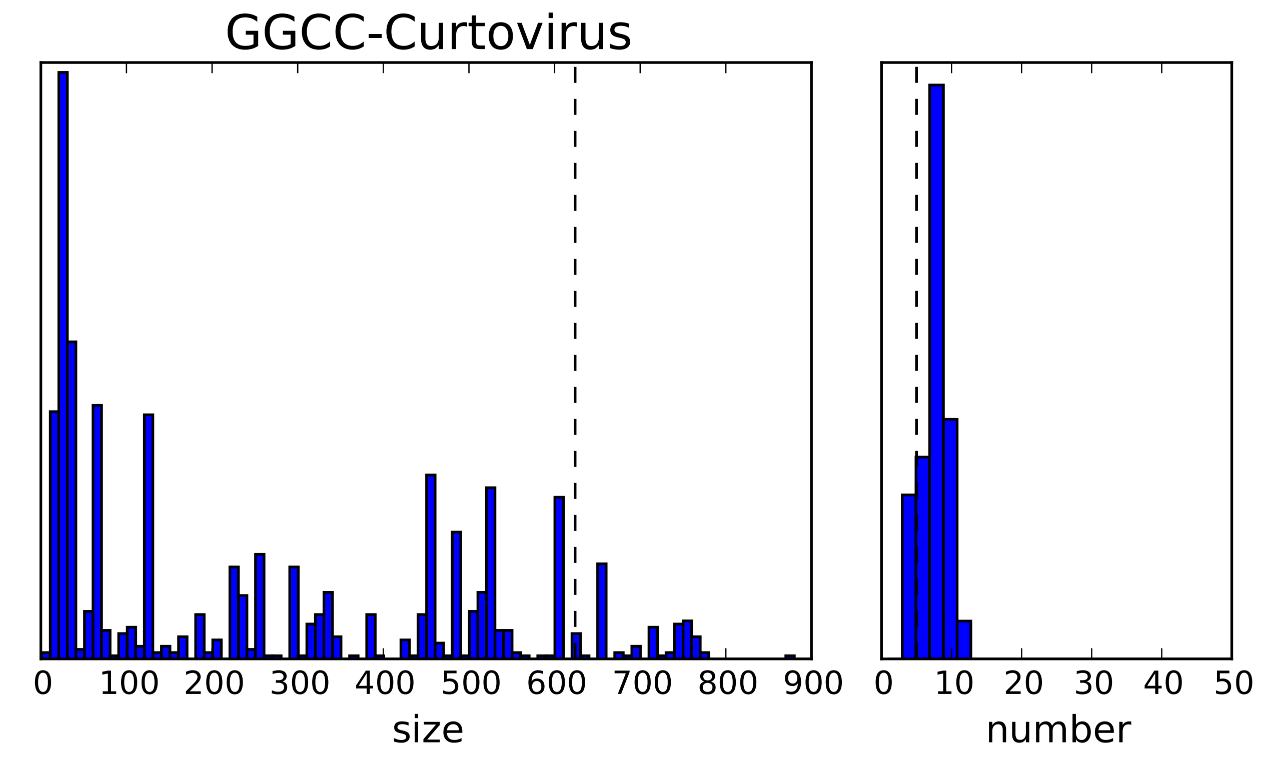
GGCC-Curtovirus
0
100
200
300
400
500
600
700
800
900
size
0
10
20
30
40
50
number

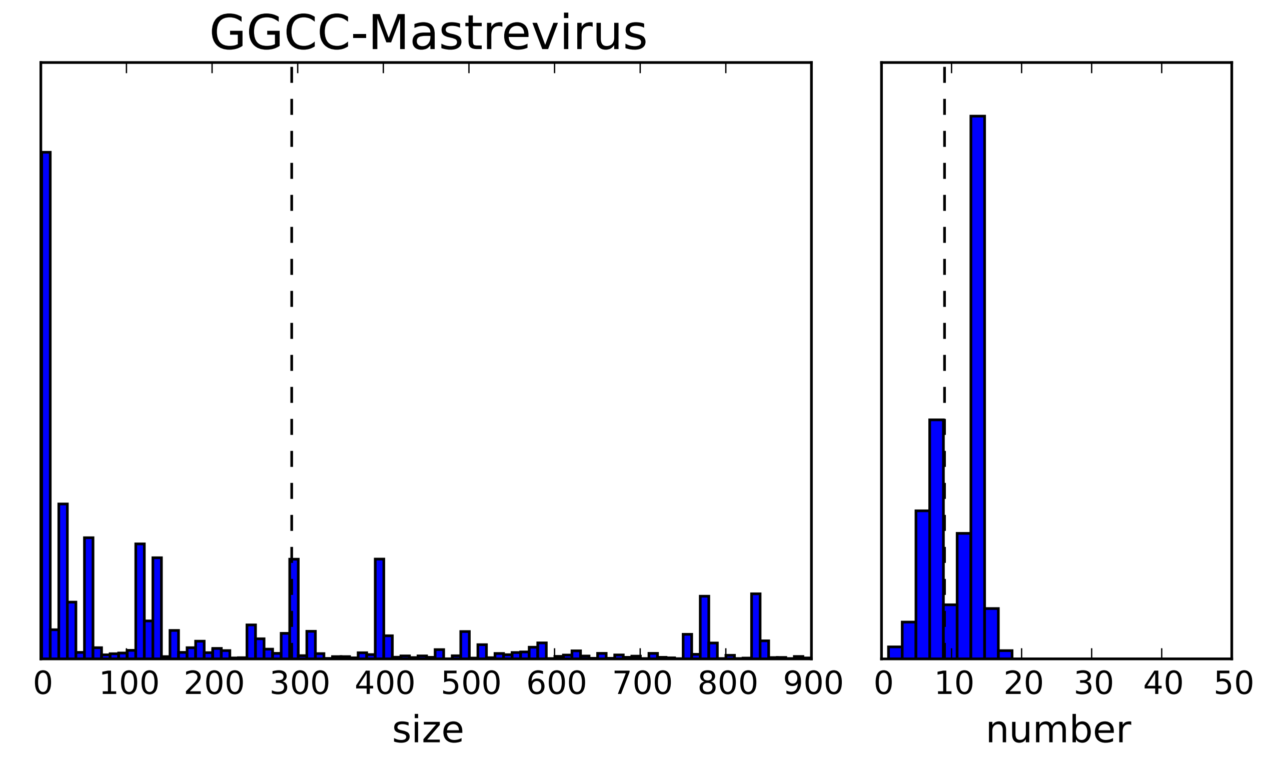
GGCC-Mastrevirus
0
100
200
300
400
500
600
700
800
900
size
0
10
20
30
40
50
number

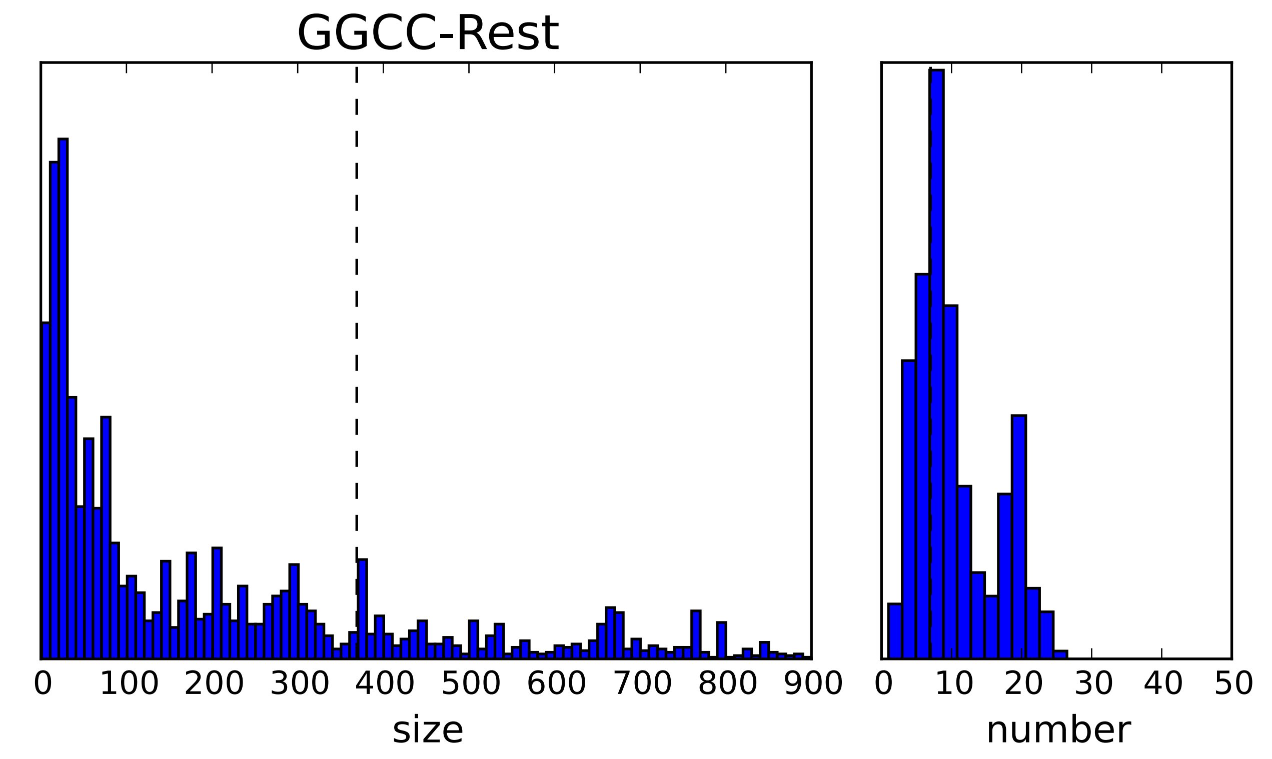

GGCC-Rest
0
100
200
300
400
500
600
700
800
900
size
0
10
20
30
40
50
number

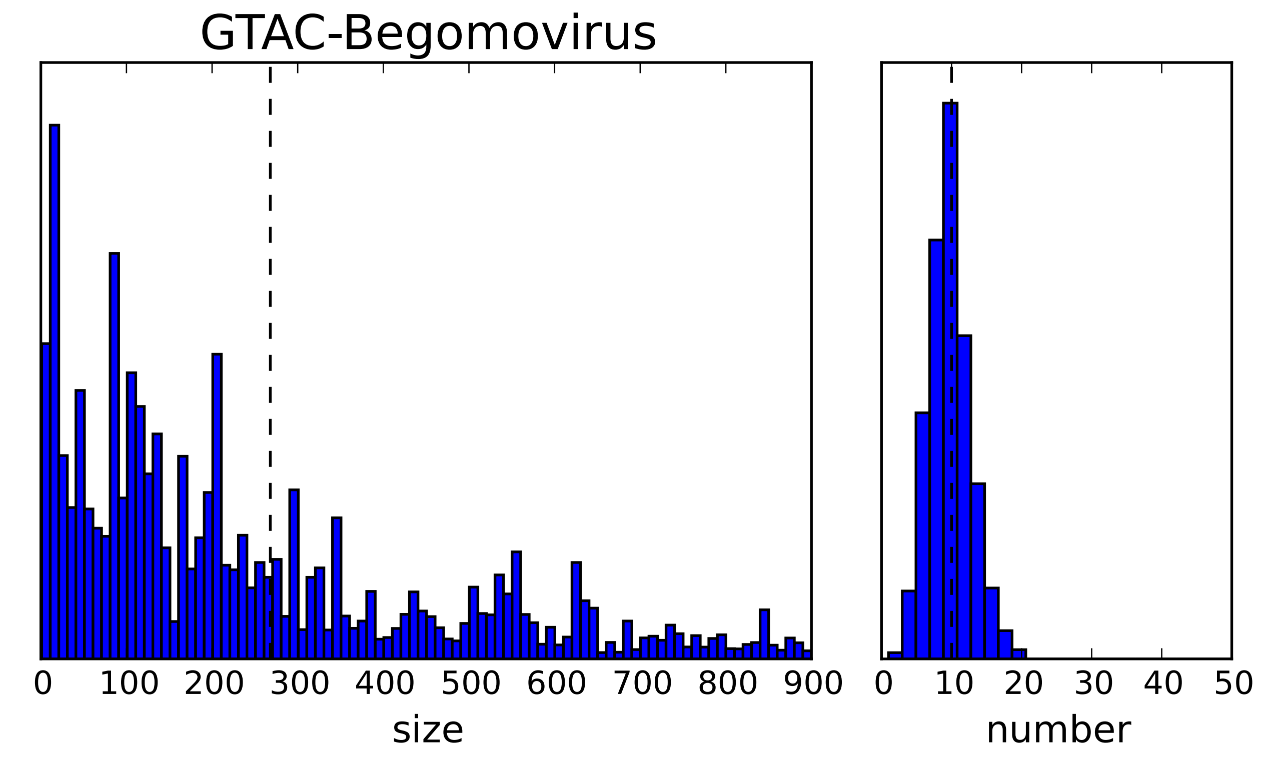
GTAC-Begomovirus
0
100
200
300
400
500
600
700
800
900
size
0
10
20
30
40
50
number

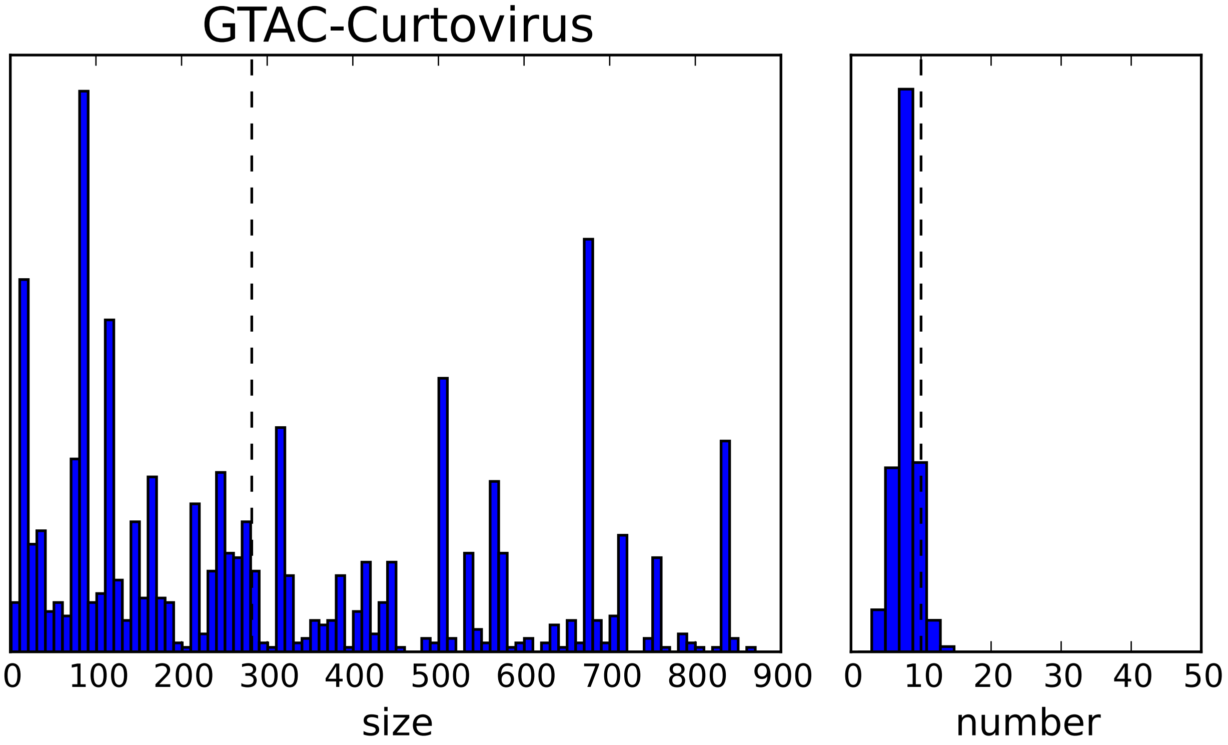
GTAC-Curtovirus
0
100
200
300
400
500
600
700
800
900
size
0
10
20
30
40
50
number

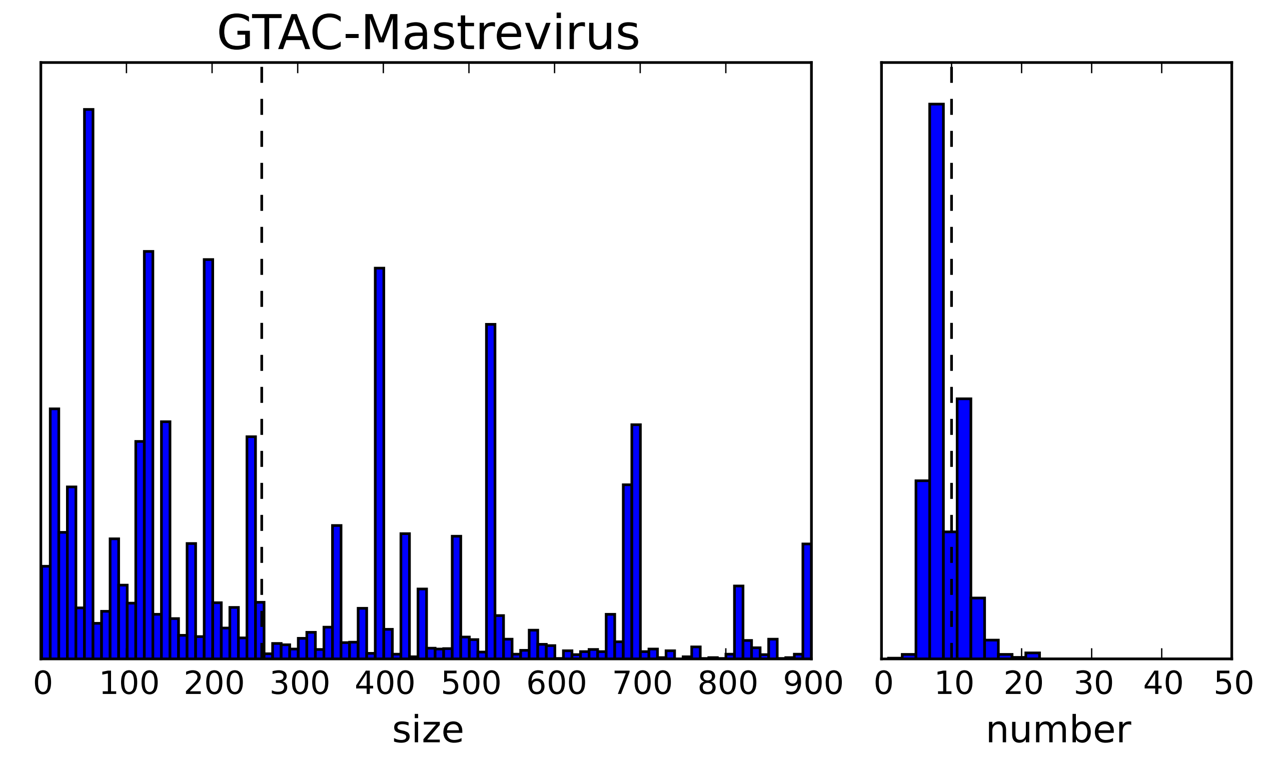
GTAC-Mastrevirus
0
100
200
300
400
500
600
700
800
900
size
0
10
20
30
40
50
number

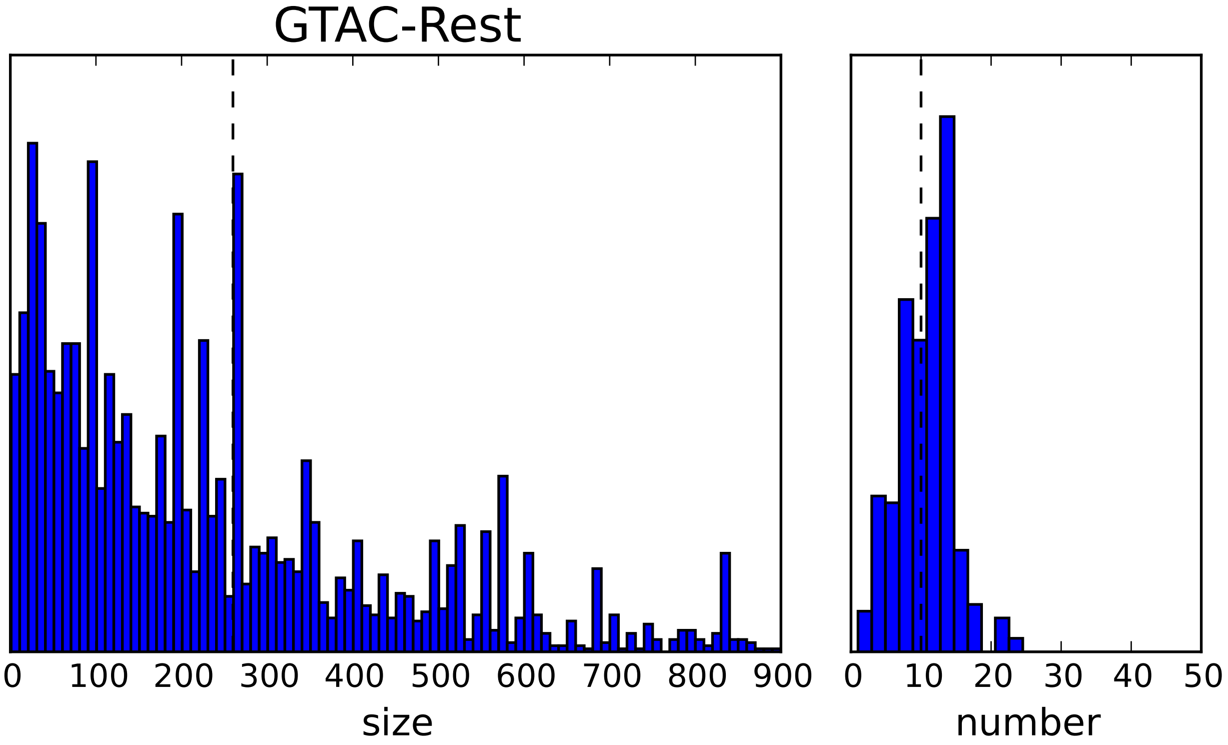
GTAC-Rest
0
100
200
300
400
500
600
700
800
900
size
0
10
20
30
40
50
number

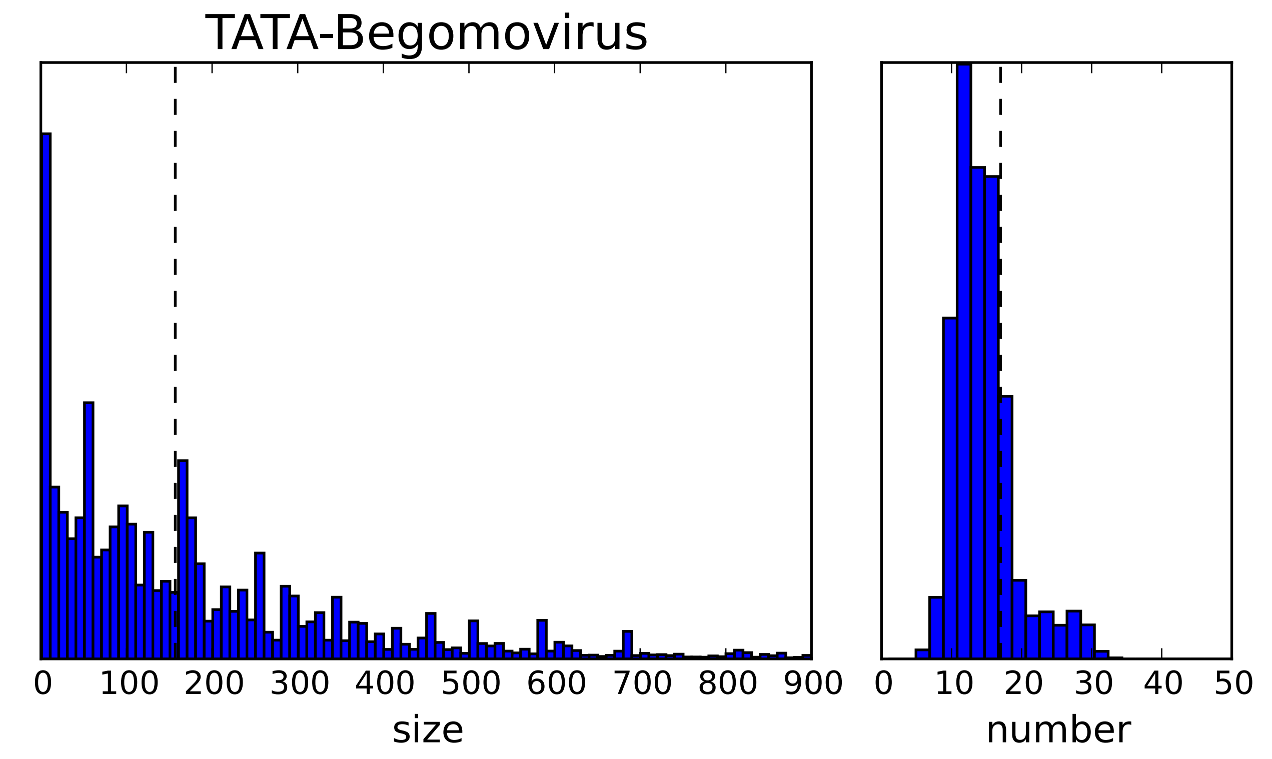

TATA-Begomovirus
0
100
200
300
400
500
600
700
800
900
size
0
10
20
30
40
50
number

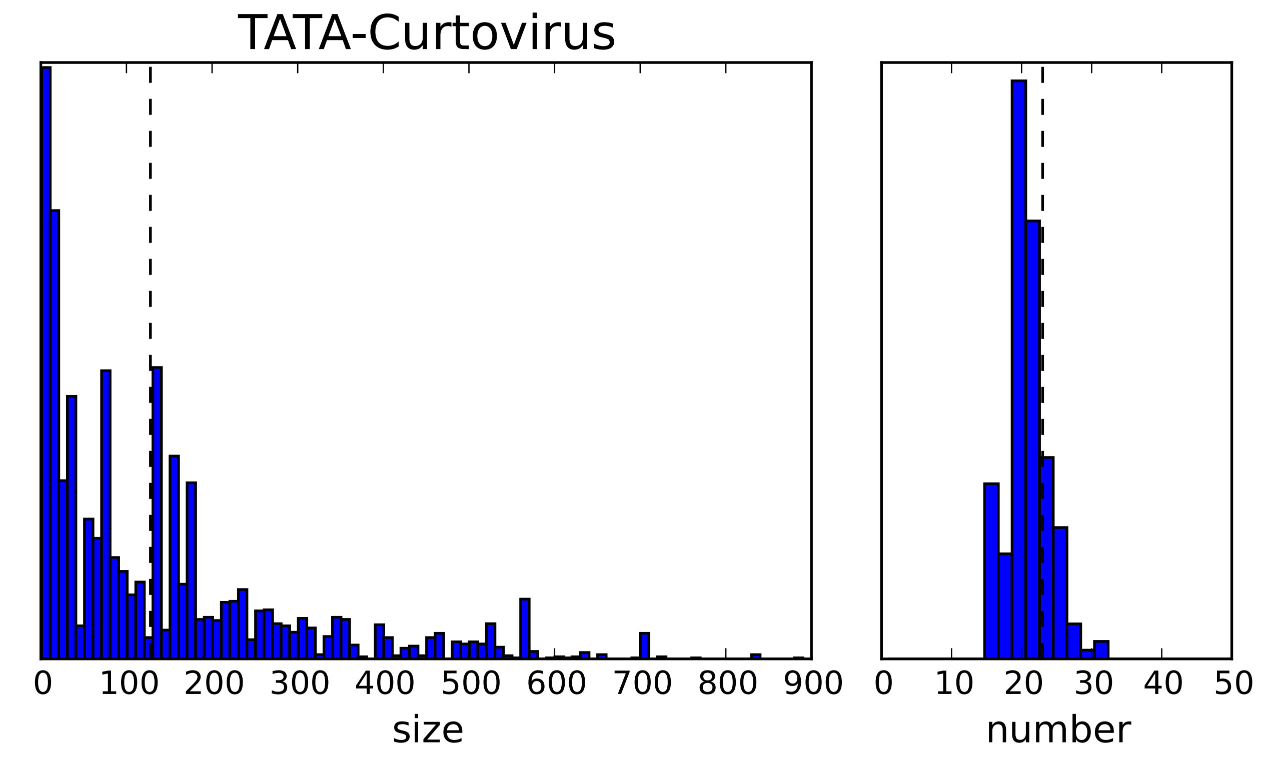
TATA-Curtovirus
0
100
200
300
400
500
600
700
800
900
size
0
10
20
30
40
50
number

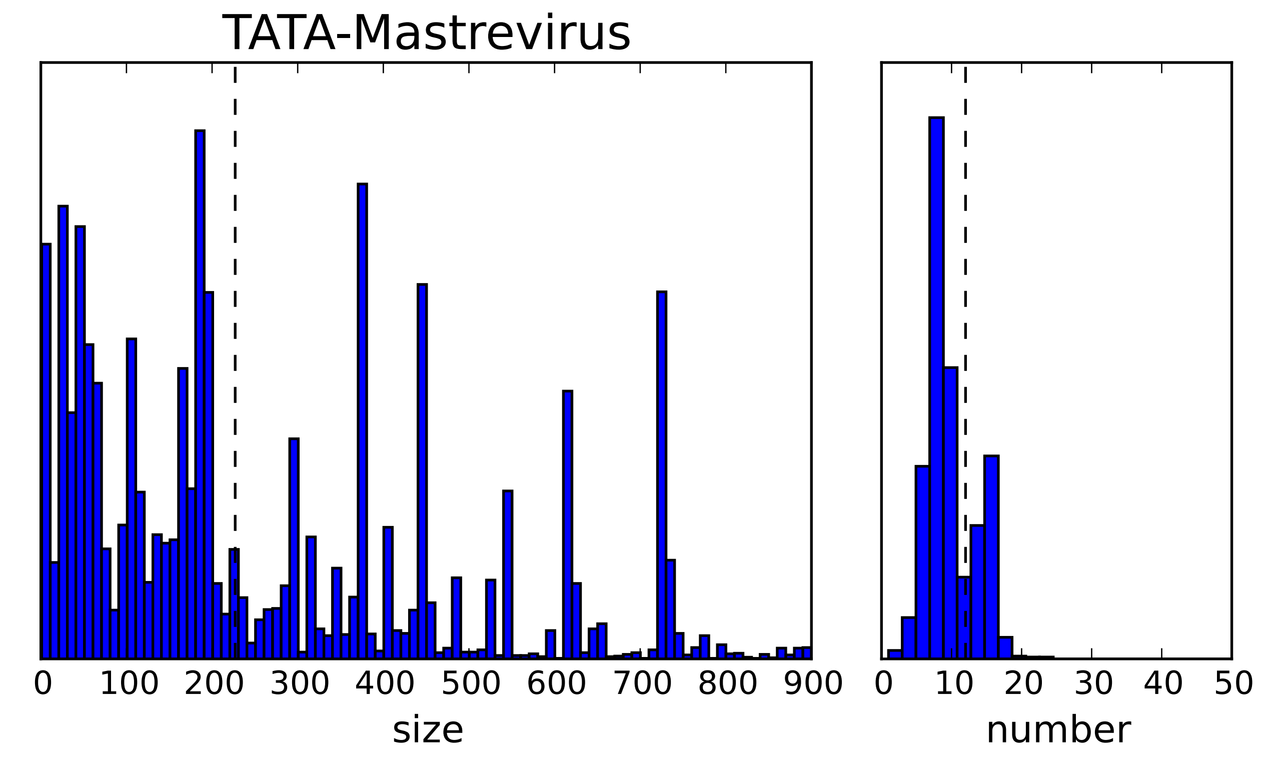
TATA-Mastrevirus
0
100
200
300
400
500
600
700
800
900
size
0
10
20
30
40
50
number

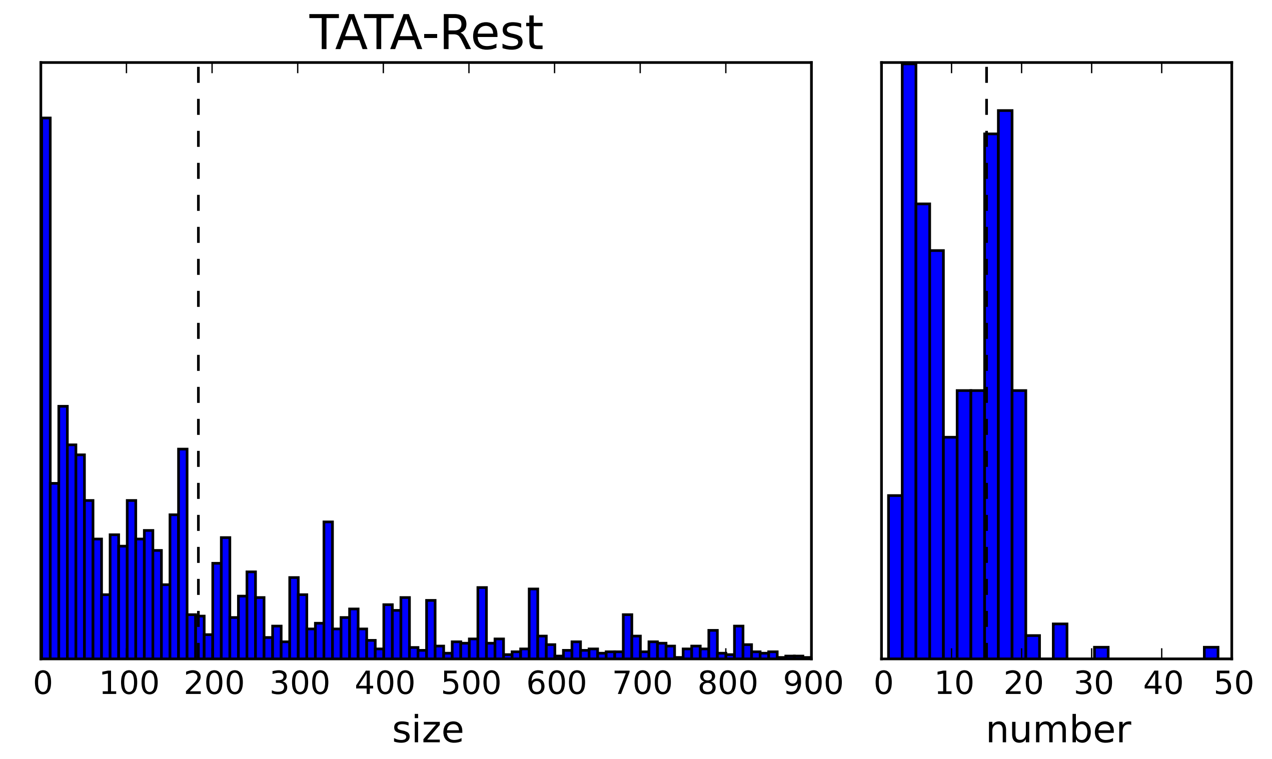
TATA-Rest
0
100
200
300
400
500
600
700
800
900
size
0
10
20
30
40
50
number

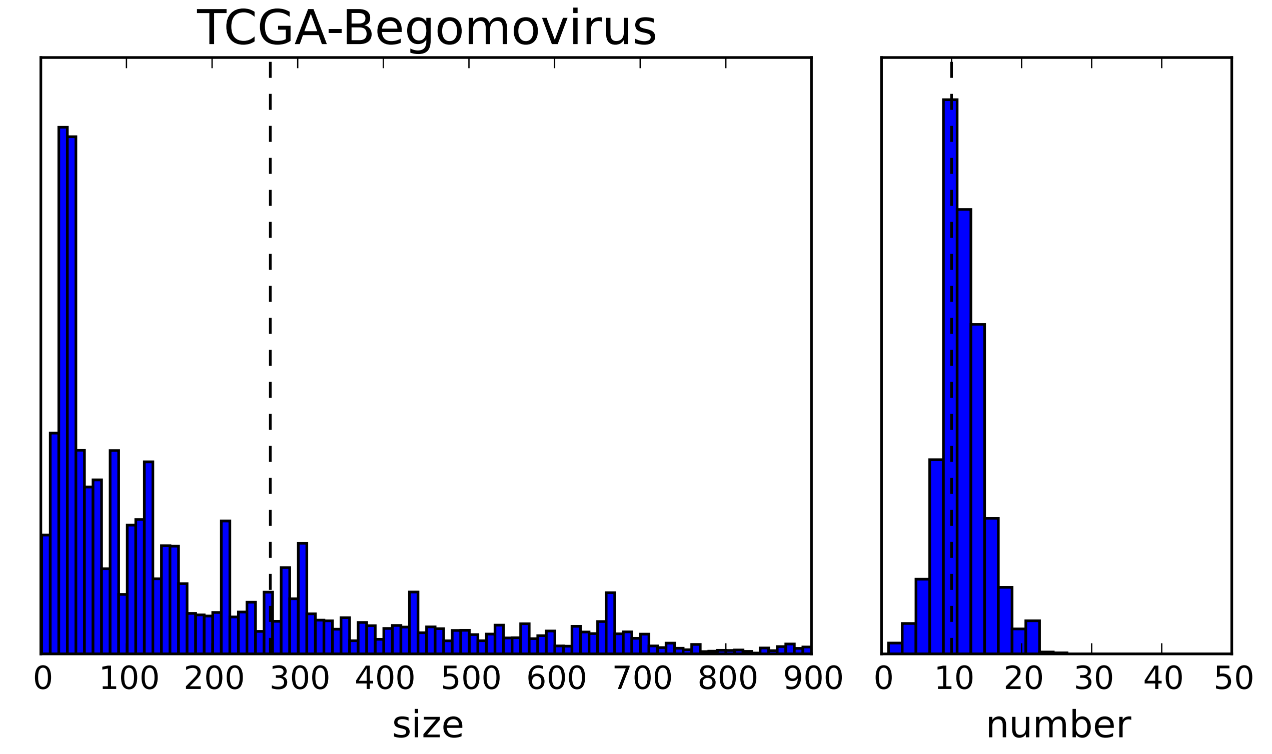
TCGA-Begomovirus
0
100
200
300
400
500
600
700
800
900
size
0
10
20
30
40
50
number

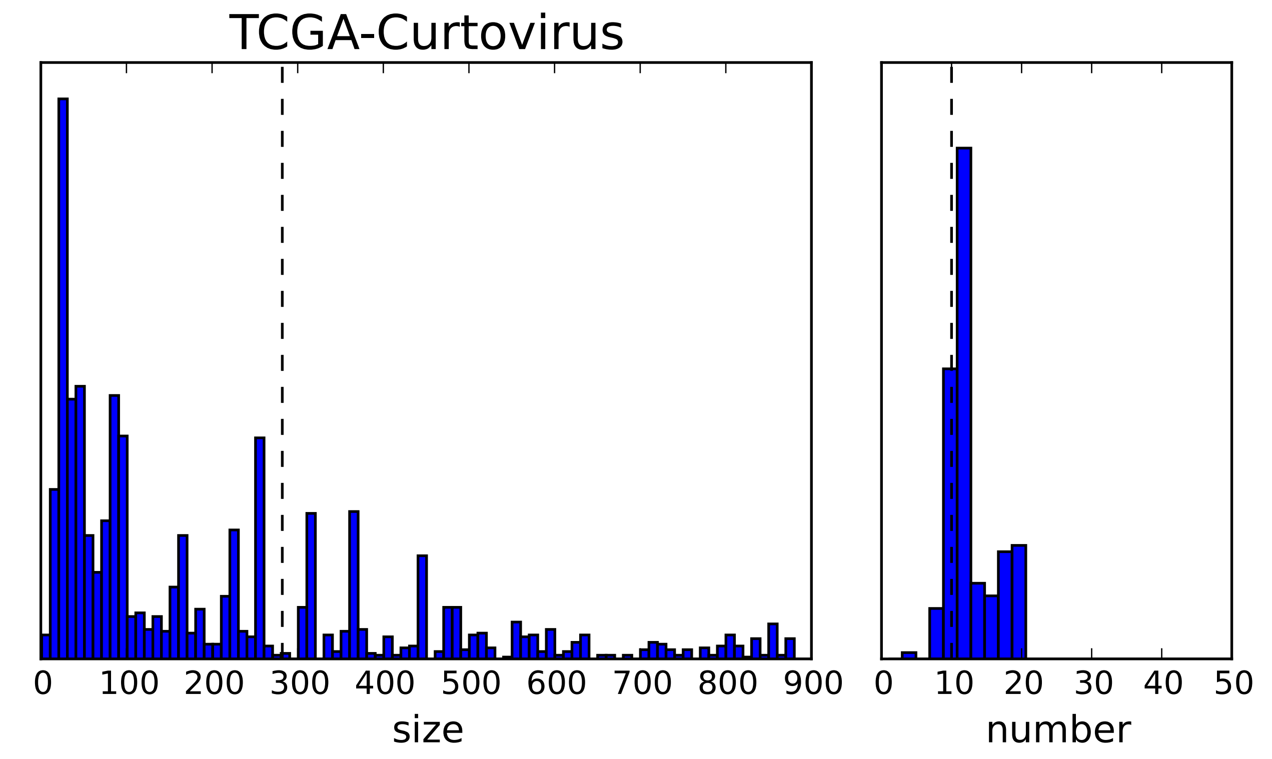
TCGA-Curtovirus
0
100
200
300
400
500
600
700
800
900
size
0
10
20
30
40
50
number

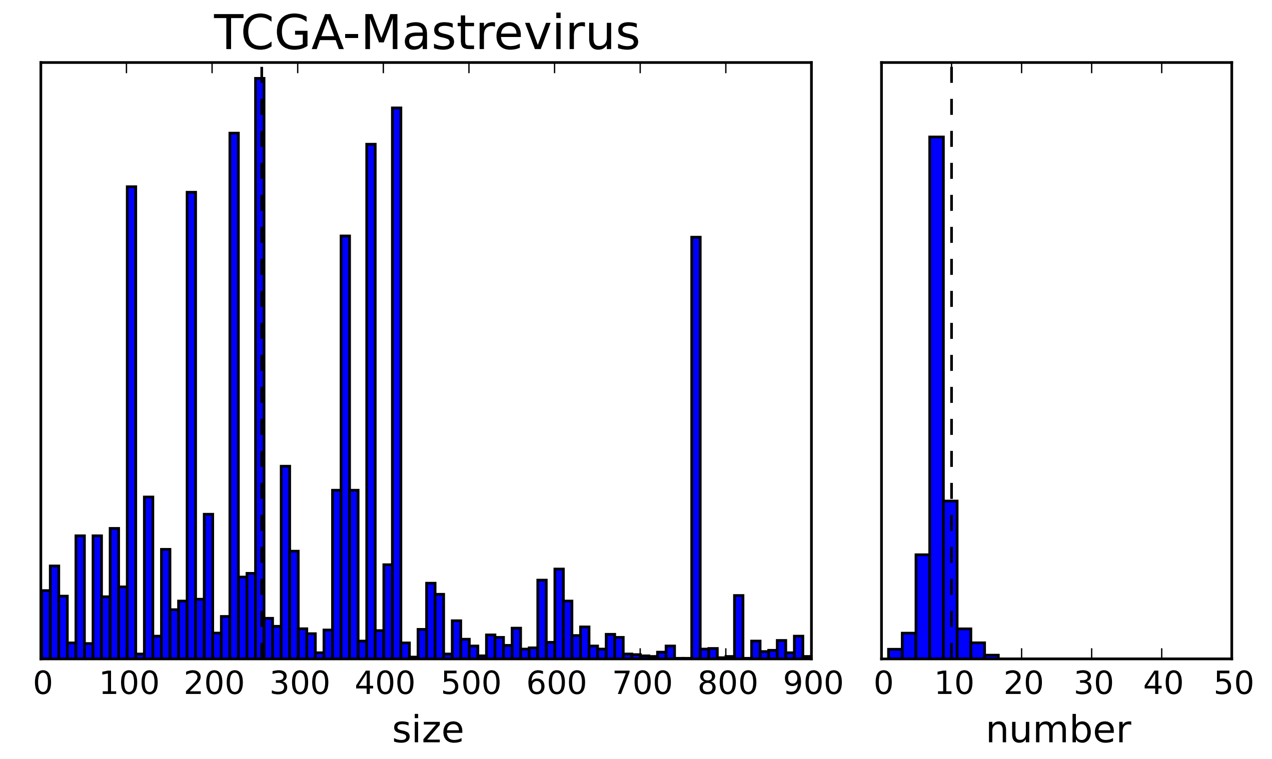
TCGA-Mastrevirus
0
100
200
300
400
500
600
700
800
900
size
0
10
20
30
40
50
number

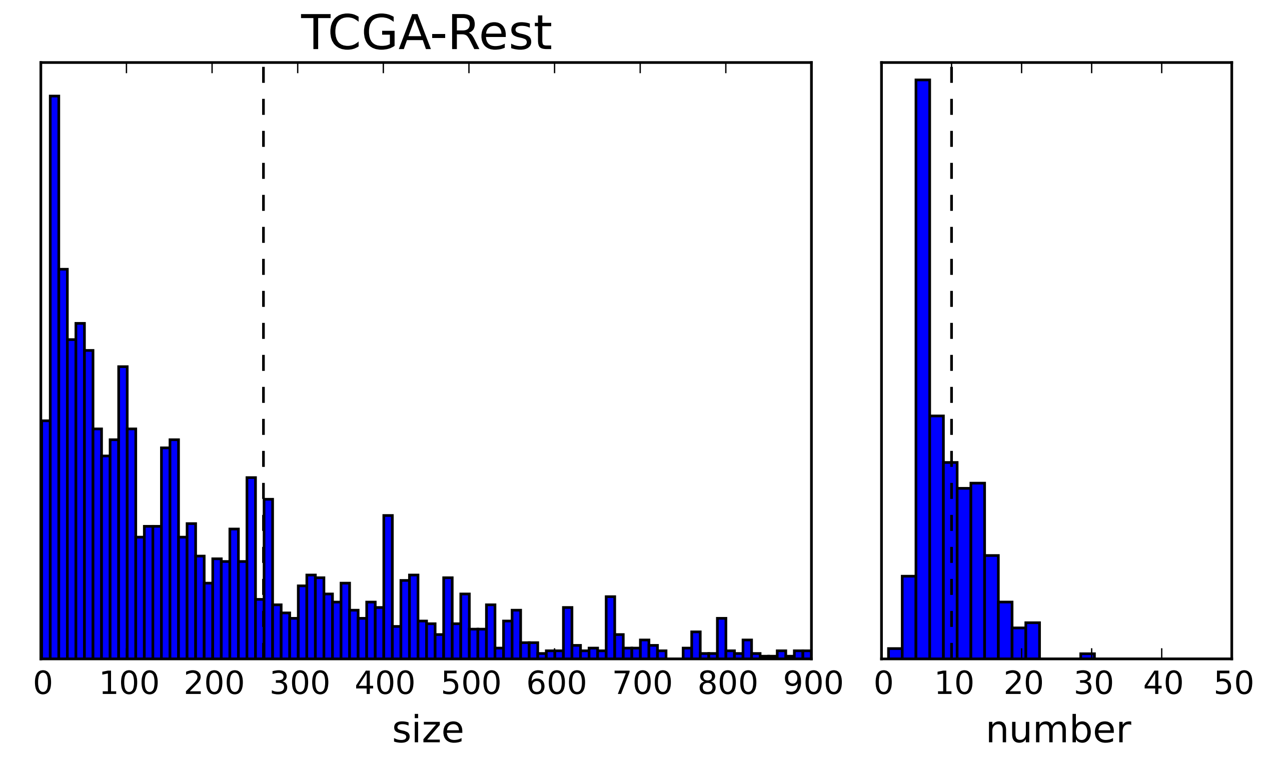
TCGA-Rest
0
100
200
300
400
500
600
700
800
900
size
0
10
20
30
40
50
number

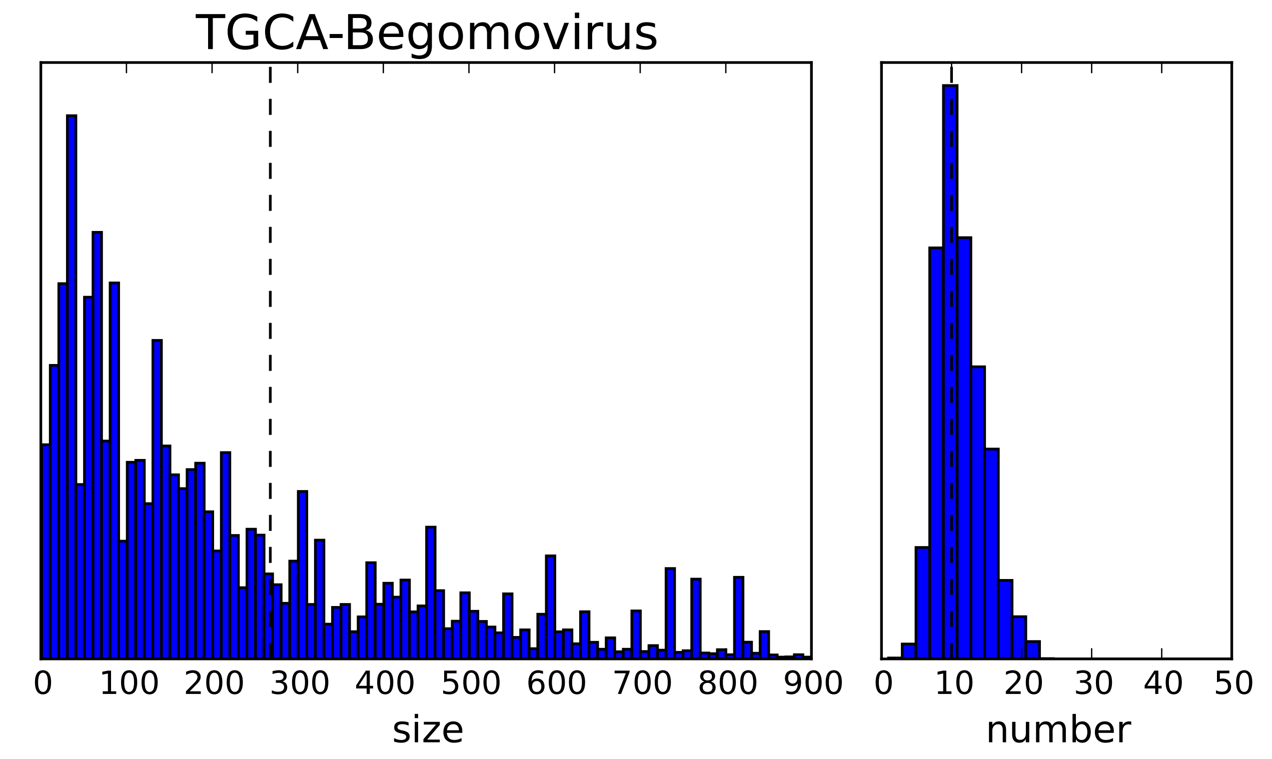

TGCA-Begomovirus
0
100
200
300
400
500
600
700
800
900
size
0
10
20
30
40
50
number

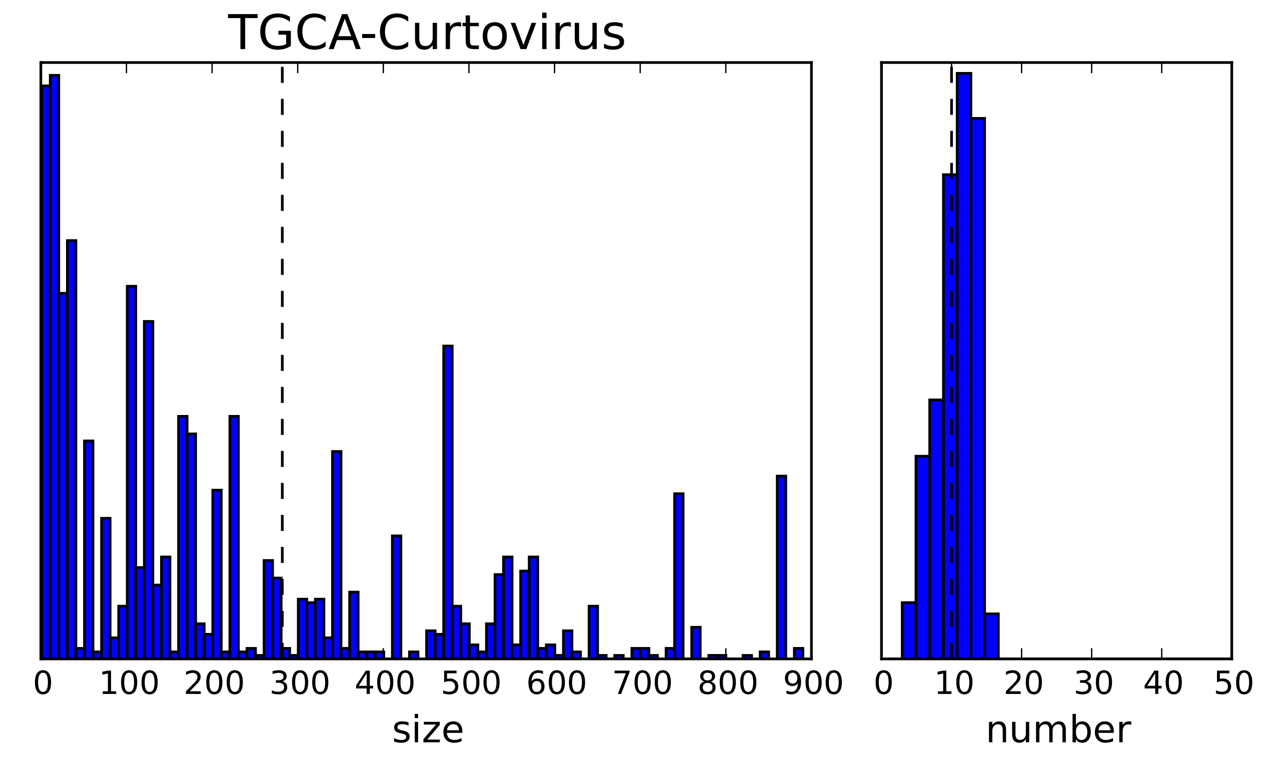
TGCA-Curtovirus
0
100
200
300
400
500
600
700
800
900
size
0
10
20
30
40
50
number

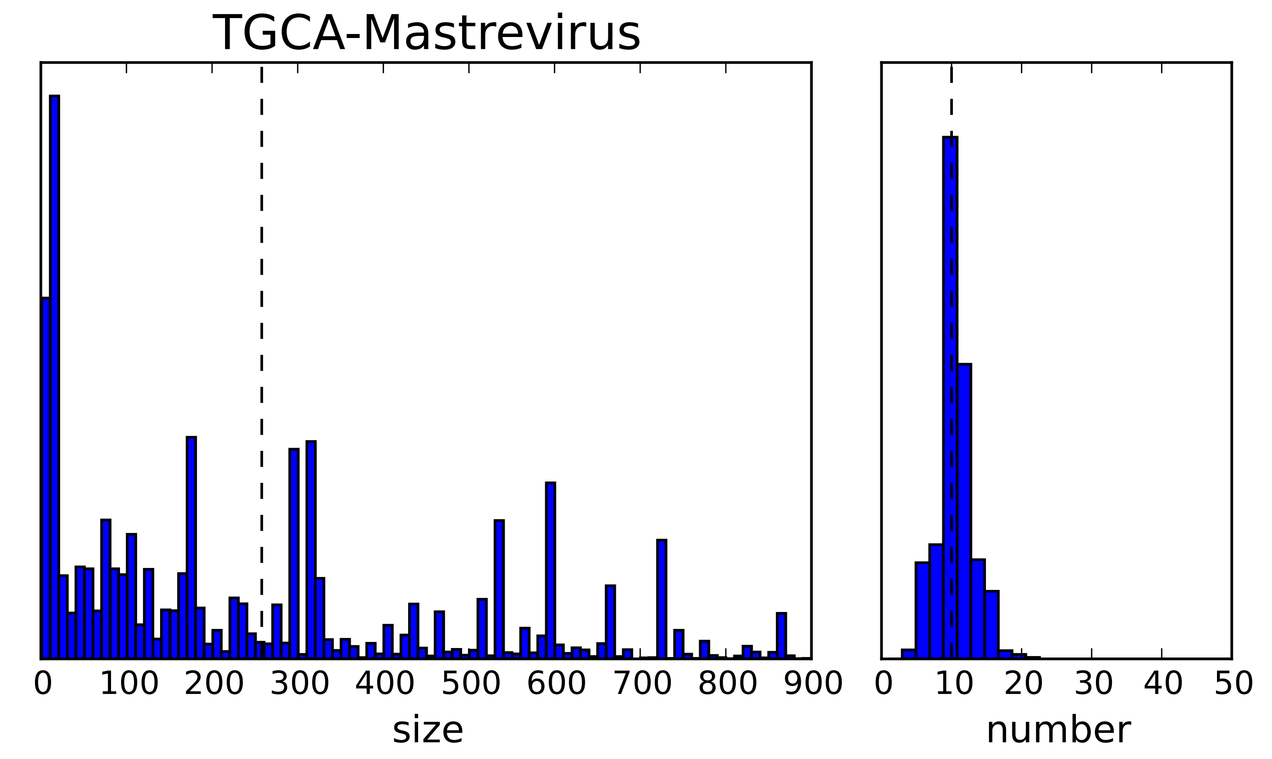
TGCA-Mastrevirus
0
100
200
300
400
500
600
700
800
900
size
0
10
20
30
40
50
number

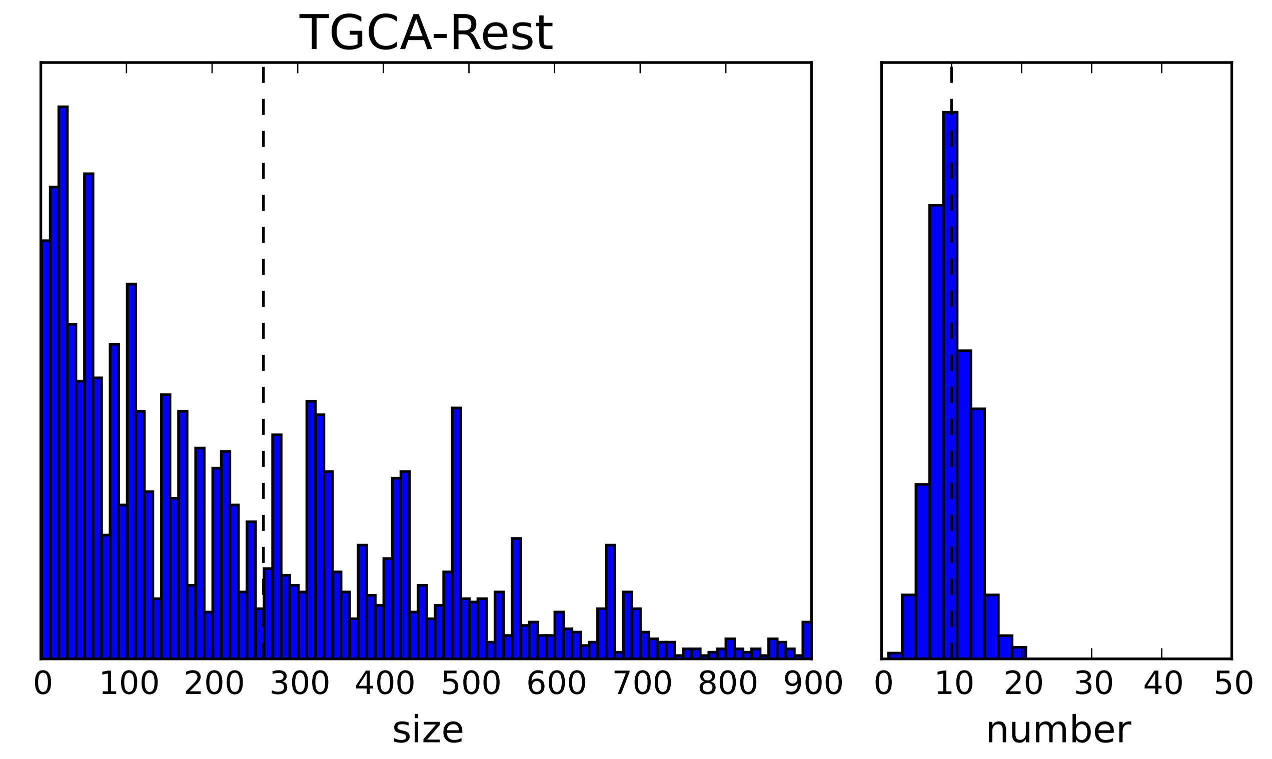
TGCA-Rest
0
100
200
300
400
500
600
700
800
900
size
0
10
20
30
40
50
number

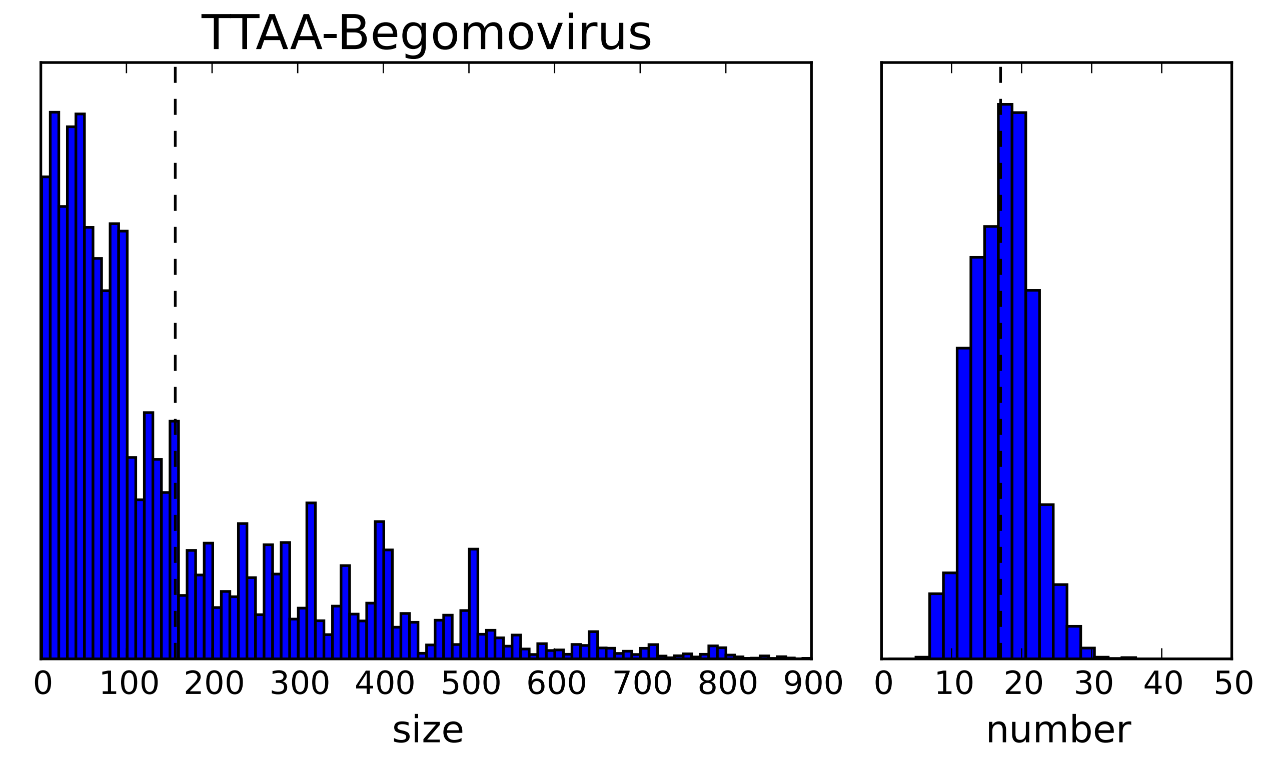

TTAA-Begomovirus
0
100
200
300
400
500
600
700
800
900
size
0
10
20
30
40
50
number

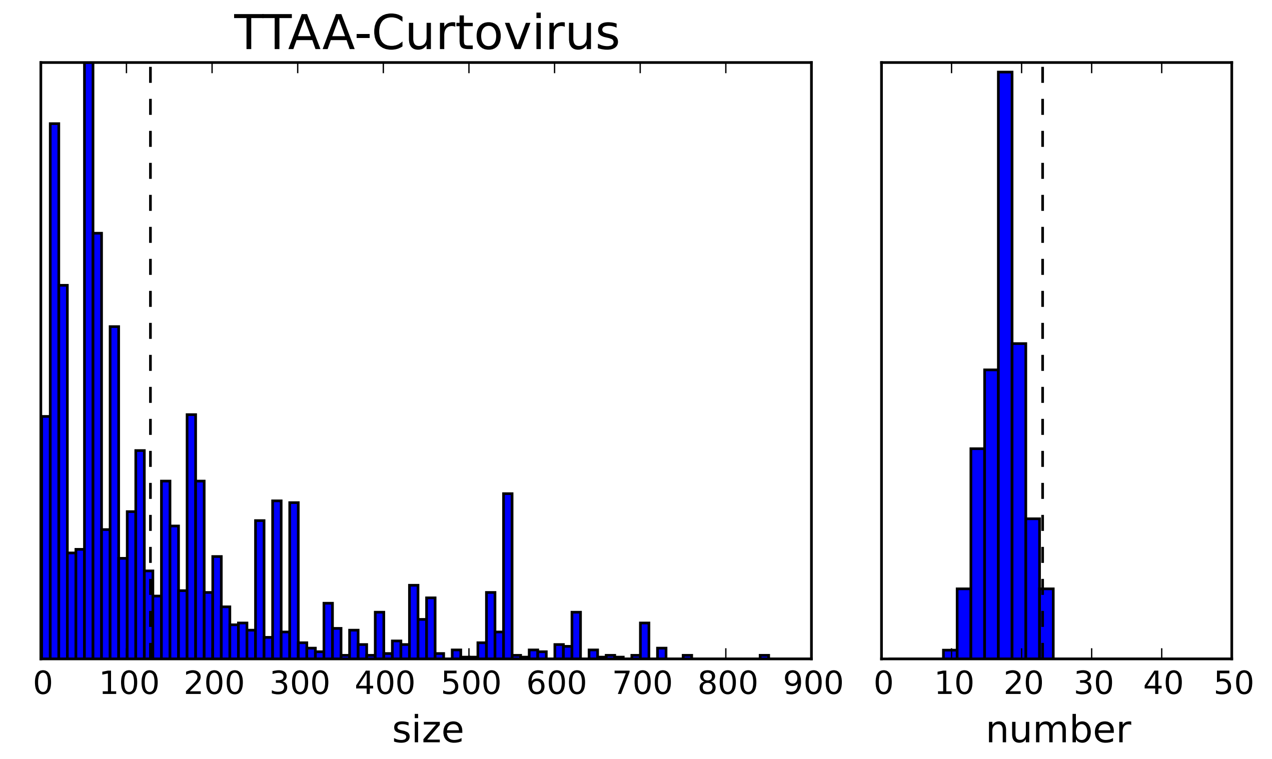
TTAA-Curtovirus
0
100
200
300
400
500
600
700
800
900
size
0
10
20
30
40
50
number

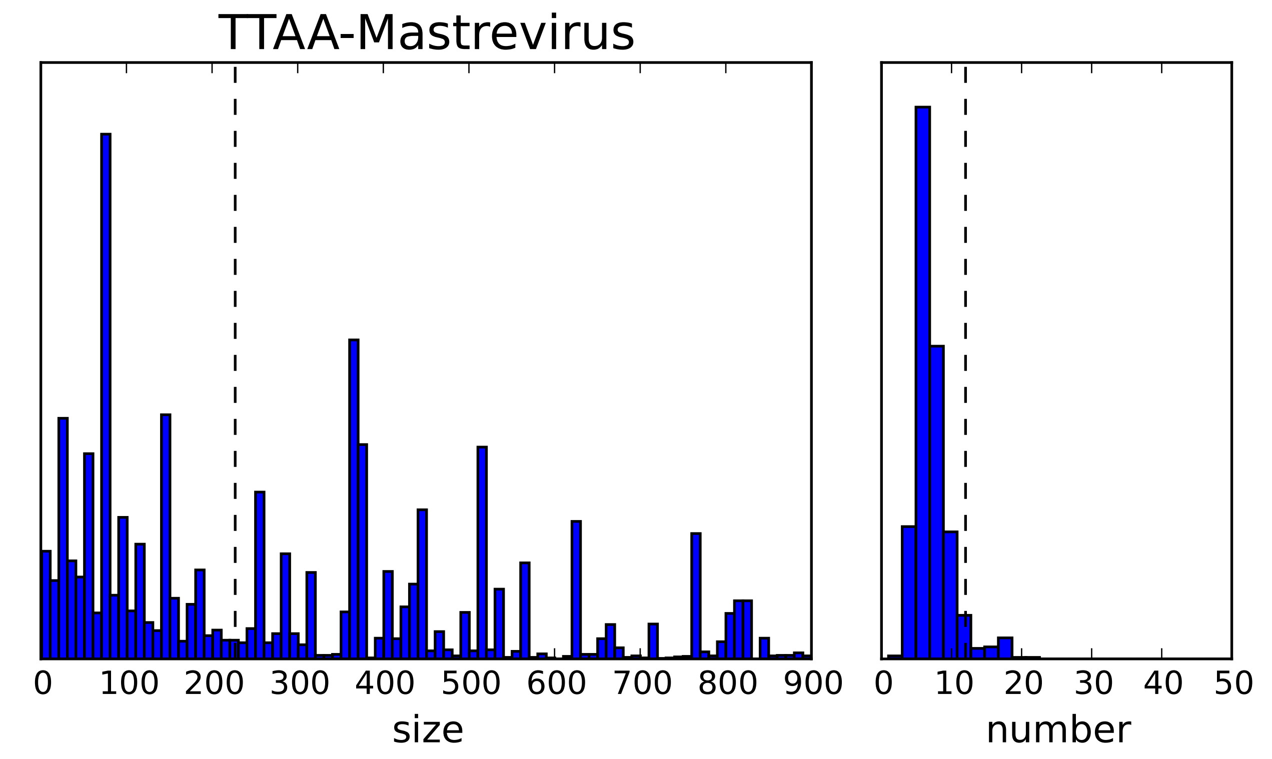
TTAA-Mastrevirus
0
100
200
300
400
500
600
700
800
900
size
0
10
20
30
40
50
number

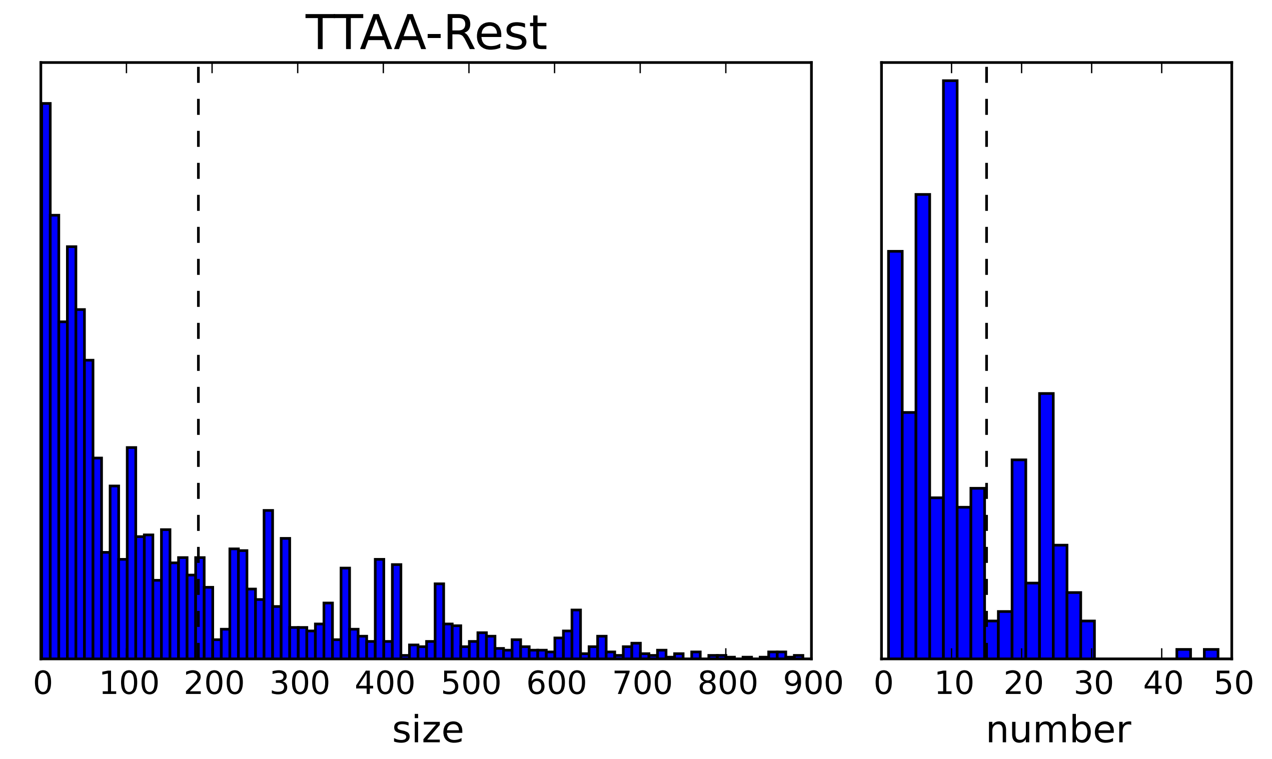
TTAA-Rest
0
100
200
300
400
500
600
700
800
900
size
0
10
20
30
40
50
number

REn Statistics DB1705

	# entries
Begomovirus	7003
Curtovirus	195
Mastrevirus	1621
Rest	329

REn	Genus	mean length	exp fragment size	exp fragment number
AATT	Begomovirus	2718	157	17
AATT	Curtovirus	2902	128	23
AATT	Mastrevirus	2681	227	12
AATT	Rest	2691	184	15
ACGT	Begomovirus	2718	268	10
ACGT	Curtovirus	2902	282	10
ACGT	Mastrevirus	2681	258	10
ACGT	Rest	2691	260	10
AGCT	Begomovirus	2718	268	10
AGCT	Curtovirus	2902	282	10
AGCT	Mastrevirus	2681	258	10
AGCT	Rest	2691	260	10
CATG	Begomovirus	2718	268	10
CATG	Curtovirus	2902	282	10
CATG	Mastrevirus	2681	258	10
CATG	Rest	2691	260	10
CCGC	Begomovirus	2718	523	5
CCGC	Curtovirus	2902	830	3
CCGC	Mastrevirus	2681	335	8
CCGC	Rest	2691	390	7
CCGG	Begomovirus	2718	457	6
CCGG	Curtovirus	2902	624	5
CCGG	Mastrevirus	2681	293	9
CCGG	Rest	2691	369	7
CGCG	Begomovirus	2718	457	6
CGCG	Curtovirus	2902	624	5
CGCG	Mastrevirus	2681	293	9
CGCG	Rest	2691	369	7
CTAG	Begomovirus	2718	268	10
CTAG	Curtovirus	2902	282	10
CTAG	Mastrevirus	2681	258	10
CTAG	Rest	2691	260	10
GATC	Begomovirus	2718	268	10
GATC	Curtovirus	2902	282	10
GATC	Mastrevirus	2681	258	10
GATC	Rest	2691	260	10
GCGC	Begomovirus	2718	457	6
GCGC	Curtovirus	2902	624	5
GCGC	Mastrevirus	2681	293	9
GCGC	Rest	2691	369	7
GGCC	Begomovirus	2718	457	6

GGCC	Curtovirus	2902	624	5
GGCC	Mastrevirus	2681	293	9
GGCC	Rest	2691	369	7
GTAC	Begomovirus	2718	268	10
GTAC	Curtovirus	2902	282	10
GTAC	Mastrevirus	2681	258	10
GTAC	Rest	2691	260	10
TATA	Begomovirus	2718	157	17
TATA	Curtovirus	2902	128	23
TATA	Mastrevirus	2681	227	12
TATA	Rest	2691	184	15
TCGA	Begomovirus	2718	268	10
TCGA	Curtovirus	2902	282	10
TCGA	Mastrevirus	2681	258	10
TCGA	Rest	2691	260	10
TGCA	Begomovirus	2718	268	10
TGCA	Curtovirus	2902	282	10
TGCA	Mastrevirus	2681	258	10
TGCA	Rest	2691	260	10
TTAA	Begomovirus	2718	157	17
TTAA	Curtovirus	2902	128	23
TTAA	Mastrevirus	2681	227	12
TTAA	Rest	2691	184	15
