# Supplementary material for: Barcoding of Plant Viruses with Circular Single-Stranded DNA Based on Rolling Circle Amplification
Source: Viruses. 2018 Aug 31;10(9):469. doi: 10.3390/v10090469 (PMC6164888; doi:10.3390/v10090469)
Supplement: Supplementary file 1 [file viruses-10-00469-s001.zip › 3-viruses-345336-supplymentary/Jeske18R_Figures S2.pdf]

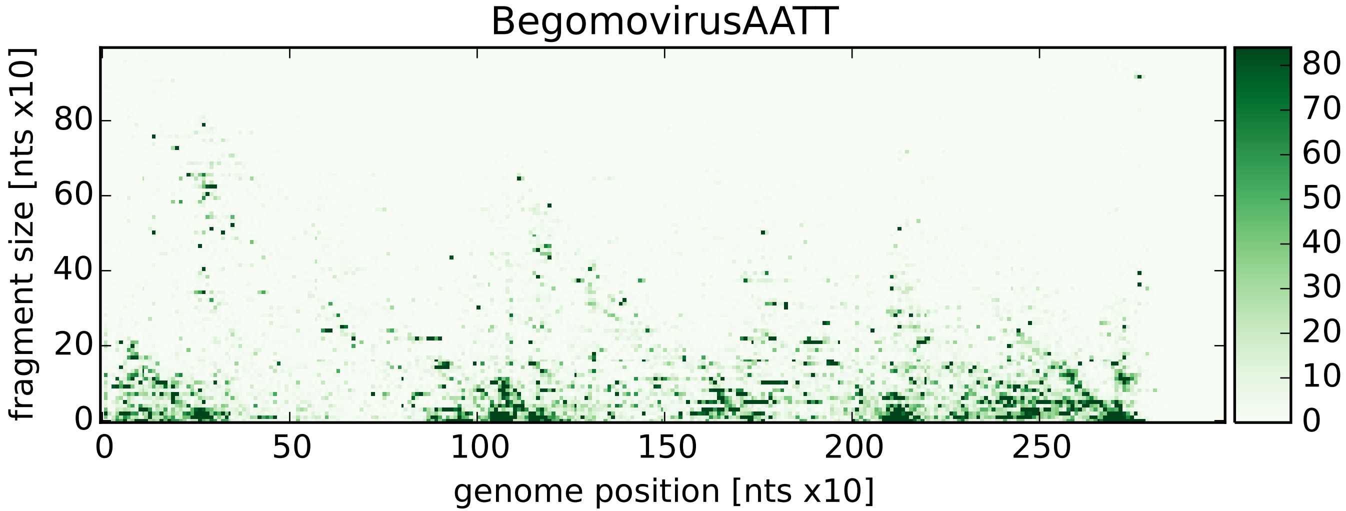
BegomovirusAATT
fragment size [nts x10]
genome position [nts x10]
0
20
40
60
80
0
50
100
150
200
250
0
10
20
30
40
50
60
70
80

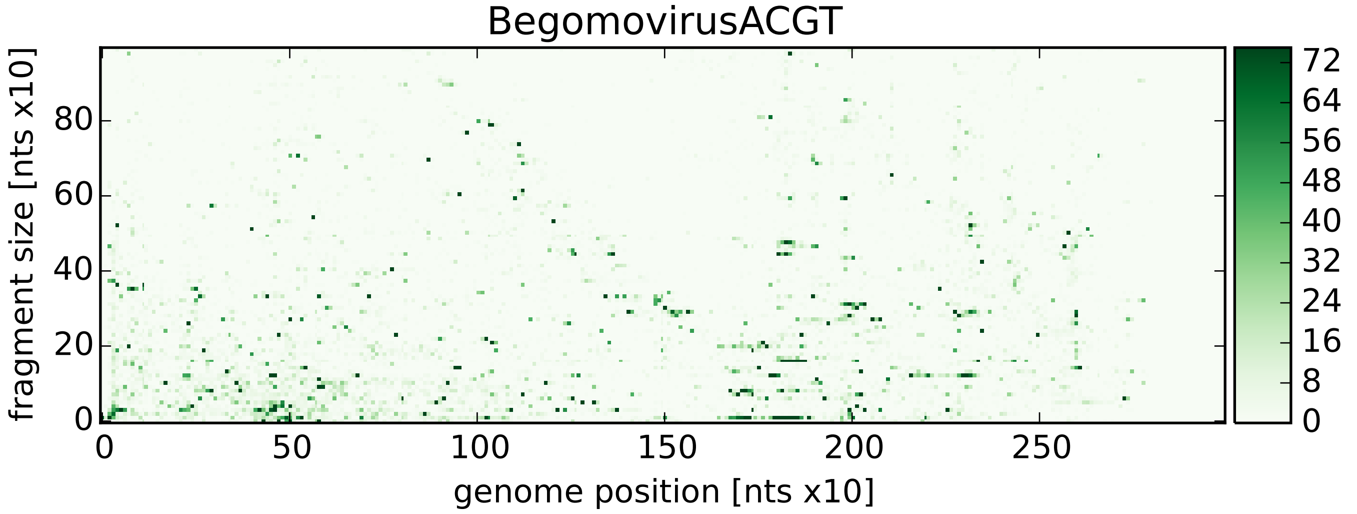

BegomovirusACGT
fragment size [nts x10]
0
20
40
60
80
genome position [nts x10]
0
50
100
150
200
250
72
64
56
48
40
32
24
16
8
0

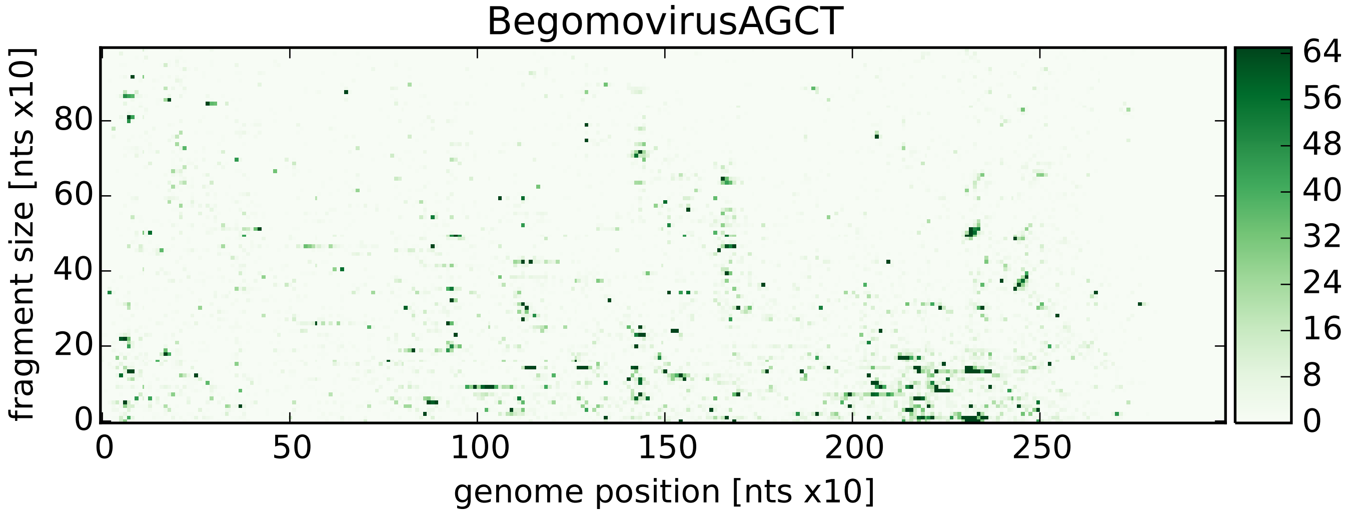

BegomovirusAGCT
fragment size [nts x10]
genome position [nts x10]
0
20
40
60
80
0
50
100
150
200
250
64
56
48
40
32
24
16
8
0

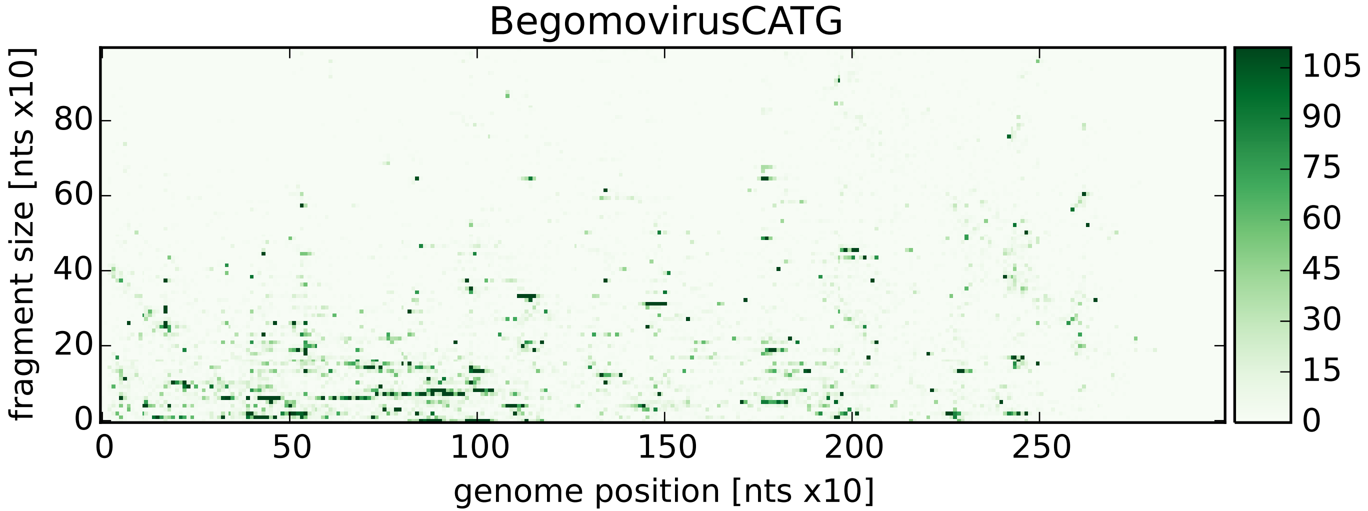
BegomovirusCATG
fragment size [nts x10]
genome position [nts x10]
0
20
40
60
80
0
50
100
150
200
250
0
15
30
45
60
75
90
105

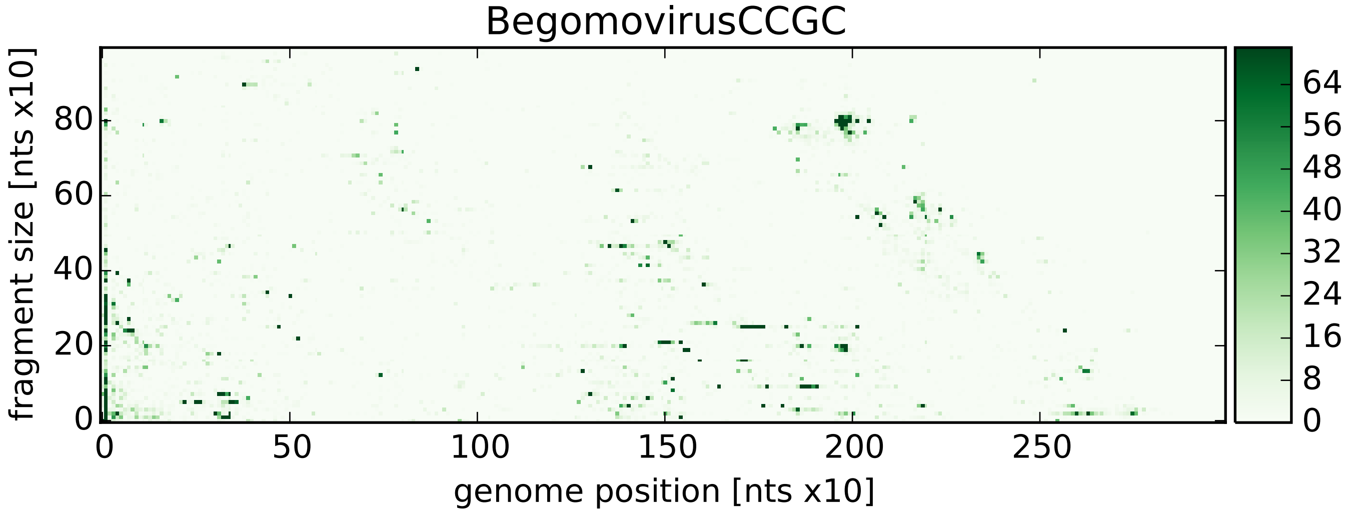

BegomovirusCCGC
fragment size [nts x10]
genome position [nts x10]
0
20
40
60
80
0
50
100
150
200
250
0
8
16
24
32
40
48
56
64

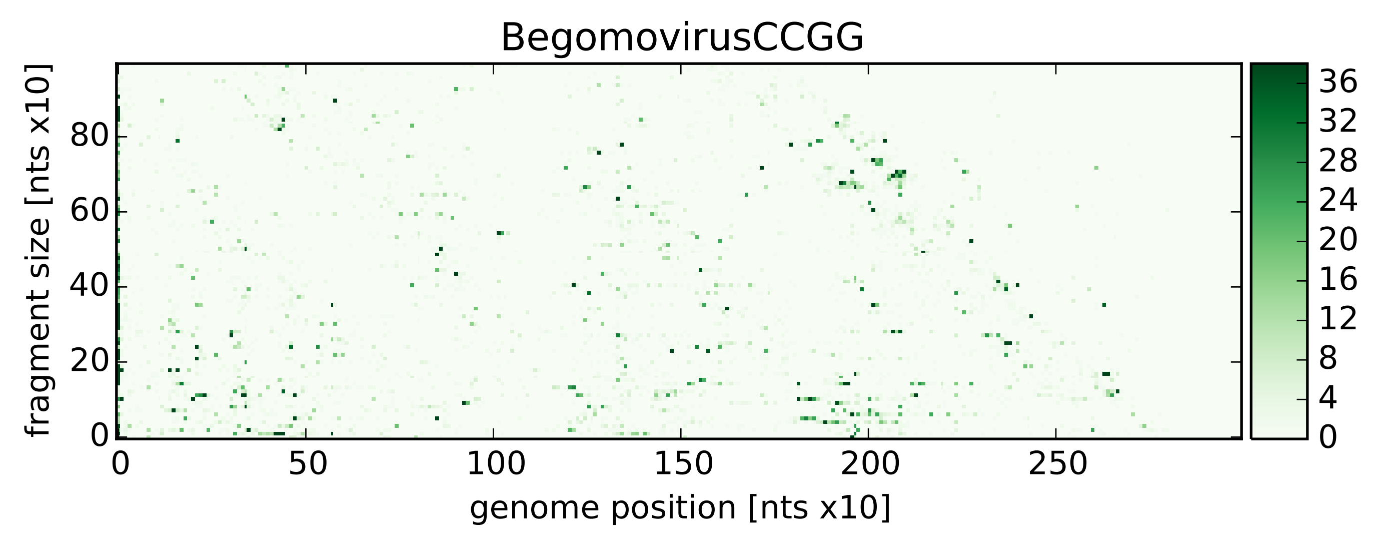
BegomovirusCCGG
fragment size [nts x10]
genome position [nts x10]
0
20
40
60
80
0
50
100
150
200
250
0
4
8
12
16
20
24
28
32
36

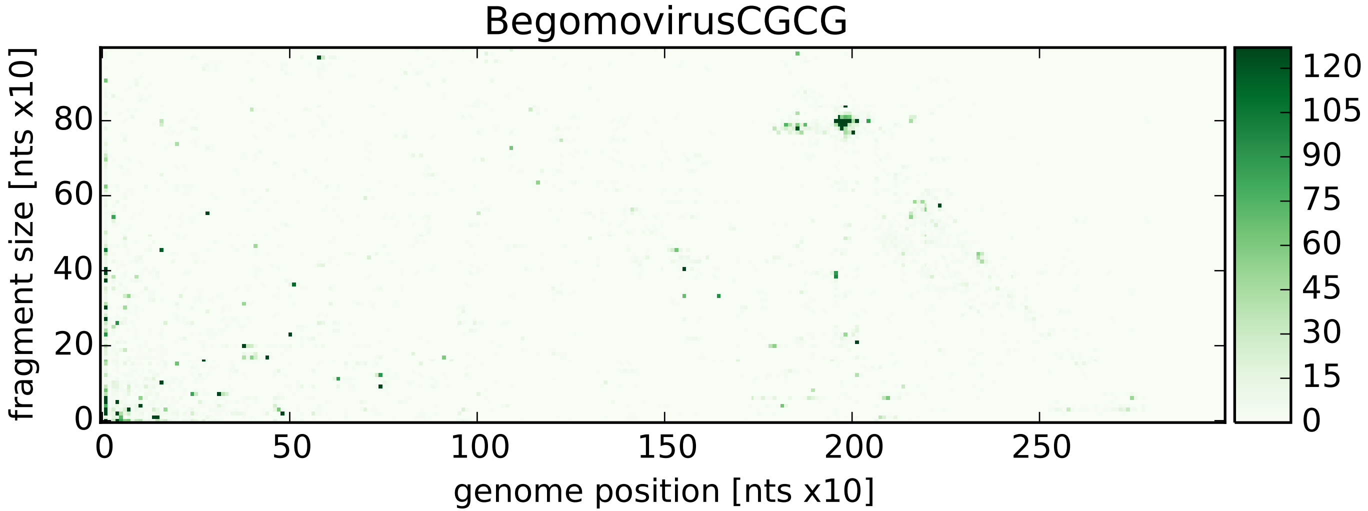

BegomovirusCGCG
fragment size [nts x10]
genome position [nts x10]
0
20
40
60
80
0
50
100
150
200
250
0
15
30
45
60
75
90
105
120

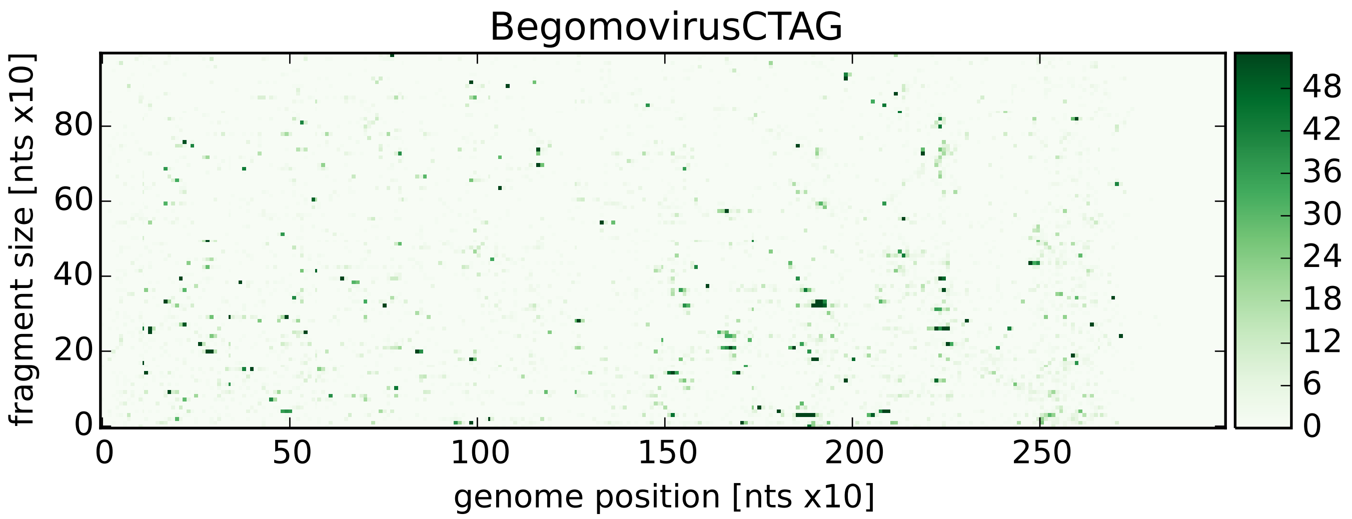
BegomovirusCTAG
fragment size [nts x10]
genome position [nts x10]
0
20
40
60
80
0
50
100
150
200
250
0
6
12
18
24
30
36
42
48

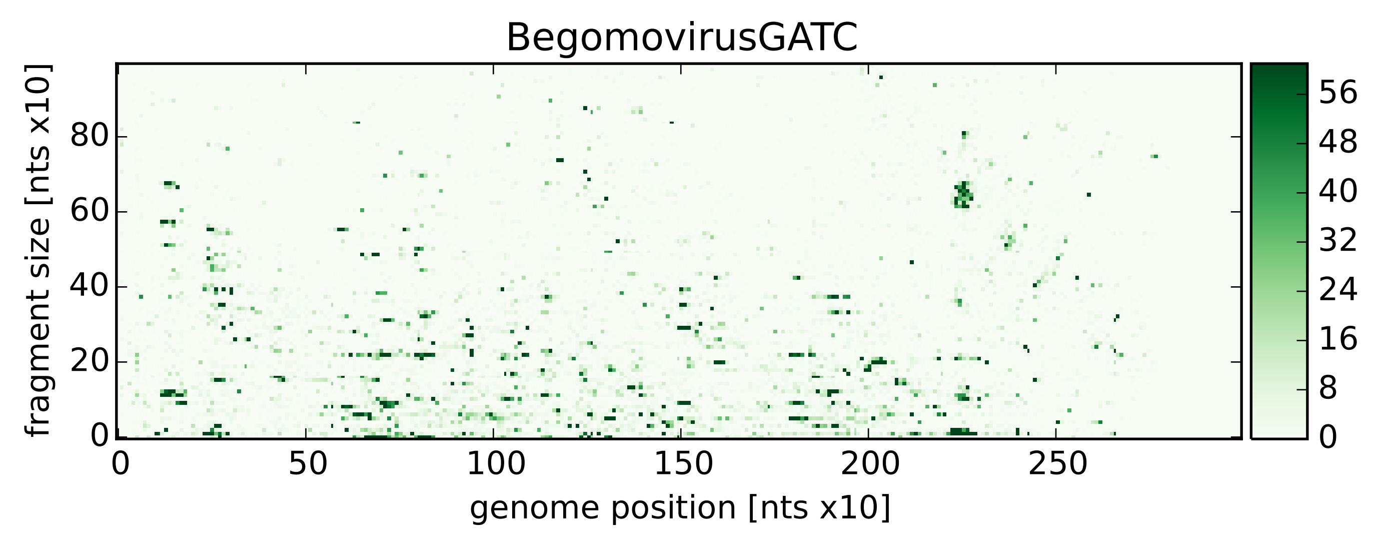
BegomovirusGATC
fragment size [nts x10]
genome position [nts x10]
0
20
40
60
80
0
50
100
150
200
250
0
8
16
24
32
40
48
56

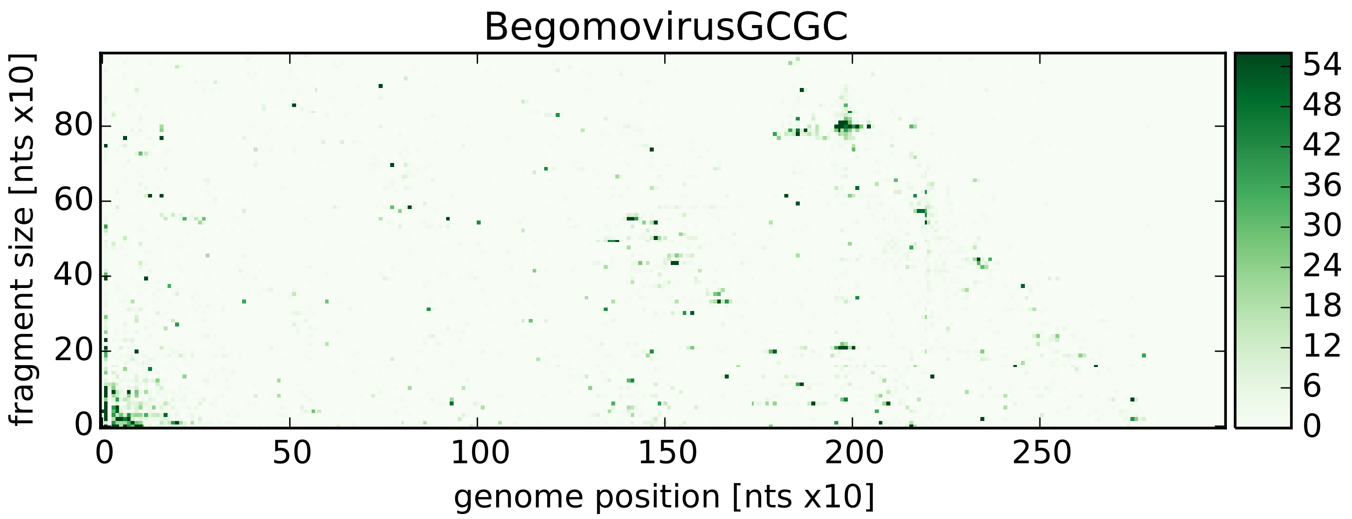
BegomovirusGCGC
fragment size [nts x10]
genome position [nts x10]
0
20
40
60
80
0
50
100
150
200
250
0
6
12
18
24
30
36
42
48
54

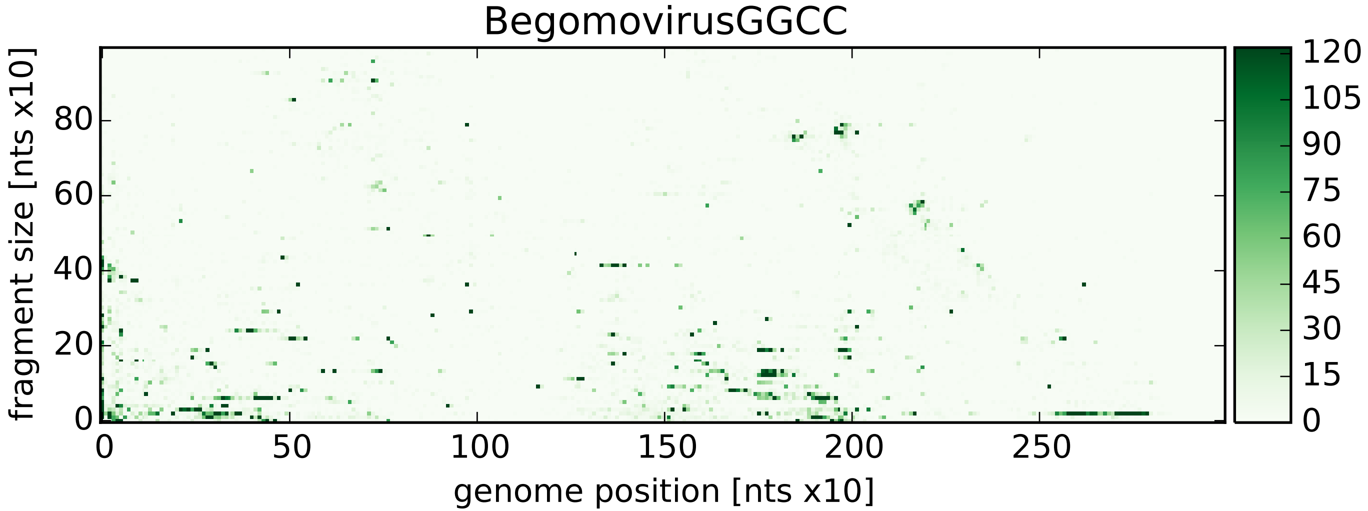
BegomovirusGGCC
fragment size [nts x10]
genome position [nts x10]
0
20
40
60
80
0
50
100
150
200
250
0
15
30
45
60
75
90
105
120

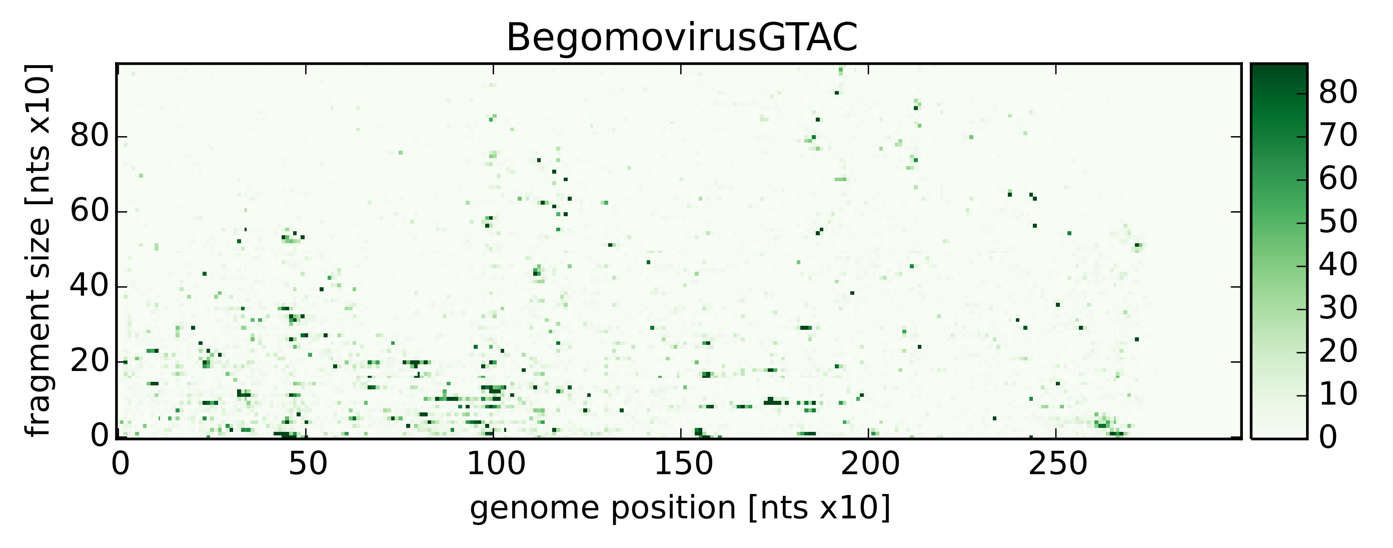

BegomovirusGTAC
fragment size [nts x10]
genome position [nts x10]
0
20
40
60
80
0
50
100
150
200
250
0
10
20
30
40
50
60
70
80

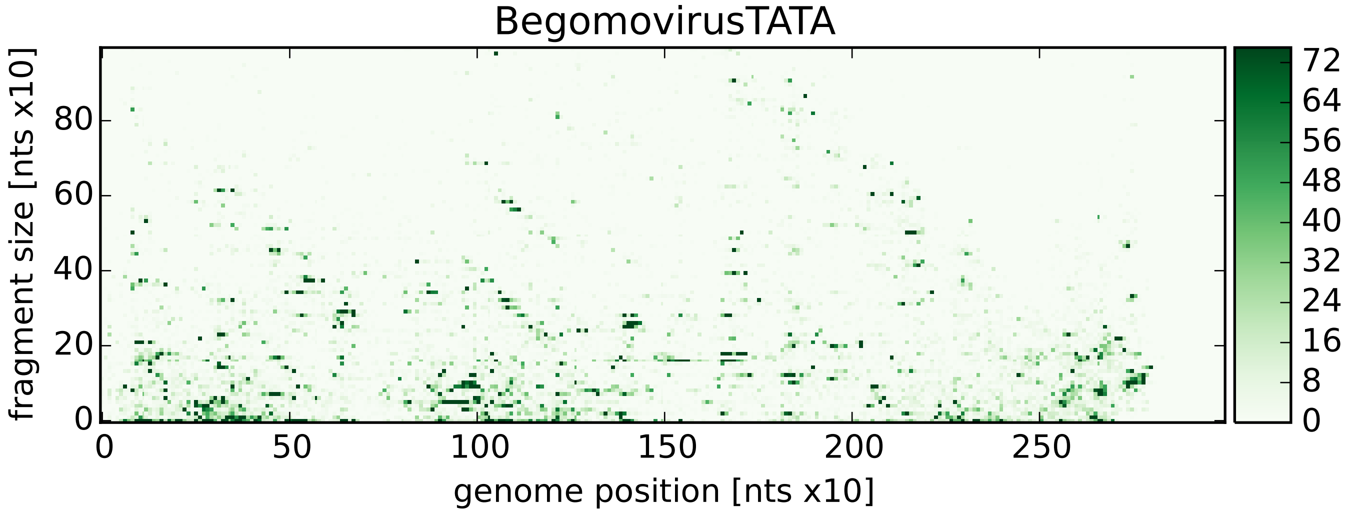

BegomovirusTATA
fragment size [nts x10]
genome position [nts x10]
0
20
40
60
80
50
100
150
200
250
8
16
24
32
40
48
56
64
72

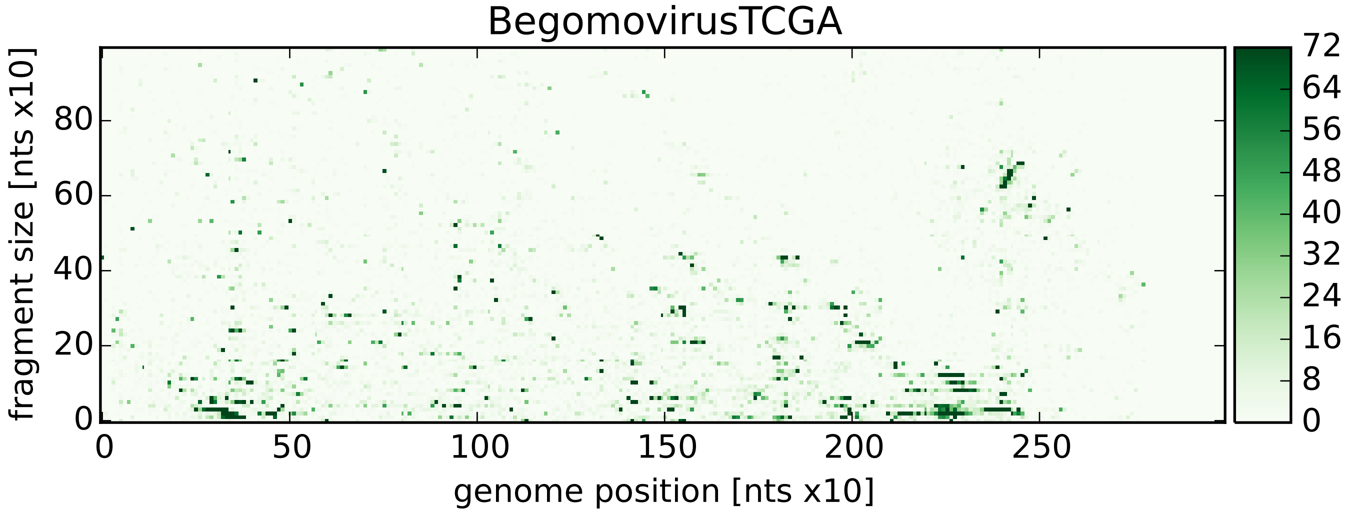

BegomovirusTCGA
fragment size [nts x10]
genome position [nts x10]
0
20
40
60
80
0
50
100
150
200
250
0
8
16
24
32
40
48
56
64
72

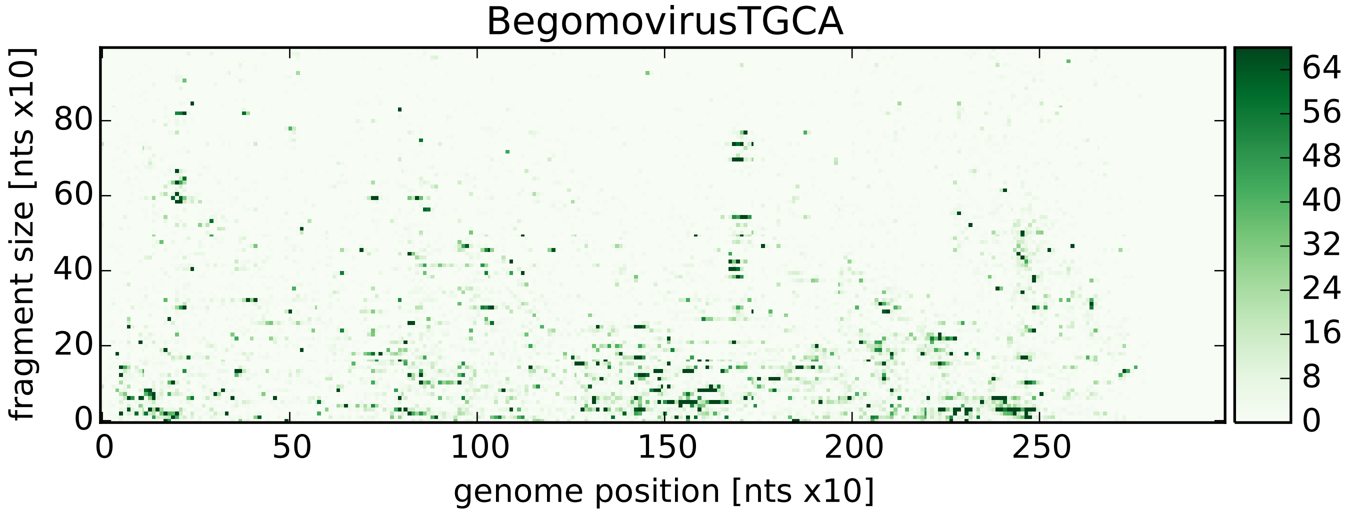

BegomovirusTGCA
fragment size [nts x10]
0
20
40
60
80
0
50
100
150
200
250
genome position [nts x10]
0
8
16
24
32
40
48
56
64

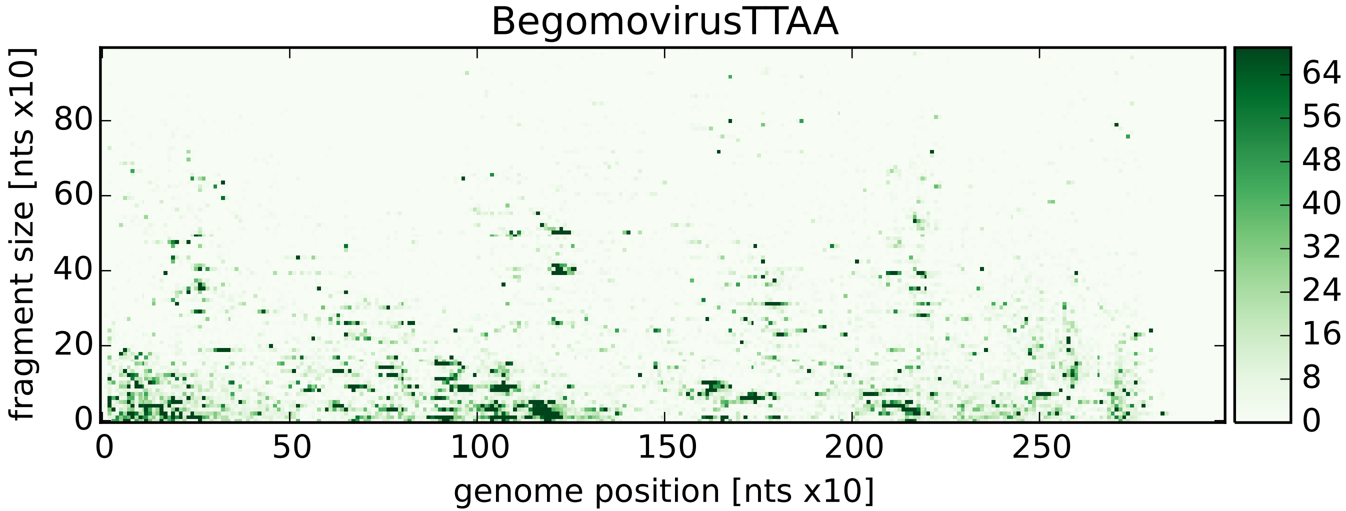

BegomovirusTTAA
fragment size [nts x10]
genome position [nts x10]
0
20
40
60
80
0
50
100
150
200
250
0
8
16
24
32
40
48
56
64

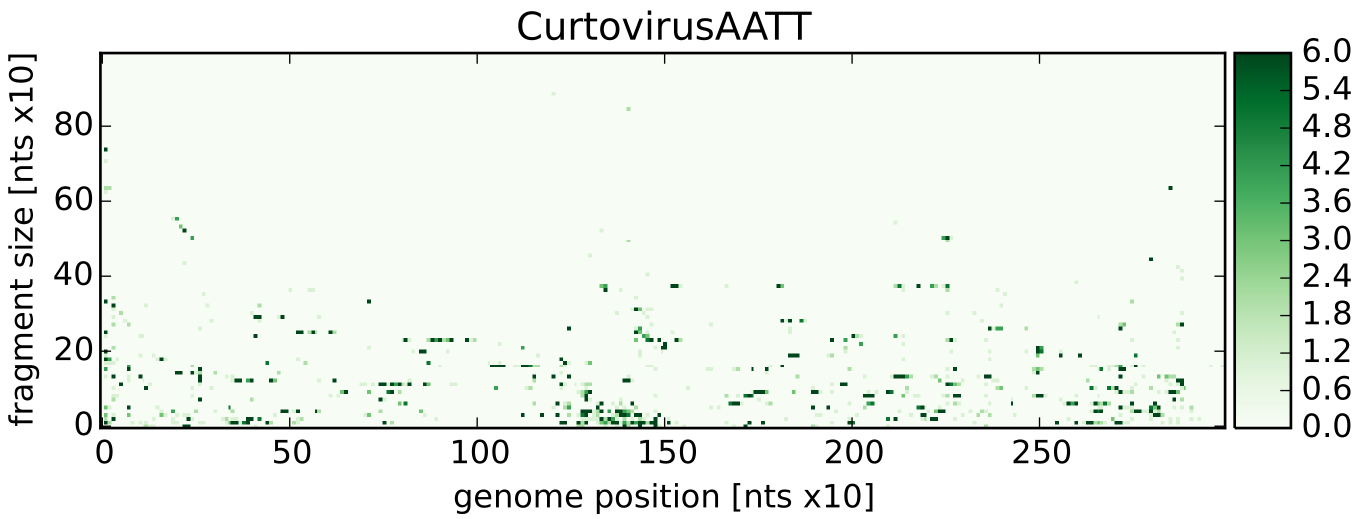

CurtovirusAATT
fragment size [nts x10]
genome position [nts x10]
0
20
40
60
80
0
50
100
150
200
250
0.0
0.6
1.2
1.8
2.4
3.0
3.6
4.2
4.8
5.4
6.0

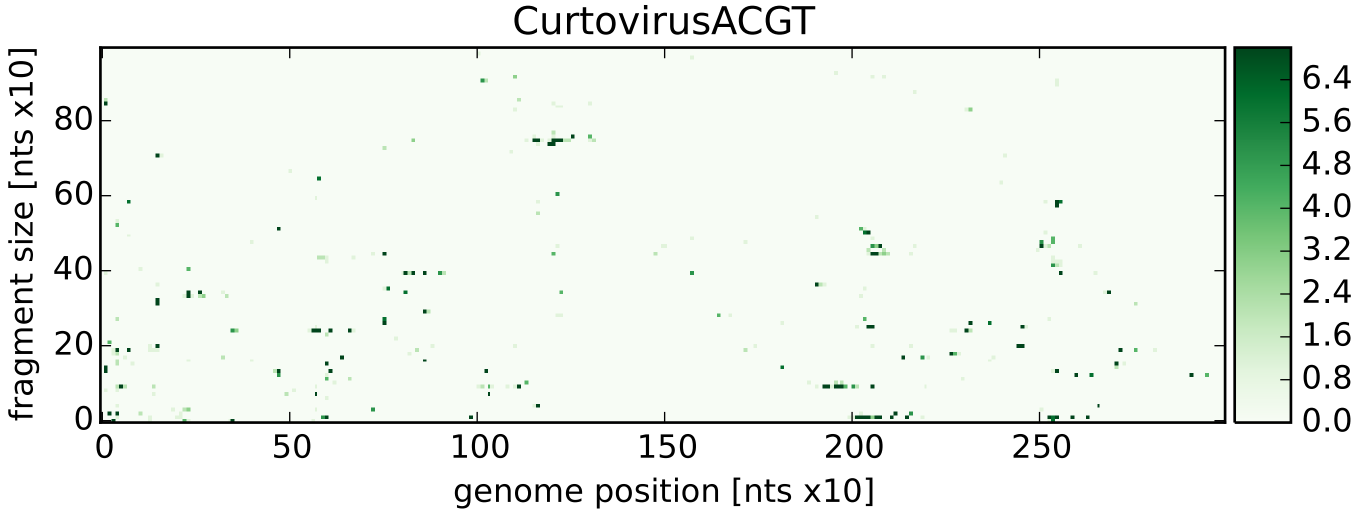
CurtovirusACGT
fragment size [nts x10]
genome position [nts x10]
0
20
40
60
80
0
50
100
150
200
250
0.0
0.8
1.6
2.4
3.2
4.0
4.8
5.6
6.4

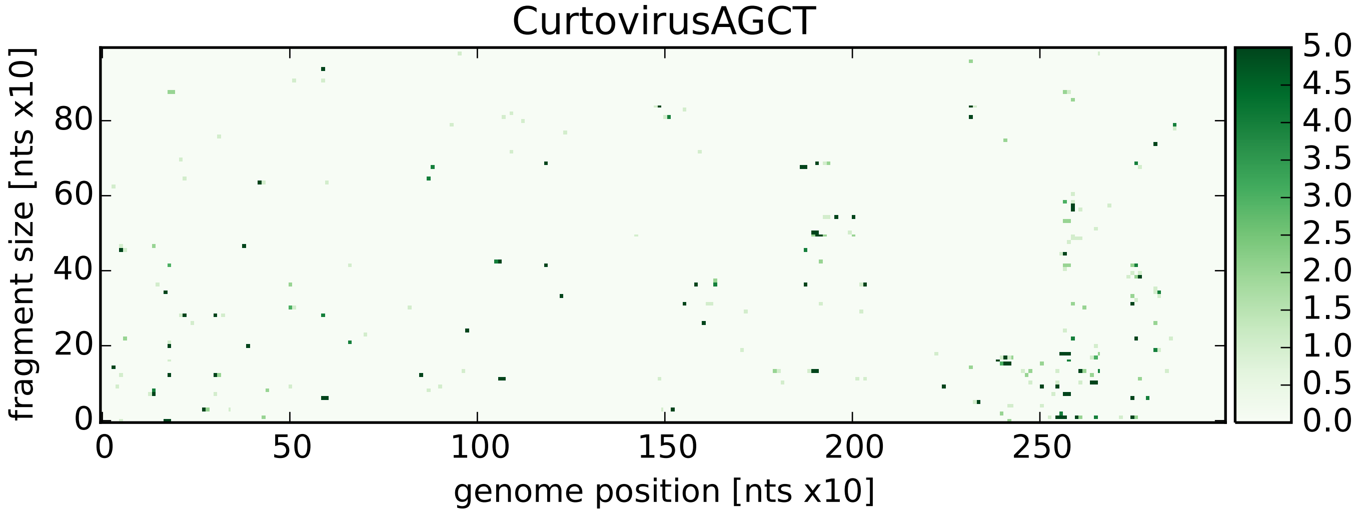

CurtovirusAGCT
fragment size [nts x10]
genome position [nts x10]
0
20
40
60
80
0
50
100
150
200
250
0.0
0.5
1.0
1.5
2.0
2.5
3.0
3.5
4.0
4.5
5.0

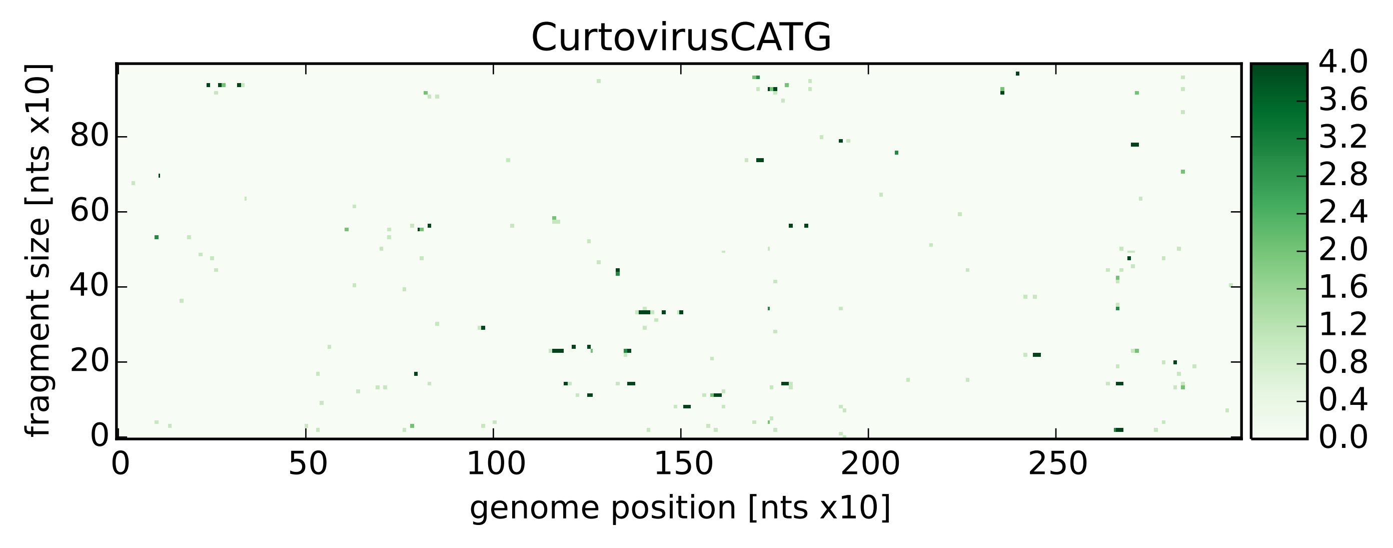

CurtovirusCATG
fragment size [nts x10]
genome position [nts x10]
0
20
40
60
80
0
50
100
150
200
250
0.0
0.4
0.8
1.2
1.6
2.0
2.4
2.8
3.2
3.6
4.0

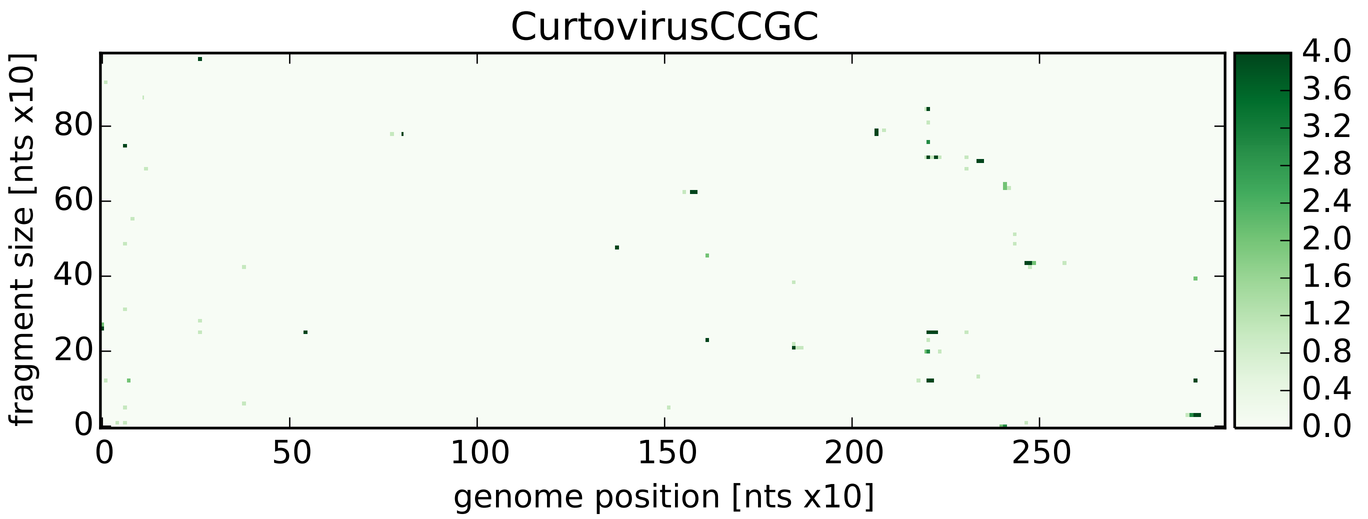
CurtovirusCCGC
fragment size [nts x10]
genome position [nts x10]
0
20
40
60
80
0
50
100
150
200
250
0.0
0.4
0.8
1.2
1.6
2.0
2.4
2.8
3.2
3.6
4.0

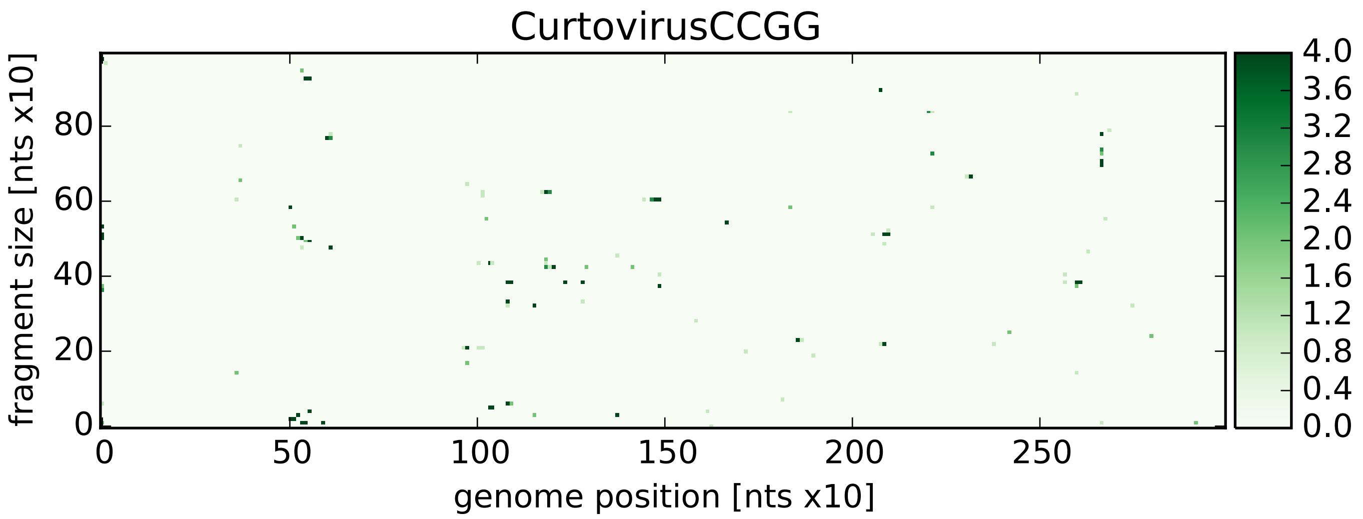

CurtovirusCCGG
fragment size [nts x10]
genome position [nts x10]
0
20
40
60
80
0
50
100
150
200
250
4.0
3.6
3.2
2.8
2.4
2.0
1.6
1.2
0.8
0.4
0.0

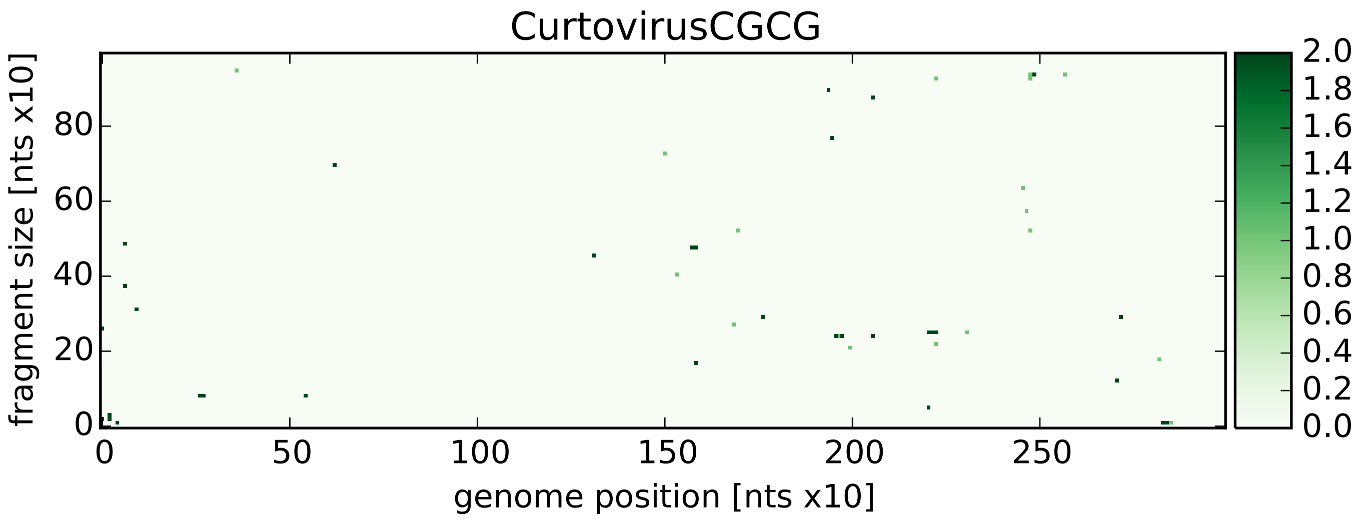
CurtovirusCGCG
fragment size [nts x10]
genome position [nts x10]
0
20
40
60
80
0
50
100
150
200
250
0.0
0.2
0.4
0.6
0.8
1.0
1.2
1.4
1.6
1.8
2.0

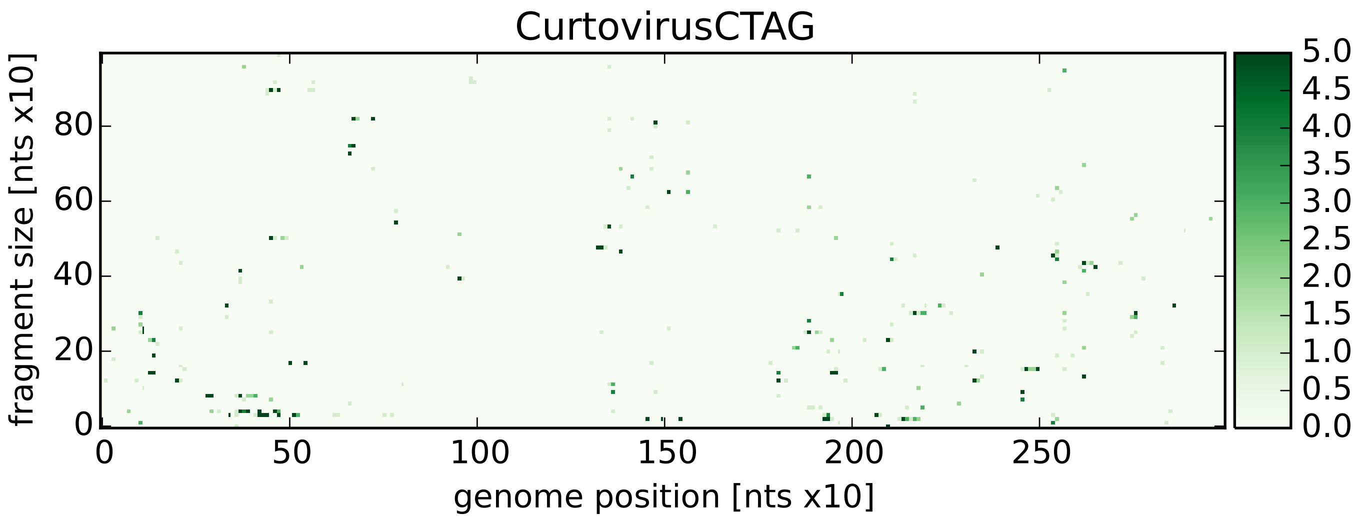

CurtovirusCTAG
fragment size [nts x10]
genome position [nts x10]
0
20
40
60
80
0
50
100
150
200
250
0.0
0.5
1.0
1.5
2.0
2.5
3.0
3.5
4.0
4.5
5.0

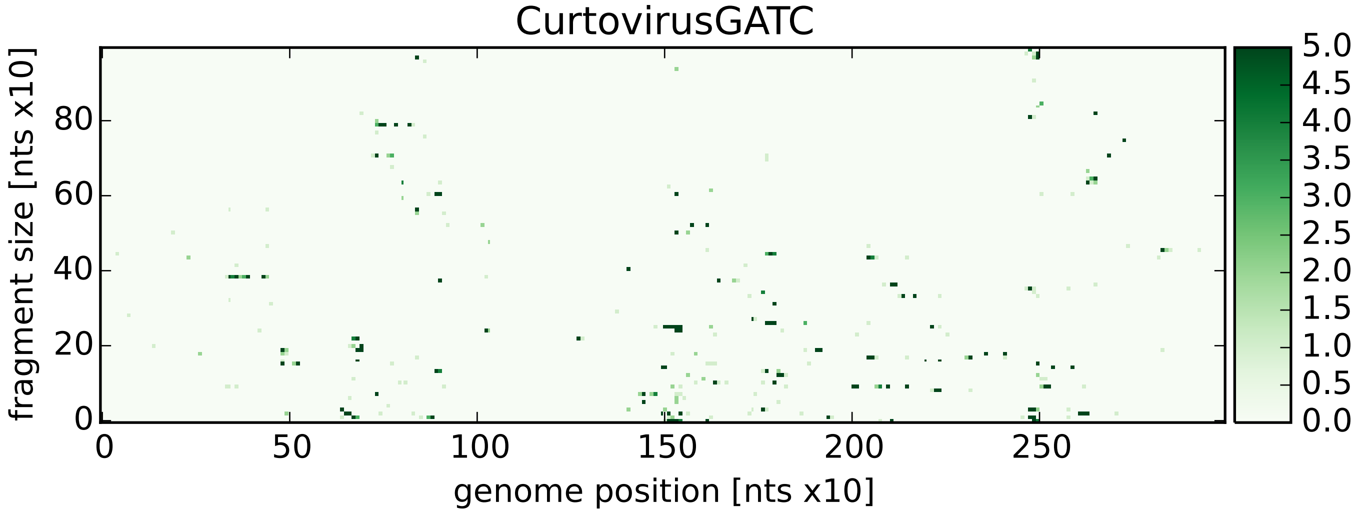
CurtovirusGATC
fragment size [nts x10]
genome position [nts x10]
0
20
40
60
80
0
50
100
150
200
250
0.0
0.5
1.0
1.5
2.0
2.5
3.0
3.5
4.0
4.5
5.0

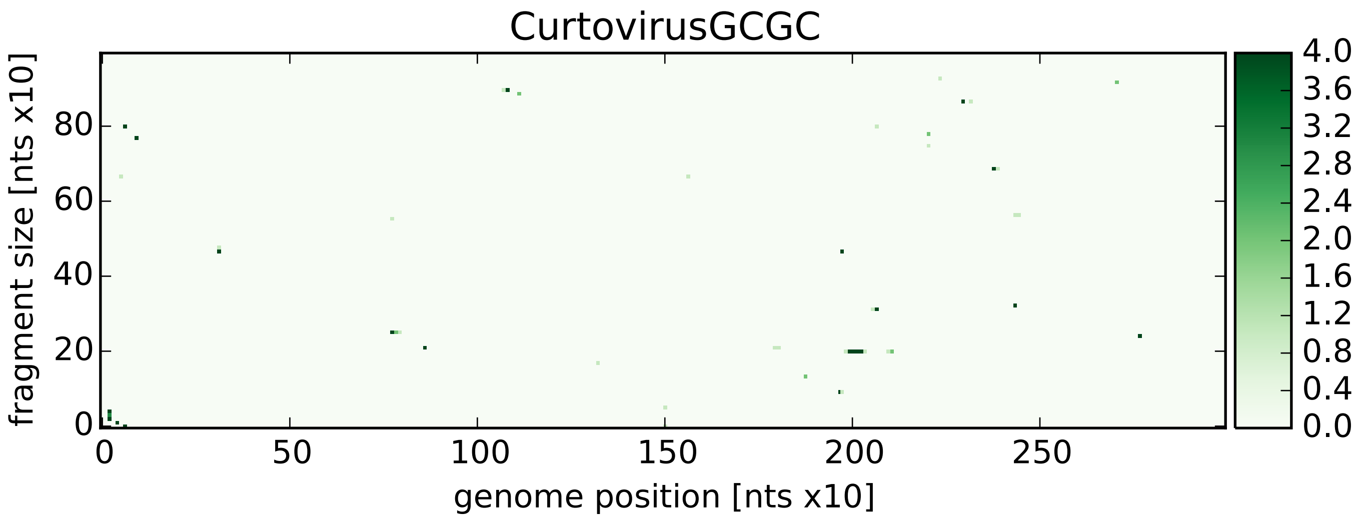
CurtovirusGCGC
fragment size [nts x10]
0
20
40
60
80
genome position [nts x10]
0
50
100
150
200
250
4.0
3.6
3.2
2.8
2.4
2.0
1.6
1.2
0.8
0.4
0.0

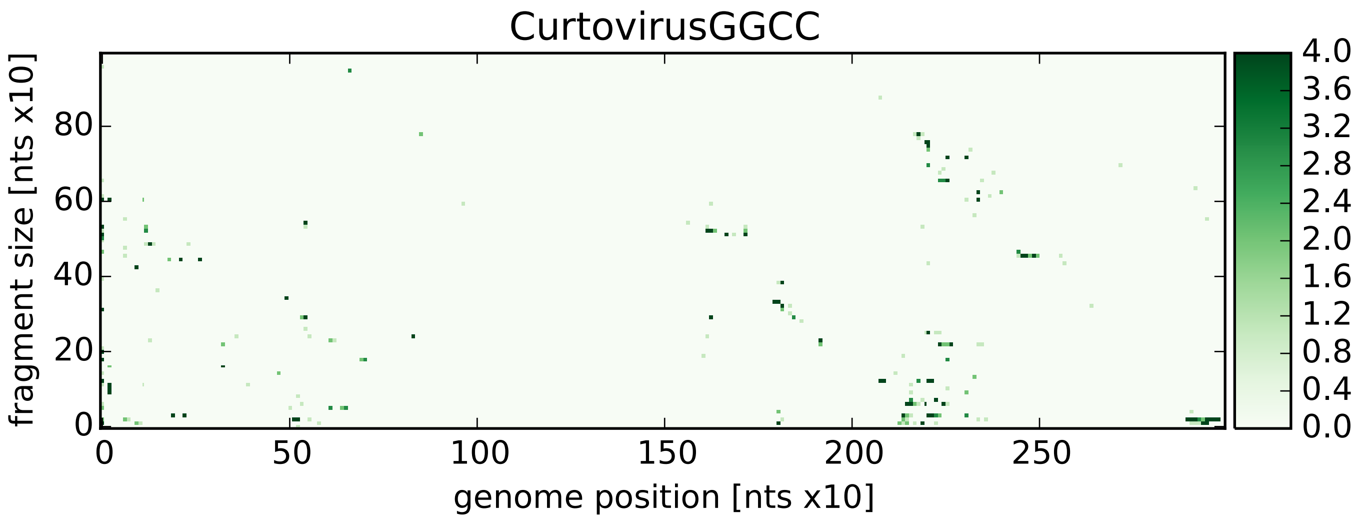
CurtovirusGGCC
fragment size [nts x10]
0
20
40
60
80
genome position [nts x10]
0
50
100
150
200
250
4.0
3.6
3.2
2.8
2.4
2.0
1.6
1.2
0.8
0.4
0.0

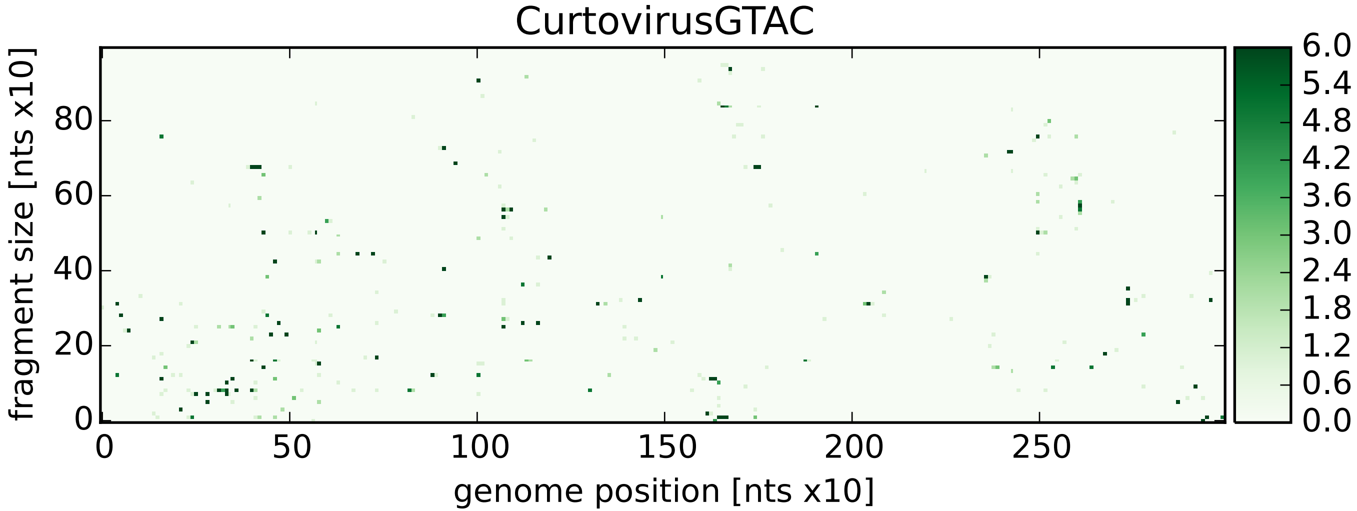

CurtovirusGTAC
fragment size [nts x10]
0
20
40
60
80
genome position [nts x10]
0
50
100
150
200
250
6.0
5.4
4.8
4.2
3.6
3.0
2.4
1.8
1.2
0.6
0.0

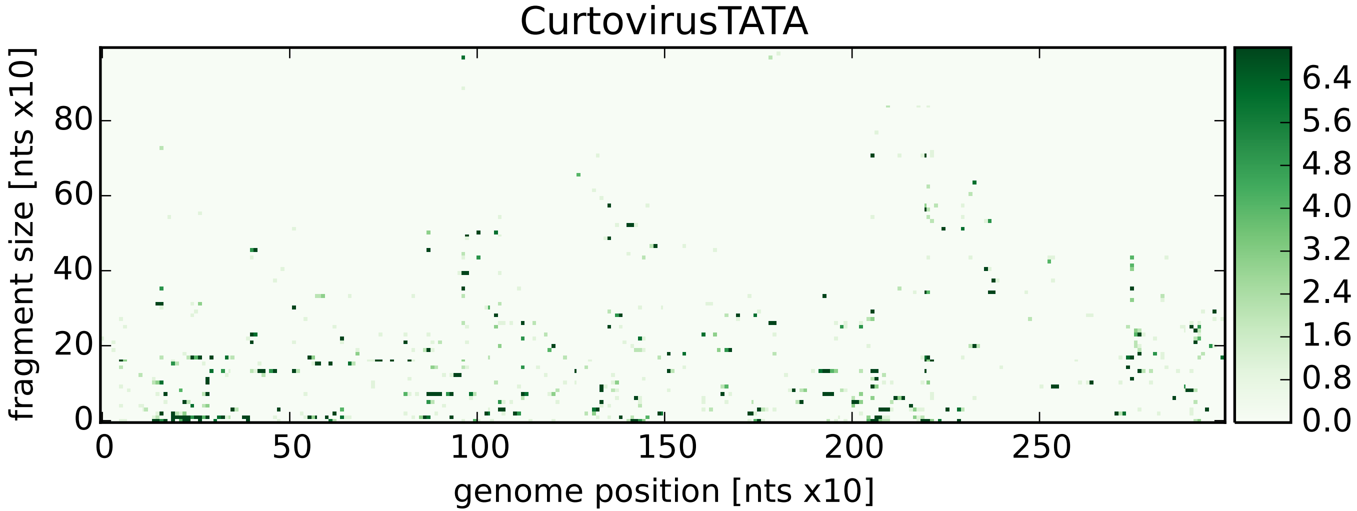

CurtovirusTATA
fragment size [nts x10]
genome position [nts x10]
0
20
40
60
80
0
50
100
150
200
250
0.0
0.8
1.6
2.4
3.2
4.0
4.8
5.6
6.4

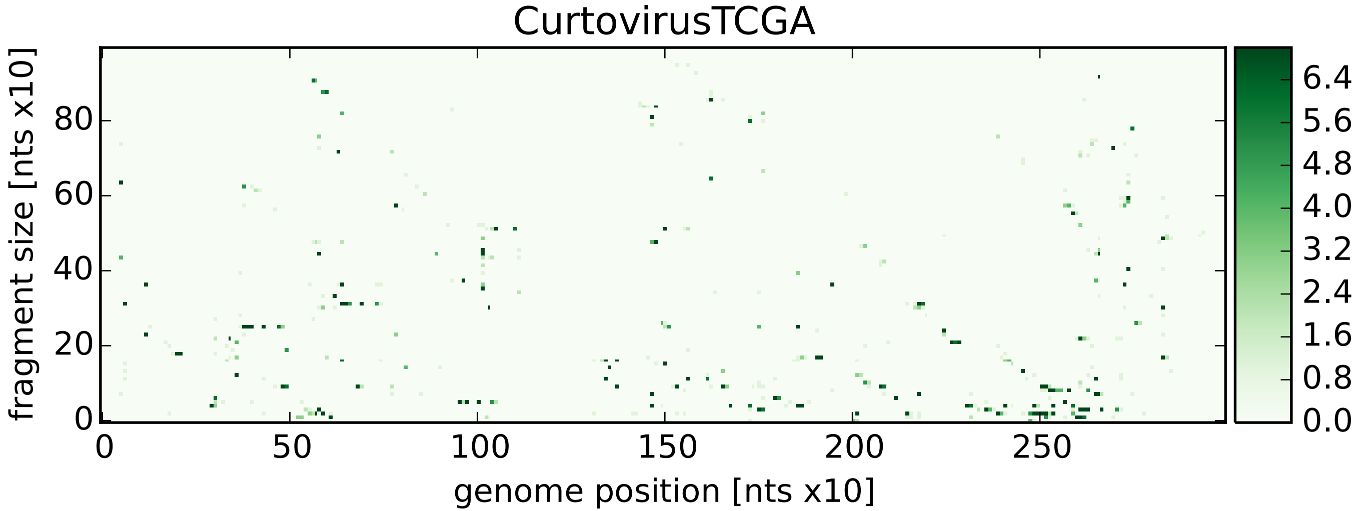

CurtovirusTCGA
fragment size [nts x10]
genome position [nts x10]
0
20
40
60
80
0
50
100
150
200
250
0.0
0.8
1.6
2.4
3.2
4.0
4.8
5.6
6.4

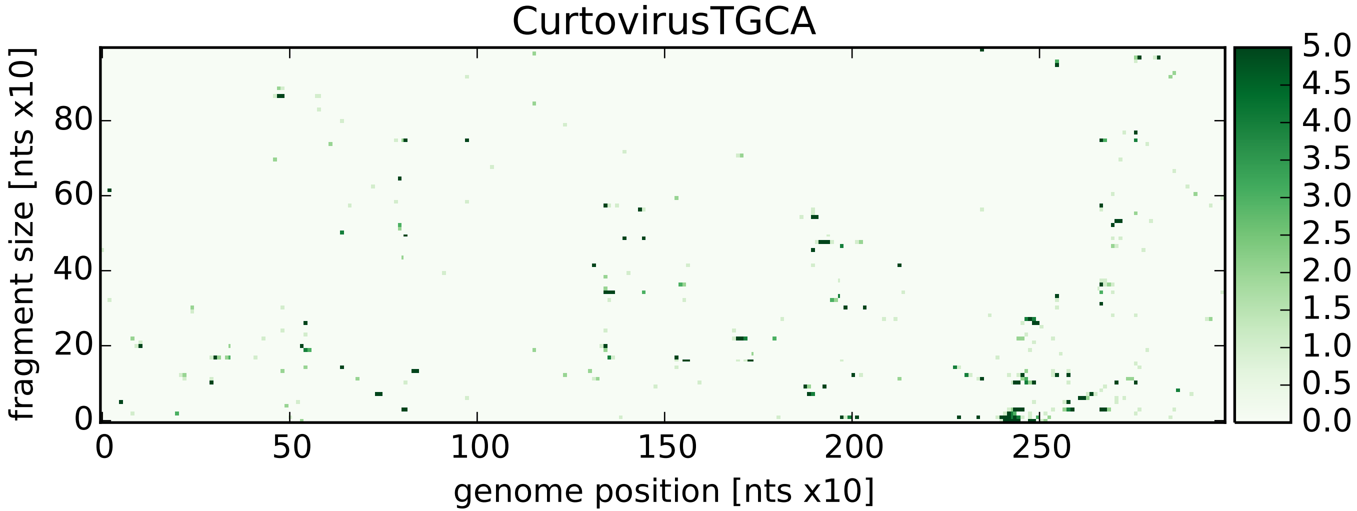

CurtovirusTGCA
fragment size [nts x10]
genome position [nts x10]
0
20
40
60
80
0
50
100
150
200
250
0.0
0.5
1.0
1.5
2.0
2.5
3.0
3.5
4.0
4.5
5.0

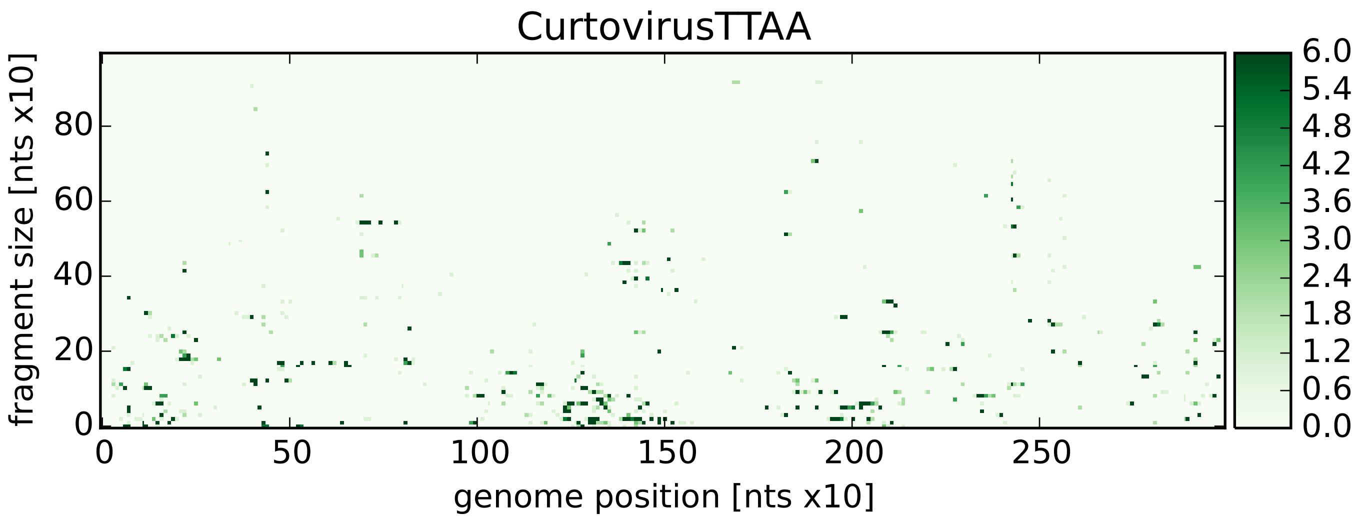

CurtovirusTTAA
fragment size [nts x10]
genome position [nts x10]
0
20
40
60
80
0
50
100
150
200
250
0.0
0.6
1.2
1.8
2.4
3.0
3.6
4.2
4.8
5.4
6.0

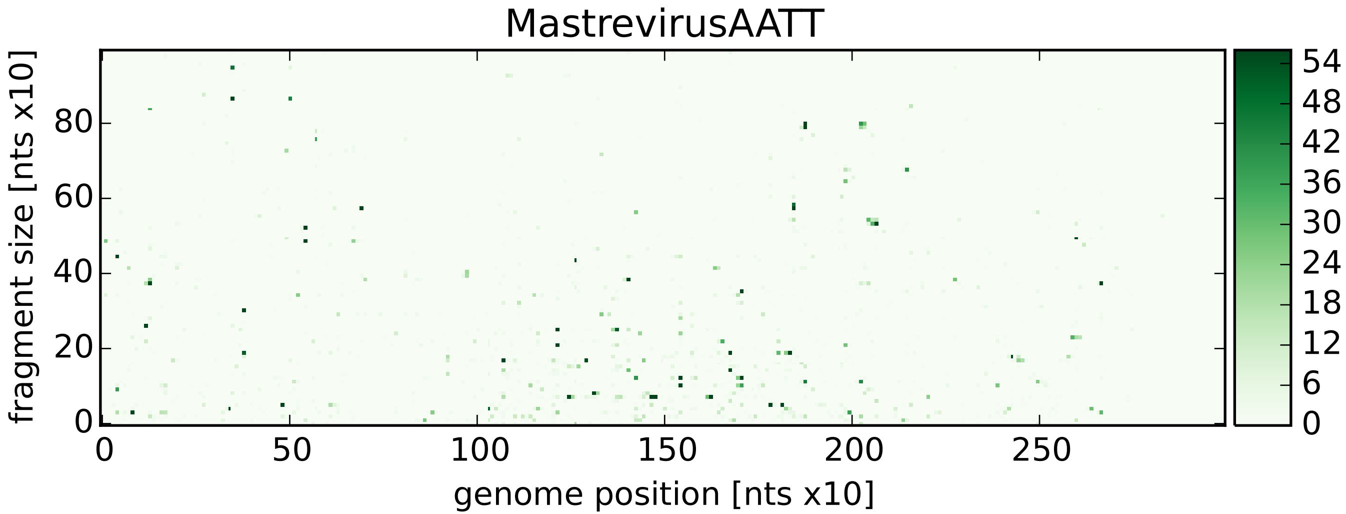
MastrevirusAATT
fragment size [nts x10]
genome position [nts x10]
0
20
40
60
80
0
50
100
150
200
250
0
6
12
18
24
30
36
42
48
54

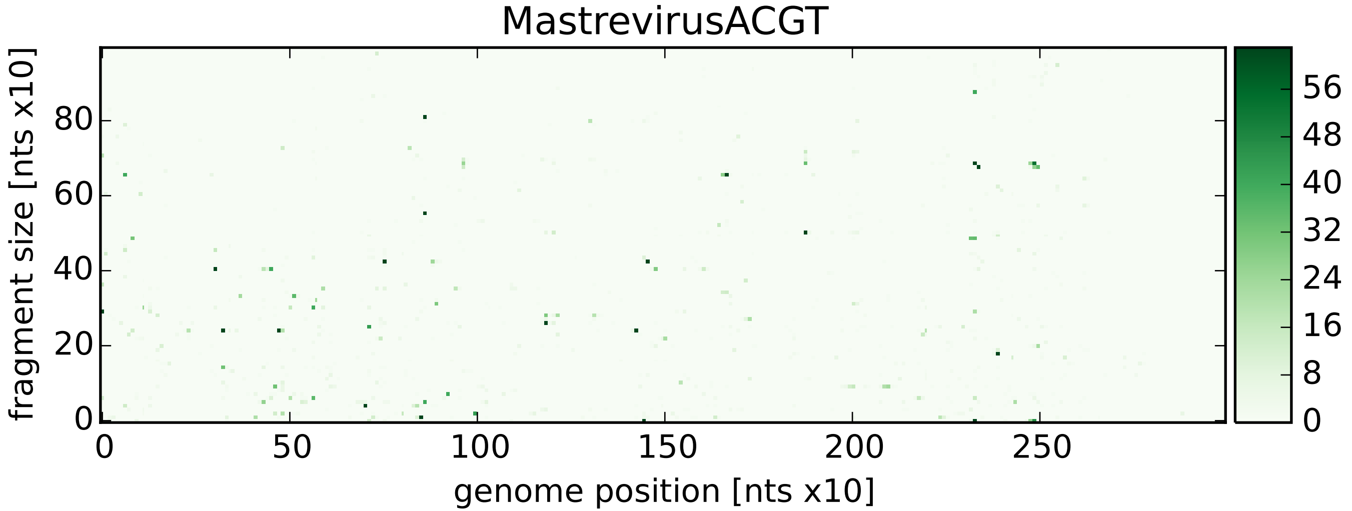
MastrevirusACGT
fragment size [nts x10]
genome position [nts x10]
0
20
40
60
80
0
50
100
150
200
250
0
8
16
24
32
40
48
56

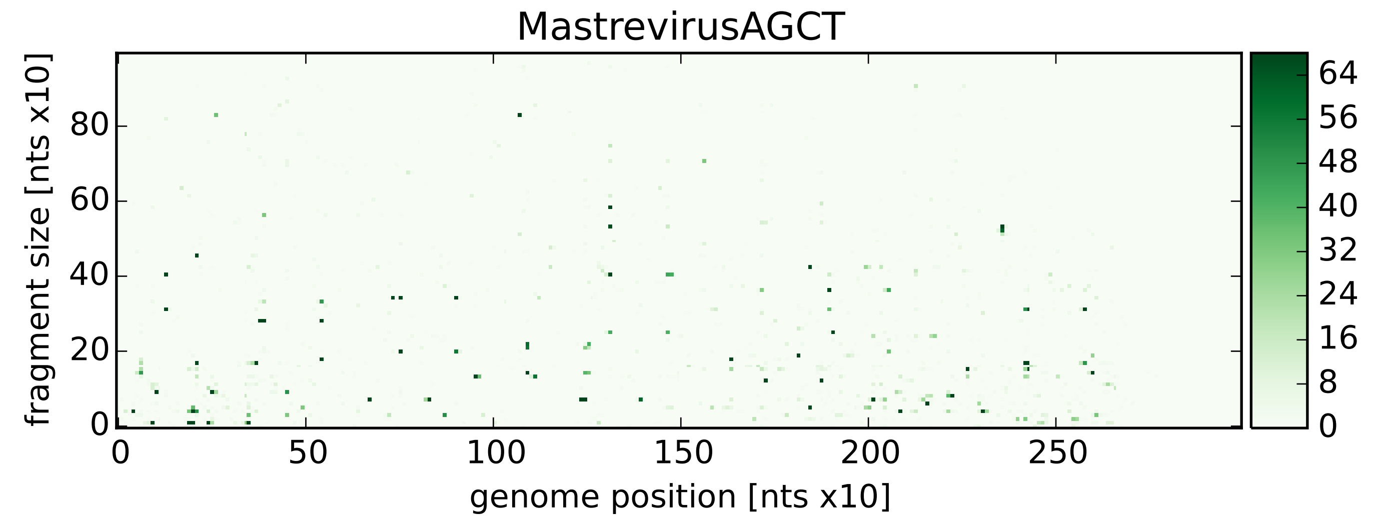
MastrevirusAGCT
fragment size [nts x10]
genome position [nts x10]
0
20
40
60
80
0
50
100
150
200
250
0
8
16
24
32
40
48
56
64

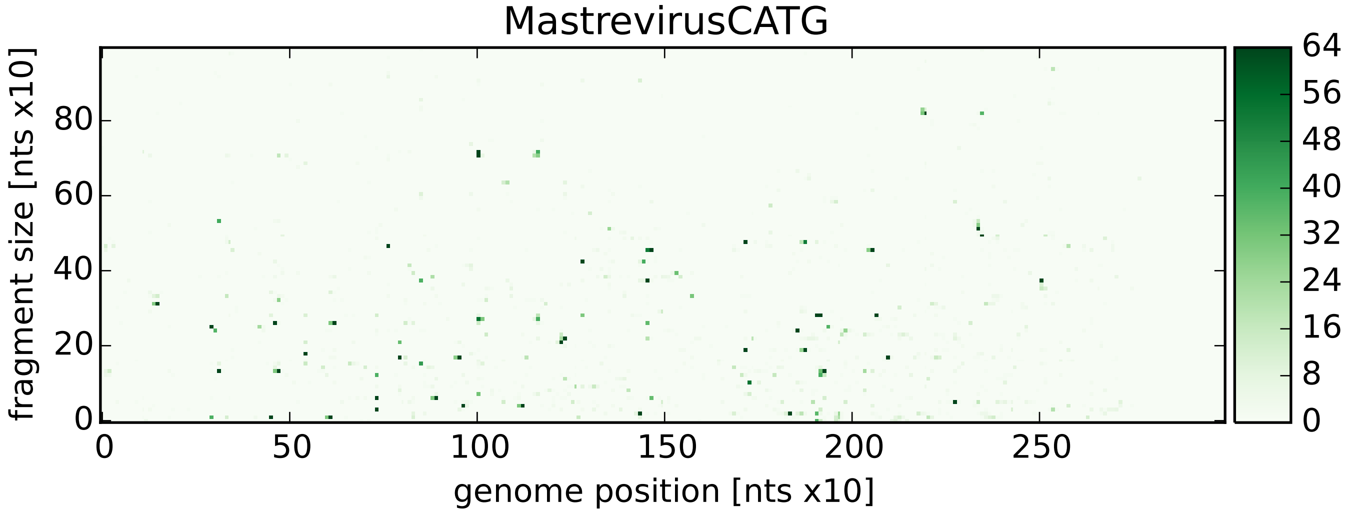
MastrevirusCATG
fragment size [nts x10]
genome position [nts x10]
0
20
40
60
80
0
50
100
150
200
250
64
56
48
40
32
24
16
8
0

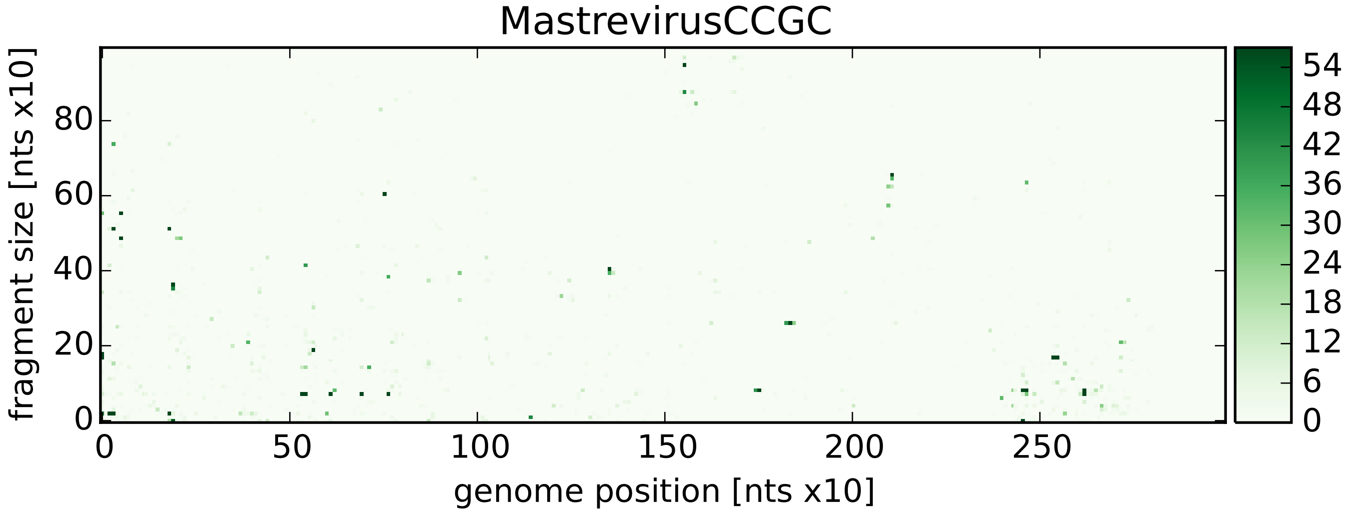

MastrevirusCCGC
fragment size [nts x10]
genome position [nts x10]
0
20
40
60
80
0
50
100
150
200
250
0
6
12
18
24
30
36
42
48
54

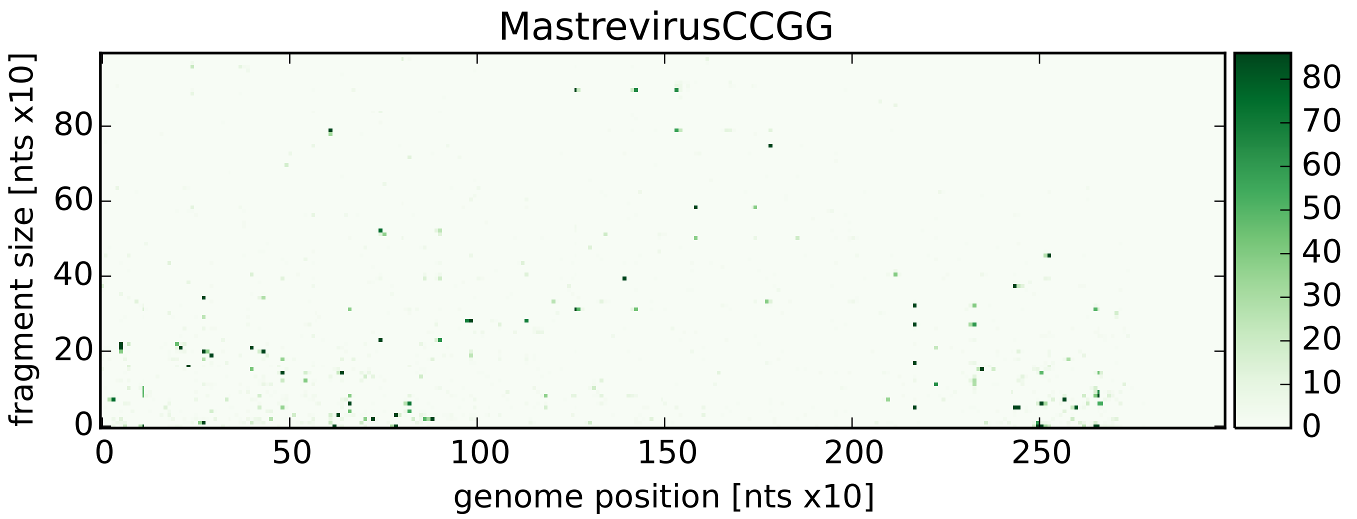

MastrevirusCCGG
fragment size [nts x10]
genome position [nts x10]
0
20
40
60
80
0
50
100
150
200
250
0
10
20
30
40
50
60
70
80

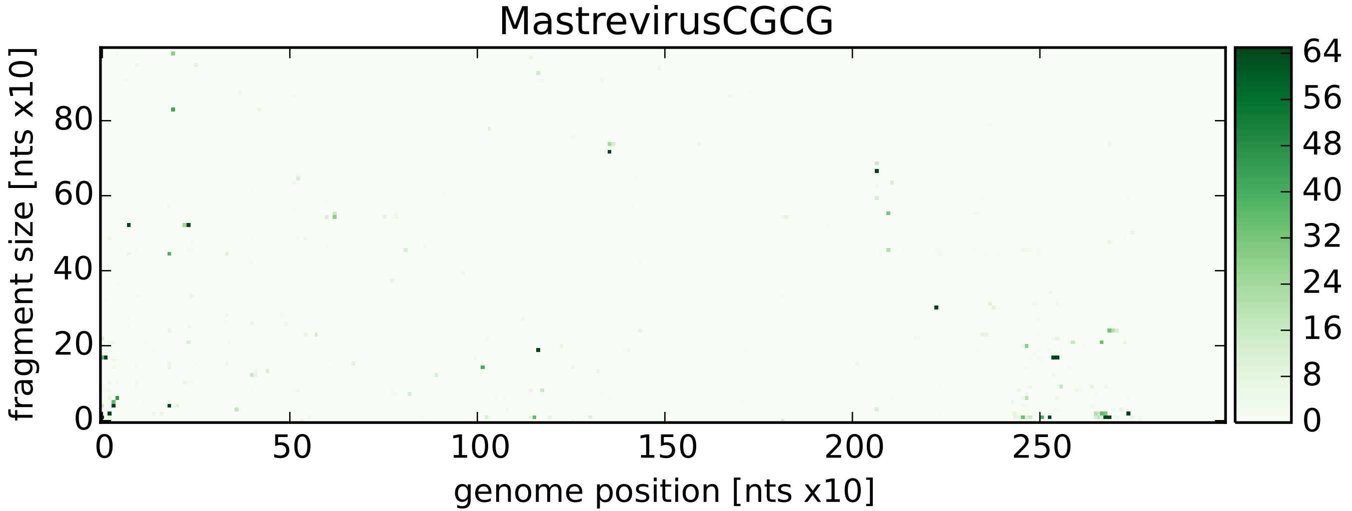
MastrevirusCGCG
fragment size [nts x10]
genome position [nts x10]
0
20
40
60
80
50
100
150
200
250
0
8
16
24
32
40
48
56
64

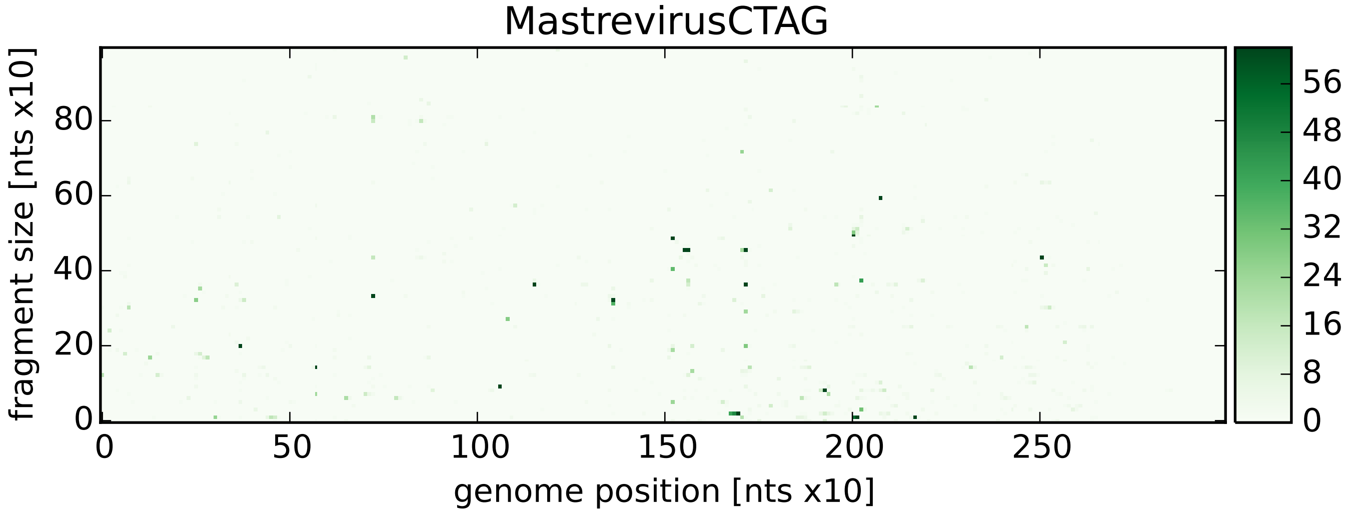
MastrevirusCTAG
fragment size [nts x10]
genome position [nts x10]
0
50
100
150
200
250
0
20
40
60
80
0
8
16
24
32
40
48
56

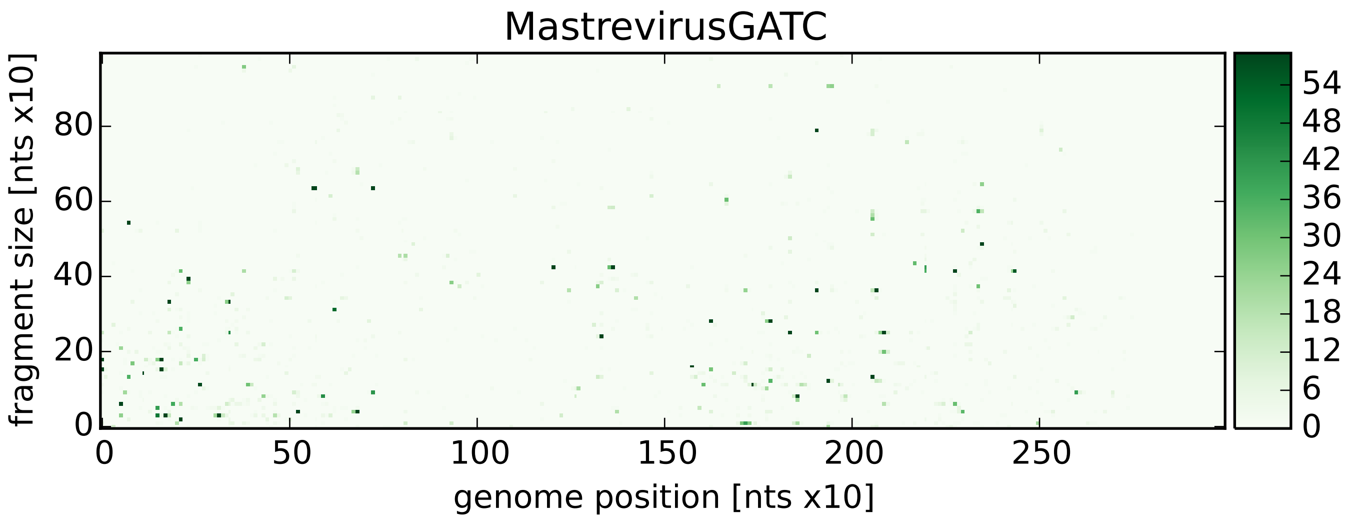
MastrevirusGATC
fragment size [nts x10]
genome position [nts x10]
0
20
40
60
80
0
50
100
150
200
250
0
6
12
18
24
30
36
42
48
54

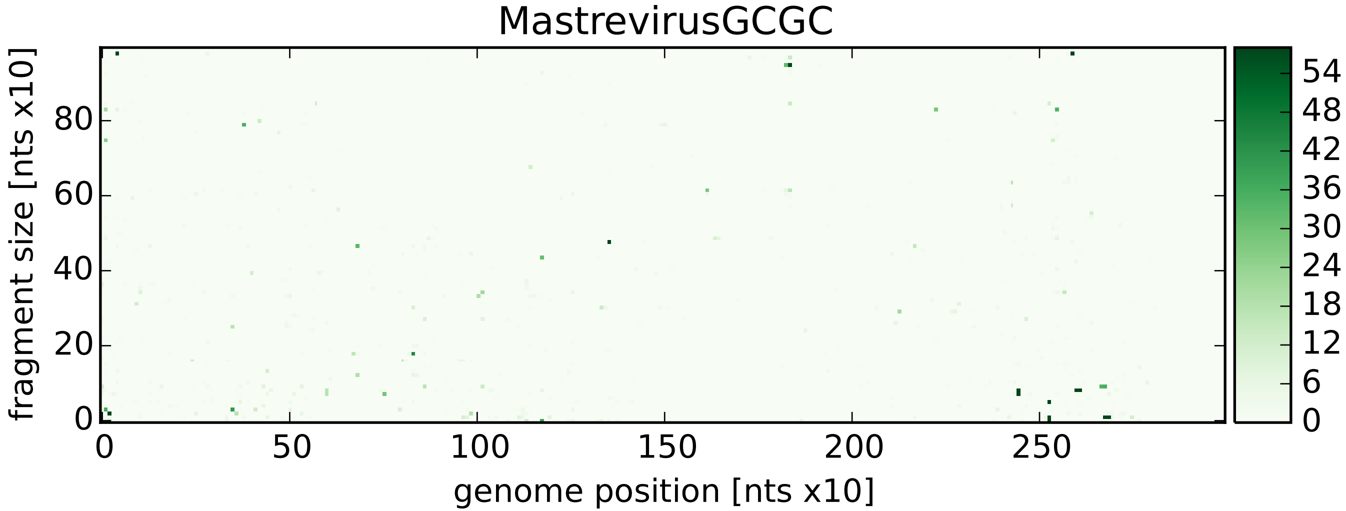

MastrevirusGCGC
fragment size [nts x10]
0
20
40
60
80
genome position [nts x10]
0
50
100
150
200
250
0
6
12
18
24
30
36
42
48
54

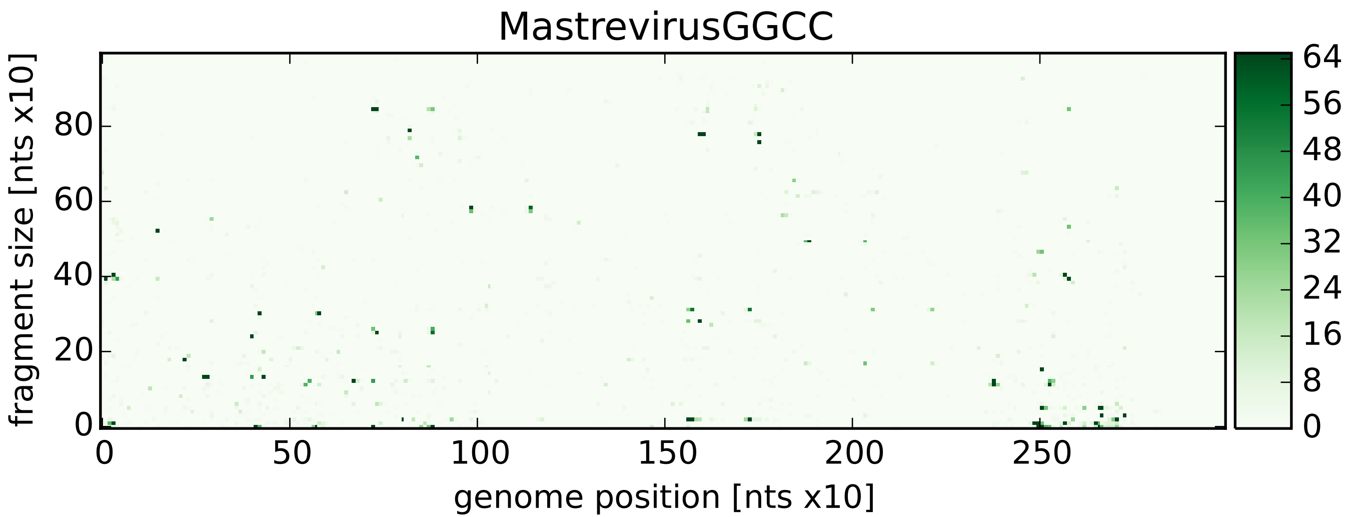

MastrevirusGGCC
fragment size [nts x10]
genome position [nts x10]
0
20
40
60
80
0
50
100
150
200
250
0
8
16
24
32
40
48
56
64

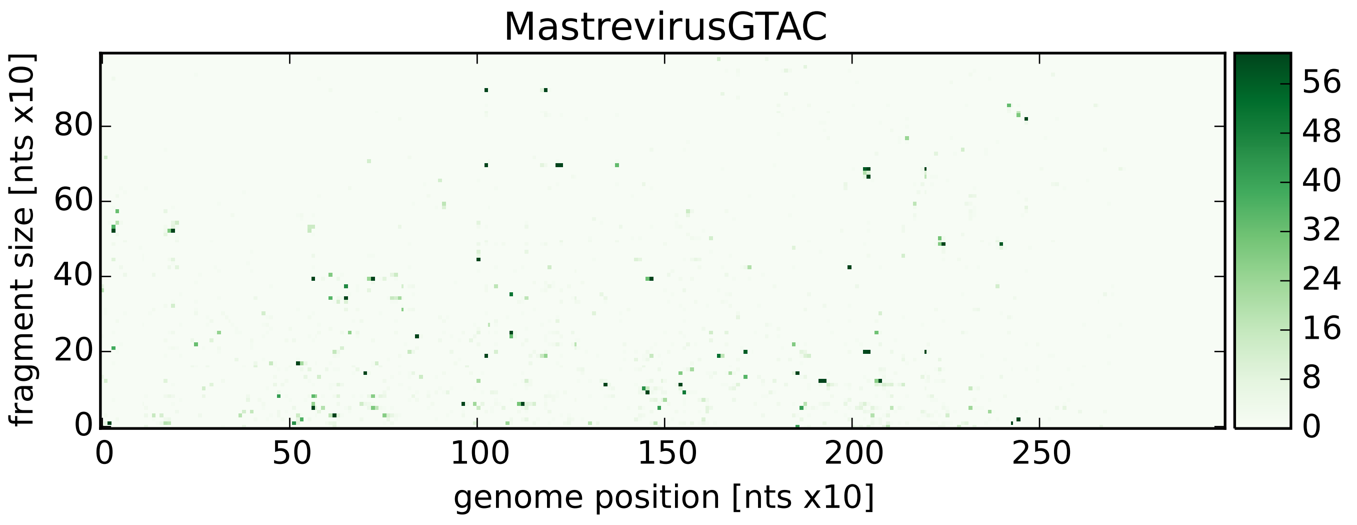

MastrevirusGTAC
fragment size [nts x10]
genome position [nts x10]
0
20
40
60
80
0
50
100
150
200
250
0
8
16
24
32
40
48
56

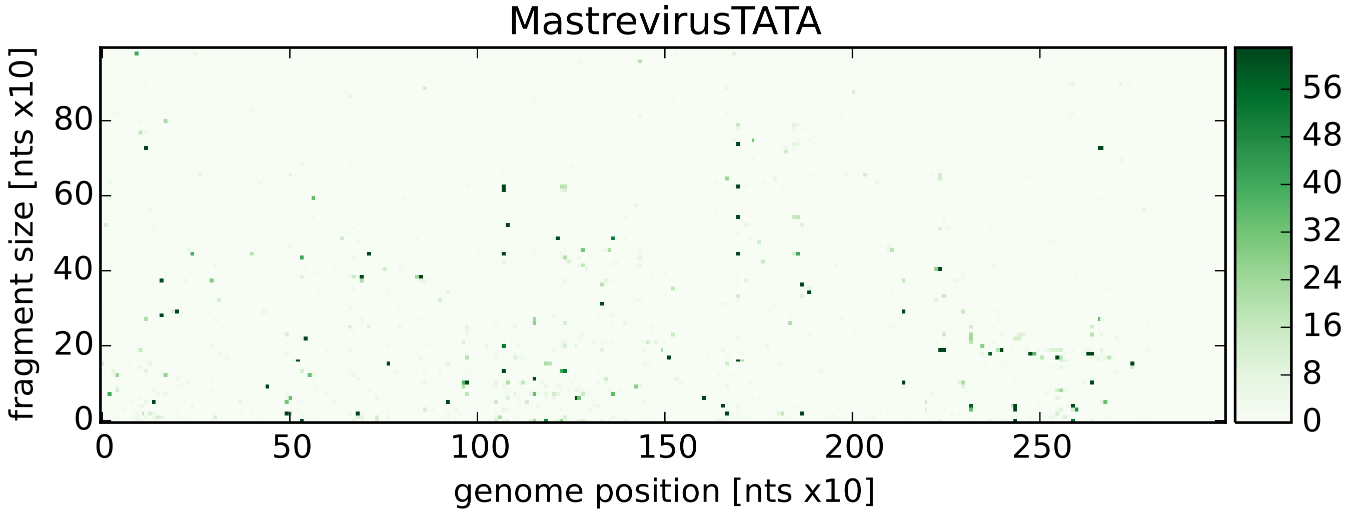

MastrevirusTATA
fragment size [nts x10]
genome position [nts x10]
0
20
40
60
80
0
50
100
150
200
250
0
8
16
24
32
40
48
56

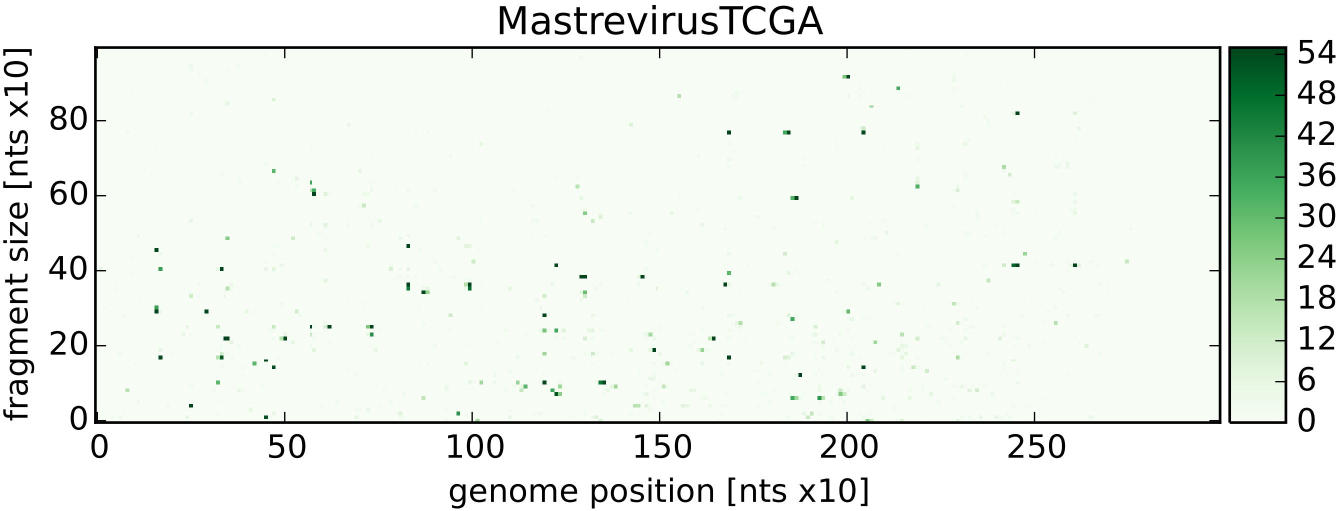

MastrevirusTCGA
fragment size [nts x10]
genome position [nts x10]
0
20
40
60
80
0
50
100
150
200
250
0
6
12
18
24
30
36
42
48
54

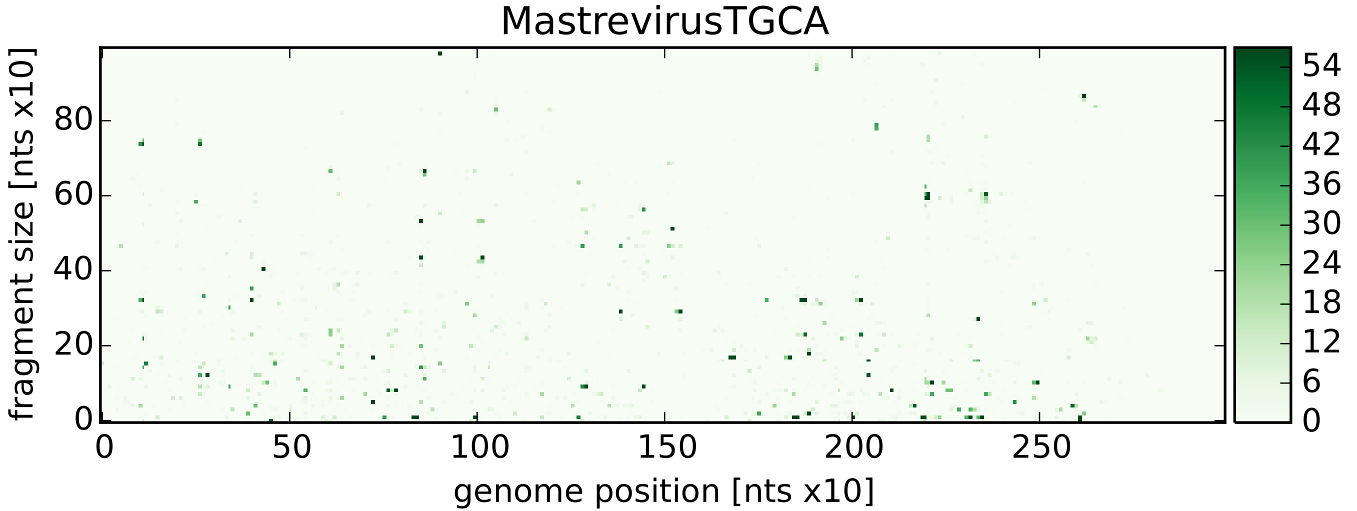
MastrevirusTGCA
fragment size [nts x10]
0
20
40
60
80
genome position [nts x10]
0
50
100
150
200
250
0
6
12
18
24
30
36
42
48
54

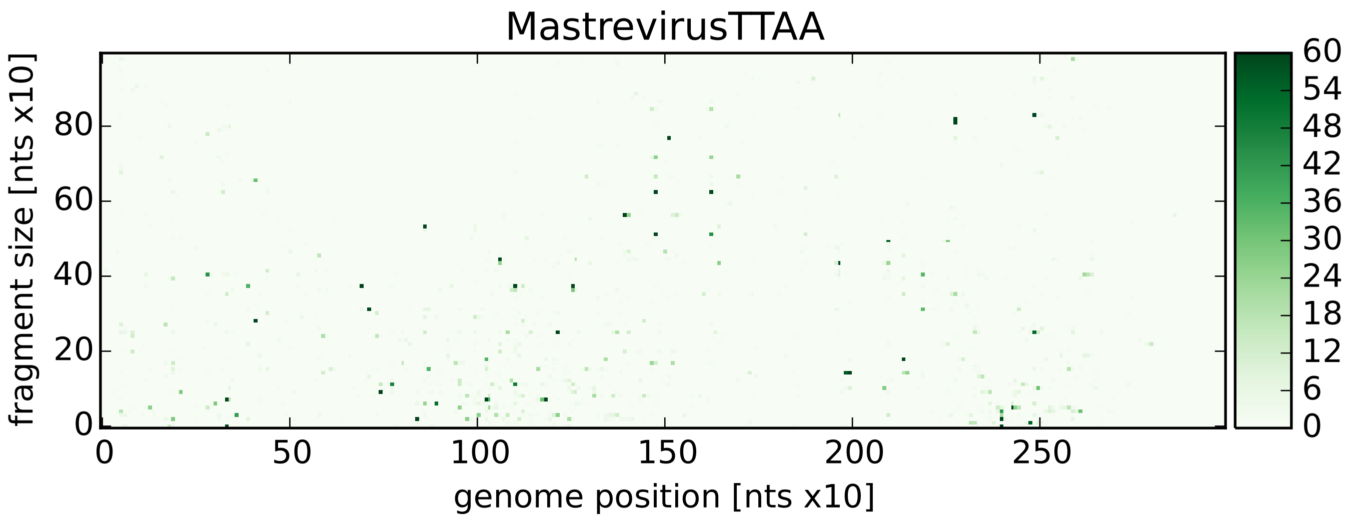
MastrevirusTTAA
fragment size [nts x10]
genome position [nts x10]
0
20
40
60
80
0
50
100
150
200
250
0
6
12
18
24
30
36
42
48
54
60

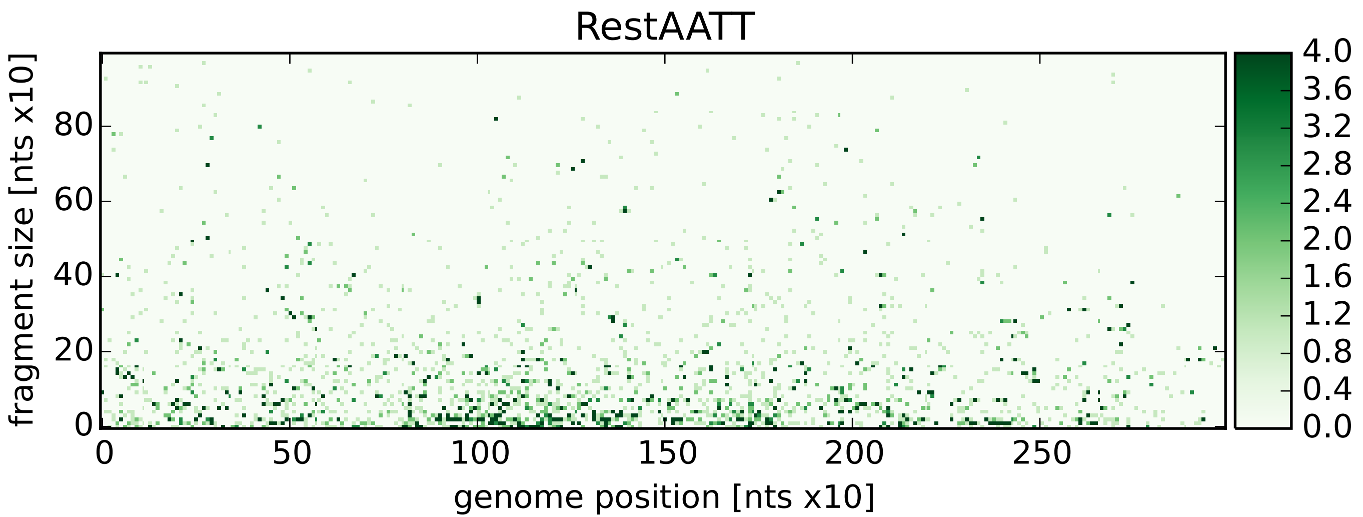

RestAATT
fragment size [nts x10]
genome position [nts x10]
0
20
40
60
80
0
50
100
150
200
250
0.0
0.4
0.8
1.2
1.6
2.0
2.4
2.8
3.2
3.6
4.0

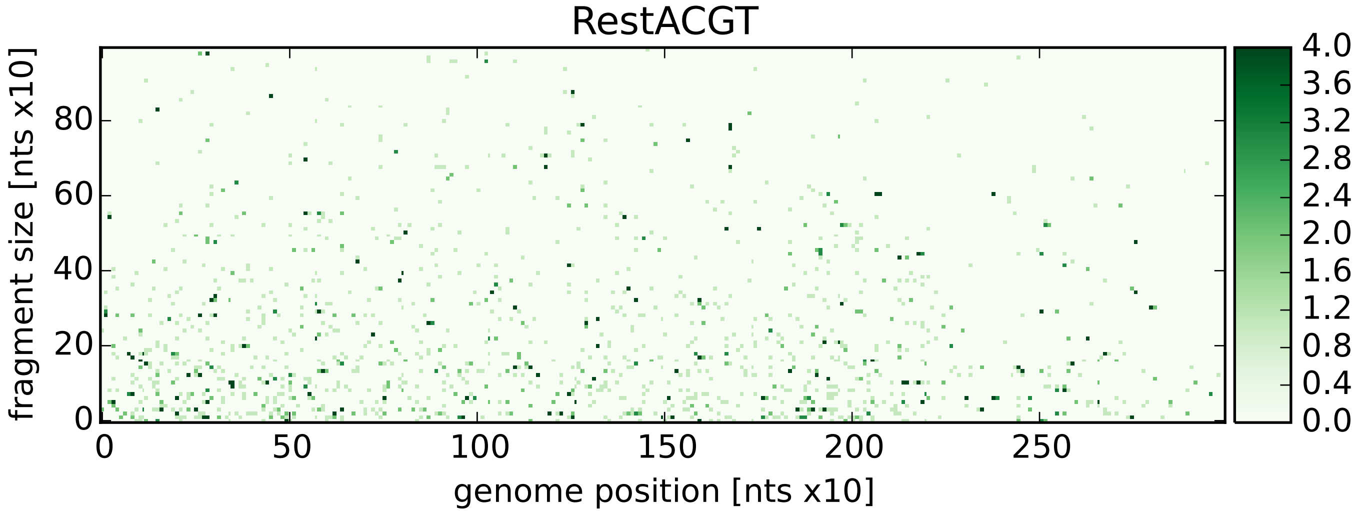

RestACGT
fragment size [nts x10]
genome position [nts x10]
0
20
40
60
80
0
50
100
150
200
250
4.0
3.6
3.2
2.8
2.4
2.0
1.6
1.2
0.8
0.4
0.0

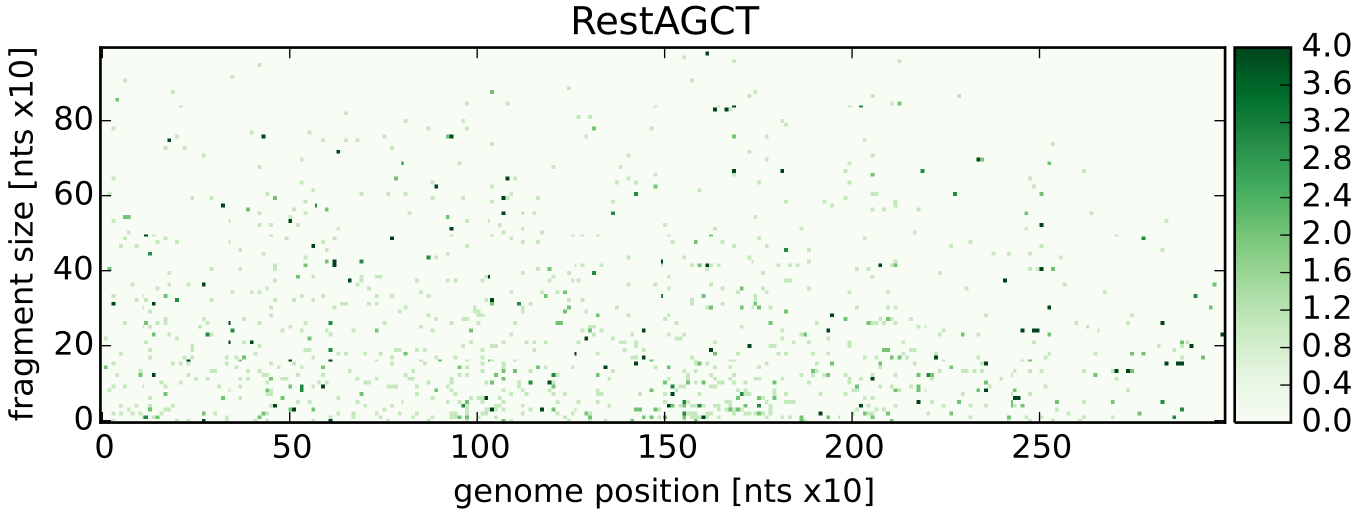
RestAGCT
fragment size [nts x10]
genome position [nts x10]
0
20
40
60
80
0
50
100
150
200
250
0.0
0.4
0.8
1.2
1.6
2.0
2.4
2.8
3.2
3.6
4.0

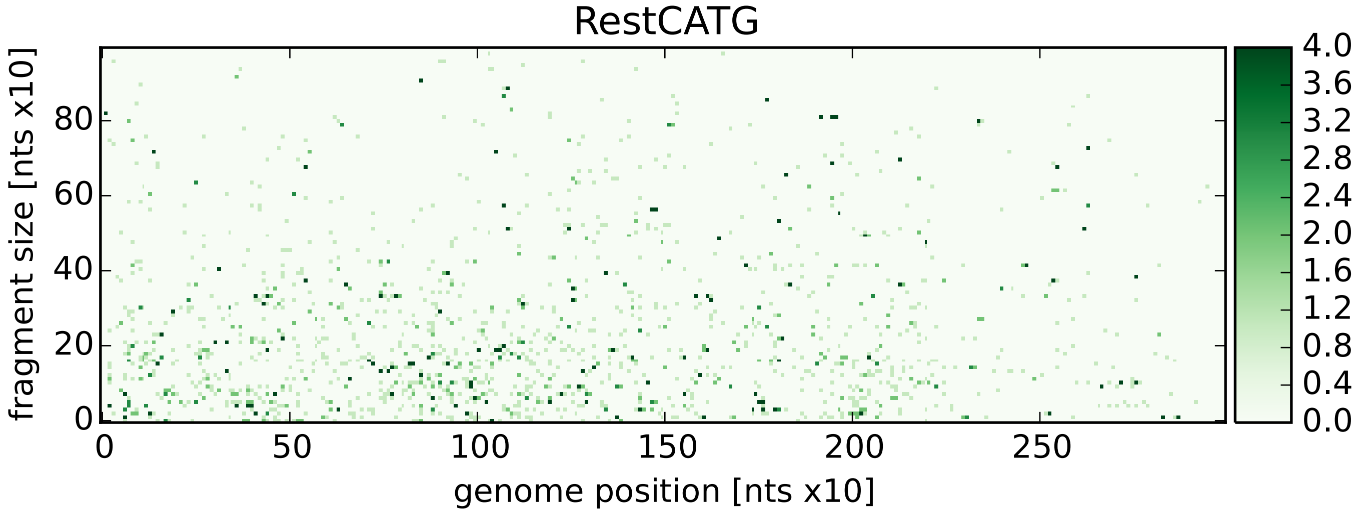

RestCATG
fragment size [nts x10]
genome position [nts x10]
0
20
40
60
80
0
50
100
150
200
250
0.0
0.4
0.8
1.2
1.6
2.0
2.4
2.8
3.2
3.6
4.0

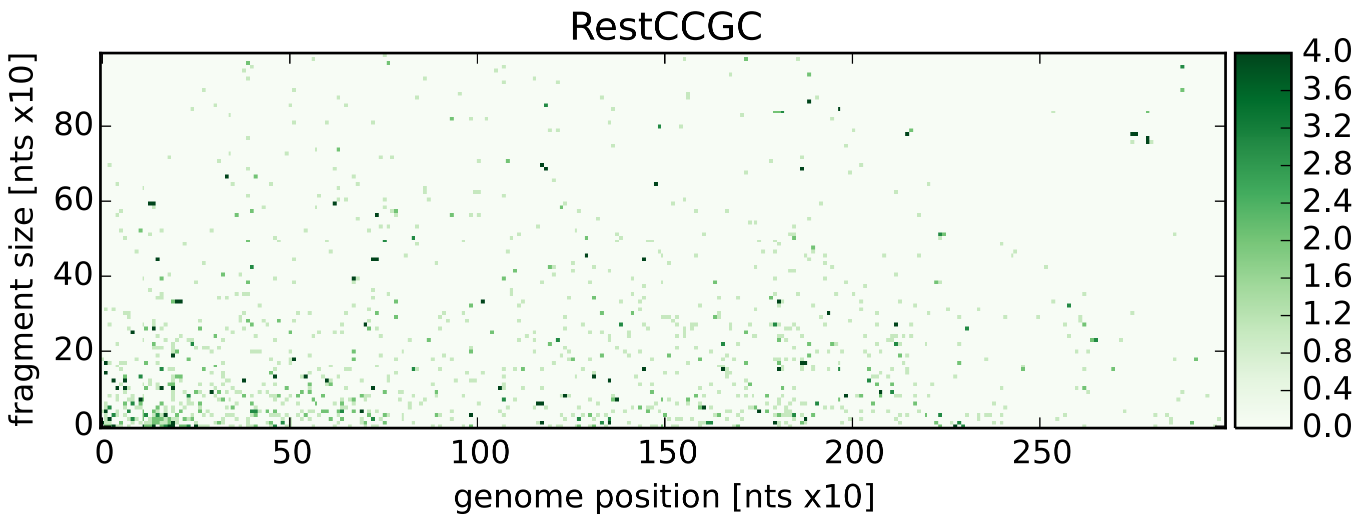
RestCCGC
fragment size [nts x10]
genome position [nts x10]
0
20
40
60
80
0
50
100
150
200
250
0.0
0.4
0.8
1.2
1.6
2.0
2.4
2.8
3.2
3.6
4.0

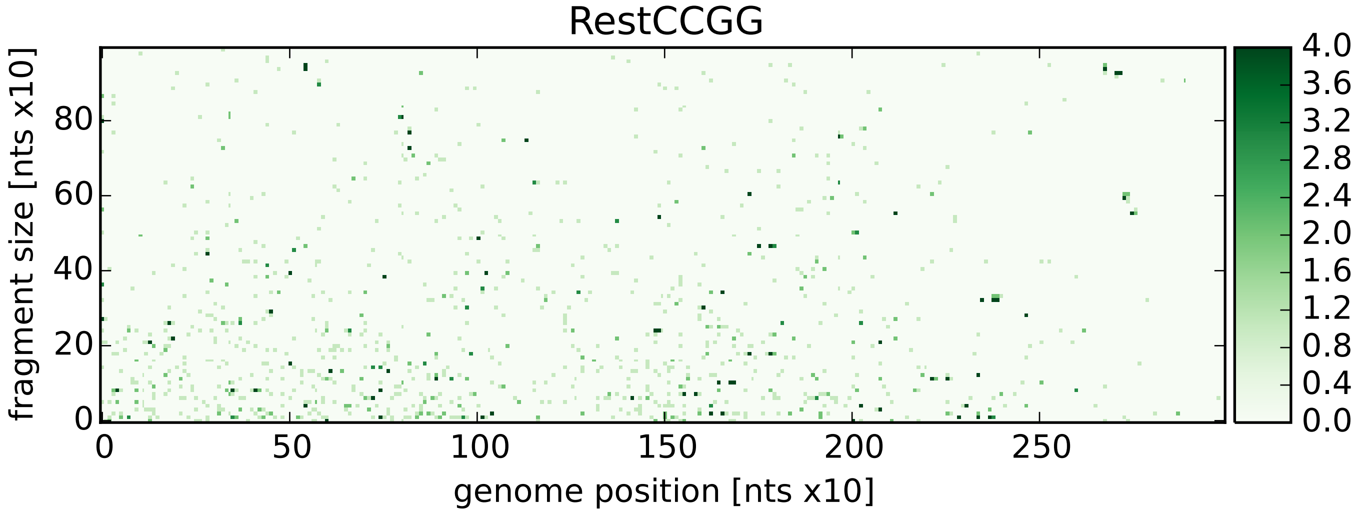

RestCCGG
fragment size [nts x10]
genome position [nts x10]
0
20
40
60
80
0
50
100
150
200
250
0.0
0.4
0.8
1.2
1.6
2.0
2.4
2.8
3.2
3.6
4.0

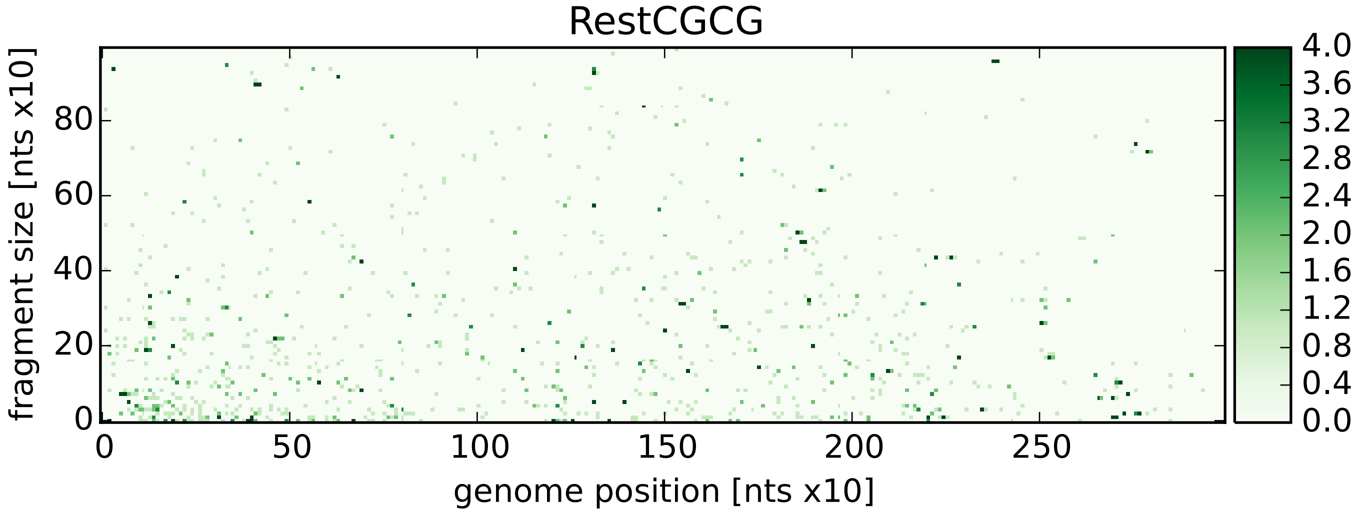
RestCGCG
fragment size [nts x10]
0
20
40
60
80
0
50
100
150
200
250
genome position [nts x10]
4.0
3.6
3.2
2.8
2.4
2.0
1.6
1.2
0.8
0.4
0.0

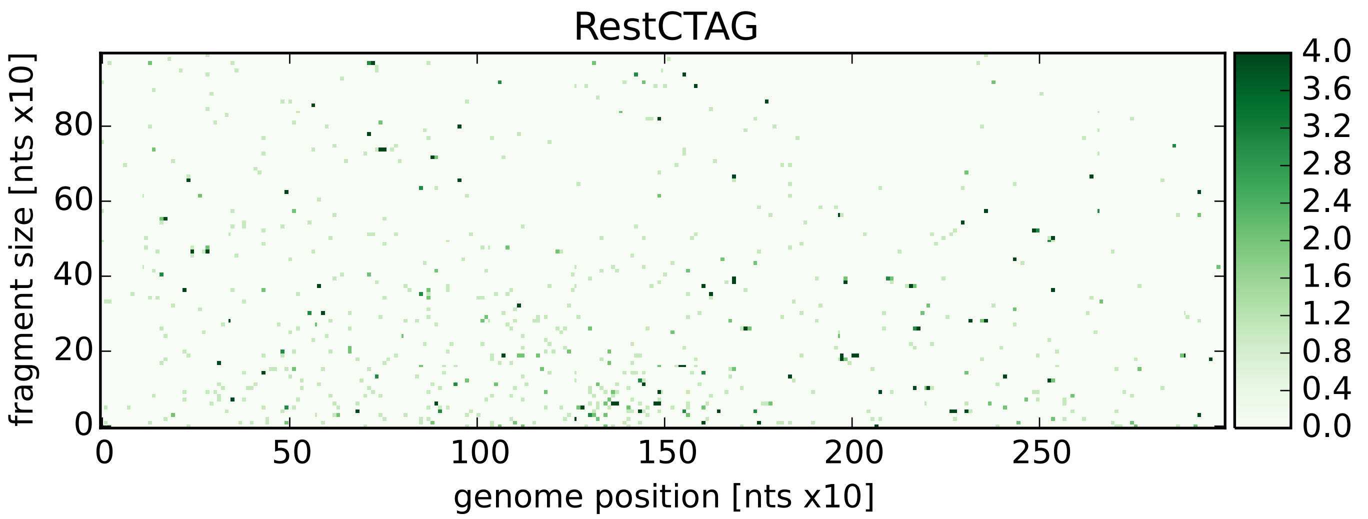

RestCTAG
fragment size [nts x10]
genome position [nts x10]
0
20
40
60
80
0
50
100
150
200
250
0.0
0.4
0.8
1.2
1.6
2.0
2.4
2.8
3.2
3.6
4.0

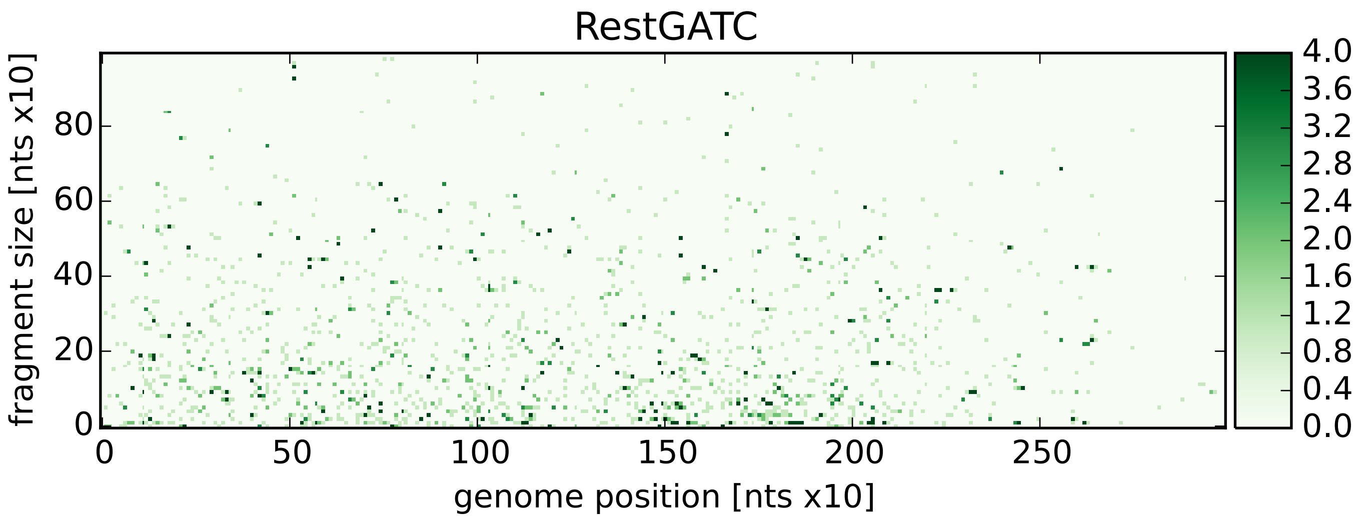
RestGATC
fragment size [nts x10]
genome position [nts x10]
0
20
40
60
80
0
50
100
150
200
250
0.0
0.4
0.8
1.2
1.6
2.0
2.4
2.8
3.2
3.6
4.0

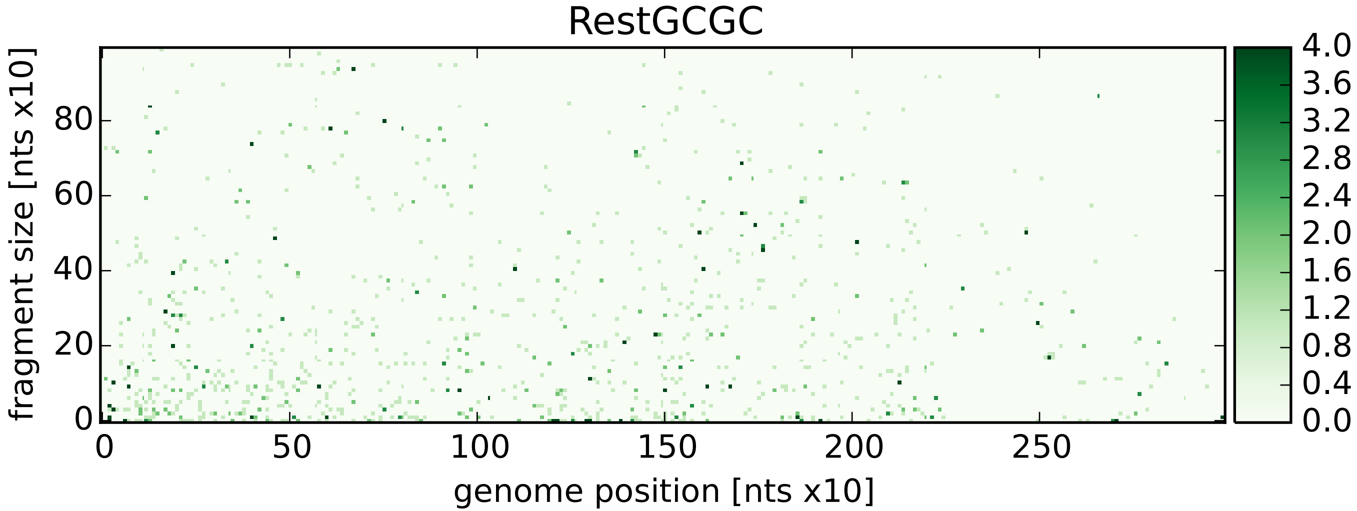
RestGCGC
fragment size [nts x10]
0
20
40
60
80
genome position [nts x10]
0
50
100
150
200
250
0.0
0.4
0.8
1.2
1.6
2.0
2.4
2.8
3.2
3.6
4.0

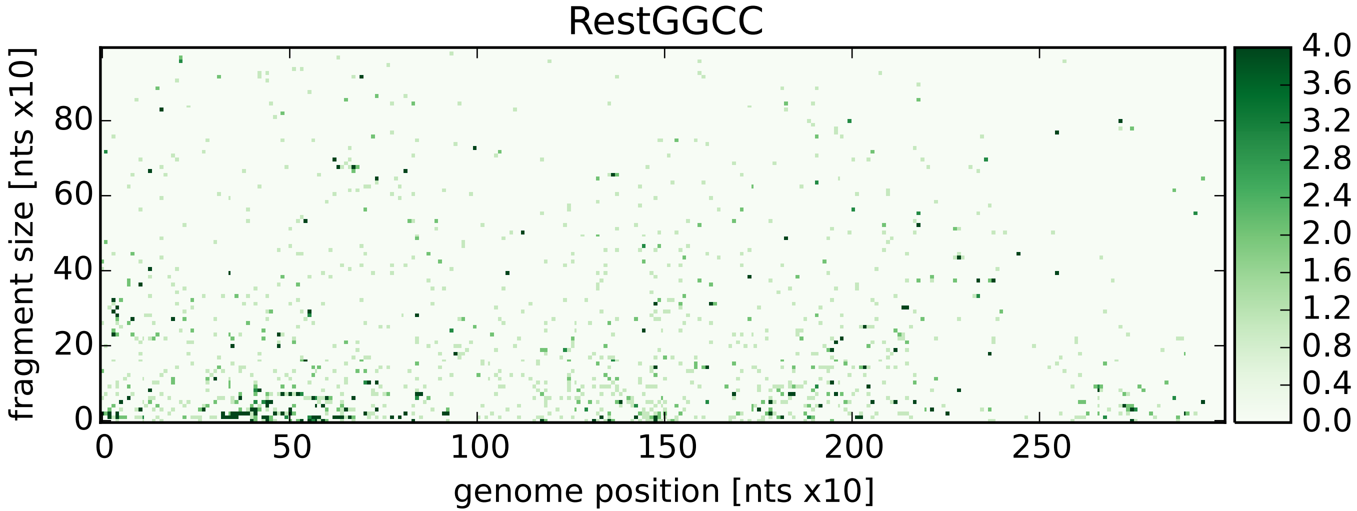

RestGGCC
fragment size [nts x10]
genome position [nts x10]
0
20
40
60
80
0
50
100
150
200
250
4.0
3.6
3.2
2.8
2.4
2.0
1.6
1.2
0.8
0.4
0.0

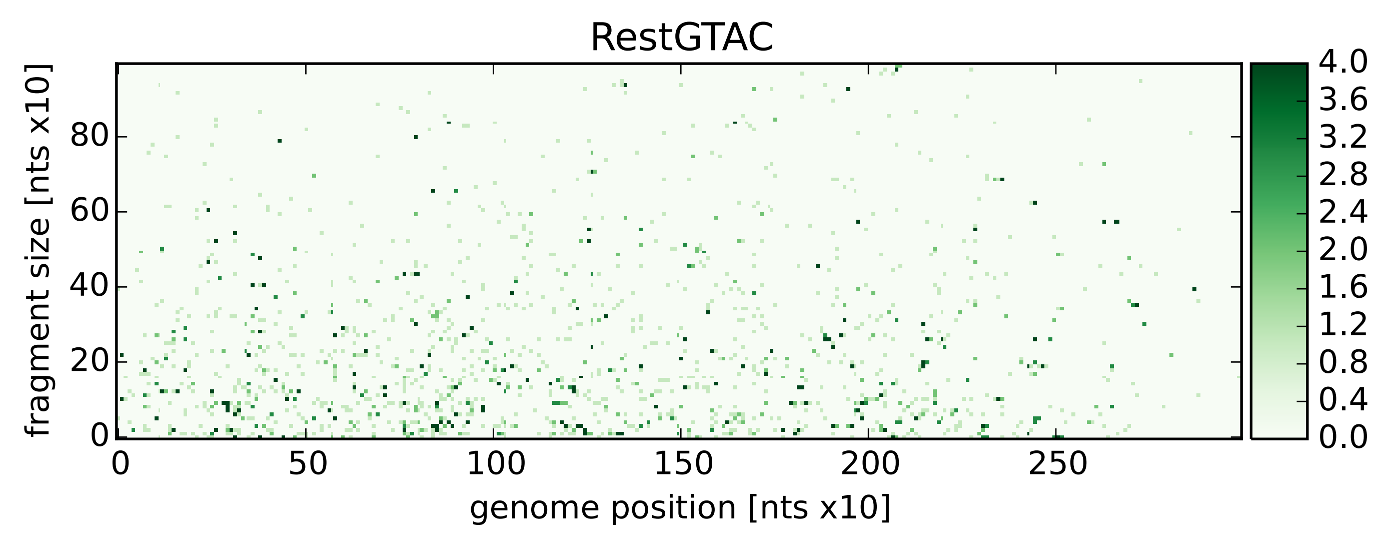
RestGTAC
fragment size [nts x10]
genome position [nts x10]
0
50
100
150
200
250
0
20
40
60
80
0.0
0.4
0.8
1.2
1.6
2.0
2.4
2.8
3.2
3.6
4.0

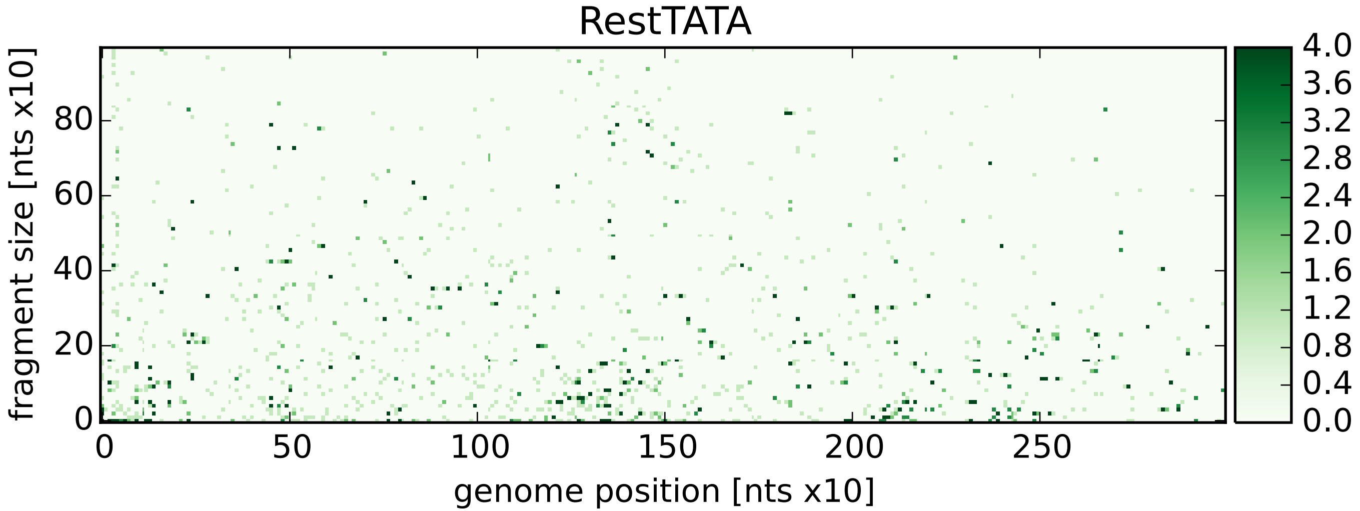
RestTATA
fragment size [nts x10]
genome position [nts x10]
0
20
40
60
80
0
50
100
150
200
250
0.0
0.4
0.8
1.2
1.6
2.0
2.4
2.8
3.2
3.6
4.0

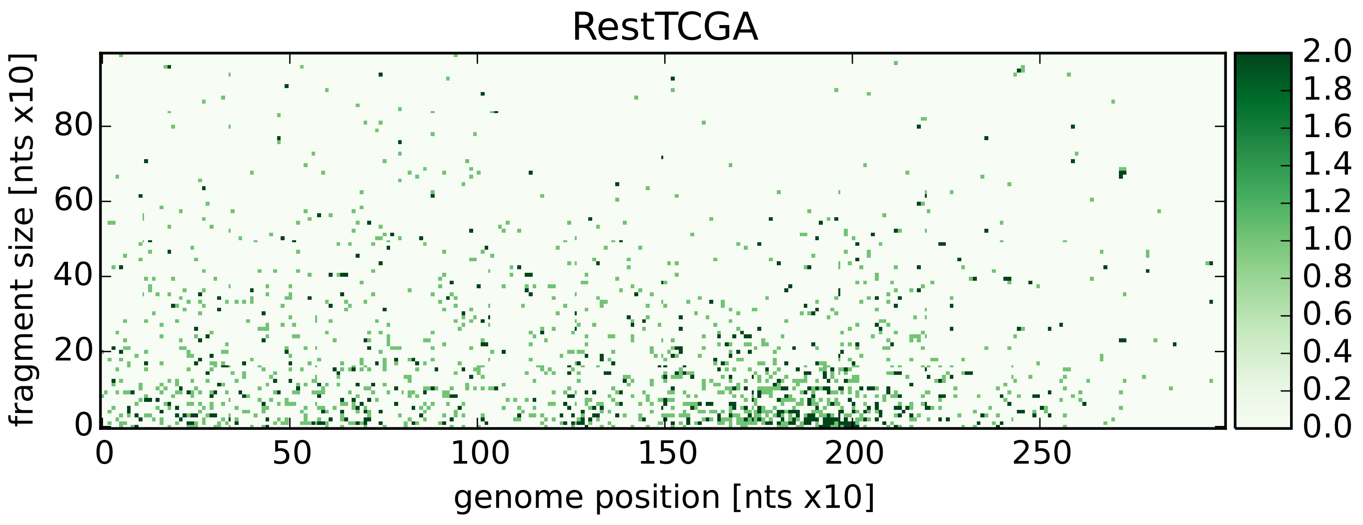
RestTCGA
fragment size [nts x10]
genome position [nts x10]
0
20
40
60
80
0
50
100
150
200
250
2.0
1.8
1.6
1.4
1.2
1.0
0.8
0.6
0.4
0.2
0.0

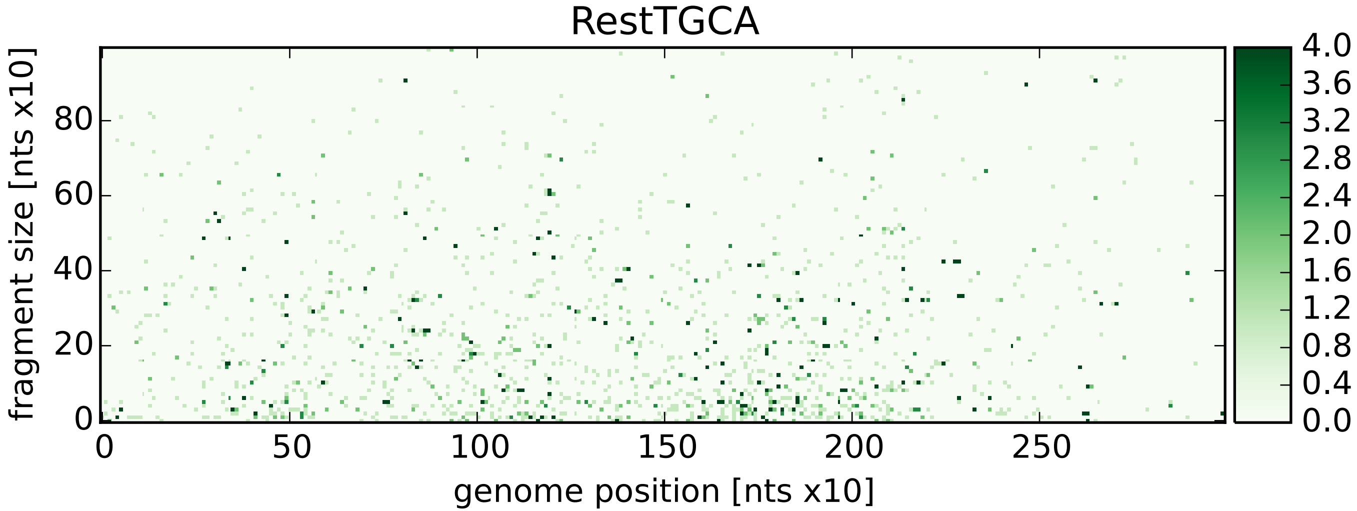

RestTGCA
fragment size [nts x10]
0
20
40
60
80
0
50
100
150
200
250
genome position [nts x10]
4.0
3.6
3.2
2.8
2.4
2.0
1.6
1.2
0.8
0.4
0.0

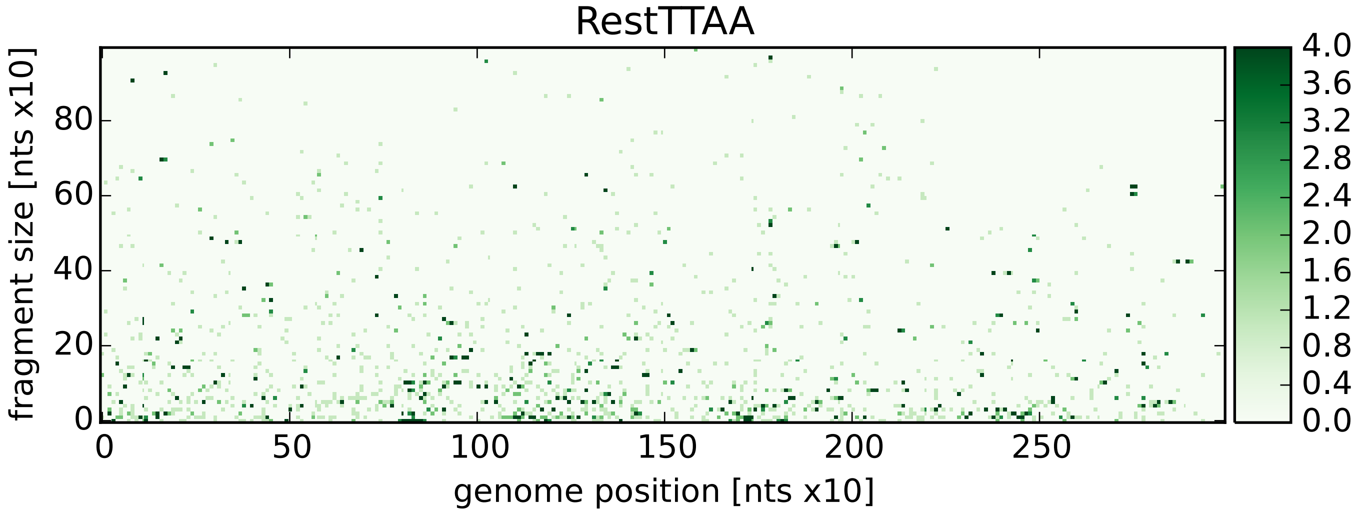
RestTTAA
fragment size [nts x10]
genome position [nts x10]
0
20
40
60
80
0
50
100
150
200
250
0.0
0.4
0.8
1.2
1.6
2.0
2.4
2.8
3.2
3.6
4.0
