# Supplementary material for: Barcoding of Plant Viruses with Circular Single-Stranded DNA Based on Rolling Circle Amplification
Source: Viruses. 2018 Aug 31;10(9):469. doi: 10.3390/v10090469 (PMC6164888; doi:10.3390/v10090469)
Supplement: Supplementary file 1 [file viruses-10-00469-s001.zip › 3-viruses-345336-supplymentary/Jeske18R_Table S1-R1.pdf]

Ref	disease/virus	host plant	country
Akmal et al., 2017	cotton leaf curl	Gossypium hirsutum	India
Al Rwahnih et al., 2013	red blotch	grapevine	California
Ali-Shtayeh et al., 2014a	squash leaf curl	cucurbit	Palestine
Ali-Shtayeh et al., 2014b	watermelon chlorotic stunt	melon, tomato	Palestine
Ammara et al., 2015	begomo, bSat	basil	Oman
Anwar, 2017	tomato leaf curl New Delhi, aSat, bSat	cucurbit	Pakistan
Aranha et al., 2011	Sida micrantha mosaic	okra	Brazil
Bach and Jeske, 2014	beet curly top	sugar beet	Germany
Bang et al., 2014	tomato yellow leaf curl	tomato	South Korea
Bernardo et al., 2013	gemini	several	several
Bernardo et al., 2016	capula	Alfalfa, Euphorbia	France, South Africa
Blinkova et al., 2010	circular DNA	environmental	Tanzania
Boukari et al., 2017	mastre	sugarcane	Florida, Guadeloupe, Reunion
Briddon et al., 2010	turnip curly top	turnip	Iran
Carvajal-Yepes et al., 2017	bean leaf crumple	common bean	Colombia
Chen et al., 2015	mastre	maize	China
Coco et al., 2013	soybean chlorotic spot	soybean	Brazil
Dayaram et al., 2012	cassava associated circular DNA	cassava/fungi	Ghana
Delwart and Li, 2012	circoviridae	several	several
Deuschle et al., 2016	Abutilon mosaic, tomato yellow leaf curl	Nicotiana benthamiana	Germany
Dominguez-Duran et al., 2018	watermelon chlorotic stunt	cucurbits	Western Hemisphere
Dugdale et al., 2014	in-plant activation cassette	tobacco	
Fernandes et al., 2009	bean golden mosaic, Sida micrantha mosaic, okra mottle	soybean	Brazil
Ferreira et al., 2008	construction of agroinfectious begomo, clones		
Ferro et al., 2017	begomo, aSat	Sida, Leonurus spp	Brazil
Fischer et al., 2015	Abutilon mosaic	Abutilon spp.	Germany
Geetanjali et al., 2013	sweet potato leaf curl	Ipomoea purpurea	India
Godara et al., 2017	cotton leaf curl, Sats	cotton	India
Grigoras et al., 2009	faba bean necrotic stunt	faba bean	Ethiopia
Gronenborn et al., 2018	coconut foliar decay	palms	Vanuatu
Guenoune-Gelbart et al., 2010	bean golden yellow mosaic, cabbage leaf curl, squash leaf curl, tomato mottle, watermelon chlorotic stunt, tomato yellow leaf curl	bean, tomato, watermelon	Israel
Haible et al., 2006	Abutilon mosaic, African cassava mosaic, tomato golden mosaic, tomato yellow leaf curl Sardinia, Sri Lankan cassava mosaic, Indian cassava mosaic	Abution spp., cassava, N. benthamiana	Germany, Brazil, India
Herrera et al., 2015	begomo	Oxalis debilis	several
Heydarnejad et al., 2018	turnip leaf curl	turnip	Iran
Homs et al., 2008	beet curly top, mitochondrial plasmid	sugar beet	Germany
Horn et al., 2011	beet curly top, D-DNAs	sugar beet	Germany
Idris et al., 2014	begomo	several	several
Idris et al., 2012	leaf curl, bSats	Solanaceae	Southwest Arabia
Inoue-Nagata et al., 2004	begomo	tomato	Brazil
Jeske et al., 2010	Sida micrantha mosaic	Sida spp; Malva spp	Brazil, Germany
Jeske et al., 2014	begomo, aSat	Sida spp; Malva spp	Cuba
Just et al., 2014	tomato yellow leaf curl	tomato	northern Europe
Just et al., 2017	tomato yellow leaf curl	tomato	Estonia
Jyothsna et al., 2013	Abutilon mosaic	Abutilon	India
Kamaal et al., 2013	begomo, bSat	French bean	India
Kamali et al., 2016	turncurto	turnip	India
Kanakala et al., 2013	chickpea stunt	chickpea	India
Kardani et al., 2013	beet curly top Iran	several	Iran
Khatri et al., 2014	begomo, bSat	rose	Pakistan
Kil et al., 2014	tomato yellow leaf curl	sweet pepper	South Korea
Kleinow et al., 2009	Abutilon mosaic	N. benthamiana	Germany
Knierim and Maiss, 2007	tomato yellow leaf curl Thailand, tobacco leaf curl Thailand	tomato, tobacco	Thailand
Krenz et al., 2011	Abutilon mosaic	N. benthamiana	Germany

Krenz et al., 2010	Abutilon mosaic	N. benthamiana	Germany
Kumar et al., 2018	croton yellow vein mosaic, bSat	Crambe spp	India
Kumar et al., 2010a	cotton leaf curl	cotton	India
Kumar et al., 2013	begomo; bSat	carrot	India
Kumar et al., 2012	mastre	wheat	India
Kumar et al., 2016	leaf curl	Parthenium hysterophorus	India
Kumar et al., 2010b	Ageratum enation, aSat	Zinnia	India
Kumar et al., 2008, 2011	begomo, Ageratum enation	tomato, Crassocephalum crepidioides, Ageratum conyzoides	India
Kushawaha et al., 2015	Sri Lankan cassava mosaic	N. benthamiana	India
Leke et al., 2016	begomo	cotton	Benin
Liang et al., 2015	apple gemini	apple trees	China
Ma et al., 2015	mulberry mosaic	Chinese mulberry tree	China
Macedo et al., 2018	tomato leaf curl purple vein	tomato	Brazil
Marquez-Martin et al., 2011	tomato leaf deformation	tomato	Brazil
Matic et al., 2012	cassava mosaic	cassava	Angola
Mendoza-Figueroa et al., 2018	tomato yellow leaf curl	N. benthamiana	
Mishra et al., 2017	several	Okra	India
Mulabisana et al., 2018	several	sweet potato	South Africa
Nagendran et al., 2016	begomo	ivy gourd	India
Nahid et al., 2008	chickpea stunt	chickpea	Pakistan
Naimuddin et al., 2016	begomo	lentil	India
Nawaz-Ul-Rehman et al., 2012	several	cotton	Pakistan
Nehra and Gaur, 2015	chilli leaf curl	Petunia	India
Oteng-Frimpong et al., 2012	cassava mosaic	cassava	Ghana
Owor et al., 2007	maize streak	maize	Africa
Packialakshmi et al., 2010	begomo, bSat	Vernonia cinerea	India
Packialakshmi and Usha, 2011	Vernonia yellow vein	Vernonia cinerea	India
Pandey et al., 2010	begomo	tomato	India
Paprotka et al., 2010a	sweet potato gemini, mitochondrial plasmids	sweet potato	Brazil
Paprotka et al., 2011	Abutilon mosaic, tomato yellow leaf curl, African cassava mosaic	N. benthamiana	Germany
Paprotka et al., 2010b	Euphorbia yellow mosaic, Cleome leaf crumple	Euphorbia spp; Cleome spp.	Brazil
Paprotka et al., 2010c	Abutilon mosaic	Abutilon	Brazil
Parizipour et al., 2017	wheat dwarf	several	Iran
Park et al., 2011	tobacco leaf curl	tomato	South Korea
Passos et al., 2017	begomo	Macroptilium lathyroides	Brazil
Paz-Carrasco et al., 2014	begomo	tomato	Ecuador
Pearson et al., 2016	several	wastewater treatment plant	US
Perry et al., 2018	grablo	grapevine	US
Pramesh et al., 2013	croton yellow vein	tomato	India
Priyavathi et al., 2016	yellow vein mosaic	bhendi	India
Ramsell et al., 2009	wheat dwarf	barley	Sweden
Regnard et al., 2017	beak and feather	mammalian, N. benthamiana	South Africa
Richter et al., 2014	Euphorbia yellow mosaic, Cleome leaf crumple	N. benthamiana	Germany
Rishishwar et al., 2015	bhendi yellow vein mosaic	okra	India
Rocha et al., 2012	tomato severe rugose	Capsicum spp.	Brazil
Rosario et al., 2012	several	dragonflies	several
Rosario et al., 2009	circov-like	environmental	US
Roy et al., 2013a	leaf curl, bSat	rapeseed-mustard	India
Roy et al., 2013b	tomato leaf curl New Delhi	ash gourd	India
Sahu et al., 2015	begomo, bSat	spinach	India
Satya et al., 2013	yellow mosaic	grain legumes	India
Schubert et al., 2007	wheat dwarf	wheat, barley, oat	Germany
Shahid et al., 2017	mungbean yellow mosaic Indian	Phaseolus vulgaris	Oman
Shahid et al., 2015	tomato yellow leaf curl	Cucurbita maxima	Japan

Sharma et al., 2016	Ageratum enation, aSat	Glycine max	India
Sharma et al., 2015	banana streak	banana	India
Shepherd et al., 2008	several	several	South Africa
Sikorski et al., 2013	myco-like DNA	environmental	New Zealand
Silva et al., 2014	tomato severe rugose, tomato rugose mosaic	tomato	Brazil
Silva et al., 2017a	several	several (in silico)	several
Silva et al., 2017b	several	several (in silico)	several
Silva et al., 2012	several	leguminous weeds	Brazil
Snehi et al., 2011	yellow mosaic	Jatropha gossypifolia	India
Snehi et al., 2012	Indian cassava mosaic	Jatropha curcas	India
Srivastava et al., 2015a	leaf crumple	Jatropha curcas	India
Srivastava et al., 2015b	papaya leaf curl	grain amaranth	India
Srivastava et al., 2015c, d	leaf yellow mosaic	Jatropha curcas	India
Srivastava et al., 2014	yellow mosaic	Jatropha	India
Srivastava et al., 2013	Pedilanthus leaf curl	jasmine	India
Srivastava et al., 2013	Ageratum enation, aSat, bSat	Amaranthus cruentus	India
Sudarshana et al., 2015	grapevine red blotch-associated	grapevine	US
Tang et al., 2017	begomo	Lycianthes biflora	China
Tavares et al., 2012	begomo	Sida spp	Brazil
Tiwari et al., 2010	tomato leaf curl Bangalore, bSat	tomato	India
Tiwari et al., 2013	tomato leaf curl Joydebpur	tomato	India
Tobias et al., 2010	wheat Dwarf	barley	Hungary
Tobias et al., 2011	wheat Dwarf	barley, wheat	Hungary, Ukraine
Ullah et al., 2015	cotton leaf curl Burewala, bSat	eggplant	Pakistan
Ullah et al., 2014	cotton leaf curl	Gossypium arboreum	Pakistan
Valverde et al., 2012	Clerodendron golden mosaic China	Salvia	US
Van Brunschot et al., 2010	tomato yellow leaf curl, D-DNA	tomato	Australia
Varsani et al., 2017	capula, grablo	several	several
Whon et al., 2012	several	environmental	South Korea
Wu et al., 2008	begomo	N. benthamiana	Taiwan
Wyant et al., 2015	begomo	Asystasia gangetica	West Africa
Wyant et al., 2011	Sida micrantha mosaic	Sida spp	Bolivia
Wyant et al., 2012a	tomato golden mosaic	tomato	Brazil
Wyant et al., 2012b	begomo	bean, weeds	Brazil
Yang et al., 2014	tomato yellow leaf curl	tomato	China
Zaffalon et al., 2012	begomo, Sats	cotton	India
Zaim et al., 2011	Velvet bean severe mosaic	Mucuna pruriens	India
Zhang and Ling, 2011	sweet potato begomo	sweet potato	US

References

Akmal, M., Baig, M.S., Khan, J.A., 2017. Suppression of cotton leaf curl disease symptoms in Gossypium hirsutum through over expression of host-encoded miRNAs. J Biotechnol 263, 21-29.

Al Rwahnih, M., Dave, A., Anderson, M.M., Rowhani, A., Uyemoto, J.K., Sudarshana, M.R., 2013. Association of a DNA virus with grapevines affected by red blotch disease in California. Phytopathology 103, 1069-1076.

Ali-Shtayeh, M.S., Jamous, R.M., Hussein, E.Y., Mallah, O.B., Abu-Zeitoun, S.Y., 2014a. Squash leaf curl virus (SLCV): a serious disease threatening cucurbits production in Palestine. Virus genes 48, 320-328.

Ali-Shtayeh, M.S., Jamous, R.M., Mallah, O.B., Abu-Zeitoun, S.Y., 2014b. Molecular Characterization of Watermelon Chlorotic Stunt Virus (WmCSV) from Palestine. Viruses-Basel 6, 2444-2462.

Ammara, U.E., Al-Ansari, M., Al-Shihi, A., Amin, I., Mansoor, S., Al-Maskari, A.Y., Al-Sadi, A.M., 2015. Association of three begomoviruses and a betasatellite with leaf curl disease of basil in Oman. Can J Plant Pathol 37, 506-513.

Anwar, S., 2017. Distinct association of an alphasatellite and a betasatellite with Tomato leaf curl New Delhi virus in field-infected cucurbit. J Gen Plant Pathol 83, 185-188.

Aranha, S.D., de Albuquerque, L.C., Boiteux, L.S., Inoue-Nagata, A.K., 2011. Detection and complete genome characterization of a begomovirus infecting okra (Abelmoschus esculentus) in Brazil. Trop Plant Pathol 36, 14-20.

Bach, J., Jeske, H., 2014. Defective DNAs of beet curly top virus from long-term survivor sugar beet plants. Virus Res 183, 89-94.

Bang, B., Lee, J., Kim, S., Park, J., Nguyen, T.T., Seo, Y.S., 2014. A Rapid and Efficient Method for Construction of an Infectious Clone of Tomato yellow leaf curl virus. Plant Pathology J 30, 310-315.

Bernardo, P., Golden, M., Akram, M., Naimuddin, Nadarajan, N., Fernandez, E., Granier, M., Rebelo, A.G., Peterschmitt, M., Martin, D.P., Roumagnac, P., 2013. Identification and characterisation of a highly divergent geminivirus: Evolutionary and taxonomic implications. Virus Res 177, 35-45.

Bernardo, P., Muhire, B., Francois, S., Deshoux, M., Hartnady, P., Farkas, K., Kraberger, S., Filloux, D., Fernandez, E., Galzi, S., Ferdinand, R., Granier, M., Marais, A., Blasco, P.M., Candresse, T., Escriu, F., Varsani, A., Harkins, G.W., Martin, D.P., Roumagnac, P., 2016. Molecular characterization and prevalence of two capulaviruses: Alfalfa leaf curl virus from France and Euphorbia caput-medusae latent virus from South Africa. Virology 493, 142-153.

Blinkova, O., Victoria, J., Li, Y.Y., Keele, B.F., Sanz, C., Ndjango, J.B.N., Peeters, M., Travis, D., Lonsdorf, E.V., Wilson, M.L., Pusey, A.E., Hahn, B.H., Delwart, E.L., 2010. Novel circular DNA viruses in stool samples of wild-living chimpanzees. Journal of General Virology 91, 74-86.

Boukari, W., Alcala-Briseno, R.I., Kraberger, S., Fernandez, E., Filloux, D., Daugrois, J.H., Comstock, J.C., Lett, J.M., Martin, D.P., Varsani, A., Roumagnac, P., Polston, J.E., Rott, P.C., 2017. Occurrence of a novel mastrevirus in sugarcane germplasm collections in Florida, Guadeloupe and Reunion. Virol J 14.

Briddon, R.W., Heydarnejad, J., Khosrowfar, F., Massumi, H., Martin, D.P., Varsani, A., 2010. Turnip curly top virus, a highly divergent geminivirus infecting turnip in Iran. Virus Res 152, 169-175.

Carvajal-Yepes, M., Zambrano, L., Bueno, J.M., Raatz, B., Cuellar, W.J., 2017. Complete genome sequence of bean leaf crumple virus, a novel begomovirus infecting common bean in Colombia. Archives of virology 162, 1773-1776.

Chen, S., Huang, Q.Q., Wu, L.Q., Qian, Y.J., 2015. Identification and characterization of a maize-associated mastrevirus in China by deep sequencing small RNA populations. Virol J 12.

Coco, D., Calil, I.P., Brustolini, O.J.B., Santos, A.A., Inoue-Nagata, A.K., Fontes, E.P.B., 2013. Soybean chlorotic spot virus, a novel begomovirus infecting soybean in Brazil. Archives of virology 158, 457-462.

Dayaram, A., Opong, A., Jaschke, A., Hadfield, J., Baschiera, M., Dobson, R.C.J., Offei, S.K., Shepherd, D.N., Martin, D.P., Varsani, A., 2012. Molecular characterisation of a novel cassava associated circular ssDNA virus. Virus Res 166, 130-135.

Delwart, E., Li, L.L., 2012. Rapidly expanding genetic diversity and host range of the Circoviridae viral family and other Rep encoding small circular ssDNA genomes. Virus Res 164, 114-121.

Deuschle, K., Kepp, G., Jeske, H., 2016. Differential methylation of the circular DNA in geminiviral minichromosomes. Virology 499, 243-258.

Dominguez-Duran, G., Rodriguez-Negrete, E.A., Morales-Aguilar, J.J., Camacho-Beltran, E., Romero-Romero, J.L., Rivera-Acosta, M.A., Leyva-Lopez, N.E., Arroyo-Becerra, A., Mendez-Lozano, J., 2018. Molecular and biological characterization of Watermelon chlorotic stunt virus (WmCSV): An Eastern Hemisphere begomovirus introduced in the Western Hemisphere. Crop Prot 103, 51-55.

Dugdale, B., Mortimer, C.L., Kato, M., James, T.A., Harding, R.M., Dale, J.L., 2014. Design and construction of an in-plant activation cassette for transgene expression and recombinant protein production in plants. Nat Protoc 9, 1010-1027.

Fernandes, F.R., Cruz, A.R.R., Faria, J.C., Zerbini, F.M., Aragao, F.J.L., 2009. Three distinct begomoviruses associated with soybean in central Brazil. Archives of virology 154, 1567-1570.

Ferreira, P.D.T.D.O., Lemos, T.O., Nagata, T., Inoue-Nagata, A.K., 2008. One-step cloning approach for construction of agroinfectious begomovirus clones. J Virol Methods 147, 351-354.

Ferro, C.G., Silva, J.P., Xavier, C.A.D., Godinho, M.T., Lima, A.T.M., Mar, T.B., Lau, D., Zerbini, F.M., 2017. The ever increasing diversity of begomoviruses infecting non-cultivated hosts: new species from Sida spp. and Leonurus sibiricus, plus two New World alphasatellites. Ann Appl Biol 170, 204-218.

Fischer, A., Strohmeier, S., Krenz, B., Jeske, H., 2015. Evolutionary liberties of the Abutilon mosaic virus cluster. Virus Genes 50, 63-70.

Geetanjali, A.S., Shilpi, S., Mandal, B., 2013. Natural association of two different betasatellites with Sweet potato leaf curl virus in wild morning glory (Ipomoea purpurea) in India. Virus genes 47, 184-188.

Godara, S., Khurana, S.M.P., Biswas, K.K., 2017. Three variants of cotton leaf curl begomoviruses with their satellite molecules are associated with cotton leaf curl disease aggravation in New Delhi. J Plant Biochem Biot 26, 97-105.

Grigoras, I., Timchenko, T., Katul, L., Grande-Perez, A., Vetten, H.J., Gronenborn, B., 2009. Reconstitution of Authentic Nanovirus from Multiple Cloned DNAs. J Virol 83, 10778-10787.

Gronenborn, B., Randles, J.W., Knierim, D., Barriere, Q., Vetten, H.J., Warthmann, N., Cornu, D., Sileye, T., Winter, S., Timchenko, T., 2018. Analysis of DNAs associated with coconut foliar decay disease implicates a unique single-stranded DNA virus representing a new taxon. Sci Rep-Uk 8.

Guenoune-Gelbart, D., Sufrin-Ringwald, T., Capobianco, H., Gaba, V., Polston, J.E., Lapidot, M., 2010. Inoculation of plants with begomoviruses by particle bombardment without cloning: Using rolling circle amplification of total DNA from infected plants and whiteflies. J Virol Methods 168, 87-93.

Haible, D., Kober, S., Jeske, H., 2006. Rolling circle amplification revolutionizes diagnosis and genomics of geminiviruses. J Virol Methods 135, 9-16.

Herrera, F., Aboughanem-Sabanadzovic, N., Valverde, R.A., 2015. A begomovirus associated with yellow vein symptoms of Oxalis debilis. Eur J Plant Pathol 142, 203-208.

Heydarnejad, J., Kamali, M., Hassanvand, V., Massumi, H., Shamshiri, M., Varsani, A., 2018. Turnip leaf curl disease associated with two begomoviruses in south-eastern Iran. Trop Plant Pathol 43, 165-169.

Homs, M., Kober, S., Kepp, G., Jeske, H., 2008. Mitochondrial plasmids of sugar beet amplified via rolling circle method detected during curtovirus screening. Virus Res 136, 124-129.

Horn, J., Lauster, S., Krenz, B., Kraus, J., Frischmuth, T., Jeske, H., 2011. Ambivalent effects of defective DNA in beet curly top virus-infected transgenic sugarbeet plants. Virus Res 158, 169-178.

Idris, A., Al-Saleh, M., Piatek, M.J., Al-Shahwan, I., Ali, S., Brown, J.K., 2014. Viral Metagenomics: Analysis of Begomoviruses by Illumina High-Throughput Sequencing. Viruses-Basel 6, 1219-1236.

Idris, A.M., Abdullah, N.M., Brown, J.K., 2012. Leaf curl diseases of two solanaceous species in Southwest Arabia are caused by a monopartite begomovirus evolutionarily most closely related to a species from the Nile Basin and unique suite of betasatellites. Virus Res 169, 296-300.

Inoue-Nagata, A.K., Albuquerque, L.C., Rocha, W.B., Nagata, T., 2004. A simple method for cloning the complete begomovirus genome using the bacteriophage phi 29 DNA polymerase. J Virol Methods 116, 209-211.

Jeske, H., Gotthardt, D., Kober, S., 2010. In planta cloning of geminiviral DNA: The true Sida micrantha mosaic virus. J Virol Methods 163, 301-308.

Jeske, H., Kober, S., Schafer, B., Strohmeier, S., 2014. Circomics of Cuban geminiviruses reveals the first alpha-satellite DNA in the Caribbean. Virus Genes 49, 312-324.

Just, K., Leke, W.N., Sattar, M.N., Luik, A., Kvarnheden, A., 2014. Detection of Tomato yellow leaf curl virus in imported tomato fruit in northern Europe. Plant Pathol 63, 1454-1460.

Just, K., Sattar, M.N., Arif, U., Luik, A., Kvarnheden, A., 2017. Infectivity of Tomato yellow leaf curl virus isolated from imported tomato fruit in Estonia. Zemdirbyste 104, 47-52.

Jyothsna, P., Haq, Q.M.I., Jayaprakash, P., Malathi, V.G., 2013. Molecular Evidence for the Occurrence of Abutilon mosaic virus, A New World Begomovirus in India. Indian J Virol 24, 284-288.

amaal, N., Akram, M., Pratap, A., Yadav, P., 2013. Characterization of a new begomovirus and a beta satellite associated with the leaf curl disease of French bean in northern India. Virus genes 46, 120-127.

Kamali, M., Heydarnejad, J., Massumi, H., Kvarnheden, A., Kraberger, S., Varsani, A., 2016. Molecular diversity of turncurtoviruses in Iran. Arch Virol 161, 551-561.

Kanakala, S., Sakhare, A., Verma, H.N., Malathi, V.G., 2013. Infectivity and the phylogenetic relationship of a mastrevirus causing chickpea stunt disease in India. Eur J Plant Pathol 135, 429-438.

Kardani, S.G., Heydarnejad, J., Zakiaghl, M., Mehrvar, M., Kraberger, S., Varsani, A., 2013. Diversity of Beet curly top Iran virus isolated from different hosts in Iran. Virus genes 46, 571-575.

Khatri, S., Nahid, N., Fauquet, C.M., Mubin, M., Nawaz-ul-Rehman, M.S., 2014. A betasatellite-dependent begomovirus infects ornamental rose: characterization of begomovirus infecting rose in Pakistan. Virus Genes 49, 124-131.

Kil, E.J., Byun, H.S., Kim, S., Kim, J., Park, J., Cho, S., Yang, D.C., Lee, K.Y., Choi, H.S., Kim, J.K., Lee, S., 2014. Sweet pepper confirmed as a reservoir host for tomato yellow leaf curl virus by both agro-inoculation and whitefly-mediated inoculation. Archives of virology 159, 2387-2395.

Kleinow, T., Nischang, M., Beck, A., Kratzer, U., Tanwir, F., Preiss, W., Kepp, G., Jeske, H., 2009. Three C-terminal phosphorylation sites in the Abutilon mosaic virus movement protein affect symptom development and viral DNA accumulation. Virology 390, 89-101.

Knierim, D., Maiss, E., 2007. Application of Phi29 DNA polymerase in identification and full-length clone inoculation of tomato yellow leaf curl Thailand virus and tobacco leaf curl Thailand virus. Arch Virol 152, 941-954.

Krenz, B., Neugart, F., Kleinow, T., Jeske, H., 2011. Self-interaction of Abutilon mosaic virus replication initiator protein (Rep) in plant cell nuclei. Virus Res 161, 194-197.

Krenz, B., Wege, C., Jeske, H., 2010. Cell-free construction of disarmed Abutilon mosaic virus-based gene silencing vectors. J Virol Methods 169, 129-137.

Kumar, A., Bag, M.K., Singh, R., Jailani, A.A.K., Mandal, B., Roy, A., 2018. Natural infection of croton yellow vein mosaic virus and its cognate betasatellite in germplasm of different Crambe spp in India. Virus Res 243, 60-64.

Kumar, A., Kumar, J., Khan, J.A., 2010a. Sequence characterization of cotton leaf curl virus from Rajasthan: phylogenetic relationship with other members of geminiviruses and detection of recombination. Virus Genes 40, 282-289.

Kumar, J., Gunapati, S., Singh, S.P., Gadre, R., Sharma, N.C., Tuli, R., 2013. Molecular characterization and pathogenicity of a carrot (Daucus carota) infecting begomovirus and associated betasatellite from India. Virus Res 178, 478-485.

Kumar, J., Singh, S.P., Kumar, J., Tuli, R., 2012. A novel mastrevirus infecting wheat in India. Archives of virology 157, 2031-2034.

Kumar, S., Srivastava, A., Jaidi, M., Chauhan, P.S., Raj, S.K., 2016. Molecular Characterization of a Begomovirus, alpha-Satellite, and beta-Satellite Associated with Leaf Curl Disease of Parthenium hysterophorus in India. Plant Dis 100, 2299-2305.

Kumar, Y., Bhardwaj, P., Hallan, V., Zaidi, A.A., 2010b. Detection and characterization of Ageratum enation virus and a nanovirus-like satellite DNA1 from zinnia causing leaf curl symptoms in India. J Gen Plant Pathol 76, 395-398.

Kumar, Y., Hallan, V., Zaidi, A.A., 2008. Molecular characterization of a distinct bipartite begomovirus species infecting tomato in India. Virus Genes 37, 425-431.

Kumar, Y., Hallan, V., Zaidi, A.A., 2011. First report of Ageratum enation virus infecting Crassocephalum crepidioides (Benth.) S. Moore and Ageratum conyzoides L. in India. J Gen Plant Pathol 77, 214-216.

Kushawaha, A.K., Rabindran, R., Dasgupta, I., 2015. Phylogenetic analysis and biolistic infectivity of a cloned Sri Lankan cassava mosaic virus DNA-A from Tamil Nadu, India on Nicotiana benthamiana. Acta Virol 59, 57-63.

Leke, W.N., Khatabi, B., Mignouna, D.B., Brown, J.K., Fondong, V.N., 2016. Complete genome sequence of a new bipartite begomovirus infecting cotton in the Republic of Benin in West Africa. Archives of virology 161, 2329-2333.

Liang, P.B., Navarro, B., Zhang, Z.X., Wang, H.Q., Lu, M.G., Xiao, H., Wu, Q.F., Zhou, X.P., Di Serio, F., Li, S.F., 2015. Identification and characterization of a novel geminivirus with a monopartite genome infecting apple trees. Journal of General Virology 96, 2411-2420.

Ma, Y.X., Navarro, B., Zhang, Z.X., Lu, M.G., Zhou, X.P., Chi, S.Q., Di Serio, F., Li, S.F., 2015. Identification and molecular characterization of a novel monopartite geminivirus associated with mulberry mosaic dwarf disease. Journal of General Virology 96, 2421-2434.

Macedo, M.A., Albuquerque, L.C., Maliano, M.R., Souza, J.O., Rojas, M.R., Inoue-Nagata, A.K., Gilbertson, R.L., 2018. Characterization of tomato leaf curl purple vein virus, a new monopartite New World begomovirus infecting tomato in Northeast Brazil. Archives of virology 163, 737-743.

Marquez-Martin, B., Aragon-Caballero, L., Fiallo-Olive, E., Navas-Castillo, J., Moriones, E., 2011. Tomato leaf deformation virus, a novel begomovirus associated with a severe disease of tomato in Peru. Eur J Plant Pathol 129, 1-7.

Matic, S., da Cunha, A.T.P., Thompson, J.R., Tepfer, M., 2012. An Analysis of Viruses Associated with Cassava Mosaic Disease in Three Angolan Provinces. J Plant Pathol 94, 443-450.

Mendoza-Figueroa, J.S., Kvarnheden, A., Mendez-Lozano, J., Rodriguez-Negrete, E.A., de los Monteros, R.A.E., Soriano-Garcia, M., 2018. A peptide derived from enzymatic digestion of globulins from amaranth shows strong affinity binding to the replication origin of Tomato yellow leaf curl virus reducing viral replication in Nicotiana benthamiana. Pestic Biochem Phys 145, 56-65.

Mishra, G.P., Singh, B., Seth, T., Singh, A.K., Halder, J., Krishnan, N., Tiwari, S.K., Singh, P.M., 2017. Biotechnological Advancements and Begomovirus Management in Okra (Abelmoschus esculentus L.): Status and Perspectives. Front Plant Sci 8.

Mulabisana, M.J., Cloete, M., Mabasa, K.G., Laurie, S.M., Oelofse, D., Esterhuizen, L.L., Rey, M.E.C., 2018. Surveys in the Gauteng, Limpopo and Mpumalanga provinces of South Africa reveal novel isolates of sweet potato viruses. S Afr J Bot 114, 280-294.

Nagendran, K., Satya, V.K., Mohankumar, S., Karthikeyan, G., 2016. Molecular characterization of a distinct bipartite Begomovirus species infecting ivy gourd (Coccinia grandis L.) in Tamil Nadu, India. Virus genes 52, 146-151.

Nahid, N., Amin, I., Mansoor, S., Rybicki, E.P., van der Walt, E., Briddon, R.W., 2008. Two dicot-infecting mastreviruses (family Geminiviridae) occur in Pakistan. Archives of virology 153, 1441-1451.

Naimuddin, K., Akram, M., Agnihotri, A.K., 2016. Molecular characterization of a first begomovirus associated with lentil (Lens culinaris) from India. Acta Virol 60, 217-223.

Nawaz-Ul-Rehman, M.S., Briddon, R.W., Fauquet, C.M., 2012. A Melting Pot of Old World Begomoviruses and Their Satellites Infecting a Collection of Gossypium Species in Pakistan. Plos One 7.

Nehra, C., Gaur, R.K., 2015. Molecular characterization of Chilli leaf curl viruses infecting new host plant Petunia hybrida in India. Virus genes 50, 58-62.

Oteng-Frimpong, R., Levy, Y., Torkpo, S.K., Danquah, E.Y., Offei, S.K., Gafni, Y., 2012. Complete genome sequencing of two causative viruses of cassava mosaic disease in Ghana. Acta Virol 56, 305-314.

Owor, B.E., Shepherd, D.N., Taylor, N.J., Edema, R., Monjane, A.L., Thomson, J.A., Martin, D.P., Varsani, A., 2007. Successful application of FTA((R)) Classic Card technology and use of bacteriophage phi 29 DNA polymerase for large-scale field sampling and cloning of complete maize streak virus genomes. J Virol Methods 140, 100-105.

Packialakshmi, R.M., Srivastava, N., Girish, K.R., Usha, R., 2010. Molecular characterization of a distinct begomovirus species from Vernonia cinerea and its associated DNA-beta using the bacteriophage I broken vertical bar 29 DNA polymerase. Virus genes 41, 135-143.

Packialakshmi, R.M., Usha, R., 2011. A simple and efficient method for agroinfection of Vernonia cinerea with infectious clones of Vernonia yellow vein virus. Virus genes 43, 465-470.

Pandey, P., Mukhopadhya, S., Naqvi, A.R., Mukherjee, S.K., Shekhawat, G.S., Choudhury, N.R., 2010. Molecular characterization of two distinct monopartite begomoviruses infecting tomato in india. Virology Journal 7.

Paprotka, T., Boiteux, L.S., Fonseca, M.E.N., Resende, R.O., Jeske, H., Faria, J.C., Ribeiro, S.G., 2010a. Genomic diversity of sweet potato geminiviruses in a Brazilian germplasm bank. Virus Res 149, 224-233.

Paprotka, T., Deuschle, K., Metzler, V., Jeske, H., 2011. Conformation-Selective Methylation of Geminivirus DNA. J Virol 85, 12001-12012.

Paprotka, T., Metzler, V., Jeske, H., 2010b. The complete nucleotide sequence of a new bipartite begomovirus from Brazil infecting Abutilon. Archives of virology 155, 813-816.

Paprotka, T., Metzler, V., Jeske, H., 2010c. The first DNA 1-like alpha satellites in association with New World begomoviruses in natural infections. Virology 404, 148-157.

Parizipour, M.H.G., Schubert, J., Behjatnia, S.A.A., Afsharifar, A., Habekuss, A., Wu, B.L., 2017. Phylogenetic analysis of Wheat dwarf virus isolates from Iran. Virus genes 53, 266-274.

Park, J., Lee, H., Kim, M.K., Kwak, H.R., Auh, C.K., Lee, K.Y., Kim, S., Choi, H.S., Lee, S., 2011. Phylogenetic lineage of Tobacco leaf curl virus in Korea and estimation of recombination events implicated in their sequence variation. Virus Res 159, 124-131.

Passos, L.S., Rodrigues, J.S., Soares, E.C.S., Silva, J.P., Zerbini, F.M., Araujo, A.S.F., Beserra, J.E.A., 2017. Complete genome sequence of a new bipartite begomovirus infecting Macroptilium lathyroides in Brazil. Archives of virology 162, 3551-3554.

Paz-Carrasco, L.C., Castillo-Urquiza, G.P., Lima, A.T.M., Xavier, C.A.D., Vivas-Vivas, L.M., Mizubuti, E.S.G., Zerbini, F.M., 2014. Begomovirus diversity in tomato crops and weeds in Ecuador and the detection of a recombinant isolate of rhynchosia golden mosaic Yucatan virus infecting tomato. Archives of virology 159, 2127-2132.

Pearson, V.M., Caudle, S.B., Rokyta, D.R., 2016. Viral recombination blurs taxonomic lines: examination of single-stranded DNA viruses in a wastewater treatment plant. Peerj 4.

Perry, K.L., McLane, H., Thompson, J.R., Fuchs, M., 2018. A novel grablovirus from non-cultivated grapevine (Vitis sp.) in North America. Arch Virol 163, 259-262.

Pramesh, D., Mandal, B., Phaneendra, C., Muniyappa, V., 2013. Host range and genetic diversity of croton yellow vein mosaic virus, a weed-infecting monopartite begomovirus causing leaf curl disease in tomato. Arch Virol 158, 531-542.

Priyavathi, P., Kavitha, V., Gopal, P., 2016. Complex nature of infection associated with yellow vein mosaic disease in Bhendi (Abelmoschus esculentus). Curr Sci India 111, 1511-1515.

Ramsell, J.N.E., Boulton, M.I., Martin, D.P., Valkonen, J.P.T., Kvarnheden, A., 2009. Studies on the host range of the barley strain of Wheat dwarf virus using an agroinfectious viral clone. Plant Pathol 58, 1161-1169.

Regnard, G.L., de Moor, W.R.J., Hitzeroth, I.I., Williamson, A.L., Rybicki, E.P., 2017. Xenogenic rolling-circle replication of a synthetic beak and feather disease virus genomic clone in 293TT mammalian cells and Nicotiana benthamiana. Journal of General Virology 98, 2329-2338.

Richter, K.S., Kleinow, T., Jeske, H., 2014. Somatic homologous recombination in plants is promoted by a geminivirus in a tissue-selective manner. Virology 452, 287-296.

Rishishwar, R., Mazumdar, B., Dasgupta, I., 2015. Diverse and recombinant begomoviruses and various satellites are associated with Bhendi yellow vein mosaic disease of okra in India. J Plant Biochem Biot 24, 470-475.

Rocha, K.C.G., Marubayashi, J.M., Mituti, T., Gioria, R., Kobori, R.F., Melo, A.M.T., Pavan, M.A., Krause-Sakate, R., 2012. Evaluation of resistance to Tomato severe rugose virus (ToSRV) in Capsicum spp. genotypes. Trop Plant Pathol 37, 314-318.

Rosario, K., Dayaram, A., Marinov, M., Ware, J., Kraberger, S., Stainton, D., Breitbart, M., Varsani, A., 2012. Diverse circular ssDNA viruses discovered in dragonflies (Odonata: Epiprocta). Journal of General Virology 93, 2668-2681.

Rosario, K., Duffy, S., Breitbart, M., 2009. Diverse circovirus-like genome architectures revealed by environmental metagenomics. Journal of General Virology 90, 2418-2424.

Roy, A., Spoorthi, P., Bag, M.K., Prasad, T.V., Singh, R., Dutta, M., Mandal, B., 2013a. A Leaf Curl Disease in Germplasm of Rapeseed-Mustard in India: Molecular Evidence of a Weed-Infecting Begomovirus-Betasatellite Complex Emerging in a New Crop. J Phytopathol 161, 522-535.

Roy, A., Spoorthi, P., Panwar, G., Bag, M.K., Prasad, T.V., Kumar, G., Gangopadhyay, K.K., Dutta, M., 2013b. Molecular Evidence for Occurrence of Tomato leaf curl New Delhi virus in Ash Gourd (Benincasa hispida) Germplasm Showing a Severe Yellow Stunt Disease in India. Indian J Virol 24, 74-77.

Sahu, A.K., Nehra, C., Marwal, A., Gaur, R.K., 2015. First report of a begomovirus associated with betasatellites infecting spinach (Spinacia oleracea) in India. J Gen Plant Pathol 81, 146-150.

Satya, V.K., Malathi, V.G., Velazhahan, R., Rabindran, R., Jayamani, P., Alice, D., 2013. Characterization of betasatellite associated with the yellow mosaic disease of grain legumes in Southern India. Acta Virol 57, 405-414.

Schubert, J., Habekuss, A., Kazmaier, K., Jeske, H., 2007. Surveying cereal-infecting geminiviruses in Germany - Diagnostics and direct sequencing using rolling circle amplification. Virus Research 127, 61-70.

Shahid, M.S., Briddon, R.W., Al-Sadi, A.M., 2017. Identification of Mungbean yellow mosaic Indian virus Associated with Tomato Leaf Curl Betasatellite Infecting Phaseolus vulgaris in Oman. J Phytopathol 165, 204-211.

Shahid, M.S., Ikegami, M., Briddon, R.W., Natsuaki, K.T., 2015. Characterization of Tomato yellow leaf curl virus and associated alphasatellite infecting Cucurbita maxima in Japan. J Gen Plant Pathol 81, 92-95.

Sharma, S., Singh, L., Roshan, P., Kulshreshtha, A., Singh, N., Hallan, V., 2016. Complete Nucleotide Sequence of Ageratum enation virus and an Alphasatellite Infecting a New Host Glycine max in India. J Phytopathol 164, 554-557.

Sharma, S.K., Kumar, P.V., Geetanjali, A.S., Pun, K.B., Baranwal, V.K., 2015. Subpopulation level variation of banana streak viruses in India and common evolution of banana and sugarcane badnaviruses. Virus genes 50, 450-465.

Shepherd, D.N., Martin, D.P., Lefeuvre, P., Monjane, A.L., Owor, B.E., Rybicki, E.P., Varsani, A., 2008. A protocol for the rapid isolation of full geminivirus genomes from dried plant tissue. J Virol Methods 149, 97-102.

Sikorski, A., Massaro, M., Kraberger, S., Young, L.M., Smalley, D., Martin, D.P., Varsani, A., 2013. Novel myco-like DNA viruses discovered in the faecal matter of various animals. Virus Res 177, 209-216.

Silva, F.N., Lima, A.T.M., Rocha, C.S., Castillo-Urquiza, G.P., Alves, M., Zerbini, F.M., 2014. Recombination and pseudorecombination driving the evolution of the begomoviruses Tomato severe rugose virus (ToSRV) and Tomato rugose mosaic virus (ToRMV): two recombinant DNA-A components sharing the same DNA-B. Virol J 11.

Silva, J.C.F., Carvalho, T.F.M., Basso, M.F., Deguchi, M., Pereira, W.A., Sobrinho, R.R., Vidigal, P.M.P., Brustolini, O.J.B., Silva, F.F., Dal-Bianco, M., Fontes, R.L.F., Santos, A.A., Zerbini, F.M., Cerqueira, F.R., Fontes, E.P.B., 2017a. Geminivirus data warehouse: a database enriched with machine learning approaches. Bmc Bioinformatics 18.

Silva, J.C.F., Carvalho, T.F.M., Fontes, E.P.B., Cerqueira, F.R., 2017b. Fangorn Forest (F2): a machine learning approach to classify genes and genera in the family Geminiviridae. Bmc Bioinformatics 18.

Silva, S.J.C., Castillo-Urquiza, G.P., Hora, B.T., Assuncao, I.P., Lima, G.S.A., Pio-Ribeiro, G., Mizubuti, E.S.G., Zerbini, F.M., 2012. Species diversity, phylogeny and genetic variability of begomovirus populations infecting leguminous weeds in northeastern Brazil. Plant Pathol 61, 457-467.

Snehi, S.K., Raj, S.K., Khan, M.S., Prasad, V., 2011. Molecular identification of a new begomovirus associated with yellow mosaic disease of Jatropha gossypifolia in India. Archives of virology 156, 2303-2307.

Snehi, S.K., Srivastava, A., Raj, S.K., 2012. Biological Characterization and Complete Genome Sequence of a Possible Strain of Indian cassava mosaic virus from Jatropha curcas in India. J Phytopathol 160, 547-553.

Srivastava, A., Jaidi, M., Kumar, S., Raj, S.K., 2015a. Molecular identification of a new begomovirus associated with leaf crumple disease of Jatropha curcas L. in India. Archives of virology 160, 617-619.

Srivastava, A., Jaidi, M., Kumar, S., Raj, S.K., Shukla, S., 2015b. Association of Papaya leaf curl virus with the leaf curl disease of grain amaranth (Amaranthus cruentus L.) in India. Phytoparasitica 43, 97-101.

Srivastava, A., Kumar, S., Jaidi, M., Raj, S.K., 2015c. Characterization of a novel begomovirus associated with yellow mosaic disease of three ornamental species of Jatropha grown in India. Virus Res 201, 41-49.

Srivastava, A., Kumar, S., Jaidi, M., Raj, S.K., 2015d. Molecular characterization of a new begomovirus associated with leaf yellow mosaic disease of Jatropha curcas in India. Archives of virology 160, 1359-1362.

Srivastava, A., Kumar, S., Raj, S.K., 2014. Association of Pedilanthus leaf curl virus with yellow mottling and leaf curl symptoms in two jasmine species grown in India. J Gen Plant Pathol 80, 370-373.

Srivastava, A., Raj, S.K., Kumar, S., Snehi, S.K., Kulshreshtha, A., Hallan, V., Pande, S.S., 2013. Molecular identification of Ageratum enation virus, betasatellite and alphasatellite molecules isolated from yellow vein diseased Amaranthus cruentus in India. Virus Genes 47, 584-590.

Sudarshana, M.R., Perry, K.L., Fuchs, M.F., 2015. Grapevine Red Blotch-Associated Virus, an Emerging Threat to the Grapevine Industry. Phytopathology 105, 1026-1032.

Tang, Y.F., He, Z.F., Brown, J.K., She, X.M., Lan, G.B., 2017. Molecular characterization of a novel bipartite begomovirus isolated from Lycianthes biflora in China. Archives of virology 162, 2473-2476.

Tavares, S.S., Ramos-Sobrinho, R., Gonzalez-Aguilera, J., Lima, G.S.A., Assuncao, I.P., Zerbini, F.M., 2012. Further Molecular Characterization of Weed-Associated Begomoviruses in Brazil with an Emphasis on Sida Spp. Planta Daninha 30, 305-315.

Tiwari, N., Padmalatha, K.V., Singh, V.B., Haq, Q.M.I., Malathi, V.G., 2010. Tomato leaf curl Bangalore virus (ToLCBV): infectivity and enhanced pathogenicity with diverse betasatellites. Archives of virology 155, 1343-1347.

Tiwari, N., Singh, V.B., Sharma, P.K., Malathi, V.G., 2013. Tomato leaf curl Joydebpur virus: a monopartite begomovirus causing severe leaf curl in tomato in West Bengal. Archives of virology 158, 1-10.

Tobias, I., Kiss, B., Salanki, K., Palkovics, L., 2010. The Nucleotide Sequence of Barley Strain of Wheat Dwarf Virus Isolated in Hungary. Cereal Res Commun 38, 67-74.

Tobias, I., Shevchenko, O., Kiss, B., Bysov, A., Snihur, H., Polischuk, V., Salanki, K., Palkovics, L., 2011. Comparison of the Nucleotide Sequences of Wheat Dwarf Virus (WDV) isolates from Hungary and Ukraine. Pol J Microbiol 60, 125-131.

Ullah, R., Akhtar, K.P., Hassan, I., Saeed, M., Sarwar, N., Mansoor, S., 2015. Evidence of Cotton leaf curl Burewala virus Variant and its Associate Betasatellite Causing Yellow Mosaic of Eggplant (Solanum melongena) in Pakistan. J Phytopathol 163, 233-237.

Ullah, R., Akhtar, K.P., Moffett, P., Mansoor, S., Briddon, R.W., Saeed, M., 2014. An analysis of the resistance of Gossypium arboreum to cotton leaf curl disease by grafting. Eur J Plant Pathol 139, 837-847.

Valverde, R.A., Singh, R., Sabanadzovic, S., 2012. Detection and identification of Clerodendron golden mosaic China virus in Salvia splendens. Eur J Plant Pathol 133, 499-503.

Van Brunschot, S.L., Persley, D.M., Geering, A.D.W., Campbell, P.R., Thomas, J.E., 2010. Tomato yellow leaf curl virus in Australia: distribution, detection and discovery of naturally occurring defective DNA molecules. Australas Plant Path 39, 412-423.

Varsani, A., Roumagnac, P., Fuchs, M., Navas-Castillo, J., Moriones, E., Idris, A., Briddon, R.W., Rivera-Bustamante, R., Zerbini, F.M., Martin, D.P., 2017. Capulavirus and Grablovirus: two new genera in the family Geminiviridae. Archives of virology 162, 1819-1831.

Whon, T.W., Kim, M.S., Roh, S.W., Shin, N.R., Lee, H.W., Bae, J.W., 2012. Metagenomic Characterization of Airborne Viral DNA Diversity in the Near-Surface Atmosphere. J Virol 86, 8221-8231.

Wu, C.Y., Lai, Y.C., Lin, N.S., Hsu, Y.H., Tsai, H.T., Liao, J.Y., Hu, C.C., 2008. A simplified method of constructing infectious clones of begomovirus employing limited restriction enzyme digestion of products of rolling circle amplification. J Virol Methods 147, 355-359.

Wyant, P., Strohmeier, S., Fischer, A., Schafer, B., Briddon, R.W., Krenz, B., Jeske, H., 2015. Light-dependent segregation of begomoviruses in Asystasia gangetica leaves. Virus Res 195, 225-235.

Wyant, P.S., Gotthardt, D., Schafer, B., Krenz, B., Jeske, H., 2011. The genomes of four novel begomoviruses and a new Sida micrantha mosaic virus strain from Bolivian weeds. Archives of virology 156, 347-352.

Wyant, P.S., Kober, S., Schwierzok, A., Kocher, C., Schafer, B., Jeske, H., Wege, C., 2012a. Cloned tomato golden mosaic virus back in tomatoes. Virus Res 167, 397-403.

Wyant, P.S., Strohmeier, S., Schafer, B., Krenz, B., Assuncao, I.P., Lima, G.S.D., Jeske, H., 2012b. Circular DNA genomics (circomics) exemplified for geminiviruses in bean crops and weeds of northeastern Brazil. Virology 427, 151-157.

Yang, X.L., Zhou, M.N., Qian, Y.J., Xie, Y., Zhou, X.P., 2014. Molecular variability and evolution of a natural population of tomato yellow leaf curl virus in Shanghai, China. J Zhejiang Univ-Sc B 15, 133-142.

Zaffalon, V., Mukherjee, S.K., Reddy, V.S., Thompson, J.R., Tepfer, M., 2012. A survey of geminiviruses and associated satellite DNAs in the cotton-growing areas of northwestern India. Arch Virol 157, 483-495.

Zaim, M., Kumar, Y., Hallan, V., Zaidi, A.A., 2011. Velvet bean severe mosaic virus: a distinct begomovirus species causing severe mosaic in Mucuna pruriens (L.) DC. Virus genes 43, 138-146.

Zhang, S.C., Ling, K.S., 2011. Genetic diversity of sweet potato begomoviruses in the United States and identification of a natural recombinant between sweet potato leaf curl virus and sweet potato leaf curl Georgia virus. Archives of virology 156, 955-968.
